# Unveiling the proteomic landscape: Exploring differentially expressed proteins in patients with jaw cysts

**DOI:** 10.1097/MD.0000000000048872

**Published:** 2026-05-22

**Authors:** Xuefeng Wang, Yanbo Zhang, Lingli Lan, Shangzhi Han, Feng Huo, Jiashun Du, Xibo Chen

**Affiliations:** a Department of Stomatology, The Affiliated Hospital of Chengde Medical University, Chengde, China.

**Keywords:** bioinformatics, differential proteins, GO, jaw cysts, KEGG, proteomics

## Abstract

This study aimed to investigate the pathogenic mechanisms of jaw cysts by screening for differentially expressed proteins in the tissues of patients with jaw cysts using proteomic techniques. Tissue samples were collected from 4 patients with jaw cysts and 4 healthy volunteers (control group). Non-labeled proteomic techniques combined with liquid chromatography-mass spectrometry were used to identify differentially expressed proteins between the 2 groups. Proteins showing significant differences in expression were selected and Gene Ontology and Kyoto Encyclopedia of Genes and Genomes (KEGG) pathway enrichment analyses were performed. These conclusions were validated using quantitative reverse transcription polymerase chain reaction (RT-qPCR). To mitigate potential confounding effects, logistic regression analyses were used to adjust for variables. Non-labeled proteomic analysis revealed 214 significantly differentially expressed proteins in the tissues of patients with jaw cysts, including 50 upregulated and 164 downregulated proteins. These differentially expressed proteins were mainly enriched in cellular components, such as the vesicle lumen and glutamatergic synapses. They exhibit molecular functions such as protein tyrosine kinase activity and transmembrane receptor protein tyrosine kinase activity and are primarily involved in biological processes such as the immune response and regulation of neuron projection development. The main KEGG pathways enriched in jaw cysts included thermogenesis and Wnt signaling pathway. Protein network interaction analysis identified 6 core proteins that may be associated with the development of jaw cysts, which were further validated using RT-qPCR. The identified 6 proteins, namely CD79A, CD5, KRR1, UTP20, AATF, and WDR43 – may be closely associated with the pathogenesis of jaw cysts. UTP20, AATF, and CD5 may represent potential candidate biomarkers associated with jaw cysts.

## 1. Introduction

Jaw cysts are pathological cystic lesions characterized by an epithelial lining containing either fluid or semifluid material.^[1,2]^ It can be monocystic or polycystic and is common in the maxillofacial area. Jaw cysts are oral cystic lesions that can develop anywhere within the jawbone. Over time, they expand and invade the surrounding tissues. The compression of neurons by a cyst can lead to symptoms such as facial numbness and deformities, significantly affecting the patient’s daily life. Most jaw cysts are odontogenic and occur more frequently in the jawbone than in other bones due to the presence of neuroectodermal cell remnants.^[3]^

Proteomics is a high-throughput sequencing method that combines liquid chromatography and mass spectrometry to determine protein levels in a sample based on the charge-to-mass ratio.^[4-9]^ Leveraging the advantages of high throughput, precision, and time-efficiency offered by proteomics, this study employed liquid chromatography-mass spectrometry (LC-MS/MS).^[10-14]^ Differentially expressed proteins in patients with jaw cysts were analyzed, along with the biological processes, cellular components, and molecular functions involved in cyst formation. Bioinformatic analysis was conducted to identify the potential signaling pathways implicated in the pathogenesis and development of jaw cysts.^[15,16]^ These findings aim to provide a foundation for the prevention and treatment of jaw cysts.

## 2. Materials and methods

### 2.1. Research participants

A total of 4 patients, including 2 males and 2 females aged between 19 and 48 (mean age 28.5 ± 11.41) years old, diagnosed with jaw cysts at the Affiliated Hospital of Chengde Medical College between January 2020 and December 2022, were selected as the study subjects. The inclusion criteria were as follows: patients diagnosed with jaw cystic lesions based on preoperative CT and x-ray examinations, treated for jaw cysts, and postoperative pathology consistent with odontogenic jaw cystic lesions, such as root cysts and tooth-containing cysts; complete case information available; absence of infectious diseases; and no history of mental disorders, with normal communication or exchange abilities.

The exclusion criteria were: presence of hematologic diseases or coagulation dysfunction; pathological results indicating non-JC disease; presence of systemic diseases affecting bone healing and regeneration, along with severe organ dysfunction; jaw lesions causing pathological fractures. Surgical history of jaw lesions; incomplete clinical data; and history of bisphosphonate use and radiotherapy or chemotherapy within the past 6 months.

Additionally, 4 healthy volunteers with no jaw cysts who underwent physical examinations at our hospital during the same period were selected as controls, including 2 males and 2 females, aged between 14 and 29 (mean age 21.75 ± 5.715) years old. All study participants provided informed consent and the study protocol was reviewed and approved by the hospital’s ethics committee (ethics number: CYFYLL2020264).

### 2.2. Protein extracts

Three formalin-fixed, paraffin-embedded (FFPE) tissue sections were processed for analysis. Each section had a thickness of 10 μm and was placed in a 1.5 mL safety lock tube. Initially, 1 mL of xylene was mixed with xylene at a temperature of 30°C while being stirred at 1500 rpm for 2 minutes. The samples were then centrifuged at 12,000 rpm for 2 minutes at room temperature to remove the supernatant. This step was repeated to ensure effective deparaffinization. Next, the deparaffinized tissue samples were sequentially incubated with 67% and 33% xylene ethanol (v/v), absolute ethanol, and phosphate-buffered saline for 2 minutes each. After each incubation step, the samples were centrifuged to remove the residual xylene and ethanol. The cleaned tissue pellet was then lysed and resuspended using 500 μL of 4% sodium dodecyl sulfate. Sonication was performed for 2 minutes with a 2-second cycle and a 1-second pause to further break down the tissue. Finally, the samples were incubated in a thermomixer at 1500 rpm for 2 hours at a temperature of 95°C. At the end of the incubation period, the protein supernatant was recovered by centrifugation at 12,000 rpm, and its concentration was determined using the BCA method.^[17]^

### 2.3. Sample preparation

The extract was subjected to reduction at 55°C using 10 mM dithiothreitol for a duration of 45 minutes. Subsequently, alkylation was performed using 50 mM iodoacetamide at room temperature in the dark for 30 minutes. The samples were then precipitated using acetone and incubated overnight. After overnight incubation, the mixture was centrifuged at 12,000 rpm for 10 minutes and the resulting pellet was collected and washed with 90% acetone.

Next, the purified pellet was dissolved in 50 mM ammonium bicarbonate and subjected to overnight digestion at 37°C using trypsin in an enzyme-to-protein mass ratio of 1:30. Finally, the hydrolysate underwent desalting using a C18 stage tip and was subsequently dried using a vacuum desiccator at a temperature of 60°C.^[18]^

### 2.4. LC-MS analysis

The samples were analyzed using an LC-MS instrument, which consisted of an EASY-nLC 1000 ultra-high-pressure system coupled with an Orbitrap Fusion Tribrid mass spectrometer (Thermo Fisher Scientific).^[19,20]^ The cleaned peptides were separated on a 25 cm × 75 μm C18 column (Aurora Ultimate, #AUR3-25075C18-CSI) at a temperature of 55°C. Mobile phase A consisted of 99.9% water and 0.1% formic acid (volume ratio), while mobile phase B consisted of 80% acetonitrile, 20% water, and 0.1% formic acid (volume ratio).^[21,22]^ MS analysis was performed using the data-independent acquisition (DIA) mode. This involved an initial MS scan with a resolution of 60,000 in the range of 350 to 1250 m/z. The automatic gain control (AGC) target was set to 1e6 or a maximum of 100 ms.^[23,24]^ Subsequently, 27 DIA rolling segments were acquired at a resolution of 30,000, targeting isolation windows of 17, 23, 29, and 35 m/z. The AGC target for these segments was set to 8e5 (40 ms) to optimize the injection time. The setting “inject ions for all available parallelizable time” was enabled to maximize the efficiency of ion injection. High-energy collision dissociation (HCD) fragmentation was applied with a normalized collision energy of 35%. The spectra were recorded in contour mode, and the default charge state for MS2 was set to 2.

### 2.5. MS database analysis

In this study, we used a library of computer-predicted spectra generated from the UniProt Human Protein Database to search raw data. Precursor ions were generated using default settings, wherein cysteine residues were methylated by carbamoyl as a fixed modification, and methionine residues were oxidized as a variable modification. The peptide length range was set to 7 to 30 amino acids, the precursor ion m/z range was defined as 300 to 1800, and the fragment m/z range was 200 to 1800. During the analysis, we utilized the match-between-runs (lot-to-lot matching) method in combination with a library that was filtered from the DIA data and created a false discovery rate (FDR) of 0.01.^[25,26]^ The algorithm was configured for quantitative analysis by employing a neural network classifier operating in dual-channel mode. The quantitation strategy relied on stable LC (high accuracy) and cross-run homogenization through the retention time (RT).

### 2.6. Screening and analysis of differentially expressed proteins

Statistical analysis of the protein quantification results was performed using *t* tests. Proteins with a fold change (logFC) ≥ 0 and a *P*-value ≤ .05 were defined as significantly differentially expressed proteins.^[27,28]^ Multiple testing correction was not applied at the initial discovery stage; therefore, the identified proteins were treated as exploratory candidates for downstream clustering, enrichment analysis, protein–protein interaction analysis, and subsequent RT-qPCR validation. Hierarchical clustering analysis was conducted on the differentially expressed proteins to group those with similar expression patterns. GO functional annotation, Kyoto Encyclopedia of Genes and Genomes (KEGG) pathway enrichment analysis,^[29,30]^ and protein expression heatmap generation were performed using RStudio software (version 4.3.3; RStudio Inc., Boston).^[31,32]^ Interactions among differentially expressed proteins were analyzed using the STRING online tool (https://cn.string-db.org/), and the protein interaction network was visualized using Cytoscape software (version 3.10.2; Cytoscape Consortium, San Diego).^[33-36]^ Core proteins were analyzed using the maximal clique centrality,^[37-39]^ density of maximum neighborhood component,^[40,41]^ maximum neighborhood component,^[42,43]^ and degree^[44-46]^ algorithms provided by the CytoHubba plugin.^[47,48]^

### 2.7. The significance of core proteins in the diagnosis of jaw cysts

To evaluate the capability of core proteins to differentiate between non-jaw cyst controls and patients with jaw cysts, we used the R software to analyze the expression of these core proteins within the proteomics data. The diagnostic performance of the core proteins was visualized via box plots.

### 2.8. Reverse transcription quantitative PCR validation

Total RNA was extracted using an efficient column-based method and the extracted total RNA was subsequently reverse transcribed into cDNA using a kit sourced from TaKaRa. The RT-qPCR reaction mixture was prepared in a total volume of 25 μL, consisting of 12.5 μL of TB Green Premix Ex Taq II (purchased from TaKaRa), 2 μL of cDNA template, 2 μL of a mixture containing 1 μL each of 10 μmol/L forward and reverse primers (the primer sequences are shown in Table 1), and the volume was adjusted to 25 μL with nuclease-free water. The RT-qPCR amplification protocol was set as follows: an initial denaturation step at 95°C for 30 seconds, followed by 40 cycles of denaturation at 95°C for 5 seconds, annealing and extension at 60°C for 30 seconds. HA067812 gene was selected as the reference gene, and the relative gene expression levels were quantified using the 2^−△△Ct^ method.

**Table 1:** The sequences of genes and internal reference genes are presented.

Gene	Primer sequence (5′–3′)
AATF	F:AGAGAAGAAGCAGCAACGAAGAAG R:GAAGTGTGCGGTTCCTGTAGAC
CD79A	F:CAGTCCTGCGGCACCTACC R:CTCGGCTGTGATGATTCGGTTC
CD5	F:GCACGGTGGCAAGCATCATC R:TTCTGGCGGAATTTCTTCACTAGC
UTP20	F:AGAATAGAGCACAAGTCAGTAAAGAGC R:GAGGAATGGAAGGAGAAGCGTAATG
WDR43	F:ACCAAAGAAGTCTACAGGCATTTCAC R:CATCAAAGGGCTGGCTCTCATTAG
KRR1	F:GCCATTCCCACCACCACAAC R:TCTGCCGCTTCTTCTGATTTGC
HA067812	F:GCACCGTCAAGGCTGAGAAC R:CAGTCCTGCGGCACCTACC

### 2.9. Statistical analysis

All data were statistically analyzed using the R software version 4.1.3 (R Foundation for Statistical Computing, Vienna, Austria). Normality and log-normality tests were performed to assess the data distribution. Differences between 2 groups were evaluated using the Mann–Whitney *U* test and *t* test. A *P*-value of <.05 was considered statistically significant. All univariate and multivariate logistic regression analyses were implemented utilizing the “rms” package (The Comprehensive R Archive Network [CRAN]).^[49]^

## 3. Results

### 3.1. Cluster analysis

**Figure 1.**
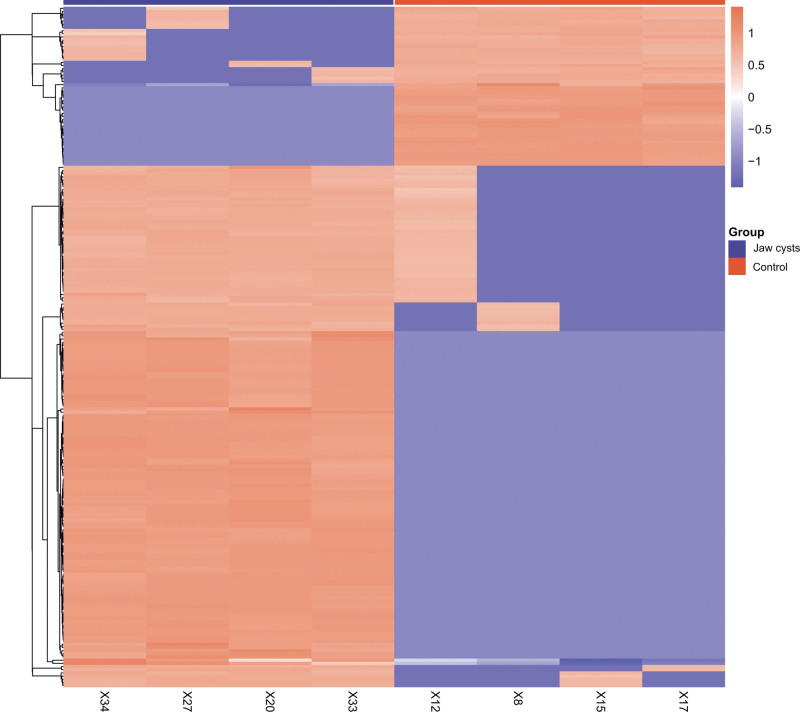
Examination of protein expression differences between the jaw cyst group and the control group.

Using the established screening criteria, we identified distinctively expressed proteins in samples taken from both healthy controls and jaw cyst groups. A total of 214 differentially expressed proteins were observed in the jaw cyst group compared with the normal control cohort. As depicted in Figure 1, the results of the cluster analysis revealed that purple illustrates the downregulation of protein expression levels in the jaw cyst group relative to healthy controls. In stark contrast, orange signifies an elevation in protein expression within the jaw cyst group compared to that in their healthy counterparts.

### 3.2. Detection results of differentially expressed proteins

A comprehensive proteomic analysis was performed on archival clinical samples (FFPE) collected from patients with jaw cysts and healthy controls. Using the predefined exploratory screening criteria, we identified 214 proteins showing differential expression between the jaw cyst and control groups. Among these, 50 proteins were upregulated and 164 were downregulated (Fig. 1 and Table S1). Given the exploratory nature of the discovery proteomic analysis and the absence of multiple testing correction at this stage, these proteins should be interpreted as candidate differentially expressed proteins requiring further validation. Additionally, Tables 2 and 3 highlight several proteins that exhibited marked differences in expression levels, underscoring their potential relevance in the context of jaw cyst pathology.

**Table 2:** Some low-expressed proteins in the tissues of patients with jaw cysts.

Gene	logFC	*P* value	FDR
FGR	−2.214693386	.035666232	1
HID1	−2.207184836	.036233925	1
S100P	−2.193994956	.037010081	1
NUFIP2	−2.180122611	.038564947	1
SDF2L1	−2.175287905	.038972077	1
DOK3	−2.162304696	.040157075	1
HELZ2	−2.156429498	.038464089	1
IRAG2	−2.153863332	.040904589	1
CRISP3	−2.153279944	.040407179	1
YTHDC1	−2.153104404	.040570769	1
SMPDL3B	−2.135566079	.042378278	1
RABAC1	−2.132678032	.042822224	1
THEMIS2	−2.13117893	.04304792	1
NBEAL2	−2.131020851	.042989597	1
CENPC	−2.130630038	.042999136	1
ITM2C	−2.129365019	.04267467	1
RETN	−2.129261898	.042678542	1
CASP10	−2.127160615	.043425742	1
MAN1A2	−2.125074805	.04359536	1
BTK	−2.119405038	.044134364	1
TIMMDC1	−2.116601309	.044449779	1
CD5	−2.113650528	.044423237	1
DPAGT1	−2.112099843	.044458396	1
TMEM245	−2.110603708	.044951075	1
ATP8A1	−2.110565168	.044998322	1

FDR = false discovery rate.

**Table 3:** Some of the highly expressed proteins in the tissues of patients with jaw cysts.

Gene	logFC	*P* value	FDR
HAPLN1	0.314862119	8.51E−05	0.516036412
TPST1	2.063634816	.049710771	1
TLCD3A	2.06576156	.049621188	1
GPRC5B	2.070971963	.049204088	1
P4HA3	2.074039293	.048794962	1
NFIC	2.075271642	.048768189	1
GULP1	2.076561925	.048460827	1
PTN	2.080169981	.047969356	1
WNT5A	2.081122628	.048017778	1
F13B	2.082060588	.047864979	1
LIMCH1	2.084036866	.047173677	1
KCTD15	2.087479196	.047486807	1
PLAT	2.088502478	.047115027	1
NCALD	2.091580511	.046855453	1
DCHS1	2.117287142	.044299973	1
ARHGAP6	2.125625991	.043482987	1
CLSTN1	2.140480839	.0417238	1
FAT4	2.148776837	.041332073	1
PIP	2.149944792	.040984911	1
SGCG	2.159309606	.040338284	1
PDGFRL	2.170039486	.03943595	1
EFS	2.179520967	.038607814	1
ANGPTL2	2.230464154	.034323234	1

FDR = false discovery rate.

### 3.3. GO and KEGG enrichment analysis of differentially expressed proteins

**Figure 2.**
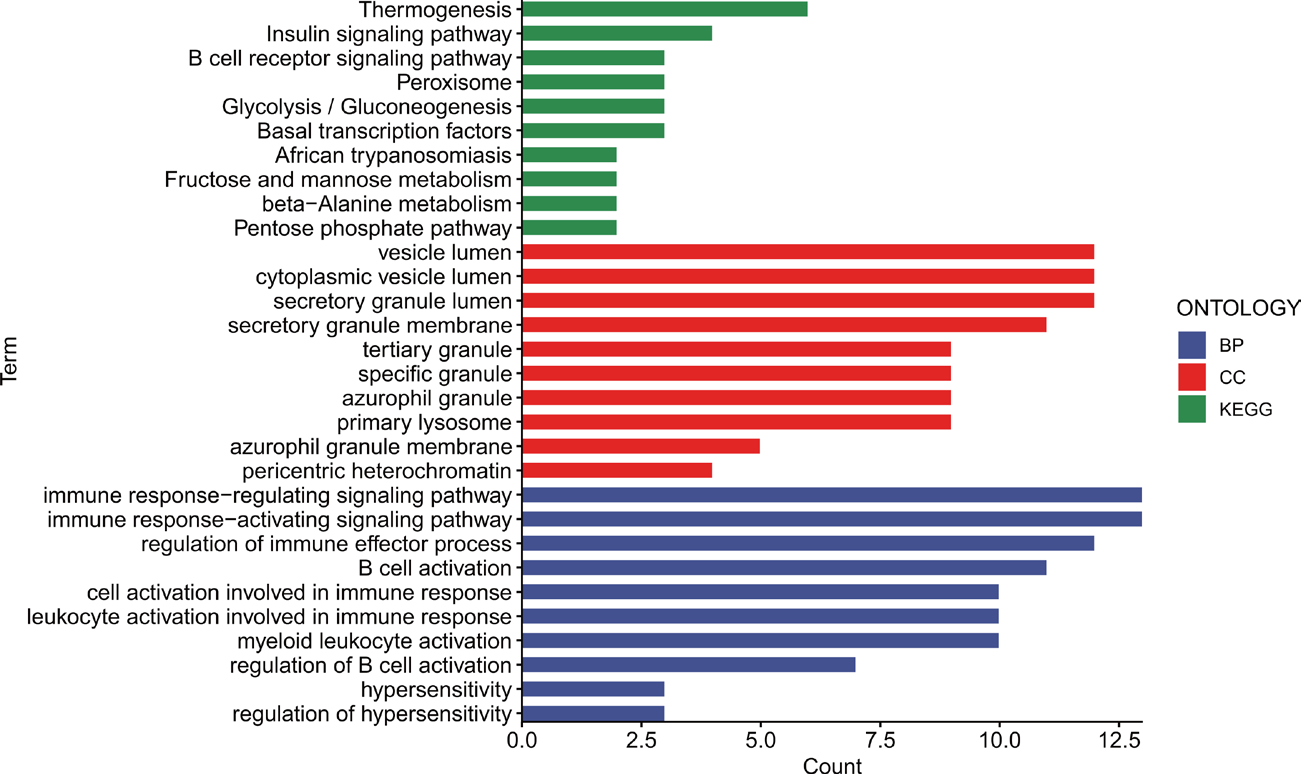
Analysis of functional enrichment lowly expressed proteins using GO and KEGG in both the jaw cyst and control groups. BP = biological process, CC = cellular component, GO = Gene Ontology, KEGG = Kyoto Encyclopedia of Genes and Genomes.

Gene function annotation and enrichment analysis comparing the jaw cyst group with the healthy control group yielded several noteworthy insights. In the category of low-expression proteins, significant enrichment was detected in cellular compartments, such as the vesicle lumen, cytoplasmic vesicle lumen, and secretory granule lumen. These proteins are primarily implicated in critical biological processes, including regulation of immune responses, immune response activation, regulation of immune effector processes, and B cell activation. Furthermore, pathway enrichment analysis revealed that these differentially expressed proteins were predominantly associated with thermogenesis, insulin signaling pathway, and B cell receptor signaling pathway. Collectively, these pathways may play pivotal roles in the pathogenesis of jaw cysts (Fig. 2).

**Figure 3.**
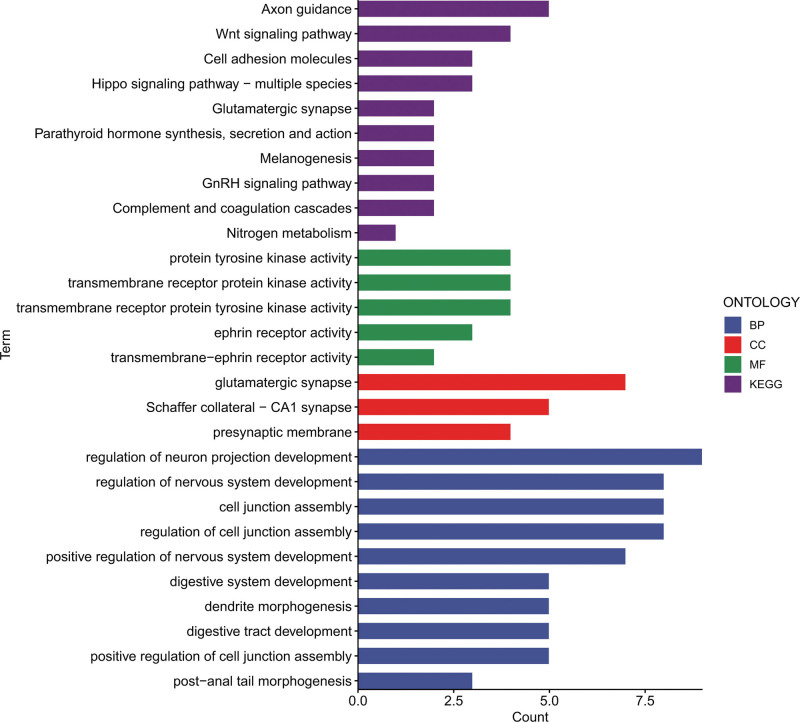
Analysis of functional enrichment for highly expressed proteins using GO and KEGG in both the jaw cyst and control groups. GO = Gene Ontology, KEGG = Kyoto Encyclopedia of Genes and Genomes, MF = molecular function.

In contrast, the analysis of high-expression proteins revealed significant enrichment in key areas such as the glutamatergic synapse, Schaffer collateral-CA1 synapse, and the presynaptic membrane. These proteins are characterized by important molecular functions, including protein tyrosine kinase, transmembrane receptor protein kinase, and transmembrane receptor protein tyrosine kinase activities. Additionally, they played vital roles in various biological processes, such as regulation of nervous system development, cell junction assembly, and regulation of cell junction assembly. Pathway enrichment analysis highlighted that these proteins with elevated expression were significantly associated with critical pathways, including axon guidance, Wnt signaling, and cell adhesion molecule pathways. These pathways may also contribute to the mechanisms underlying the pathogenesis of jaw cysts (Fig. 3).

It is important to note that these findings provide valuable insights into the potential mechanisms underlying jaw cyst formation. However, further research is required to validate and expand upon these findings.

### 3.4. Network analysis of differentially expressed proteins

**Figure 4.**
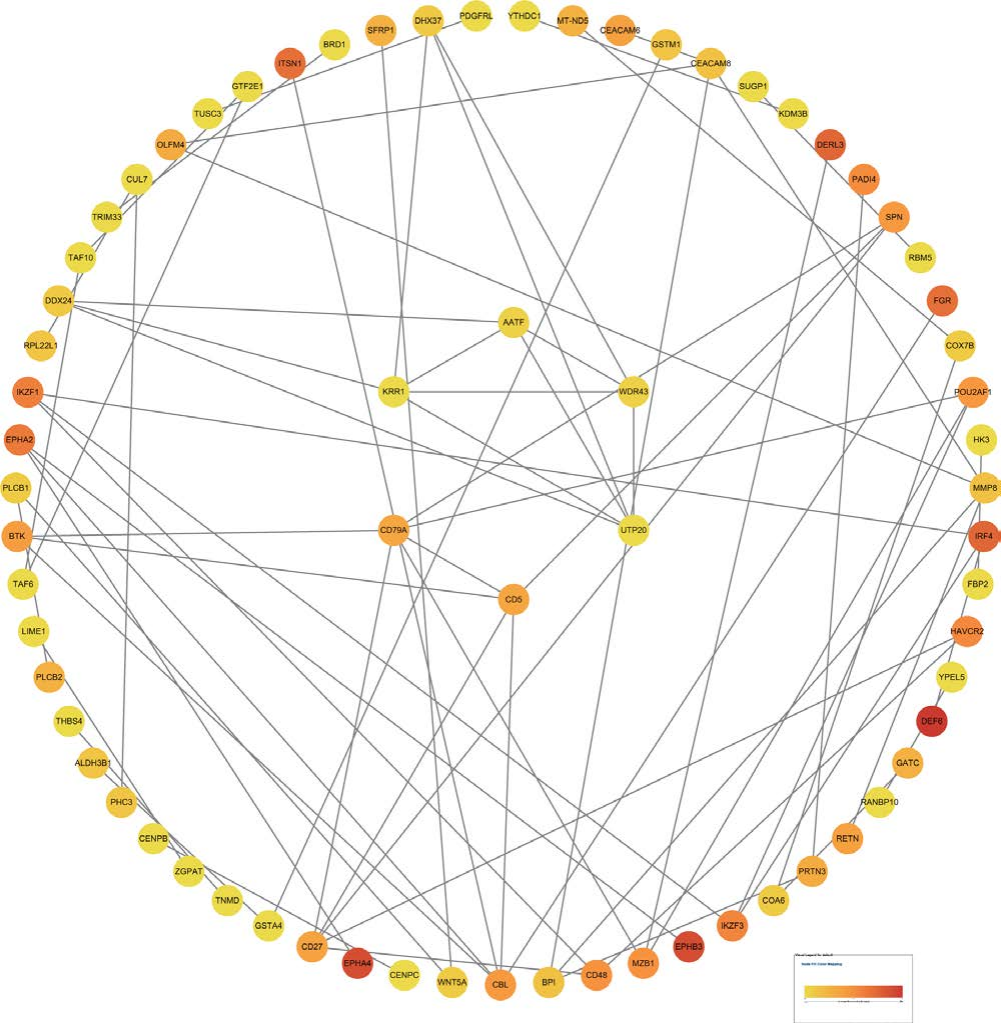
Analysis of protein interaction networks showing differentially expressed proteins between the jaw cyst group and the control group.

**Figure 5.**
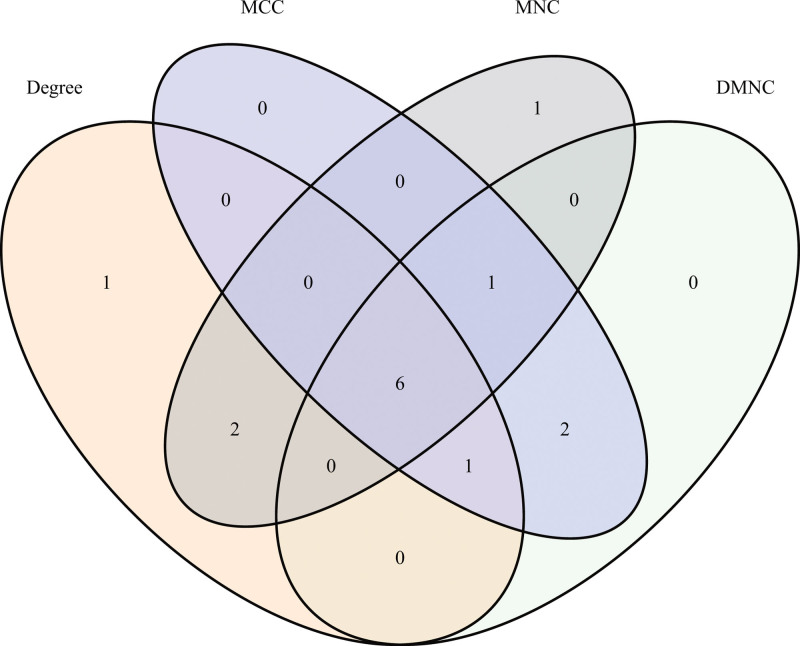
From the overlap of the 4 algorithms, the 6 main proteins identified are CD79A, CD5, KRR1, UTP20, AATF, and WDR43.

Using the STRING online tool, we analyzed the interaction relationships among the differentially expressed proteins.^[50-55]^ The results (Fig. 4) revealed that the protein interaction network comprised 214 nodes and 71 edges, yielding an average node degree of 0.664. To identify the core proteins, we intersected the proteins identified by the maximal clique centrality, density of maximum neighborhood component, maximum neighborhood component, and degree algorithms. This process led to the identification of 6 key core proteins: CD79A, CD5, KRR1, UTP20, AATF, and WDR43 (Fig. 5).

### 3.5. The significance of core proteins in the diagnosis of jaw cysts

**Figure 6.**
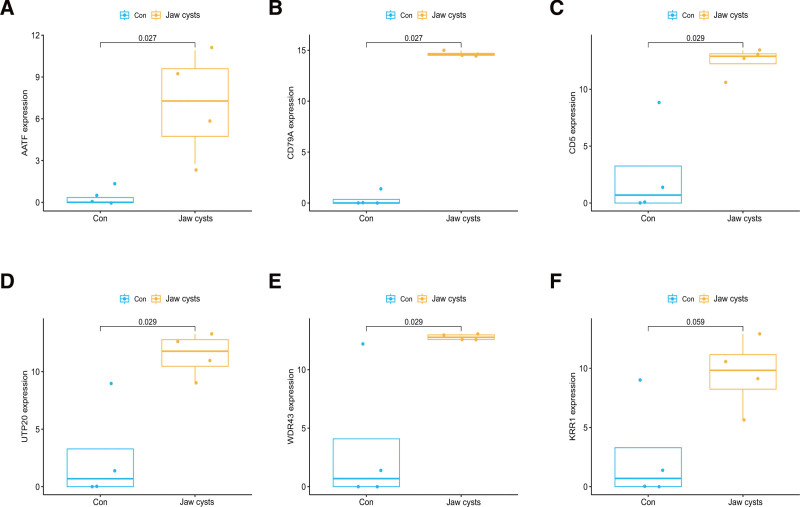
The expression of the core protein AATF varied between the blank group and the jaw cysts group in the proteomics data (A). The expression of the core protein. CD79A varied between the blank group and the jaw cysts group in the proteomics data (B). The expression of the core protein CD5 varied between the blank group and the jaw cysts group in the proteomics data (C). The expression of the core protein UTP20 varied between the blank group and the jaw cysts group in the proteomics data (D). The expression of the core protein WDR43 varied between the blank group and the jaw cysts group in the proteomics data (E). The expression of the core protein KRR1 varied between the blank group and the jaw cysts group in the proteomics data (F).

To evaluate the expression disparities of the 6 core proteins between non-jaw cyst controls and patients with jaw cysts, we constructed box plots to visualize the expression levels of these proteins within the proteomic dataset. As depicted in Figure 6, 5 genes – AATF, CD79A, CD5, UTP20, and WDR43 – showed significant differences in expression, all exhibiting notable upregulation in patients with jaw cysts.

### 3.6. Validation of quantitative reverse transcription polymerase chain reaction

**Figure 7.**
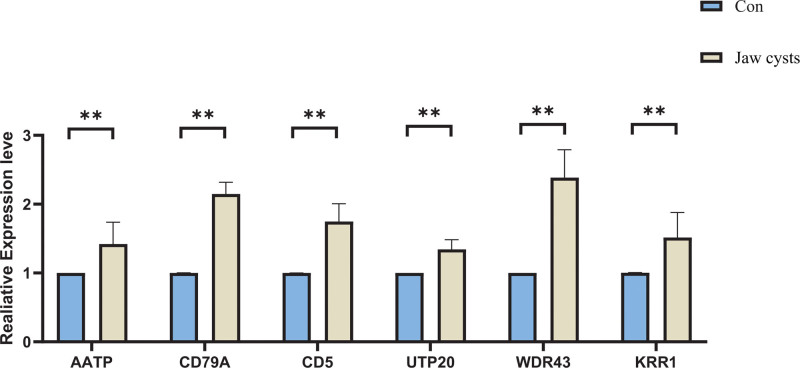
The expression of core protein in both the control and disease groups was confirmed using quantitative reverse transcription polymerase chain reaction (** represents *P* < .01).

The study cohort for the RT-qPCR analysis comprised 30 healthy controls and 30 patients with jaw cysts. Demographic and clinical characteristics, including age and sex, are summarized in Table 4. RT-qPCR analysis revealed significant upregulation of AATF, CD79A, CD5, UTP20, WDR43, and KRR1 in the patient group compared to the controls (Fig. 7). Detailed information on these 6 genes is presented in Table 5. Univariate and multivariate logistic regression analyses were performed to assess the diagnostic value of UTP20, AATF, and CD5 in relation to the clinical features of jaw cysts (Table 6). The results identified UTP20, AATF, and CD5 as independent diagnostic factors (*P* < .05).

**Table 4:** Clinical sample information of quantitative reverse transcription polymerase chain reaction is completed.

Variables	Total (n = 60)	Control (n = 30)	Jaw cysts (n = 30)	*P*
Sex, n (%)				.301
Female	29 (48)	12 (40)	17 (57)	
Male	31 (52)	18 (60)	13 (43)	
Age, median (Q1, Q3)	28 (23.75, 40)	26 (24, 36.5)	35.5 (21, 41.5)	.45

**Table 5:** The details of the 6 genes.

Gene	logFC	*P* value	Lower 95% CI	Upper 95% CI	Effect_size	FDR
AATF	−36.784	4.67E−05	−12.874	−10.789	−25.537	0.0022
CD79A	−37.092	2.35E−06	−15.127	−14.175	−69.259	0.0003
CD5	−2.114	.044	−19.422	−0.463	−2.347	0.1703
UTP20	−2.074	.049	−19.300	−0.117	−2.265	0.1770
WDR43	−2.073	.049	−19.595	−0.084	−2.267	0.1778
KRR1	−2.072	.049	−18.640	−0.100	−2.258	0.1774

CI = confidence interval, FDR = false discovery rate.

**Table 6:** Univariate and multivariate logistic regression.

	Univariate analysis OR (95% CI)	*P* value	Multivariate analysis OR (95% CI)	*P* value
Sex	0.510 (0.183–1.424)	.199	NA	
Age	1.023 (0.986–1.062)	.223	NA	
KRR1	1.110 (0.976–1.262)	.111	NA	
UTP20	1.594 (1.309–1.940)	<.001	1.678 (1.263–2.229)	<.001
AATF	1.261 (1.049–1.516)	.013	1.495 (1.050–2.127)	.026
CD79A	1.229 (1.048–1.442)	.011	NA	
WDR43	1.276 (1.011–1.612)	.040	NA	
CD5	2.203 (1.532–3.168)	<.001	1.798 (1.018–3.177)	.043

CI = confidence interval, NA = not applicable, OR = odds ratio.

## 4. Discussion

Mass spectrometry-based proteomics techniques have emerged as valuable tools for identifying potential biomarkers of benign odontogenic lesions and enhancing prediction and diagnostic capabilities.^[56]^ In this study, label-free quantitative proteomic techniques were employed to analyze protein profiles from FFPE tissue specimens of patients with jaw cysts and healthy volunteers. Label-free quantitative identification revealed 214 differential proteins in patients with jaw cysts. Among these, 50 proteins were upregulated and 164 proteins were downregulated. These differentially expressed proteins were enriched in several cellular compartments, such as the vesicle lumen, cytoplasmic vesicle lumen, secretory granule lumen, glutamatergic synapse, Schaffercollateral–CA1 synapse, and presynaptic membrane. The molecular functions of these proteins included protein tyrosine kinase activity, transmembrane receptor protein kinase activity, and transmembrane receptor protein tyrosine kinase activity. They are also involved in various biological processes, such as immune response regulation and activation, regulation of immune effector processes, B cell activation, regulation of neuron projection development, regulation of nervous system development, cell junction assembly, and regulation of cell junction assembly. These findings provide insights into the potential molecular mechanisms underlying jaw cysts and highlight the involvement of specific biological pathways. However, further research is necessary to validate these findings and to understand the precise roles of these differentially expressed proteins in the pathogenesis of jaw cysts.

The presence of the inflammasome Nlrp3 and its immune response to bacterial infection have been shown to contribute to the development of apical periodontitis, an inflammatory disease.^[57]^ Additionally, the formation of tertiary lymphoid structures within periapical granulomas and cysts is linked to ongoing immune responses and bone loss in apical lesions.^[58]^ Odontogenic keratocystic tumors exhibit significant components of the cell-mediated immune response that differentiate them from other conditions involving Langerhans cells.^[59]^ Furthermore, B lymphocytes are found in the monocytes of dental periapical inflammatory lesions, highlighting the role of immune responses in these conditions. Jaw cysts are also commonly observed in Gorlin–Goltz syndrome, a disorder that presents with symptoms affecting the skin, central nervous system, and skeletal system.^[60]^ This observation underscores the association between jaw cysts and their systemic manifestations. Concerning the Wnt signaling pathway and β-catenin, it has been reported that they play critical roles in the pathogenesis of various odontogenic and neoplastic lesions by influencing cell-to-cell adhesion.^[61]^ KEGG analysis results corroborated these findings, indicating the involvement of the Wnt signaling pathway and β-catenin in jaw cyst pathogenesis.

The results of the PPI analysis suggest that CD79A, CD5, KRR1, UTP20, AATF, and WDR43 may be proteins potentially associated with the pathogenesis. Studies have indicated that imaging of a patient with multiple osteolytic lesions in the mandible revealed significant root resorption in the left second molar. Biopsy revealed atypical plasmacytoid cells that tested positive for anti-κ, CD138, MUM1, and CD79a antibodies.^[62]^ Immunohistochemical analysis of a patient with a submandibular tumor revealed CD5 positivity.^[63]^ Although the connection between other proteins and jaw cysts has not yet been established, it is premature to rule out their involvement. Further investigations are required to elucidate their potential roles. Genes indirectly regulate protein expression by modulating the synthesis of messenger RNA (mRNA).^[64]^ Therefore, we used RT-qPCR to validate our findings. The RT-qPCR results indicated that the expression of core proteins was upregulated in the disease group compared to the blank control group.

The research findings presented indicate that proteomics analysis techniques have identified 214 proteins associated with jaw cysts. The GO enrichment analysis showed that these differential genes were mainly involved in the biological process of immune response to jaw cysts. Additionally, the KEGG enrichment pathway analysis revealed that the Wnt signaling pathway, axon guidance, and thermogenesis pathways play important roles in the pathogenesis of jaw cysts.

This study has several limitations. First, although GO and KEGG enrichment analyses highlighted multiple biological processes and pathways potentially involved in jaw cyst pathogenesis, additional relevant pathways or regulatory networks may not have been captured and therefore warrant further investigation. Second, the sample size of the discovery-phase proteomic analysis, particularly the FFPE-based cohort, was relatively small, which limits statistical power and increases the likelihood of false-positive findings. Third, the initial screening of differentially expressed proteins was performed in an exploratory manner and did not include multiple testing correction; therefore, the 214 identified proteins should be interpreted as candidate proteins rather than definitive disease-associated markers. Although selected targets were further validated by RT-qPCR, additional confirmation in larger and independent cohorts will be necessary to strengthen the robustness and reproducibility of these findings. Finally, given the exploratory design of this study, the identified biomarkers and enriched pathways should be regarded as hypothesis-generating and require further functional and clinical validation before being applied as reliable diagnostic indicators for jaw cysts.

## 5. Conclusion

In this study, proteomic technology was employed to identify and analyze the proteome of jaw cyst tissues, resulting in the identification of 6584 proteins. Among these, 214 proteins exhibited significant differential expression, with 50 highly expressed and 164 showing low expression. Functional enrichment analysis and protein–protein interaction network analysis of these differentially expressed proteins led to the identification of 6 core proteins potentially associated with jaw cyst development: CD79A, CD5, KRR1, UTP20, AATF, and WDR43. These findings provide valuable data to support the development of molecular markers for jaw cysts.

## Acknowledgments

The authors thank the Medical Science Research Project of Hebei and Affiliated Hospital of Chengde Medical University. We appreciate the patient’s support.

## Author contributions

**Data curation:** Yanbo Zhang, Jiashun Du.

**Formal analysis:** Lingli Lan.

**Investigation:** Feng Huo.

**Software:** Shangzhi Han.

**Writing – original draft:** Xuefeng Wang.

**Writing – review & editing:** Xibo Chen.


