# Supplementary material for: Unveiling the proteomic landscape: Exploring differentially expressed proteins in patients with jaw cysts
Source: Medicine (Baltimore). 2026 May 22;105(21):e48872. doi: 10.1097/MD.0000000000048872 (PMC13201010; doi:10.1097/MD.0000000000048872)
Supplement: Supplementary file 1 [file medi-105-e48872-s001.docx]

**Table S1.** All the differentially expressed proteins.

|  | logFC | p.value | CI_lower | CI_upper | effect_size | FDR |
| --- | --- | --- | --- | --- | --- | --- |
| A1BG | 0.0554369052171172 | 0.0554369052171172 | -0.133330516 | 1.33144119479798 | 1.45612255273851 | 0.244457704982357 |
| A2M | 0.0604483048903418 | 0.0604483048903418 | -0.401881327 | 1.808627743 | 1.16073915664014 | 0.363745897784638 |
| A2ML1 | -0.040447639 | 0.959552361 | -13.67324789 | 13.0248781000518 | -0.042027365 | 0.994613821823136 |
| AAAS | 0.00659187547191537 | 0.00659187547191537 | -0.186257829 | 0.315986138503144 | 0.468150166897132 | 0.652016273769328 |
| AACS | -1.023178214 | -0.023178214 | -14.89324841 | 8.52392457548704 | -0.473495535 | 0.646546567721165 |
| AAK1 | -0.021858245 | 0.978141755 | -0.47613222 | 0.0691892058768113 | -1.547737785 | 0.269580762375348 |
| AAMDC | 0.0623842395689106 | 0.0623842395689106 | -0.010331953 | 1.19839889577844 | 2.01839085050151 | 0.1803694486231 |
| AAMP | -1.604137697 | -0.604137697 | -16.9729491 | 4.43647714490389 | -1.013194061 | 0.381625389845557 |
| AAR2 | 36.3473762884668 | 36.3473762884668 | -0.534192566 | 18.0197918784964 | 2.12074973342397 | 0.1803694486231 |
| AARS1 | -0.035472897 | 0.964527103 | -0.9369483 | 0.20504732920071 | -1.376977091 | 0.334509726783066 |
| AARS2 | -0.019376234 | 0.980623766 | -0.586831607 | 0.235362289615903 | -0.798046561 | 0.517642586759047 |
| AARSD1 | 0.030693892482301 | 0.030693892482301 | -0.055975637 | 0.636897588 | 1.50640492463776 | 0.230465982266928 |
| AASDHPPT | 0.566695569020773 | 0.566695569020773 | -9.068599168 | 15.3499417246844 | 0.447685366256201 | 0.657423743030449 |
| AASS | 0.0435628055867874 | 0.0435628055867874 | -0.159554754 | 0.916072044646794 | 1.21862869775798 | 0.329385561368832 |
| AATF | -36.78382388 | -35.78382388 | -12.87392483 | -10.78877735 | -25.53722172 | 0.00221572006546813 |
| ABAT | 0.0863757698896053 | 0.0863757698896053 | 0.377838107629355 | 1.15543201750296 | 4.05178632683955 | 0.0558555221012115 |
| ABCA1 | 0.0266623871275956 | 0.0266623871275956 | -11.26410464 | 11.6297989654963 | 0.0276400153034045 | 0.998435102357591 |
| ABCA12 | -34.98705113 | -33.98705113 | -14.24236865 | 7.43182400094131 | -0.707106781 | 0.539497285386707 |
| ABCA8 | -34.96622204 | -33.96622204 | -14.03821996 | 7.32529697861281 | -0.707106781 | 0.539497285386707 |
| ABCB10 | -0.997418895 | 0.002581105 | -16.42088646 | 4.87222229672688 | -1.219985915 | 0.366275438800159 |
| ABCB6 | 0.0660848731914365 | 0.0660848731914365 | -0.090797763 | 1.46781638170327 | 1.83066535879001 | 0.199327137008101 |
| ABCB7 | -0.472124351 | 0.527875649 | -13.80841929 | 6.43151461897246 | -0.819088632 | 0.531441347278013 |
| ABCB8 | -1.068138173 | -0.068138173 | -18.2698321 | 4.61060023124782 | -1.343045596 | 0.354826113544616 |
| ABCC1 | 0.00863618634381643 | 0.00863618634381643 | -0.290595255 | 0.443729567074308 | 0.379789825349708 | 0.708693825202794 |
| ABCC4 | 0.0550707810218742 | 0.0550707810218742 | 0.166429913832105 | 0.904416608106284 | 2.53104955956803 | 0.0809531445735674 |
| ABCD1 | 0.306040630830122 | 0.306040630830122 | -8.035233012 | 12.6609498004598 | 0.499032507347596 | 0.647660636368325 |
| ABCD3 | -0.019575019 | 0.980424981 | -0.552575508 | 0.196534602680326 | -0.935179741 | 0.450754258845013 |
| ABCE1 | -0.019902974 | 0.980097026 | -0.30263739 | -0.089887451 | -3.399755339 | 0.0525595373037303 |
| ABCF1 | -0.027473098 | 0.972526902 | -0.451822434 | -0.09828501 | -2.75869273 | 0.0704375074851147 |
| ABCF2 | -0.028517101 | 0.971482899 | -0.739817584 | 0.212227105524967 | -0.96054171 | 0.405497473271405 |
| ABCF3 | -0.053938561 | 0.946061439 | -0.828005552 | -0.202764563 | -3.284503578 | 0.0743338569306362 |
| ABHD10 | -0.009996763 | 0.990003237 | -0.480795494 | 0.278753875685235 | -0.462379426 | 0.652187811666889 |
| ABHD11 | -0.051246716 | 0.948753284 | -0.754112404 | -0.262319844 | -3.768312427 | 0.0400762818574951 |
| ABHD12 | -0.022531488 | 0.977468512 | -0.689413969 | 0.255558242071497 | -0.822964043 | 0.495865092003581 |
| ABHD14B | -0.001839761 | 0.998160239 | -0.474347114 | 0.434169233111087 | -0.077039845 | 0.97318663217728 |
| ABHD16A | -0.031776167 | 0.968223833 | -0.630032908 | 0.0310807285715496 | -1.625047809 | 0.196629205056468 |
| ABHD5 | -34.82298484 | -33.82298484 | -12.71139894 | 6.6329472324506 | -0.707106781 | 0.539497285386707 |
| ABI1 | 0.0111340902091014 | 0.0111340902091014 | -0.122150474 | 0.343374802677269 | 1.0208056028021 | 0.424267295692596 |
| ABI3 | -36.9458454 | -35.9458454 | -13.59246657 | -12.88263752 | -83.93246816 | 0.000220658446544722 |
| ABI3BP | 0.156538131296661 | 0.156538131296661 | 1.12554560603254 | 2.05824222023945 | 5.91029059989255 | 0.00623830265285425 |
| ABLIM1 | 0.0416474013239561 | 0.0416474013239561 | -0.234734944 | 1.01035338796921 | 1.09582595166801 | 0.366275438800159 |
| ABLIM2 | 0 | 1 | NA | NA | NA | NA |
| ABR | 0.00114051443476501 | 0.00114051443476501 | -0.771751626 | 0.793260865294315 | 0.0294604734184138 | 0.998435102357591 |
| ABRACL | -0.060222438 | 0.939777562 | -1.823818977 | 0.787501732928033 | -0.68852956 | 0.539497285386707 |
| ABRAXAS2 | 0.0236771904674528 | 0.0236771904674528 | -0.073458341 | 0.4947911509962 | 1.28671983253369 | 0.299804204175093 |
| ABT1 | -0.52425241 | 0.47574759 | -13.93027017 | 8.60948655768773 | -0.411714666 | 0.680219321264066 |
| ACAA1 | -0.040955028 | 0.959044972 | -0.857987256 | 0.0717905775882551 | -1.464790228 | 0.229893724436788 |
| ACAA2 | -0.022422316 | 0.977577684 | -0.520130059 | 0.0249590143847265 | -1.597944857 | 0.196580602683032 |
| ACACA | 0.636118769337056 | 0.636118769337056 | -8.589046053 | 15.6437944484689 | 0.505135732473303 | 0.628146333032989 |
| ACAD10 | -1.667476252 | -0.667476252 | -15.63705705 | 3.79558484741485 | -1.055246336 | 0.36731973075278 |
| ACAD8 | -0.028742922 | 0.971257078 | -0.444862373 | -0.105674764 | -2.8432049 | 0.0618056075000179 |
| ACAD9 | -0.025535679 | 0.974464321 | -0.463778312 | -0.042474795 | -2.323862205 | 0.129339758242125 |
| ACADM | -0.08659052 | 0.91340948 | -1.141338671 | -0.748921517 | -8.658908874 | 0.00231224898897599 |
| ACADS | -0.063109113 | 0.936890887 | -0.957498714 | -0.248533491 | -2.992749473 | 0.0559026095958365 |
| ACADSB | -0.048444494 | 0.951555506 | -0.626331482 | -0.306239238 | -5.428822095 | 0.0187515300021514 |
| ACADVL | -0.035227759 | 0.964772241 | -0.670491444 | -0.098507749 | -2.365251223 | 0.0962572718737131 |
| ACAN | 0.703679047973303 | 0.703679047973303 | -4.365189452 | 16.9803568400944 | 1.32543683998575 | 0.359018356387444 |
| ACAP1 | -0.494758732 | 0.505241268 | -13.71230612 | 6.08026038397788 | -0.867464951 | 0.509932647461076 |
| ACAP2 | -0.006092906 | 0.993907094 | -0.393973995 | 0.283867172937423 | -0.281843357 | 0.790369456962067 |
| ACAT1 | -0.049218771 | 0.950781229 | -0.734987449 | -0.325415347 | -4.579783001 | 0.0196658587740803 |
| ACAT2 | 0.0316223759287459 | 0.0316223759287459 | 0.0711796525226074 | 0.575860166064873 | 2.22578786248079 | 0.106312511057113 |
| ACBD3 | -0.047565546 | 0.952434454 | -0.675648545 | -0.237862085 | -3.791566806 | 0.0390940564917974 |
| ACBD5 | -1.595166591 | -0.595166591 | -16.78429218 | 4.44838260348429 | -1.005267549 | 0.384405979663039 |
| ACE | 0.0601729366302563 | 0.0601729366302563 | 0.29861109820169 | 0.862884182070938 | 3.58156056416825 | 0.0350690872816596 |
| ACHE | 0.0624409884950346 | 0.0624409884950346 | -11.91784456 | 12.4451370499357 | 0.03746464179877 | 0.996395923375039 |
| ACIN1 | -0.051381644 | 0.948618356 | -0.871989276 | -0.152441163 | -2.600813943 | 0.0876930912577496 |
| ACLY | 0.00327653973655002 | 0.00327653973655002 | -0.327283132 | 0.394901937256511 | 0.164089590697791 | 0.899377223159709 |
| ACO1 | 0.0170385925675175 | 0.0170385925675175 | -0.003727623 | 0.347646671300189 | 1.76863944203121 | 0.1803694486231 |
| ACO2 | -0.08061628 | 0.91938372 | -1.123296687 | -0.612321185 | -5.974869189 | 0.00742007994773924 |
| ACOT1 | 1.96602225149579 | 1.96602225149579 | -0.884684332 | 20.5062485138928 | 2.0484649944481 | 0.184643860681916 |
| ACOT11 | 0.191188895310611 | 0.191188895310611 | 0.926589799321126 | 2.73249567916023 | 3.61587235052955 | 0.0395805649269449 |
| ACOT13 | -0.057612363 | 0.942387637 | -0.956327226 | -0.250523172 | -3.718706972 | 0.0773628411633584 |
| ACOT7 | 0.00876698752536385 | 0.00876698752536385 | -0.285632867 | 0.453632023618246 | 0.397097628304116 | 0.691260480224731 |
| ACOT8 | -34.78477222 | -33.78477222 | -12.37913231 | 6.45956686830451 | -0.707106781 | 0.539497285386707 |
| ACOT9 | 0.0112728925282866 | 0.0112728925282866 | -0.249198387 | 0.486019304620469 | 0.586482244293764 | 0.580844906261604 |
| ACOX1 | -0.006788631 | 0.993211369 | -0.226497025 | 0.0912601417898183 | -0.742790334 | 0.534972335802981 |
| ACOX2 | 34.8289829141699 | 34.8289829141699 | -6.660581445 | 12.7643572206883 | 0.707106781186548 | 0.539497285386707 |
| ACOX3 | -0.033976982 | 0.966023018 | -0.859641496 | 0.269847102647588 | -0.947403863 | 0.42457532978048 |
| ACP1 | -0.039576835 | 0.960423165 | -0.996862964 | 0.107681982939031 | -1.581081192 | 0.241079441821201 |
| ACP2 | -0.06197099 | 0.93802901 | -1.00293996 | -0.253229734 | -2.927041259 | 0.0565936076207959 |
| ACP3 | 0 | 1 | NA | NA | NA | NA |
| ACP5 | 0.388689837644631 | 0.388689837644631 | -7.955150768 | 14.5187268258241 | 0.653889760648617 | 0.562639809199418 |
| ACP7 | 0 | 1 | NA | NA | NA | NA |
| ACSBG1 | 0 | 1 | NA | NA | NA | NA |
| ACSF2 | 0.0101730944835865 | 0.0101730944835865 | -0.283003724 | 0.484665935732337 | 0.473987284599976 | 0.648356244122926 |
| ACSF3 | -0.046021547 | 0.953978453 | -0.908320567 | 0.0225107529786026 | -1.663036665 | 0.180515175219602 |
| ACSL1 | -0.058398829 | 0.941601171 | -1.077922759 | -0.034621117 | -1.894587948 | 0.161848075171975 |
| ACSL3 | 0.00125902954689591 | 0.00125902954689591 | -0.392853903 | 0.416854986224779 | 0.0593963424216611 | 0.987185871906287 |
| ACSL4 | -0.476587132 | 0.523412868 | -14.0288459 | 6.46550273641897 | -0.830251718 | 0.526930183942254 |
| ACSL5 | -36.91860778 | -35.91860778 | -13.24179231 | -12.73815912 | -116.0833896 | 0.000170529874772727 |
| ACSS1 | -0.143923901 | 0.856076099 | -1.70369027 | -1.011918231 | -6.984162125 | 0.00493742595937386 |
| ACSS2 | 0.000562662728407642 | 0.000562662728407642 | -0.303199306 | 0.313595871575103 | 0.0313632973927317 | 0.998435102357591 |
| ACSS3 | 0.480539009689951 | 0.480539009689951 | -6.634884442 | 14.5123844167472 | 0.838096219076414 | 0.523510473710632 |
| ACTA1 | 0.0674549434482947 | 0.0674549434482947 | -0.456201709 | 1.88445816076882 | 1.06248376799647 | 0.366735091564231 |
| ACTBL2 | 0.0765403643732895 | 0.0765403643732895 | -1.053186365 | 2.97663090196772 | 1.06806819351099 | 0.410175342882834 |
| ACTL6A | -0.018662477 | 0.981337523 | -0.545782028 | 0.178832548583537 | -0.936377613 | 0.434740592587164 |
| ACTN1 | 0.0670251451628124 | 0.0670251451628124 | 0.279276573828492 | 1.27935152208579 | 2.90036767804089 | 0.0757421504201993 |
| ACTN2 | 0.39837311477985 | 0.39837311477985 | -7.695726341 | 14.2768275753434 | 0.650039686449822 | 0.562031639448264 |
| ACTN3 | 0 | 1 | NA | NA | NA | NA |
| ACTN4 | 0.0307801506416771 | 0.0307801506416771 | -0.053450714 | 0.730334517208415 | 1.72439699034322 | 0.209473314816446 |
| ACTR10 | 0.0539174395516803 | 0.0539174395516803 | 0.177297405213313 | 0.831578495882539 | 2.81757466969234 | 0.0753698265741615 |
| ACTR1A | 0.0349500038298419 | 0.0349500038298419 | 0.0660746696858868 | 0.614166513813919 | 2.57925036017522 | 0.126513452167711 |
| ACTR1B | 0.0409624943129168 | 0.0409624943129168 | -0.207024794 | 0.974584763322959 | 1.20026249996728 | 0.354826113544616 |
| ACTR2 | 0.00366365653124013 | 0.00366365653124013 | -0.398422616 | 0.480985133174782 | 0.16950347638076 | 0.895313790427314 |
| ACTR3 | -0.007865256 | 0.992134744 | -0.361470955 | 0.191126177277683 | -0.632854231 | 0.56596689778833 |
| ACY1 | -0.003065583 | 0.996934417 | -0.445540029 | 0.391956699917046 | -0.115692547 | 0.941738705317131 |
| ACYP1 | -1.978238993 | -0.978238993 | -19.15157965 | 0.75327429120994 | -2.078160491 | 0.182584688249216 |
| ACYP2 | -0.495414745 | 0.504585255 | -14.27850118 | 6.32127100353601 | -0.868886274 | 0.509607148662422 |
| ADA | -0.034837096 | 0.965162904 | -0.746673152 | 0.119182105391288 | -1.296961471 | 0.306724636864787 |
| ADA2 | 0.016007829261916 | 0.016007829261916 | -0.334275619 | 0.65223267024937 | 0.574772695823291 | 0.586418380770364 |
| ADAM10 | -6.99E-05 | 1.00E+00 | -0.467527703 | 0.4661858991197 | -0.002748063 | 0.999376962148702 |
| ADAM15 | 0.00295770693208058 | 0.00295770693208058 | -10.47604716 | 10.5133465731612 | 0.00307472266811069 | 0.999376962148702 |
| ADAM17 | -0.422524484 | 0.577475516 | -13.91971153 | 7.15563414601921 | -0.721488406 | 0.539497285386707 |
| ADAM28 | -36.72115456 | -35.72115456 | -11.80191251 | -10.85490951 | -53.83859587 | 0.00040238102253647 |
| ADAM9 | 35.9819516874348 | 35.9819516874348 | -5.775978073 | 19.3490064197128 | 1.21567375479221 | 0.366735091564231 |
| ADAMTSL3 | 36.3792065111325 | 36.3792065111325 | -0.558708148 | 18.4343800146261 | 2.11793621677994 | 0.1803694486231 |
| ADAMTSL4 | 0.0549857476737607 | 0.0549857476737607 | -0.287429563 | 1.2464542425823 | 1.30332833469377 | 0.350426488005461 |
| ADAR | -0.023792279 | 0.976207721 | -0.402181733 | -0.066415525 | -2.784870943 | 0.100763636811655 |
| ADARB1 | -0.014029742 | 0.985970258 | -0.621300026 | 0.374029779229328 | -0.432300162 | 0.665818265109824 |
| ADD1 | 0.0327039707450331 | 0.0327039707450331 | -0.069757953 | 0.743942746830379 | 1.73682111214125 | 0.223010900988626 |
| ADD2 | 0.0270174621259965 | 0.0270174621259965 | -0.848508733 | 1.36321873434069 | 0.413374471744697 | 0.680219321264066 |
| ADD3 | 0.0586780779058656 | 0.0586780779058656 | 0.348146170031403 | 0.883102327799128 | 4.00023445094203 | 0.0256475213740026 |
| ADGB | -0.604390636 | 0.395609364 | -15.75926266 | 8.94812742679355 | -0.478924741 | 0.643273573113642 |
| ADGRA2 | 36.6562648951933 | 36.6562648951933 | -0.660052999 | 22.3203924355333 | 2.12105975013586 | 0.1803694486231 |
| ADGRE5 | -0.147482576 | 0.852517424 | -2.34577679 | -0.339667825 | -2.92746774 | 0.114364743075441 |
| ADGRG3 | -34.72374707 | -33.72374707 | -11.86642244 | 6.19202923716031 | -0.707106781 | 0.539497285386707 |
| ADGRL2 | 2.01939169714305 | 2.01939169714305 | -0.42691158 | 21.1792902342064 | 2.15815870489234 | 0.1803694486231 |
| ADH1B | 0 | 1 | NA | NA | NA | NA |
| ADH1C | 0 | 1 | NA | NA | NA | NA |
| ADH5 | 0.0504865961252088 | 0.0504865961252088 | 0.278185017776768 | 0.786024168726412 | 4.03192444252351 | 0.0443975166225279 |
| ADH7 | -34.93090137 | -33.93090137 | -13.69870338 | 7.14813350566194 | -0.707106781 | 0.539497285386707 |
| ADHFE1 | -0.918724935 | 0.081275065 | -18.14483232 | 6.24706780650382 | -1.08462216 | 0.402643473413631 |
| ADI1 | -2.016032146 | -1.016032146 | -20.05975752 | 0.424856865936201 | -2.149688577 | 0.1803694486231 |
| ADIPOQ | 1.1161112680214 | 1.1161112680214 | -4.000405174 | 18.004190717296 | 1.42822938240196 | 0.329699705770463 |
| ADIRF | 0.079255375737065 | 0.079255375737065 | 0.000495138251198358 | 1.46753665535561 | 1.78154204639795 | 0.179117559639718 |
| ADK | 0.0459683373122309 | 0.0459683373122309 | -0.047571586 | 1.01468171385509 | 1.80950823666836 | 0.189983954626358 |
| ADNP | -1.069259033 | -0.069259033 | -15.27881435 | 8.53369395870838 | -0.494403012 | 0.63426154106085 |
| ADO | 0.0021798608613462 | 0.0021798608613462 | -0.312581176 | 0.351295641344843 | 0.111446986701142 | 0.945587956124106 |
| ADPGK | -0.006581438 | 0.993418562 | -0.423641644 | 0.293241676066659 | -0.359086393 | 0.731672476698841 |
| ADPRH | -1.024135558 | -0.024135558 | -17.8515569 | 4.98948929361692 | -1.265150452 | 0.366275438800159 |
| ADPRHL1 | 0 | 1 | NA | NA | NA | NA |
| ADPRS | -0.001888721 | 0.998111279 | -0.252960869 | 0.217114497068625 | -0.151801963 | 0.912305317283097 |
| ADRM1 | -0.034798811 | 0.965201189 | -0.507304673 | -0.166491036 | -3.717637424 | 0.0478090728526005 |
| ADSL | 0.00124376752753262 | 0.00124376752753262 | -0.281897715 | 0.30613772149318 | 0.072434190263464 | 0.976555054584308 |
| ADSS1 | 36.5740106921077 | 36.5740106921077 | -0.950960974 | 21.410897202532 | 2.0589342307603 | 0.185146238018798 |
| ADSS2 | -0.011146664 | 0.988853336 | -0.238608516 | 0.0186208349199369 | -1.481823824 | 0.224772101663755 |
| AEBP1 | 0.147707097896924 | 0.147707097896924 | 0.753482632374338 | 2.41998312102966 | 3.65369231761609 | 0.0532609661311465 |
| AFAP1 | 36.8389250578402 | 36.8389250578402 | 11.7695610450183 | 12.8143766219411 | 52.9490160920714 | 0.000407188114622109 |
| AFAP1L2 | 0 | 1 | NA | NA | NA | NA |
| AFDN | 0.0242243975346392 | 0.0242243975346392 | -0.174543436 | 0.624801355458625 | 0.976072967399812 | 0.397816771093531 |
| AFG3L2 | -0.017004134 | 0.982995866 | -0.334469755 | 0.000972245696925215 | -1.726232416 | 0.1803694486231 |
| AFM | 0.0613507539779846 | 0.0613507539779846 | -0.411371684 | 1.5844506086508 | 1.04806593859614 | 0.374572457284349 |
| AGA | -0.127012032 | 0.872987968 | -1.644538562 | -0.809418293 | -5.152174914 | 0.0122496344082122 |
| AGAP2 | -34.62093686 | -33.62093686 | -11.05021803 | 5.7661248345925 | -0.707106781 | 0.539497285386707 |
| AGAP3 | -0.001367343 | 0.998632657 | -0.799655217 | 0.776625840949518 | -0.025281941 | 0.999039582512423 |
| AGBL1 | 0 | 1 | NA | NA | NA | NA |
| AGFG1 | -0.007455054 | 0.992544946 | -0.317459755 | 0.176379608472658 | -0.524869505 | 0.621259342074536 |
| AGK | -0.035424779 | 0.964575221 | -0.660614607 | 0.0181680969230238 | -1.6435836 | 0.181452258590238 |
| AGL | 0.0481416807441457 | 0.0481416807441457 | 0.0185952193865352 | 0.902123694781816 | 1.94981518379231 | 0.16964907443098 |
| AGO1 | 0.0191885796776611 | 0.0191885796776611 | 0.00846709879666877 | 0.346553705045163 | 1.93749499620416 | 0.167509566752535 |
| AGO2 | 0.0342685311966156 | 0.0342685311966156 | -0.07288726 | 0.72518525325213 | 1.58251758639784 | 0.235622397821313 |
| AGO3 | 0.0126552024560282 | 0.0126552024560282 | -0.694840626 | 0.928441836315863 | 0.263506318629935 | 0.809423868459677 |
| AGPAT1 | 0.0177304765577628 | 0.0177304765577628 | -0.416741066 | 0.749369572313496 | 0.522650174107342 | 0.621704967022904 |
| AGPAT2 | 0.00380667179225883 | 0.00380667179225883 | -0.382694844 | 0.452919167456846 | 0.146905640222414 | 0.913203838069743 |
| AGPAT3 | -0.424582291 | 0.575417709 | -13.75097754 | 7.04056459392375 | -0.725258429 | 0.539497285386707 |
| AGPAT5 | -36.89210609 | -35.89210609 | -13.10258992 | -12.40447766 | -82.22073424 | 0.000220658446544722 |
| AGPS | -0.014912281 | 0.985087719 | -0.569076759 | 0.280575668532324 | -0.605285095 | 0.567716600263508 |
| AGR2 | 0 | 1 | NA | NA | NA | NA |
| AGRN | 0.0941111632556815 | 0.0941111632556815 | 0.567158379241853 | 1.24005523994188 | 4.67096276370182 | 0.0156637250586441 |
| AGT | 0.0574698533462751 | 0.0574698533462751 | 0.167808810253794 | 0.991260966257061 | 2.44259273691331 | 0.0849373816717707 |
| AGTRAP | -0.320341514 | 0.679658486 | -13.82477599 | 8.57983926317822 | -0.525220409 | 0.634031727574291 |
| AHCTF1 | -0.012119097 | 0.987880903 | -0.404795173 | 0.182037192935911 | -0.682461134 | 0.539497285386707 |
| AHCY | 0.00973168123402701 | 0.00973168123402701 | -0.148586568 | 0.364983389688063 | 0.730278625155835 | 0.538825992065788 |
| AHCYL1 | 0.000581106571666732 | 0.000581106571666732 | -0.423067795 | 0.4340637421893 | 0.0224820634504978 | 0.999130434024431 |
| AHCYL2 | 0.0380745849530205 | 0.0380745849530205 | -12.50421693 | 12.838772026134 | 0.0228446323785712 | 0.999130434024431 |
| AHNAK | 0.0589269941823815 | 0.0589269941823815 | 0.449650058779432 | 0.945605295809793 | 5.61374970469238 | 0.0278215075619543 |
| AHNAK2 | 0.0202053043581073 | 0.0202053043581073 | -0.102980096 | 0.517596981527265 | 1.22577503249467 | 0.344111735183475 |
| AHR | 0.0161313037039012 | 0.0161313037039012 | -0.400218679 | 0.688678136380178 | 0.463726737650569 | 0.652016273769328 |
| AHSA1 | -0.001616763 | 0.998383237 | -0.180311212 | 0.149120971199492 | -0.165436261 | 0.898252563509827 |
| AHSG | 0.0502436994139201 | 0.0502436994139201 | -0.258525256 | 1.34358436199937 | 1.23081195647365 | 0.339659600758583 |
| AHSP | 0.00981484482971832 | 0.00981484482971832 | -1.346901949 | 1.54550544014236 | 0.12096558705143 | 0.936760076765164 |
| AIDA | 0.038526644695477 | 0.038526644695477 | 0.202552535671237 | 0.582586086012467 | 3.73271311077614 | 0.0393706486476966 |
| AIF1 | -0.060405728 | 0.939594272 | -1.43191381 | 0.274078133067718 | -1.201541905 | 0.343438429827773 |
| AIFM1 | -0.074127889 | 0.925872111 | -1.060239394 | -0.4564523 | -5.488233738 | 0.0424851052043768 |
| AIFM2 | -0.039415076 | 0.960584924 | -0.944283502 | 0.250812138405143 | -1.218435541 | 0.366275438800159 |
| AIMP1 | -0.023977654 | 0.976022346 | -0.456750453 | -0.038898337 | -2.052365808 | 0.126091835059107 |
| AIMP2 | -0.006685318 | 0.993314682 | -0.543343378 | 0.420520895748761 | -0.285631084 | 0.798307041931196 |
| AIP | -0.005182024 | 0.994817976 | -0.56741902 | 0.4663806044967 | -0.198833541 | 0.871020782244082 |
| AJUBA | -0.082886263 | 0.917113737 | -10.23207492 | 9.89835473886774 | -0.028706456 | 0.998435102357591 |
| AK1 | 0.0739447010580689 | 0.0739447010580689 | 0.0106468958546587 | 1.52232082555644 | 1.79820446623922 | 0.175707003568027 |
| AK2 | -0.033900497 | 0.966099503 | -0.773025127 | 0.0392573089565856 | -1.61820732 | 0.19785353728378 |
| AK3 | -0.023343482 | 0.976656518 | -0.637641938 | 0.13668428082564 | -1.394669021 | 0.329549069001056 |
| AK4 | 0.465825497948245 | 0.465825497948245 | -6.865738911 | 14.5597889964718 | 0.807522806880656 | 0.535425881029537 |
| AKAP1 | 0 | 1 | NA | NA | NA | NA |
| AKAP10 | -0.017581862 | 0.982418138 | -0.541799928 | 0.251781972672996 | -0.646267349 | 0.546109870133742 |
| AKAP12 | 0.0496004554910954 | 0.0496004554910954 | -0.205198852 | 1.20883886408094 | 1.31920325091695 | 0.311068488870619 |
| AKAP13 | 0.0191966229600849 | 0.0191966229600849 | -0.097063199 | 0.424261125341761 | 1.1443795663963 | 0.366275438800159 |
| AKAP17A | 0.090559451739476 | 0.090559451739476 | 0.456958482526772 | 1.66249426036688 | 3.77937949662832 | 0.0732983351754832 |
| AKAP2 | -0.012937864 | 0.987062136 | -0.496235191 | 0.250354536085239 | -0.575570693 | 0.583695260769375 |
| AKAP8 | -0.379064133 | 0.620935867 | -12.16651373 | 6.80596816385395 | -0.631820971 | 0.57448340541433 |
| AKAP8L | -0.46380315 | 0.53619685 | -12.84760889 | 6.08001829895944 | -0.804088899 | 0.536884906433788 |
| AKAP9 | -0.445426554 | 0.554573446 | -13.54939584 | 6.68244799244926 | -0.761729622 | 0.539497285386707 |
| AKR1A1 | -0.032751171 | 0.967248829 | -0.599812545 | -0.112049679 | -2.5605931 | 0.0805192700628092 |
| AKR1B1 | -0.019959011 | 0.980040989 | -0.681120902 | 0.243372030525566 | -0.954761107 | 0.444653259165786 |
| AKR1B10 | -1.063728261 | -0.063728261 | -15.74036695 | 8.82315832132846 | -0.491420622 | 0.635880209369287 |
| AKR1C2 | 0 | 1 | NA | NA | NA | NA |
| AKR1C3 | 0.487654153343065 | 0.487654153343065 | -6.008157832 | 13.3272685001012 | 0.850635122227503 | 0.517969356005393 |
| AKR1E2 | 0.0251533234496055 | 0.0251533234496055 | -0.062734713 | 0.624925228483449 | 1.44610433371888 | 0.245446187863138 |
| AKR7A2 | 0.080277492877315 | 0.080277492877315 | 0.388827065538582 | 1.32812157610266 | 3.22091438680124 | 0.048595741254695 |
| AKR7A3 | 36.398620213172 | 36.398620213172 | -0.58517103 | 18.7030135564699 | 2.11378695861896 | 0.1803694486231 |
| AKT1 | 0.046632106814399 | 0.046632106814399 | 0.109163148709679 | 0.711233112768121 | 2.60441752353464 | 0.101327857750333 |
| AKT1S1 | 0.0353808671068035 | 0.0353808671068035 | -0.31555263 | 0.968622220036415 | 1.0285334131333 | 0.407040781784975 |
| AKT2 | 0.0721143859034418 | 0.0721143859034418 | -0.162816223 | 1.35423293831186 | 1.48298609330872 | 0.257361167258283 |
| ALAD | 0.040066921566585 | 0.040066921566585 | -0.966967668 | 1.78100190844172 | 0.573130377733415 | 0.595334017564869 |
| ALB | 0.0478342195270188 | 0.0478342195270188 | -0.019109595 | 1.34407288265177 | 1.70829737944865 | 0.1803694486231 |
| ALCAM | 0.0137112363772989 | 0.0137112363772989 | -0.306979093 | 0.552359766720128 | 0.549510798012742 | 0.610896716415375 |
| ALDH16A1 | -0.008430198 | 0.991569802 | -0.358429256 | 0.188289724777813 | -0.629356319 | 0.566398099030184 |
| ALDH18A1 | -0.023833416 | 0.976166584 | -0.682040813 | 0.186544478488561 | -1.20995902 | 0.366275438800159 |
| ALDH1A1 | -0.010419138 | 0.989580862 | -0.966155705 | 0.751729417905342 | -0.22653318 | 0.842041784125343 |
| ALDH1A2 | 0 | 1 | NA | NA | NA | NA |
| ALDH1A3 | 0 | 1 | NA | NA | NA | NA |
| ALDH1B1 | 0.0145979635306571 | 0.0145979635306571 | -0.352515374 | 0.649071928203158 | 0.535931442247225 | 0.613579674112519 |
| ALDH1L1 | -0.057586881 | 0.942413119 | -0.855595514 | -0.285776561 | -3.48405353 | 0.0378017385946239 |
| ALDH1L2 | -0.037725011 | 0.962274989 | -0.700750729 | -0.099262599 | -2.682982201 | 0.110128890384053 |
| ALDH2 | -0.060380705 | 0.939619295 | -1.429491664 | 0.12829567614369 | -1.445378451 | 0.236213896431703 |
| ALDH3A1 | -36.51917548 | -35.51917548 | -20.31414853 | 0.617277917862605 | -2.117602755 | 0.1803694486231 |
| ALDH3A2 | -0.005512841 | 0.994487159 | -0.499418522 | 0.389877097323882 | -0.216326951 | 0.850956586496841 |
| ALDH3B1 | -36.9410693 | -35.9410693 | -14.01069387 | -12.37690823 | -36.34552444 | 0.000898618231383105 |
| ALDH4A1 | -0.009305894 | 0.990694106 | -0.418899013 | 0.239127132747336 | -0.492851692 | 0.637428610542048 |
| ALDH5A1 | -0.083918562 | 0.916081438 | -1.242646028 | -0.329551208 | -3.366098286 | 0.0665624432633567 |
| ALDH6A1 | 0.0245871375687692 | 0.0245871375687692 | -0.040880699 | 0.552382296903999 | 1.67530624761331 | 0.211899688328813 |
| ALDH7A1 | 0.0605429236513687 | 0.0605429236513687 | 0.383528702242657 | 0.915203160545545 | 5.13331860398643 | 0.0407987085677059 |
| ALDH9A1 | -0.033437673 | 0.966562327 | -0.659958396 | -0.067307745 | -2.183430533 | 0.118757738990998 |
| ALDOA | -0.013375534 | 0.986624466 | -0.34611432 | 0.0288715757332002 | -1.51795668 | 0.22670492938347 |
| ALDOC | 0.00085308066308072 | 0.00085308066308072 | -0.485357992 | 0.503178812308233 | 0.0343536071604743 | 0.99766937227783 |
| ALG1 | -0.079716023 | 0.920283977 | -1.531361738 | -0.014973209 | -1.861197406 | 0.174982558439639 |
| ALG11 | -0.412790205 | 0.587209795 | -14.09641762 | 7.38140998046105 | -0.700970437 | 0.541923974647687 |
| ALG2 | -0.096788178 | 0.903211822 | -1.160779115 | -0.706287404 | -7.712151346 | 0.00732298275747584 |
| ALG3 | -0.124030342 | 0.875969658 | -1.212304305 | -0.988462466 | -17.02888769 | 0.000146168596985017 |
| ALG5 | -0.119825231 | 0.880174769 | -1.43000654 | -0.91347136 | -8.13984507 | 0.00293476287878618 |
| ALG9 | -0.430761529 | 0.569238471 | -14.80160944 | 7.48998197133149 | -0.737106732 | 0.539497285386707 |
| ALKBH5 | -0.035455307 | 0.964544693 | -0.818694946 | 0.192401985661122 | -1.189428082 | 0.364844805787107 |
| ALOX12 | -34.9596896 | -33.9596896 | -13.97479937 | 7.29220342150097 | -0.707106781 | 0.539497285386707 |
| ALOX12B | 0 | 1 | NA | NA | NA | NA |
| ALOX15 | -35.1745125 | -34.1745125 | -16.21861327 | 8.46305009840512 | -0.707106781 | 0.539497285386707 |
| ALOX15B | -34.80170173 | -33.80170173 | -12.52525262 | 6.53581404226609 | -0.707106781 | 0.539497285386707 |
| ALOX5 | -0.05676142 | 0.94323858 | -0.912701317 | -0.174309175 | -2.648923222 | 0.0813439485664402 |
| ALOX5AP | -0.035566862 | 0.964433138 | -0.652005905 | -0.10039545 | -2.408163172 | 0.0926613647437377 |
| ALPL | 0.115593434608477 | 0.115593434608477 | 0.67962015052907 | 1.91711807044279 | 3.84494604534847 | 0.0395508495523429 |
| ALYREF | -0.003615419 | 0.996384581 | -0.56818208 | 0.49328502547848 | -0.138791458 | 0.923174378199409 |
| AMACR | -0.564949518 | 0.435050482 | -15.56814665 | 9.21873481302917 | -0.445912646 | 0.657887841997856 |
| AMBN | 36.1146943213584 | 36.1146943213584 | -6.238097254 | 21.1192395887666 | 1.22407644228394 | 0.366275438800159 |
| AMBP | 0.108922284950358 | 0.108922284950358 | 0.532474126772401 | 1.85616599550391 | 3.27901465966701 | 0.0531994762899972 |
| AMDHD2 | 0.0432490675836959 | 0.0432490675836959 | 0.100249450728068 | 0.750871673385226 | 2.6591014192013 | 0.114209650550459 |
| AMPD1 | 0 | 1 | NA | NA | NA | NA |
| AMPD2 | 0.0376421836359978 | 0.0376421836359978 | 0.0493361999109679 | 0.698125613685701 | 2.07551244126318 | 0.136597230826191 |
| AMPD3 | -0.022616617 | 0.977383383 | -0.754536324 | 0.343934893993168 | -0.681977735 | 0.539497285386707 |
| AMPH | 0.515465984376618 | 0.515465984376618 | -5.90250091 | 13.9302338346529 | 0.91051644761111 | 0.489795203893745 |
| AMZ2 | 0.034098970002894 | 0.034098970002894 | -12.99738561 | 13.3083803161346 | 0.0204580651338202 | 0.999130434024431 |
| ANAPC1 | 0.0131522397057086 | 0.0131522397057086 | -10.09748452 | 10.1508259203759 | 0.00455814001404093 | 0.999130434024431 |
| ANAPC13 | 1.59283255488977 | 1.59283255488977 | -4.061661952 | 15.2865384174082 | 1.00381176465114 | 0.384778549204034 |
| ANAPC4 | 0.395960236738831 | 0.395960236738831 | -6.64373149 | 12.2763764187156 | 0.669561022531841 | 0.556873624960557 |
| ANAPC5 | 0.006795665355631 | 0.006795665355631 | -9.846366567 | 9.92693568505426 | 0.00705019714090866 | 0.999130434024431 |
| ANAPC7 | 0.00808352599966501 | 0.00808352599966501 | -1.107876274 | 1.25127959201048 | 0.133257850380126 | 0.929902462812226 |
| ANG | 0.131846165910078 | 0.131846165910078 | 0.769923343587566 | 1.87115377852562 | 4.15053486955767 | 0.0218015022926752 |
| ANGPTL2 | 2.2304641542368 | 2.2304641542368 | 1.43790666510489 | 19.2278756030674 | 2.60896022942539 | 0.144707875132243 |
| ANGPTL7 | 36.4055157000373 | 36.4055157000373 | -0.579477157 | 18.784122768681 | 2.11564217015172 | 0.1803694486231 |
| ANK1 | 0.0203691917441093 | 0.0203691917441093 | -0.723439369 | 1.1685383412793 | 0.407741369896443 | 0.682516504408689 |
| ANK2 | 0.0896568086800263 | 0.0896568086800263 | 0.277031742443745 | 1.31655571321243 | 2.7053277475147 | 0.0727934915179143 |
| ANK3 | 1.09984181974639 | 1.09984181974639 | -4.294696301 | 18.5069813328038 | 1.39837838238592 | 0.339347909882518 |
| ANKEF1 | -0.089231298 | 0.910768702 | -1.805661469 | 0.182053721715785 | -1.420310628 | 0.247539986331275 |
| ANKFY1 | 0.0452836501063151 | 0.0452836501063151 | 0.126706578105913 | 0.757130619856445 | 3.10572657680394 | 0.107543581937135 |
| ANKHD1 | -1.062230537 | -0.062230537 | -16.51466883 | 4.22568130123061 | -1.332190145 | 0.357358860125466 |
| ANKLE2 | -0.988661692 | 0.011338308 | -18.58467837 | 5.61464903806104 | -1.204882725 | 0.36731973075278 |
| ANKRD1 | 0 | 1 | NA | NA | NA | NA |
| ANKRD13A | 0.410003436085455 | 0.410003436085455 | -6.768353469 | 12.8415957537211 | 0.696093484903474 | 0.544203424467315 |
| ANKRD17 | -0.059492972 | 0.940507028 | -1.053474791 | -0.000313515 | -1.747833211 | 0.179117559639718 |
| ANKRD2 | 0 | 1 | NA | NA | NA | NA |
| ANKRD22 | -35.72754417 | -34.72754417 | -16.14325772 | 4.76456257798684 | -1.224700205 | 0.366275438800159 |
| ANKRD28 | -0.420166059 | 0.579833941 | -12.31802068 | 6.36290370907337 | -0.715861902 | 0.539497285386707 |
| ANKRD35 | 0 | 1 | NA | NA | NA | NA |
| ANKRD44 | 0.00149842849029203 | 0.00149842849029203 | -0.204469902 | 0.232074935539085 | 0.115017342389437 | 0.942308526162401 |
| ANKRD50 | 35.7477492740275 | 35.7477492740275 | -4.83245973 | 16.3716359208939 | 1.22461940547932 | 0.366275438800159 |
| ANKS1A | -35.77709158 | -34.77709158 | -16.70695514 | 4.93068608989867 | -1.224739968 | 0.366275438800159 |
| ANKZF1 | -35.8117907 | -34.8117907 | -17.11402301 | 5.05108224521601 | -1.224699342 | 0.366275438800159 |
| ANO1 | 0 | 1 | NA | NA | NA | NA |
| ANO10 | -0.344038677 | 0.655961323 | -13.58079816 | 8.07548687821539 | -0.569953828 | 0.611080779751137 |
| ANO6 | 0.0273445067821721 | 0.0273445067821721 | 0.0122992942747758 | 0.52867542954143 | 2.09773259820103 | 0.169111987184949 |
| ANP32A | -0.011965 | 0.988035 | -0.389970626 | 0.130329115520905 | -0.865774165 | 0.463634410254677 |
| ANP32B | -0.03288854 | 0.96711146 | -0.773542951 | 0.117020681930187 | -1.29358492 | 0.300897614616112 |
| ANP32E | -0.06643953 | 0.93356047 | -1.080514828 | -0.147198444 | -2.571428429 | 0.110128890384053 |
| ANPEP | 0.0477717080078367 | 0.0477717080078367 | -0.107335329 | 1.14904319912036 | 1.53025165855221 | 0.233489153817926 |
| ANTXR1 | 0.0401748359134956 | 0.0401748359134956 | -0.056353611 | 0.822630599336394 | 1.73870230606598 | 0.206536129150154 |
| ANTXR2 | 0.426637759801151 | 0.426637759801151 | -6.305664381 | 12.3783911584994 | 0.725234721335329 | 0.539497285386707 |
| ANXA1 | 0.0505336981009314 | 0.0505336981009314 | 0.345411836548157 | 0.86242189213384 | 4.07591962389282 | 0.0250509813312517 |
| ANXA11 | 0.00775701552648069 | 0.00775701552648069 | -0.081704996 | 0.251627433116501 | 1.04011292460367 | 0.403808907662046 |
| ANXA2 | 0.068266693660224 | 0.068266693660224 | 0.459066280667643 | 1.22687559532267 | 3.83803628748216 | 0.0297884771457012 |
| ANXA3 | -0.087265441 | 0.912734559 | -1.399043511 | -0.422342548 | -3.411855764 | 0.0503964725846024 |
| ANXA4 | 0.0551208561184467 | 0.0551208561184467 | 0.269323750224549 | 0.987759512278041 | 3.04547579243011 | 0.0521354111315747 |
| ANXA5 | 0.0482026214750476 | 0.0482026214750476 | 0.397840369563151 | 0.835843591383733 | 4.93793166349584 | 0.0143440996769497 |
| ANXA6 | 0.0349759046691832 | 0.0349759046691832 | -0.0583137 | 0.899670587897766 | 1.73110760266809 | 0.204349177068714 |
| ANXA7 | 0.0122762938261624 | 0.0122762938261624 | -0.097567295 | 0.371971019517304 | 1.01161146977016 | 0.382116118312665 |
| ANXA8 | 0 | 1 | NA | NA | NA | NA |
| ANXA8L1 | 0 | 1 | NA | NA | NA | NA |
| ANXA9 | 0 | 1 | NA | NA | NA | NA |
| AOC3 | 0.133832850881965 | 0.133832850881965 | 0.501463784348192 | 2.26982220848325 | 3.00869835955308 | 0.0779962726389472 |
| AOX1 | 34.8170528467711 | 34.8170528467711 | -6.605730251 | 12.6592402367628 | 0.707106781186547 | 0.539497285386707 |
| AP1B1 | -0.036627987 | 0.963372013 | -0.570020265 | -0.164460945 | -3.300985405 | 0.0531994762899972 |
| AP1G1 | -0.026481727 | 0.973518273 | -0.606032091 | 0.0779128987189761 | -1.339467964 | 0.278286021962199 |
| AP1G2 | -0.443820797 | 0.556179203 | -13.17397301 | 6.48620769663656 | -0.762557437 | 0.539497285386707 |
| AP1M1 | -0.012418461 | 0.987581539 | -0.438922031 | 0.19444766610093 | -0.745690667 | 0.539497285386707 |
| AP1M2 | 0 | 1 | NA | NA | NA | NA |
| AP1S1 | 0.0334757814038617 | 0.0334757814038617 | -0.498468422 | 1.12376221588307 | 0.856946768666272 | 0.513307103063848 |
| AP1S2 | -0.061713909 | 0.938286091 | -1.204110552 | 0.0471674851155374 | -1.79325873 | 0.185946572189896 |
| AP2A1 | 0.0299815296258754 | 0.0299815296258754 | 0.0796450262058983 | 0.55712180319268 | 2.30768228615268 | 0.0963280588041145 |
| AP2A2 | 0.0429000307343196 | 0.0429000307343196 | 0.117800495323951 | 0.749257037879965 | 2.58496852068375 | 0.0987975051200024 |
| AP2B1 | 0.0397316634280269 | 0.0397316634280269 | 0.149790459648792 | 0.690278956284714 | 2.84686382393275 | 0.0743338569306362 |
| AP2M1 | 0.0336805750752599 | 0.0336805750752599 | 0.170500778487428 | 0.571618282735345 | 3.24813572069269 | 0.0462481886440984 |
| AP2S1 | 0.0350440457277528 | 0.0350440457277528 | 0.0377905429686709 | 0.666925865594039 | 2.13380915629101 | 0.148349629758657 |
| AP3B1 | 0.00186005087263927 | 0.00186005087263927 | -0.225256728 | 0.26213673207644 | 0.134572938495481 | 0.924871229952743 |
| AP3D1 | 0.0128175933548583 | 0.0128175933548583 | -0.25364919 | 0.503766582098728 | 0.594114448598285 | 0.575186153229801 |
| AP3M1 | 0.00556651729531591 | 0.00556651729531591 | -0.343207236 | 0.4516070644621 | 0.254773640249632 | 0.818049856834345 |
| AP3M2 | 1.97816761143602 | 1.97816761143602 | -0.784690024 | 20.0596953192592 | 2.07720064972819 | 0.182583953835577 |
| AP3S1 | 0.00297845076527367 | 0.00297845076527367 | -0.133537623 | 0.193026169394712 | 0.316169670515615 | 0.760373960359508 |
| AP4B1 | -35.62589623 | -34.62589623 | -15.07402813 | 4.46945029444038 | -1.221061695 | 0.366275438800159 |
| AP4S1 | -0.027437296 | 0.972562704 | -0.587669451 | 0.156935918642226 | -1.199606061 | 0.366275438800159 |
| AP5B1 | -0.019914019 | 0.980085981 | -11.01317835 | 10.8620712222734 | -0.011952407 | 0.999130434024431 |
| AP5S1 | -0.614515171 | 0.385484829 | -15.09766742 | 8.4888625210817 | -0.486471109 | 0.638269825193503 |
| AP5Z1 | -0.55027284 | 0.44972716 | -15.33612952 | 9.21608180142012 | -0.434171301 | 0.664367338267869 |
| APAF1 | -0.576401848 | 0.423598152 | -14.02465251 | 8.20194806238638 | -0.455687113 | 0.654211894695707 |
| APBB1IP | -0.46310813 | 0.53689187 | -14.25461863 | 6.76230611538683 | -0.801822836 | 0.537375417204127 |
| APBB2 | 36.3411954845775 | 36.3411954845775 | -0.531488801 | 17.9423364294414 | 2.1208461794124 | 0.1803694486231 |
| APCS | 0.0454638073769853 | 0.0454638073769853 | 0.0193294273988098 | 0.945531455489546 | 1.81920215261674 | 0.168462283243728 |
| APEH | -0.014903543 | 0.985096457 | -0.819860289 | 0.511345993070772 | -0.401016519 | 0.687598024249832 |
| APEX1 | -0.028672545 | 0.971327455 | -0.528521698 | -0.093162632 | -2.512485943 | 0.0841851891763818 |
| API5 | -0.018527861 | 0.981472139 | -0.420928385 | 0.042496678340601 | -1.717335488 | 0.228467760486518 |
| APIP | -0.015201923 | 0.984798077 | -10.89883447 | 10.7019218779274 | -0.01577316 | 0.999130434024431 |
| APMAP | -0.013968091 | 0.986031909 | -0.483895624 | 0.176116711738558 | -0.807973534 | 0.498361347437607 |
| APOA1 | 0.0460949766336375 | 0.0460949766336375 | -0.356702597 | 1.52595342870611 | 1.11041470536177 | 0.366275438800159 |
| APOA2 | 0.0340564880941132 | 0.0340564880941132 | -0.512938497 | 1.2746069343352 | 0.78099903325542 | 0.523387983067527 |
| APOA4 | 0.0606440435304622 | 0.0606440435304622 | -0.06074357 | 1.45133939056657 | 1.60031351693869 | 0.191317033179497 |
| APOB | 0.055027666375541 | 0.055027666375541 | -0.122703093 | 1.28612380917708 | 1.43184023671949 | 0.242557598169133 |
| APOBEC2 | 34.9648216451147 | 34.9648216451147 | -7.318189916 | 14.0245999644915 | 0.707106781186548 | 0.539497285386707 |
| APOBEC3A | 0 | 1 | NA | NA | NA | NA |
| APOBEC3C | -0.415208348 | 0.584791652 | -12.70063816 | 6.61792150293041 | -0.702654079 | 0.540402675468901 |
| APOBEC3G | -0.654702946 | 0.345297054 | -15.07460381 | 8.11553953846662 | -0.520355608 | 0.619453492266074 |
| APOBR | -1.087599261 | -0.087599261 | -18.00414563 | 4.33334932985826 | -1.374118116 | 0.346015552988529 |
| APOC1 | 0.0581598881000676 | 0.0581598881000676 | -0.143572772 | 1.35042779489612 | 1.41293386660876 | 0.25267531223635 |
| APOC2 | 0.0427986608406321 | 0.0427986608406321 | -0.223731111 | 1.05982311606704 | 1.20281989099816 | 0.354448343647715 |
| APOC3 | 0.0555112958541893 | 0.0555112958541893 | -0.041791969 | 1.20646125000054 | 1.77387478241343 | 0.183650685850336 |
| APOD | 0.0215572712397919 | 0.0215572712397919 | -0.260377232 | 0.734132782410405 | 0.917916483899535 | 0.455653972815953 |
| APOE | 0.00929002472799398 | 0.00929002472799398 | -0.539113681 | 0.750379914707978 | 0.292709587776712 | 0.78234850072548 |
| APOH | 0.0813704389887175 | 0.0813704389887175 | 0.294440764420653 | 1.49826139314264 | 2.88710141839443 | 0.0858002340229738 |
| APOL1 | 0.0730006691658435 | 0.0730006691658435 | -0.187189917 | 1.6130235025805 | 1.50871925917664 | 0.252060670489085 |
| APOL2 | -0.011829674 | 0.988170326 | -0.411044937 | 0.17798001076889 | -0.711035598 | 0.539497285386707 |
| APOL3 | -1.082951983 | -0.082951983 | -19.52976531 | 4.74808368142816 | -1.368516976 | 0.348478991839611 |
| APOM | 0.0753192629351972 | 0.0753192629351972 | 0.156546286668298 | 1.21436178322978 | 2.25889827352399 | 0.10404967508403 |
| APOO | -0.026249798 | 0.973750202 | -0.510054066 | 0.0137839913640597 | -1.659928947 | 0.181184707940497 |
| APOOL | -0.000653976 | 0.999346024 | -0.512503921 | 0.501012821637471 | -0.022041868 | 0.999130434024431 |
| APP | 0.0820611206766156 | 0.0820611206766156 | -0.073299693 | 1.48921478516306 | 1.92324996802466 | 0.189402068735871 |
| APPL1 | 0.0340759403344716 | 0.0340759403344716 | 0.214177631667129 | 0.47319218718396 | 4.68401023150394 | 0.0178313779071908 |
| APPL2 | 0.0596353610623893 | 0.0596353610623893 | 0.345816201260297 | 0.781020649758774 | 4.63878359862496 | 0.0209489172370465 |
| APRT | -0.012708668 | 0.987291332 | -0.430733688 | 0.158761850993861 | -0.896439771 | 0.469901501511737 |
| AQP1 | 0.0316002189473311 | 0.0316002189473311 | -0.212157251 | 0.763559711756051 | 0.979188090431973 | 0.396260923301694 |
| AQR | -0.393102374 | 0.606897626 | -13.67929099 | 7.44416840536672 | -0.664160592 | 0.559738270060838 |
| ARAF | -0.031106876 | 0.968893124 | -0.63736823 | 0.0923368609509989 | -1.305853557 | 0.295113106149497 |
| ARAP1 | -0.012849566 | 0.987150434 | -0.395630783 | 0.159381834187364 | -0.764897551 | 0.528342774559139 |
| ARCN1 | -0.019136326 | 0.980863674 | -0.644471607 | 0.22628294610131 | -1.016488243 | 0.423221837690702 |
| ARF4 | 0.0184366941474834 | 0.0184366941474834 | -0.399259882 | 0.806942905909343 | 0.708166350185246 | 0.539497285386707 |
| ARF5 | 0.0414792333826275 | 0.0414792333826275 | -0.051791746 | 0.838081892879901 | 1.53670893186638 | 0.208930833848436 |
| ARF6 | 0.00356567847028734 | 0.00356567847028734 | -0.499300795 | 0.569873841379435 | 0.132406863122105 | 0.929331185897945 |
| ARFGAP1 | -0.003680396 | 0.996319604 | -0.413620354 | 0.347398989704439 | -0.150685796 | 0.91082991373841 |
| ARFGAP2 | -0.03729113 | 0.96270887 | -0.58097011 | -0.118901885 | -2.983292184 | 0.0846619331307277 |
| ARFGAP3 | -0.053348999 | 0.946651001 | -0.917150009 | -0.13232445 | -2.661935583 | 0.10831172955225 |
| ARFGEF1 | -0.99955259 | 0.00044741 | -17.81680694 | 5.24787559290943 | -1.221930229 | 0.366275438800159 |
| ARFGEF2 | -2.059030037 | -1.059030037 | -19.79342984 | 0.0440052410169018 | -2.23873955 | 0.179833016962564 |
| ARFIP1 | 0.00494416218007222 | 0.00494416218007222 | -0.227499237 | 0.32313947150539 | 0.327422241356086 | 0.755737904002073 |
| ARFIP2 | -1.951725087 | -0.951725087 | -21.69250269 | 1.06775286197906 | -2.019371245 | 0.187606087491659 |
| ARFRP1 | -0.603328272 | 0.396671728 | -13.99071475 | 7.95375422643998 | -0.477951355 | 0.644149726560604 |
| ARG1 | 0.415618824799376 | 0.415618824799376 | -7.305317335 | 14.0197756578388 | 0.70645846189427 | 0.539497285386707 |
| ARGLU1 | -0.026151624 | 0.973848376 | -0.612323333 | 0.0981374928594947 | -1.266450559 | 0.311695808252538 |
| ARHGAP1 | 0.037950753152252 | 0.037950753152252 | 0.212610092472868 | 0.602646353379878 | 3.62701171489629 | 0.0329588009043196 |
| ARHGAP15 | -35.71973298 | -34.71973298 | -16.07725107 | 4.75999718970402 | -1.222212665 | 0.366275438800159 |
| ARHGAP17 | -0.003545789 | 0.996454211 | -0.177968922 | 0.112149078587941 | -0.422160712 | 0.677181359188282 |
| ARHGAP18 | -0.048501318 | 0.951498682 | -0.716319555 | -0.164215179 | -2.782764504 | 0.0643474047671751 |
| ARHGAP25 | -0.153213225 | 0.846786775 | -1.959013086 | -0.764138677 | -4.397411933 | 0.0383041704369688 |
| ARHGAP30 | -1.056669172 | -0.056669172 | -17.94134064 | 4.65116308612999 | -1.321463363 | 0.360245704961941 |
| ARHGAP4 | -0.482300013 | 0.517699987 | -13.91993211 | 6.34247403938307 | -0.841292224 | 0.522766152021029 |
| ARHGAP45 | -0.079494464 | 0.920505536 | -1.350249824 | -0.076585904 | -2.151317127 | 0.148635096810518 |
| ARHGAP6 | 2.12562599133736 | 2.12562599133736 | 0.519950188022291 | 18.4150024583515 | 2.37670924468384 | 0.16820338203214 |
| ARHGAP9 | -2.084595465 | -1.084595465 | -18.17800748 | -0.167900163 | -2.292005809 | 0.175707003568027 |
| ARHGDIA | 0.0135424446087522 | 0.0135424446087522 | -0.316015777 | 0.607032681625817 | 0.627187838564289 | 0.56596689778833 |
| ARHGDIB | -0.071742494 | 0.928257506 | -1.445009796 | -0.218026282 | -2.654663235 | 0.104333150555025 |
| ARHGEF1 | -0.050446233 | 0.949553767 | -0.807304887 | -0.171956922 | -2.806441847 | 0.0751894512272061 |
| ARHGEF10L | -34.92814269 | -33.92814269 | -13.67253417 | 7.13447812824502 | -0.707106781 | 0.539497285386707 |
| ARHGEF11 | 0.0430203753433874 | 0.0430203753433874 | -0.0974184 | 0.799794505154269 | 1.64094305372087 | 0.247087142236422 |
| ARHGEF12 | -0.005310629 | 0.994689371 | -0.5476476 | 0.453911855698864 | -0.174169826 | 0.892467728293403 |
| ARHGEF17 | 0.0837883451495359 | 0.0837883451495359 | 0.217631592249219 | 1.30430765412569 | 2.52940431912443 | 0.0896916084940673 |
| ARHGEF18 | -0.97782992 | 0.02217008 | -17.33908699 | 5.35082264582328 | -1.186136485 | 0.371925820869984 |
| ARHGEF2 | -0.079322341 | 0.920677659 | -1.098363781 | -0.455169147 | -5.33547961 | 0.0462481886440984 |
| ARHGEF40 | 0.027206335955989 | 0.027206335955989 | -0.362233794 | 0.866129829506732 | 0.752572841605606 | 0.53642299730317 |
| ARHGEF5 | -34.75904213 | -33.75904213 | -12.16031081 | 6.34538341144677 | -0.707106781 | 0.539497285386707 |
| ARHGEF6 | -0.025086673 | 0.974913327 | -0.402405624 | -0.100756371 | -2.966816867 | 0.0596563675187346 |
| ARHGEF7 | 0.000455017941682239 | 0.000455017941682239 | -0.256179394 | 0.264429242086265 | 0.0275528010870148 | 0.998435102357591 |
| ARID1A | -1.064583888 | -0.064583888 | -17.25666206 | 4.37266628205012 | -1.33384044 | 0.356478906085352 |
| ARID1B | 0.0175530015655095 | 0.0175530015655095 | -0.220819975 | 0.523253984622173 | 0.863523418116142 | 0.503428600091274 |
| ARID2 | -0.026885989 | 0.973114011 | -0.559952852 | 0.129412641381868 | -1.150573949 | 0.366275438800159 |
| ARIH1 | 0.0248030317042259 | 0.0248030317042259 | -0.089562222 | 0.550475301782154 | 1.24850043948095 | 0.315966997079191 |
| ARIH2 | 0.00631245947229305 | 0.00631245947229305 | -0.235577358 | 0.342616539220529 | 0.372790914124793 | 0.721293089770765 |
| ARL1 | -0.008926775 | 0.991073225 | -0.572266128 | 0.405194983209085 | -0.320993828 | 0.760407523467249 |
| ARL15 | 0.0155273629202368 | 0.0155273629202368 | -0.206428662 | 0.495834465981262 | 0.779355055005977 | 0.528130108191458 |
| ARL2 | 0.0317930626128066 | 0.0317930626128066 | -0.034171552 | 0.637898860036947 | 1.82962201349022 | 0.193828005225582 |
| ARL2BP | -0.378660227 | 0.621339773 | -13.84647869 | 7.74150190862023 | -0.63599804 | 0.5723102731992 |
| ARL3 | 0.0697685030661772 | 0.0697685030661772 | -0.170830028 | 1.47352705898556 | 1.57853565614588 | 0.247131879512359 |
| ARL6IP1 | 0.037795628935023 | 0.037795628935023 | -0.206488425 | 0.924213282411558 | 1.2213296051738 | 0.356774933113049 |
| ARL6IP4 | -36.44930508 | -35.44930508 | -19.34007343 | 0.574400302351211 | -2.120515251 | 0.1803694486231 |
| ARL6IP5 | 0.0345208534534389 | 0.0345208534534389 | 0.101474092235168 | 0.624157411494655 | 2.42618249503861 | 0.0877975327665516 |
| ARL6IP6 | 0.0016075671141494 | 0.0016075671141494 | -0.325487965 | 0.355532399622833 | 0.096084737605844 | 0.961561321805934 |
| ARL8A | 0.00900732926303573 | 0.00900732926303573 | -0.488092565 | 0.6773631047278 | 0.28241898944572 | 0.790014719434192 |
| ARL8B | 0.00567784705648029 | 0.00567784705648029 | -0.630233936 | 0.744402836249391 | 0.165908138864921 | 0.901288219322618 |
| ARMC1 | -0.493333933 | 0.506666067 | -14.68996585 | 6.53384168 | -0.864377849 | 0.511122269903125 |
| ARMC10 | -0.018837101 | 0.981162899 | -0.435715121 | 0.0804941790600427 | -1.361087996 | 0.315108462496261 |
| ARMC6 | -0.350488725 | 0.649511275 | -13.48417552 | 7.93213895876526 | -0.582491726 | 0.603041127966187 |
| ARMC8 | 0.0541225513140338 | 0.0541225513140338 | 9.81296546105596e-05 | 1.01981643771483 | 1.81758669437194 | 0.179152921529204 |
| ARMCX1 | 0.0329161032134128 | 0.0329161032134128 | -0.192637283 | 0.822722379392596 | 1.08609389017632 | 0.366275438800159 |
| ARMCX2 | 1.04660724905079 | 1.04660724905079 | -4.743295979 | 17.8768060889381 | 1.30394215662378 | 0.363596932720952 |
| ARMCX3 | -0.533389821 | 0.466610179 | -14.35435118 | 5.83888785038895 | -0.948420803 | 0.470114111141935 |
| ARMH3 | -36.42798838 | -35.42798838 | -19.05347923 | 0.563042072449857 | -2.121149465 | 0.1803694486231 |
| ARMT1 | 0.0111353781722837 | 0.0111353781722837 | -0.4794789 | 0.679685944171979 | 0.366128937188888 | 0.730217645897795 |
| ARPC1A | -0.000910858 | 0.999089142 | -0.292169643 | 0.272987302339674 | -0.067136596 | 0.981848525598098 |
| ARPC1B | -0.019375252 | 0.980624748 | -0.438381138 | 0.0202891130236467 | -2.006018111 | 0.186653518389882 |
| ARPC2 | -0.009915962 | 0.990084038 | -0.389603797 | 0.17282324540955 | -0.769833703 | 0.5377743564556 |
| ARPC3 | -0.014294526 | 0.985705474 | -0.44320894 | 0.121736819084057 | -1.152319683 | 0.366735091564231 |
| ARPC4 | -0.009344478 | 0.990655522 | -0.335784686 | 0.124921078044057 | -0.946110748 | 0.454777248349869 |
| ARPC5 | 0.00214709960875666 | 0.00214709960875666 | -0.26964918 | 0.320116297921813 | 0.14836014059127 | 0.912305317283097 |
| ARPC5L | -0.045865625 | 0.954134375 | -0.88096019 | -0.112420901 | -2.345549347 | 0.108224929033333 |
| ARPIN | 0.0952853637267147 | 0.0952853637267147 | 0.517288881507472 | 1.42139909182361 | 3.71835598269054 | 0.0309207101103281 |
| ARPP19 | 0.0622743153356233 | 0.0622743153356233 | -9.858508856 | 10.1073514206545 | 0.0215742599284146 | 0.999130434024431 |
| ARR3 | 1.08411090105487 | 1.08411090105487 | -8.173265928 | 14.7414361744579 | 0.500809362726551 | 0.631075157183946 |
| ARRB1 | -0.442652179 | 0.557347821 | -13.64059385 | 6.73639771552901 | -0.760407158 | 0.539497285386707 |
| ARRB2 | -1.611389302 | -0.611389302 | -17.53038467 | 4.54317082181926 | -1.018084368 | 0.379818912395804 |
| ARRDC1 | -0.454291745 | 0.545708255 | -13.54385388 | 6.53195338531363 | -0.784210656 | 0.539497285386707 |
| ARSA | -0.088274807 | 0.911725193 | -1.617048286 | -0.036204027 | -1.899508396 | 0.168190760193245 |
| ARSB | 0.0107259919636344 | 0.0107259919636344 | -0.1480965 | 0.348281063720459 | 0.726341543363249 | 0.539497285386707 |
| ARSD | 0.431178241221444 | 0.431178241221444 | -6.514172764 | 12.8661001780337 | 0.736731226414371 | 0.539497285386707 |
| ARSL | 34.6950522363939 | 34.6950522363939 | -6.070088064 | 11.6327340269914 | 0.707106781186548 | 0.539497285386707 |
| ARVCF | 35.8086127049185 | 35.8086127049185 | -5.039558328 | 17.0759558593104 | 1.2247447002234 | 0.366275438800159 |
| ASAH1 | -0.098662599 | 0.901337401 | -1.540135558 | -0.53034986 | -3.858968551 | 0.0437718992653409 |
| ASAP1 | 0.0239909225473531 | 0.0239909225473531 | -0.119913007 | 0.550218924399579 | 1.11531504645579 | 0.366275438800159 |
| ASAP3 | 1.04614689911317 | 1.04614689911317 | -4.467490395 | 16.8119201000196 | 1.30296104355908 | 0.363745897784638 |
| ASB6 | -35.03925521 | -34.03925521 | -14.7671681 | 7.70567010611818 | -0.707106781 | 0.539497285386707 |
| ASCC1 | -0.093843986 | 0.906156014 | -12.51558134 | 11.7282843450904 | -0.056242696 | 0.987911517811832 |
| ASCC2 | -0.060650809 | 0.939349191 | -1.118374248 | 0.0909325193782347 | -1.511601119 | 0.225406350248223 |
| ASCC3 | -0.441289247 | 0.558710753 | -14.74729522 | 7.30580135249371 | -0.758785324 | 0.539497285386707 |
| ASF1A | -0.951278204 | 0.048721796 | -16.54191493 | 5.39596013403878 | -1.143104088 | 0.383831171138254 |
| ASH2L | -36.5579007 | -35.5579007 | -20.85477616 | 0.622036838198379 | -2.119975967 | 0.1803694486231 |
| ASL | -0.027837743 | 0.972162257 | -0.519216083 | -0.005815286 | -1.876794645 | 0.174470214590395 |
| ASMTL | 0.0435396479978689 | 0.0435396479978689 | -0.289569063 | 1.15883713109958 | 1.0607851682437 | 0.368125003866442 |
| ASNS | -1.171676298 | -0.171676298 | -18.41361288 | 3.41925528166802 | -1.523911007 | 0.297619631676162 |
| ASPH | 0.0735304449147006 | 0.0735304449147006 | 0.513087586252853 | 1.04010496852386 | 5.10789452376779 | 0.0107826594792155 |
| ASPN | 0.205774691763597 | 0.205774691763597 | 1.18339220482059 | 3.74003511706077 | 3.33621863508985 | 0.0405215697770533 |
| ASPRV1 | 0 | 1 | NA | NA | NA | NA |
| ASPSCR1 | 0.0115855412469019 | 0.0115855412469019 | -0.200882413 | 0.414745605050162 | 0.60540645244093 | 0.565474048890573 |
| ASRGL1 | 0.91833099944609 | 0.91833099944609 | -9.265384893 | 15.2875624145874 | 0.425158887927359 | 0.669763107098591 |
| ASS1 | -0.020841387 | 0.979158613 | -1.337136219 | 0.904760687003976 | -0.371885383 | 0.719306708027595 |
| ATAD1 | -0.391402561 | 0.608597439 | -14.37526747 | 7.83867197800215 | -0.660215948 | 0.561015630932865 |
| ATAD3A | -0.024678519 | 0.975321481 | -0.865782416 | 0.399636254954617 | -0.67218061 | 0.539497285386707 |
| ATE1 | 0.0128436826882906 | 0.0128436826882906 | -10.5294181 | 10.5837330347511 | 0.00445120772588803 | 0.999130434024431 |
| ATF7 | 0 | 1 | NA | NA | NA | NA |
| ATG16L1 | -1.598850213 | -0.598850213 | -16.19072462 | 4.26168768999929 | -1.00919632 | 0.382684632163744 |
| ATG3 | -0.047616296 | 0.952383704 | -0.644880223 | -0.220398865 | -3.651652479 | 0.0395508495523429 |
| ATG4B | 0.793715616863897 | 0.793715616863897 | -8.001869355 | 12.2979175361274 | 0.366169448666633 | 0.717299881785849 |
| ATG5 | -0.061462706 | 0.938537294 | -11.33459972 | 10.5281055311861 | -0.063854711 | 0.983442231244532 |
| ATG7 | -0.022124742 | 0.977875258 | -0.516804839 | 0.0928622162843772 | -1.407822001 | 0.306338462175826 |
| ATG9A | 1.04185642356621 | 1.04185642356621 | -5.247952469 | 19.5327738082038 | 1.29657433641518 | 0.365181558553788 |
| ATIC | 0.00652665372577222 | 0.00652665372577222 | -0.077793502 | 0.217452238550759 | 0.823269941948459 | 0.490715864120196 |
| ATL1 | 0.0827710487678902 | 0.0827710487678902 | 0.2752280938697 | 1.23713304283264 | 2.82181393737382 | 0.0703844544157308 |
| ATL2 | -1.048593367 | -0.048593367 | -18.1945898 | 4.8169449398819 | -1.307263468 | 0.363301226496658 |
| ATL3 | 0.0475688366071309 | 0.0475688366071309 | 0.0723143263109981 | 0.884829670044294 | 2.37283938449924 | 0.136597230826191 |
| ATM | -0.036611802 | 0.963388198 | -0.69250682 | 0.0452028182090564 | -1.5900388 | 0.209204735201976 |
| ATOX1 | 0.0119692940949524 | 0.0119692940949524 | -0.044200788 | 0.277408909549153 | 1.33733552991457 | 0.300897614616112 |
| ATP12A | 0.357127113257732 | 0.357127113257732 | -6.91628924 | 11.9198217412096 | 0.592727763704011 | 0.596339870994764 |
| ATP13A1 | -0.095217255 | 0.904782745 | -1.303712929 | -0.603158792 | -4.88330758 | 0.0183568370949949 |
| ATP1A1 | -0.011715586 | 0.988284414 | -0.593478545 | 0.349226012711737 | -0.448393459 | 0.656844245290844 |
| ATP1A2 | 36.3234053928269 | 36.3234053928269 | -0.528834939 | 17.7263050589791 | 2.11994933978248 | 0.1803694486231 |
| ATP1B1 | -0.073588205 | 0.926411795 | -1.322139382 | -0.028216208 | -2.225560557 | 0.170608422382659 |
| ATP1B3 | 0.00566852825971749 | 0.00566852825971749 | -0.624756852 | 0.736546831790781 | 0.143546881135436 | 0.915921514728087 |
| ATP2A1 | -0.491345571 | 0.508654429 | -16.49371464 | 10.5394808666214 | -0.384814414 | 0.701900913939951 |
| ATP2A2 | -0.026980676 | 0.973019324 | -0.469795731 | -0.095105634 | -2.65840745 | 0.0752671805733975 |
| ATP2A3 | -0.083577935 | 0.916422065 | -1.309490206 | -0.327825947 | -3.464904906 | 0.0765716370351513 |
| ATP2B1 | -0.020074552 | 0.979925448 | -0.535574941 | 0.160404900866105 | -0.932686206 | 0.421374606651904 |
| ATP2B4 | 0.0197175797662198 | 0.0197175797662198 | -0.207059643 | 0.61580619869912 | 0.893938890301206 | 0.454697275172545 |
| ATP2C1 | -0.00526113 | 0.99473887 | -0.349726867 | 0.257245492787464 | -0.320250314 | 0.767356767117914 |
| ATP5F1A | -0.027105808 | 0.972894192 | -0.573548232 | -0.076859914 | -2.266920263 | 0.100975155016721 |
| ATP5F1B | -0.022911183 | 0.977088817 | -0.583556772 | 0.00524009097854174 | -1.709857575 | 0.1803694486231 |
| ATP5F1C | -0.025678815 | 0.974321185 | -0.748655204 | 0.193834498164317 | -1.045421737 | 0.374572457284349 |
| ATP5F1D | -0.037938093 | 0.962061907 | -0.634484879 | -0.153819588 | -3.010814129 | 0.0665100994548111 |
| ATP5IF1 | -0.043544829 | 0.956455171 | -0.868643284 | -0.020113472 | -1.835042958 | 0.167509566752535 |
| ATP5ME | -0.048858512 | 0.951141488 | -0.875397003 | -0.108327817 | -2.25424624 | 0.107543581937135 |
| ATP5MF | -0.03618488 | 0.96381512 | -0.815221262 | 0.0535004016873649 | -1.549641642 | 0.211294118489054 |
| ATP5MG | -0.029773368 | 0.970226632 | -0.456179306 | -0.192826526 | -5.138966308 | 0.0400762818574951 |
| ATP5MJ | -0.096261498 | 0.903738502 | -11.15903432 | 9.94211854254638 | -0.099884896 | 0.954604892598319 |
| ATP5MK | -0.064400591 | 0.935599409 | -1.056686011 | -0.165278271 | -2.383118396 | 0.0900859252365181 |
| ATP5PB | -0.04136913 | 0.95863087 | -0.791949486 | -0.102313844 | -2.254593624 | 0.103748773709721 |
| ATP5PD | -0.037889541 | 0.962110459 | -0.560552496 | -0.267329415 | -4.960564603 | 0.0143440996769497 |
| ATP5PF | -0.05224521 | 0.94775479 | -0.887012762 | -0.239468005 | -3.726471158 | 0.0740556368951241 |
| ATP5PO | -0.037120489 | 0.962879511 | -0.670992976 | -0.165715966 | -2.981982231 | 0.0623026754878798 |
| ATP6AP1 | -0.010237686 | 0.989762314 | -0.506131645 | 0.307830706235532 | -0.51592073 | 0.635334391248574 |
| ATP6AP2 | -0.568883119 | 0.431116881 | -15.59531472 | 9.19186242663113 | -0.449538429 | 0.656654335907203 |
| ATP6V0A1 | 0.0203134976463703 | 0.0203134976463703 | -0.038382728 | 0.419701099937739 | 1.46490593185625 | 0.236213896431703 |
| ATP6V0D1 | -0.027972471 | 0.972027529 | -0.799936344 | 0.239665895990429 | -1.161933312 | 0.371925820869984 |
| ATP6V0D2 | 0 | 1 | NA | NA | NA | NA |
| ATP6V1A | -0.013927575 | 0.986072425 | -0.407918023 | 0.111205159706315 | -0.990235715 | 0.391199220644216 |
| ATP6V1B2 | -0.036628726 | 0.963371274 | -0.890519257 | 0.118224915952423 | -1.468319181 | 0.267825774847108 |
| ATP6V1C1 | -0.036552964 | 0.963447036 | -0.826824131 | 0.135676153807266 | -1.246654791 | 0.317164124655671 |
| ATP6V1D | -0.023490647 | 0.976509353 | -0.687667814 | 0.23184704124965 | -0.86482546 | 0.465233794098861 |
| ATP6V1E1 | -0.000286038 | 0.999713962 | -0.317429592 | 0.311485189289019 | -0.016549455 | 0.999130434024431 |
| ATP6V1F | -0.000976808 | 0.999023192 | -0.396229064 | 0.376953119553996 | -0.053788844 | 0.990583831772968 |
| ATP6V1G1 | -0.036678369 | 0.963321631 | -0.992347126 | 0.265166409077264 | -1.114308416 | 0.36731973075278 |
| ATP6V1H | -0.029963449 | 0.970036551 | -0.807510437 | 0.239324307520501 | -1.025014639 | 0.393933863628188 |
| ATP7A | -1.977957765 | -0.977957765 | -19.24367853 | 0.758927150177117 | -2.077535145 | 0.182584688249216 |
| ATP8A1 | -2.110565168 | -1.110565168 | -18.68339735 | -0.399250693 | -2.345020292 | 0.171010157323467 |
| ATPAF1 | -35.80779896 | -34.80779896 | -17.07757466 | 5.047964282 | -1.223499483 | 0.366275438800159 |
| ATPAF2 | -0.062179666 | 0.937820334 | -0.824392906 | -0.252454688 | -3.954478037 | 0.0625959367903568 |
| ATRN | 0.0365669458546013 | 0.0365669458546013 | -0.346511408 | 1.00748494776509 | 0.846624178982644 | 0.475379352320255 |
| ATRX | 0.0273403832082088 | 0.0273403832082088 | -0.237230816 | 0.781628363919344 | 1.10651885938187 | 0.38162584839503 |
| ATXN10 | 0.0686451522024983 | 0.0686451522024983 | -0.088169196 | 1.33407972768693 | 1.89635507410169 | 0.20017246081774 |
| ATXN2 | -0.365253078 | 0.634746922 | -13.02494207 | 7.46048436097175 | -0.611079072 | 0.586418380770364 |
| ATXN2L | -0.025381935 | 0.974618065 | -0.496478201 | -0.007603743 | -1.970173421 | 0.172487269226316 |
| ATXN3 | 0.0476306468238762 | 0.0476306468238762 | -0.127233823 | 0.970646014321081 | 1.37811500726752 | 0.275680790309397 |
| AUH | -0.146461796 | 0.853538204 | -2.188801625 | -0.556882332 | -2.919611136 | 0.0557494448110696 |
| AUP1 | -0.031651094 | 0.968348906 | -0.428931667 | -0.14693332 | -3.578874808 | 0.0365489540648904 |
| AVEN | 0.525094114246609 | 0.525094114246609 | -8.877328956 | 14.3931743599939 | 0.413456789370745 | 0.679063111488505 |
| AXL | 0.464111154286045 | 0.464111154286045 | -6.099212353 | 12.865442572873 | 0.797473568991506 | 0.537923342132161 |
| AZGP1 | 0.047236471051025 | 0.047236471051025 | -0.111689575 | 1.09757499660863 | 1.4312007224574 | 0.247530927147405 |
| AZU1 | -0.235283211 | 0.764716789 | -3.299419175 | -1.605576803 | -5.262022724 | 0.0162947112456901 |
| B2M | -0.056612115 | 0.943387885 | -1.256958185 | 0.0602855529729606 | -1.833308506 | 0.190006156983352 |
| B3GALT6 | 0.13740934474156 | 0.13740934474156 | -12.58314571 | 13.8375434973458 | 0.0823272951075991 | 0.969742584574688 |
| B3GAT3 | -0.415808625 | 0.584191375 | -13.57250707 | 7.06971000384774 | -0.708023764 | 0.539497285386707 |
| B3GLCT | 1.18056223358824 | 1.18056223358824 | -2.981720289 | 16.6501098232773 | 1.53843710644918 | 0.292756290679516 |
| B3GNT9 | 0.63519615919737 | 0.63519615919737 | -4.284564216 | 13.6277690209315 | 1.17289313004652 | 0.375412457829878 |
| B4GALT1 | 0.0650023054734166 | 0.0650023054734166 | -0.392032457 | 1.57338702059604 | 1.28320020629376 | 0.360245704961941 |
| B4GAT1 | -35.84132562 | -34.84132562 | -17.47028898 | 5.15784999338116 | -1.224450805 | 0.366275438800159 |
| BABAM1 | -0.03307896 | 0.96692104 | -0.906211474 | 0.286043445801743 | -1.125817002 | 0.382558917227634 |
| BABAM2 | -0.415329665 | 0.584670335 | -12.71409852 | 6.63019367458082 | -0.707350508 | 0.539497285386707 |
| BACH1 | -35.81237513 | -34.81237513 | -17.1360053 | 5.06817686671644 | -1.223039357 | 0.366275438800159 |
| BAD | -0.014278598 | 0.985721402 | -10.98057623 | 10.7937927485966 | -0.014842481 | 0.999130434024431 |
| BAG1 | -1.597588079 | -0.597588079 | -17.34881756 | 4.57667346060293 | -1.007927023 | 0.383498512242829 |
| BAG2 | 0.104134438136571 | 0.104134438136571 | 0.436565142257326 | 1.61593548350236 | 3.61682712138956 | 0.0713949159081964 |
| BAG3 | 0.0528346899468781 | 0.0528346899468781 | -0.271666593 | 1.22881658662628 | 1.27770655923146 | 0.349220975890688 |
| BAG4 | -36.75229934 | -35.75229934 | -11.97140237 | -11.17985097 | -65.81751435 | 0.000260229699772871 |
| BAG5 | 0.40653252893247 | 0.40653252893247 | -6.456850419 | 12.1788099223396 | 0.689829287707873 | 0.547365405521433 |
| BAG6 | -0.001136282 | 0.998863718 | -0.113235334 | 0.0908881379210469 | -0.195729266 | 0.869890015857399 |
| BAIAP2 | -0.405881404 | 0.594118596 | -12.97439847 | 6.88159608832829 | -0.688115964 | 0.547973940707232 |
| BAIAP2L1 | 0 | 1 | NA | NA | NA | NA |
| BAIAP2L2 | 0 | 1 | NA | NA | NA | NA |
| BAK1 | -1.044205857 | -0.044205857 | -18.3844758 | 4.91312376204694 | -1.300682545 | 0.364559456625742 |
| BANF1 | 0.0334405235901769 | 0.0334405235901769 | 0.144785517926509 | 0.61565254440704 | 2.91207354570029 | 0.0665624432633567 |
| BAP18 | -0.442594249 | 0.557405751 | -13.4861789 | 6.66383298669025 | -0.760414224 | 0.539497285386707 |
| BASP1 | 0.0359095592812822 | 0.0359095592812822 | -0.098523281 | 0.840804142652468 | 1.50478526515515 | 0.253241298192162 |
| BAX | 0.0256470364785655 | 0.0256470364785655 | -0.308667131 | 0.8054880227867 | 0.810803961688717 | 0.506006432060137 |
| BAZ1A | -0.719817746 | 0.280182254 | -17.64644824 | 8.89004019091269 | -0.571145697 | 0.585817099552445 |
| BAZ1B | -0.032805739 | 0.967194261 | -0.702474471 | 0.0891946168701439 | -1.480545709 | 0.2629413517724 |
| BAZ2A | -36.38909243 | -35.38909243 | -18.55152871 | 0.552944979000474 | -2.120065809 | 0.1803694486231 |
| BBOX1 | -34.93496212 | -33.93496212 | -13.73731539 | 7.16828167532276 | -0.707106781 | 0.539497285386707 |
| BBS7 | 0.150918011770818 | 0.150918011770818 | 1.07649717062747 | 2.78789392978083 | 3.95952537347462 | 0.0280637495079433 |
| BCAM | 0.0636141021525077 | 0.0636141021525077 | 0.334008412490702 | 0.829832761316452 | 4.12973278540208 | 0.0256475213740026 |
| BCAP29 | 0.059006777313204 | 0.059006777313204 | 0.18175121827341 | 0.807642462862061 | 2.81346369843302 | 0.0686950513930572 |
| BCAP31 | -0.030681608 | 0.969318392 | -0.630116945 | -0.019776189 | -2.118734238 | 0.165023499045558 |
| BCAR1 | -35.78630847 | -34.78630847 | -16.86552434 | 5.01377986125124 | -1.218975172 | 0.366275438800159 |
| BCAS1 | -34.84673395 | -33.84673395 | -12.92238111 | 6.74304004274821 | -0.707106781 | 0.539497285386707 |
| BCAS2 | -0.033495724 | 0.966504276 | -1.099690954 | 0.500624517784367 | -0.786052976 | 0.536975363953489 |
| BCAS3 | 1.02296350448603 | 1.02296350448603 | -4.418690793 | 15.8022353438447 | 1.25884606645568 | 0.366275438800159 |
| BCAT1 | 0 | 1 | NA | NA | NA | NA |
| BCAT2 | -0.024455889 | 0.975544111 | -0.51020335 | 0.0371789600593869 | -1.504084453 | 0.219347948099816 |
| BCCIP | -36.85676662 | -35.85676662 | -14.15276496 | -10.73708573 | -16.39801576 | 0.00660663180514734 |
| BCKDHA | -0.025587363 | 0.974412637 | -0.570809767 | 0.0730631839214179 | -1.696598563 | 0.247398005265032 |
| BCKDHB | -0.04198923 | 0.95801077 | -0.615335272 | -0.147418479 | -3.361641736 | 0.0789835981194053 |
| BCKDK | -0.70444642 | 0.29555358 | -14.81231196 | 7.55982512828835 | -0.561397879 | 0.591903559837544 |
| BCL10 | -2.018075336 | -1.018075336 | -19.49348782 | 0.409441370297022 | -2.15637604 | 0.1803694486231 |
| BCL2 | -0.42641681 | 0.57358319 | -13.05144584 | 6.65472109894882 | -0.727787144 | 0.539497285386707 |
| BCL2L1 | -1.05440217 | -0.05440217 | -19.19686528 | 5.00507183986676 | -1.318269584 | 0.360763071062506 |
| BCL2L13 | -0.030597355 | 0.969402645 | -0.765037312 | 0.211588787134344 | -1.217793414 | 0.366275438800159 |
| BCL3 | -34.735763 | -33.735763 | -11.96566825 | 6.24381679145072 | -0.707106781 | 0.539497285386707 |
| BCL7C | -0.02596754 | 0.97403246 | -0.688182451 | 0.205111700553572 | -0.977941464 | 0.406765080603077 |
| BCL9L | -0.087573897 | 0.912426103 | -10.624894 | 10.2591632471996 | -0.030327527 | 0.998435102357591 |
| BCLAF1 | -0.054347218 | 0.945652782 | -0.831878031 | -0.273036784 | -3.528700717 | 0.0411209324681502 |
| BCR | -2.026392806 | -1.026392806 | -20.12483158 | 0.330682541560754 | -2.170322519 | 0.1803694486231 |
| BCS1L | -0.319959429 | 0.680040571 | -12.84479516 | 7.94630164086206 | -0.523541956 | 0.634282848629619 |
| BDH1 | -0.450424748 | 0.549575252 | -14.11451506 | 6.867401374 | -0.776563719 | 0.539497285386707 |
| BDH2 | 0.0506549912823489 | 0.0506549912823489 | 0.11798669581432 | 0.900359936653211 | 2.88180263724243 | 0.120016132092507 |
| BECN1 | -0.01840939 | 0.98159061 | -10.08507013 | 9.86477143077832 | -0.019107333 | 0.999130434024431 |
| BET1 | -36.43775599 | -35.43775599 | -19.18254149 | 0.56649200900466 | -2.121230023 | 0.1803694486231 |
| BET1L | -0.025558214 | 0.974441786 | -0.619074689 | 0.136084680805361 | -1.106925223 | 0.366275438800159 |
| BGN | 0.128588518228825 | 0.128588518228825 | 0.590050210079611 | 2.50016531130971 | 2.88942083255999 | 0.0648842992292444 |
| BHLHA15 | -37.06426303 | -36.06426303 | -14.55764676 | -14.18223214 | -172.2740401 | 0.000135517346157358 |
| BICD2 | 0.0070212364853608 | 0.0070212364853608 | -0.196673944 | 0.321589999582849 | 0.420511027432935 | 0.673967810437857 |
| BICDL2 | 0 | 1 | NA | NA | NA | NA |
| BID | -0.45708921 | 0.54291079 | -13.78619219 | 6.60545321162631 | -0.789394288 | 0.539497285386707 |
| BIN1 | -0.011910695 | 0.988089305 | -0.453987329 | 0.230979940711917 | -0.566029113 | 0.589509606996392 |
| BIN2 | -1.117038947 | -0.117038947 | -19.41564701 | 4.32105630283583 | -1.429948629 | 0.329699705770463 |
| BIRC6 | -35.73819115 | -34.73819115 | -16.26282266 | 4.79984298773821 | -1.224701573 | 0.366275438800159 |
| BLMH | 0.0203786138223779 | 0.0203786138223779 | -0.164132425 | 0.564462003164022 | 1.20676983482326 | 0.366275438800159 |
| BLOC1S1 | -1.009108224 | -0.009108224 | -19.22591706 | 5.55066638432273 | -1.238614522 | 0.366275438800159 |
| BLOC1S3 | 0.00206255232103632 | 0.00206255232103632 | -0.459130579 | 0.497873623613183 | 0.0851413162912875 | 0.96964924285189 |
| BLOC1S4 | 0.96866744831132 | 0.96866744831132 | -5.325373741 | 16.9837672323919 | 1.16896209599452 | 0.375560518269785 |
| BLOC1S5 | 0.0146635264953758 | 0.0146635264953758 | -1.763817543 | 2.02315442814167 | 0.150908255911592 | 0.914337285709502 |
| BLOC1S6 | 34.782534901171 | 34.782534901171 | -6.449557189 | 12.3599497359308 | 0.707106781186547 | 0.539497285386707 |
| BLVRA | 0.00641249830020308 | 0.00641249830020308 | -0.237858862 | 0.372012037614367 | 0.380626368835788 | 0.704421506886346 |
| BLVRB | 0.034401825873393 | 0.034401825873393 | -0.439895955 | 1.24779983660884 | 0.861795605435755 | 0.473950558779248 |
| BMP2K | -0.389340653 | 0.610659347 | -13.15903942 | 7.20642160498023 | -0.656588444 | 0.562031639448264 |
| BMS1 | -1.037989455 | -0.037989455 | -18.60066264 | 5.05484440736362 | -1.285583167 | 0.366275438800159 |
| BNIP1 | -0.004587664 | 0.995412336 | -0.225061803 | 0.141951619313604 | -0.450065018 | 0.662937154438183 |
| BNIPL | 0 | 1 | NA | NA | NA | NA |
| BOLA2B | -0.082678524 | 0.917321476 | -1.12271547 | -0.576934937 | -6.500106521 | 0.0265269392431733 |
| BOP1 | -0.041862384 | 0.958137616 | -1.048790673 | 0.313961880971678 | -1.058886079 | 0.388604115269661 |
| BORCS5 | -0.008287735 | 0.991712265 | -0.655678115 | 0.512855856005514 | -0.236296342 | 0.835152577895183 |
| BORCS6 | -0.009912663 | 0.990087337 | -0.482834813 | 0.302074945887641 | -0.480907915 | 0.652187811666889 |
| BORCS7 | 0.0056289957244719 | 0.0056289957244719 | -11.11279099 | 11.1881108090288 | 0.0058437329436548 | 0.999130434024431 |
| BPGM | 0.0500401878848156 | 0.0500401878848156 | -0.834725058 | 1.9013686661424 | 0.739551240655223 | 0.539497285386707 |
| BPHL | 0.0666784178527849 | 0.0666784178527849 | -10.46136633 | 11.333762028303 | 0.069291890304835 | 0.979078017074215 |
| BPI | -37.12751207 | -36.12751207 | -16.05012383 | -13.97776465 | -32.60662523 | 0.00120123285035057 |
| BPIFA1 | 0.0250922791743487 | 0.0250922791743487 | -11.32035286 | 11.434706774141 | 0.00869577327548324 | 0.999130434024431 |
| BPIFB1 | -34.76539321 | -33.76539321 | -12.21396135 | 6.37337884921758 | -0.707106781 | 0.539497285386707 |
| BPNT1 | -0.024105421 | 0.975894579 | -0.400407557 | -0.040685503 | -2.155542553 | 0.118102081683741 |
| BPNT2 | 0.00349661187705162 | 0.00349661187705162 | -0.32852112 | 0.399112109693809 | 0.212913466156541 | 0.861287079720759 |
| BRAT1 | 0.627675654939411 | 0.627675654939411 | -8.364029041 | 15.0908197849741 | 0.497813866036068 | 0.632355267501456 |
| BRCC3 | 0.00569089754032516 | 0.00569089754032516 | -0.309155966 | 0.413889825697741 | 0.297010106516332 | 0.785029799316631 |
| BRD1 | -36.86109224 | -35.86109224 | -13.69534714 | -11.26924248 | -23.15586377 | 0.00283516274991598 |
| BRD2 | -36.24205135 | -35.24205135 | -16.77359737 | 0.519062149697558 | -2.115236112 | 0.1803694486231 |
| BRD3 | 0.567392978834289 | 0.567392978834289 | -8.128797559 | 13.7676519482632 | 0.448191868432916 | 0.657171472553409 |
| BRD4 | -0.013881309 | 0.986118691 | -0.64328373 | 0.393310659750246 | -0.475544308 | 0.652187811666889 |
| BRD7 | -2.065675865 | -1.065675865 | -18.43542559 | -0.022649078 | -2.250817321 | 0.179117559639718 |
| BRI3BP | -0.481999438 | 0.518000562 | -14.51225979 | 6.57919417546769 | -0.836793329 | 0.523139705644887 |
| BRIX1 | -0.020554352 | 0.979445648 | -0.593065766 | 0.221914329625406 | -0.788270099 | 0.509971007859286 |
| BRK1 | -0.014667729 | 0.985332271 | -0.632647003 | 0.356494546215653 | -0.588557821 | 0.593775163912624 |
| BRMS1 | -2.010757023 | -1.010757023 | -19.50485448 | 0.466624050027857 | -2.14035113 | 0.1803694486231 |
| BROX | 0.00795293556638194 | 0.00795293556638194 | -0.155166356 | 0.302834580825985 | 0.558415550117836 | 0.593775163912624 |
| BSCL2 | -1.133414499 | -0.133414499 | -18.31932594 | 3.85855527082403 | -1.457808934 | 0.319477652441008 |
| BSDC1 | -2.006615566 | -1.006615566 | -17.91023021 | 0.461192529843335 | -2.131909972 | 0.1803694486231 |
| BSG | -0.035758102 | 0.964241898 | -0.68636543 | -0.082107343 | -2.205618825 | 0.108199037689272 |
| BST1 | 0.0241054248713865 | 0.0241054248713865 | -0.480191469 | 0.957109065806854 | 0.576299407772304 | 0.582482178881234 |
| BST2 | -2.096131396 | -1.096131396 | -22.30326733 | -0.323304625 | -2.315953765 | 0.174392217471402 |
| BTAF1 | 0.0160153059097315 | 0.0160153059097315 | -0.391710644 | 0.688900044307751 | 0.577113918356553 | 0.600281717789629 |
| BTD | 0.0573018424501277 | 0.0573018424501277 | 0.149240006216756 | 0.84585137656009 | 2.76533093145678 | 0.0926613647437377 |
| BTF3 | -0.063726295 | 0.936273705 | -1.206771159 | 0.0356251643827716 | -1.755141546 | 0.181348177308273 |
| BTF3L4 | -0.022700418 | 0.977299582 | -0.663803706 | 0.211460710619327 | -0.946499944 | 0.426992472105776 |
| BTK | -2.119405038 | -1.119405038 | -20.24454818 | -0.51162055 | -2.36407162 | 0.169701278381149 |
| BTN3A3 | -0.402559685 | 0.597440315 | -13.25692611 | 7.08322785331218 | -0.682410882 | 0.551061525652585 |
| BUB3 | -0.025924114 | 0.974075886 | -0.493660847 | -0.036772171 | -2.440541505 | 0.142396160076781 |
| BUD31 | 0.0491281902080183 | 0.0491281902080183 | -0.237154833 | 1.1959310657119 | 1.37493196616517 | 0.322232373056095 |
| BYSL | -0.064137595 | 0.935862405 | -1.199662066 | 0.107646001705282 | -1.663286129 | 0.223548214543716 |
| BZW1 | 0.0055306623925693 | 0.0055306623925693 | -0.272078736 | 0.385343955316124 | 0.363874731831946 | 0.731672476698841 |
| BZW2 | -0.000650362 | 0.999349638 | -0.579445268 | 0.567018942786398 | -0.022032632 | 0.999130434024431 |
| C11orf54 | -0.018340887 | 0.981659113 | -0.585726839 | 0.22722873226716 | -0.764169844 | 0.5238430353643 |
| C11orf68 | 0.053050782382119 | 0.053050782382119 | 0.362027786741545 | 0.698675084524841 | 5.74355601699475 | 0.0130261633012374 |
| C11orf98 | -1.010864449 | -0.010864449 | -19.07359762 | 5.49488397431328 | -1.24277655 | 0.366275438800159 |
| C12orf57 | 1.05110321611779 | 1.05110321611779 | -4.355195854 | 16.6435110845671 | 1.30959220499185 | 0.361906789426145 |
| C16orf54 | -36.96722031 | -35.96722031 | -13.60054235 | -13.26973601 | -182.7866343 | 0.000135517346157358 |
| C17orf75 | 0.0112137834740744 | 0.0112137834740744 | -0.273857652 | 0.477232131098123 | 0.500777951187503 | 0.6344490471806 |
| C19orf12 | 0.0588571599667344 | 0.0588571599667344 | 0.0439499685881544 | 0.995374964642434 | 1.93733045551338 | 0.15350046228911 |
| C19orf25 | -0.06238685 | 0.93761315 | -1.340089991 | 0.194741539991117 | -1.309594875 | 0.295113106149497 |
| C1GALT1 | -35.75268034 | -34.75268034 | -16.42968101 | 4.85099692544486 | -1.224390166 | 0.366275438800159 |
| C1QA | 0.0737247512139016 | 0.0737247512139016 | 0.443301868145467 | 1.0415351199208 | 4.77625718531925 | 0.0318429534811337 |
| C1QB | 0.0996636719584218 | 0.0996636719584218 | 0.333399824007266 | 1.76798545266616 | 3.09726625677689 | 0.0963254770149674 |
| C1QBP | -0.05380664 | 0.94619336 | -0.96442933 | -0.100628636 | -2.241378749 | 0.119181697345353 |
| C1QC | 0.0837923906745247 | 0.0837923906745247 | 0.48737985770704 | 1.35486393471031 | 4.0696258760886 | 0.0427962445758103 |
| C1QTNF5 | 0.452245636018356 | 0.452245636018356 | -6.95096203 | 14.3391941379967 | 0.78010939111005 | 0.539497285386707 |
| C1R | 0.124058972758142 | 0.124058972758142 | 0.943445950676016 | 1.75601070873744 | 5.82746306710066 | 0.00766654169092424 |
| C1RL | 34.9092034194245 | 34.9092034194245 | -7.041430896 | 13.4942181921842 | 0.707106781186548 | 0.539497285386707 |
| C1S | 0.126895851814063 | 0.126895851814063 | 1.03797769312265 | 1.75561827077959 | 6.84016988472517 | 0.00425712907930825 |
| C1orf122 | -0.5537191 | 0.4462809 | -16.20221459 | 9.70868967522812 | -0.436444814 | 0.662981888106935 |
| C1orf174 | -1.626412804 | -0.626412804 | -15.91829211 | 4.05129861654198 | -1.028405128 | 0.375642074676531 |
| C2 | 0.0850544061007237 | 0.0850544061007237 | 0.379263591100686 | 1.2610736148888 | 3.74964445749137 | 0.0597564759715416 |
| C20orf27 | 0.0134810129351194 | 0.0134810129351194 | -0.181181325 | 0.429843392628794 | 0.704474028510986 | 0.539497285386707 |
| C3 | 0.0847150118949333 | 0.0847150118949333 | 0.370183266379461 | 1.65989392071527 | 3.15796741330422 | 0.0813439485664402 |
| C4A | 0.0649591320852087 | 0.0649591320852087 | 0.0611100023058414 | 1.25034798212003 | 1.9183365433444 | 0.149285802096794 |
| C4BPA | 0.0644287820320617 | 0.0644287820320617 | 0.122298720118567 | 1.33091584320954 | 2.49579973437975 | 0.132692716166691 |
| C4BPB | 0.0199435469963315 | 0.0199435469963315 | -13.16083647 | 13.3442092300832 | 0.011970977870467 | 0.999130434024431 |
| C4B_2 | 0.0727161657076805 | 0.0727161657076805 | 0.165555536672013 | 1.3363689738402 | 2.48499710306259 | 0.114548183597488 |
| C4orf33 | 0 | 1 | NA | NA | NA | NA |
| C5 | 0.0883853946043867 | 0.0883853946043867 | 0.576625381367811 | 1.29223244501787 | 4.94196743137951 | 0.0269119029073985 |
| C5AR1 | -2.084181257 | -1.084181257 | -20.88154605 | -0.223163145 | -2.285101903 | 0.175509061115174 |
| C6 | 0.091280975190742 | 0.091280975190742 | 0.553441231231042 | 1.26444232426349 | 4.49270031162651 | 0.0196658587740803 |
| C6orf132 | 0 | 1 | NA | NA | NA | NA |
| C6orf15 | 0 | 1 | NA | NA | NA | NA |
| C6orf47 | -35.85812378 | -34.85812378 | -17.72589587 | 5.26925789257414 | -1.219019385 | 0.366275438800159 |
| C7 | 0.098142721927098 | 0.098142721927098 | 0.510564576384684 | 1.35697974873604 | 3.93905273340318 | 0.0322119000133698 |
| C7orf50 | -0.465841034 | 0.534158966 | -13.02946438 | 6.16233032488103 | -0.803975144 | 0.536884906433788 |
| C8A | 0.127882762374695 | 0.127882762374695 | 0.528886145981344 | 2.01683804932167 | 3.25795221806701 | 0.0643474047671751 |
| C8B | 0.106429696556687 | 0.106429696556687 | 0.706359283045243 | 1.61015013622734 | 4.93886320146184 | 0.0297374961775224 |
| C8G | 0.102113381607206 | 0.102113381607206 | 0.268866781837892 | 1.7611402045958 | 2.59813172551667 | 0.101710215266084 |
| C8orf33 | -0.413826986 | 0.586173014 | -12.93668139 | 6.77058813290149 | -0.703661571 | 0.540720052350242 |
| C8orf82 | 0.0145818872001889 | 0.0145818872001889 | -0.347840947 | 0.620173119219907 | 0.486814749971209 | 0.637869143504968 |
| C9 | 0.070425535881562 | 0.070425535881562 | 0.342553421450645 | 1.21997155780975 | 3.13356238458229 | 0.0509827532937539 |
| C9orf40 | 0.0384331724801573 | 0.0384331724801573 | -10.64813059 | 11.1488337597365 | 0.0397505566167979 | 0.995327285436105 |
| C9orf64 | 0.026369883204538 | 0.026369883204538 | -0.111413694 | 0.620027081953053 | 1.27523999722595 | 0.324322381401192 |
| C9orf78 | -34.6270476 | -33.6270476 | -11.09712204 | 5.79059986050732 | -0.707106781 | 0.539497285386707 |
| CA1 | 0.0276386845986341 | 0.0276386845986341 | -1.107034043 | 1.79944306776113 | 0.436110501289232 | 0.66648894933017 |
| CA13 | 0 | 1 | NA | NA | NA | NA |
| CA2 | 0.0246488690763386 | 0.0246488690763386 | -0.701468417 | 1.27944309316411 | 0.507777580961028 | 0.627253741121187 |
| CA3 | 0.0440838126715806 | 0.0440838126715806 | -1.056676353 | 1.92788313858889 | 0.560700101954531 | 0.60284290333238 |
| CA4 | -35.74917655 | -34.74917655 | -16.41889719 | 4.86829948884158 | -1.221046132 | 0.366275438800159 |
| CA6 | 34.8750733351038 | 34.8750733351038 | -6.87680552 | 13.1787296522374 | 0.707106781186548 | 0.539497285386707 |
| CA9 | 0 | 1 | NA | NA | NA | NA |
| CAB39 | -0.030132693 | 0.969867307 | -0.740757952 | 0.175233643143729 | -1.224944049 | 0.361906789426145 |
| CAB39L | 0.451634281156116 | 0.451634281156116 | -6.240009355 | 12.8223026483604 | 0.775914193616283 | 0.539497285386707 |
| CACNA1S | 0 | 1 | NA | NA | NA | NA |
| CACNA2D1 | 0.111487059459452 | 0.111487059459452 | 0.460075062969147 | 1.74044526971401 | 2.98633226575667 | 0.0537719121043574 |
| CACNB1 | 0 | 1 | NA | NA | NA | NA |
| CACTIN | -34.89980497 | -33.89980497 | -13.40659571 | 6.99570852396088 | -0.707106781 | 0.539497285386707 |
| CACYBP | 0.0145431838192331 | 0.0145431838192331 | -0.102235977 | 0.378104852613327 | 1.01917431057397 | 0.383893944845789 |
| CAD | 0.0116946391356312 | 0.0116946391356312 | -0.169172485 | 0.402066280826734 | 0.7056959520165 | 0.539497285386707 |
| CADM1 | 0.106074423529172 | 0.106074423529172 | 0.597670488134745 | 1.31159201137474 | 5.74580198364558 | 0.037807978546348 |
| CADM3 | 0.0817972575469241 | 0.0817972575469241 | -11.13685462 | 11.5073297940906 | 0.0283298021946837 | 0.998435102357591 |
| CADM4 | -1.141109949 | -0.141109949 | -15.05172895 | 8.10807767238009 | -0.525985379 | 0.616983638982579 |
| CALB1 | 35.9687675826158 | 35.9687675826158 | -5.649295976 | 19.0988518996997 | 1.22295740154166 | 0.366275438800159 |
| CALB2 | 0 | 1 | NA | NA | NA | NA |
| CALCRL | -34.63417461 | -33.63417461 | -11.15207825 | 5.81927661307865 | -0.707106781 | 0.539497285386707 |
| CALD1 | 0.103651643791844 | 0.103651643791844 | 0.597317278153002 | 1.72868447251464 | 4.11242706349387 | 0.050327216448043 |
| CALHM2 | 0.4835846339883 | 0.4835846339883 | -6.602311708 | 14.5280983081572 | 0.842989563977315 | 0.521766462620874 |
| CALML3 | 0.422678619282725 | 0.422678619282725 | -7.807297551 | 15.1629447537951 | 0.718018202956395 | 0.539497285386707 |
| CALML5 | 1.07138045552434 | 1.07138045552434 | -4.827702766 | 19.3895823582228 | 1.34565869293142 | 0.35364169857735 |
| CALR | -0.066922514 | 0.933077486 | -1.185422151 | -0.323016513 | -3.186797789 | 0.0563223029448156 |
| CALU | -0.036665563 | 0.963334437 | -1.023828967 | 0.19862252769559 | -1.184556959 | 0.347637007430169 |
| CAMK1 | 1.05170604449617 | 1.05170604449617 | -4.310729549 | 16.4346881403516 | 1.31305474919843 | 0.361906789426145 |
| CAMK1D | -0.439482681 | 0.560517319 | -12.44167038 | 6.16995322013217 | -0.751430118 | 0.539497285386707 |
| CAMK2A | 0 | 1 | NA | NA | NA | NA |
| CAMK2B | 0 | 1 | NA | NA | NA | NA |
| CAMK2D | 0.0662795128517838 | 0.0662795128517838 | 0.283001554030246 | 1.05234964138783 | 3.27797290648874 | 0.0612951020097337 |
| CAMK2G | 0.047853784768698 | 0.047853784768698 | 0.0575170747352074 | 0.817862825090744 | 2.00469288484672 | 0.134551372459989 |
| CAMLG | -1.036147258 | -0.036147258 | -17.49256793 | 4.76970504663172 | -1.284440097 | 0.366275438800159 |
| CAMP | -0.240277034 | 0.759722966 | -3.405512994 | -1.585164797 | -5.563796991 | 0.0309206522700093 |
| CAMSAP3 | 0 | 1 | NA | NA | NA | NA |
| CAND1 | 0.0226912296242356 | 0.0226912296242356 | -0.029882915 | 0.521757553754381 | 1.55709658578104 | 0.205256322967456 |
| CAND2 | 0.0980014615261085 | 0.0980014615261085 | -12.0550803 | 12.8992689250949 | 0.0585916021678468 | 0.987088251866467 |
| CANX | -0.041655164 | 0.958344836 | -0.688205836 | -0.255987289 | -3.792384254 | 0.0293796965854502 |
| CAP1 | -0.026884312 | 0.973115688 | -0.493430317 | -0.089436036 | -2.713797309 | 0.0877975327665516 |
| CAP2 | -0.004370529 | 0.995629471 | -0.462319623 | 0.380601154531995 | -0.184638487 | 0.882748127907614 |
| CAPG | -0.053236219 | 0.946763781 | -1.336453626 | 0.216277647707314 | -1.357288031 | 0.300199266232179 |
| CAPN1 | 0.0310796304480329 | 0.0310796304480329 | 0.127207504120214 | 0.557316259483046 | 2.88786580559356 | 0.0696933143714842 |
| CAPN2 | 0.0465372306559444 | 0.0465372306559444 | 0.0917586211803994 | 0.918263666982825 | 2.31093219341243 | 0.124570854411906 |
| CAPN3 | -0.088001619 | 0.911998381 | -1.270820194 | -0.515906188 | -4.111258747 | 0.0232301628989628 |
| CAPN5 | 1.07724577222042 | 1.07724577222042 | -4.677310296 | 18.9714766623324 | 1.35887628389067 | 0.350749900115579 |
| CAPN7 | -0.449003709 | 0.550996291 | -13.58853411 | 6.60706646208504 | -0.770748119 | 0.539497285386707 |
| CAPNS1 | 0.0434767452297852 | 0.0434767452297852 | -0.158695122 | 1.10280213547283 | 1.44032924684966 | 0.278781752799371 |
| CAPNS2 | 0.710370145162365 | 0.710370145162365 | -7.692933294 | 15.1654566087166 | 0.566055969536579 | 0.589099143327268 |
| CAPRIN1 | -0.000859867 | 0.999140133 | -0.272139533 | 0.256025302148924 | -0.054704797 | 0.988843825961911 |
| CAPS | 1.60308059100481 | 1.60308059100481 | -4.800649837 | 18.342845157283 | 1.01246596563396 | 0.38162584839503 |
| CAPZA1 | -0.011771292 | 0.988228708 | -0.504750176 | 0.259992830379723 | -0.669480149 | 0.551036879047474 |
| CAPZA2 | 0.0100835059778467 | 0.0100835059778467 | -0.140937232 | 0.353388708911078 | 0.74388043334821 | 0.533486957180975 |
| CAPZB | -0.000427304 | 0.999572696 | -0.18183123 | 0.172411058883041 | -0.049512274 | 0.991847671677892 |
| CARD14 | 0.138188576752257 | 0.138188576752257 | 0.446981181104739 | 2.07408112229651 | 3.06404613393765 | 0.082420509168267 |
| CARD16 | -0.401673068 | 0.598326932 | -13.52800882 | 7.23542465929774 | -0.680000698 | 0.552224117108173 |
| CARD18 | 0 | 1 | NA | NA | NA | NA |
| CARD19 | 0.0208508875924133 | 0.0208508875924133 | -0.155072436 | 0.530262380455258 | 0.979908222496794 | 0.403567666451163 |
| CARD6 | 34.6641868141549 | 34.6641868141549 | -5.94160223 | 11.386503408702 | 0.707106781186547 | 0.539497285386707 |
| CARHSP1 | 0.0187443727503216 | 0.0187443727503216 | -0.515598828 | 0.913817479376161 | 0.511178105782365 | 0.628830829680384 |
| CARM1 | 0.0166701540116654 | 0.0166701540116654 | -0.432198732 | 0.750483103483191 | 0.560067660611969 | 0.611045528370723 |
| CARMIL1 | 0.415472502790797 | 0.415472502790797 | -7.102752261 | 13.6304137884578 | 0.707355070306123 | 0.539497285386707 |
| CARNS1 | 0 | 1 | NA | NA | NA | NA |
| CARS1 | -0.048280355 | 0.951719645 | -0.879466567 | -0.081110062 | -2.618526014 | 0.135026655860983 |
| CARS2 | -0.478599029 | 0.521400971 | -14.11237847 | 6.47940982702369 | -0.832986619 | 0.525114988876483 |
| CASK | 0.0922934561514828 | 0.0922934561514828 | 0.461360923138472 | 1.34177540523463 | 3.74737616135007 | 0.0407587993982122 |
| CASP1 | -0.025109212 | 0.974890788 | -0.46052372 | -0.03504475 | -2.016954645 | 0.130567854759618 |
| CASP10 | -2.127160615 | -1.127160615 | -20.13486779 | -0.574660603 | -2.381448644 | 0.168190760193245 |
| CASP14 | 0.117791780338195 | 0.117791780338195 | -10.78244603 | 11.301952884904 | 0.040766938593346 | 0.995327285436105 |
| CASP3 | -0.068906142 | 0.931093858 | -1.004306065 | -0.319638404 | -3.392684012 | 0.0411322034316167 |
| CASP6 | -0.049522888 | 0.950477112 | -1.757327152 | 0.832212312684703 | -0.767693276 | 0.539497285386707 |
| CASP7 | 0.431075044770998 | 0.431075044770998 | -6.533716841 | 12.9314868403574 | 0.735286360984491 | 0.539497285386707 |
| CASP8 | -0.969558556 | 0.030441444 | -18.42761251 | 5.78962972784849 | -1.172969026 | 0.375358604744331 |
| CASQ1 | 0.116819434764522 | 0.116819434764522 | -11.06491061 | 11.5935475528321 | 0.0404313371436762 | 0.995327285436105 |
| CASQ2 | -34.99336757 | -33.99336757 | -14.30486167 | 7.46443354365038 | -0.707106781 | 0.539497285386707 |
| CAST | 0.0243221728441773 | 0.0243221728441773 | -0.041714441 | 0.55104273473174 | 1.80022865589667 | 0.208859935161997 |
| CASZ1 | 0 | 1 | NA | NA | NA | NA |
| CAT | 0.0247289551756695 | 0.0247289551756695 | -0.664399732 | 1.24612137887995 | 0.536090003423828 | 0.611212376392219 |
| CAV1 | 0.0765523600329709 | 0.0765523600329709 | -0.374039673 | 1.98972886458005 | 1.30454652718265 | 0.325280869222481 |
| CAV2 | 0.392493720724357 | 0.392493720724357 | -7.274349723 | 13.3681353965497 | 0.662083125623694 | 0.560747760724172 |
| CAV3 | 0 | 1 | NA | NA | NA | NA |
| CAVIN1 | 0.0549322618314667 | 0.0549322618314667 | -0.200330758 | 1.35734215650811 | 1.41782862591462 | 0.283708019792457 |
| CAVIN2 | 0.0273105508877035 | 0.0273105508877035 | -0.220829509 | 0.760961984070059 | 1.02528586337945 | 0.391366658132949 |
| CAVIN3 | 0.0482001321766741 | 0.0482001321766741 | -0.025932644 | 1.0223916649205 | 1.65076173838065 | 0.180616181545238 |
| CAVIN4 | 0 | 1 | NA | NA | NA | NA |
| CBFB | 0.0175564962228079 | 0.0175564962228079 | -0.085489302 | 0.447194714672737 | 1.35176074009302 | 0.320819522766063 |
| CBL | -36.92782018 | -35.92782018 | -14.00883341 | -12.13754517 | -31.44249082 | 0.00132803853932539 |
| CBLN4 | -0.029313508 | 0.970686492 | -0.490118241 | -0.083831839 | -2.488323485 | 0.0858002340229738 |
| CBR1 | 0.00929382630650291 | 0.00929382630650291 | -0.412827881 | 0.611539298885235 | 0.374190555940967 | 0.717614779094412 |
| CBR3 | 0.0454369304921248 | 0.0454369304921248 | -0.094445006 | 0.959577067508574 | 1.58114891087338 | 0.234042819988304 |
| CBR4 | -0.915413698 | 0.084586302 | -19.74372057 | 6.90312566033149 | -1.083461079 | 0.404862269255077 |
| CBS | 0 | 1 | NA | NA | NA | NA |
| CBX1 | 0.0489518408153084 | 0.0489518408153084 | -12.26228371 | 12.685755356432 | 0.0293773393954893 | 0.998435102357591 |
| CBX3 | -0.068396901 | 0.931603099 | -1.284055344 | 0.0250845314425715 | -1.999166435 | 0.1803694486231 |
| CBX5 | 0.0367104702912952 | 0.0367104702912952 | 0.00936642898108016 | 0.670636382249674 | 1.90722566694752 | 0.172764906484805 |
| CC2D1A | 0.00881222207904609 | 0.00881222207904609 | -0.172857226 | 0.333457244394494 | 0.549066414572809 | 0.600281717789629 |
| CC2D1B | -0.456597329 | 0.543402671 | -13.61761449 | 6.54006608711248 | -0.788854162 | 0.539497285386707 |
| CCAR1 | -0.047797273 | 0.952202727 | -0.84931988 | -0.098352928 | -2.557409079 | 0.120980763005907 |
| CCAR2 | -0.024771623 | 0.975228377 | -0.429946126 | -0.075694375 | -2.495862336 | 0.0839280070110771 |
| CCDC102A | 0.532053199173248 | 0.532053199173248 | -5.156028558 | 12.6758109101667 | 0.942269683295225 | 0.471872439498519 |
| CCDC115 | -35.94728102 | -34.94728102 | -18.80026708 | 5.54953582615071 | -1.224589363 | 0.366275438800159 |
| CCDC124 | -0.01467301 | 0.98532699 | -0.520720827 | 0.231096546631882 | -0.675242635 | 0.539497285386707 |
| CCDC134 | -0.89465578 | 0.10534422 | -16.32145366 | 5.91214130858511 | -1.051161309 | 0.419142231224745 |
| CCDC22 | 0.0118302092339808 | 0.0118302092339808 | 0.00639117208891102 | 0.217589085385743 | 1.84035184141266 | 0.16317973259033 |
| CCDC25 | 0.0259168323687165 | 0.0259168323687165 | -12.29721203 | 12.5202958639635 | 0.0155541451297148 | 0.999130434024431 |
| CCDC43 | -1.627653935 | -0.627653935 | -18.48355956 | 4.71803901153196 | -1.026842147 | 0.375998813722474 |
| CCDC47 | -0.040508419 | 0.959491581 | -0.820564822 | 0.0223611372486069 | -1.638880455 | 0.181386796687267 |
| CCDC50 | -0.360860941 | 0.639139059 | -13.77909909 | 7.96252808362602 | -0.601641644 | 0.591878904342529 |
| CCDC51 | -34.75747086 | -33.75747086 | -12.14707398 | 6.33847628706333 | -0.707106781 | 0.539497285386707 |
| CCDC6 | 0.0150813971006074 | 0.0150813971006074 | -0.108492122 | 0.406606095281057 | 1.11716010581369 | 0.36731973075278 |
| CCDC8 | 36.3749242409413 | 36.3749242409413 | -0.544871561 | 18.3675627495388 | 2.12066435515254 | 0.1803694486231 |
| CCDC80 | 0.513294964195859 | 0.513294964195859 | -5.525011802 | 12.9855151538745 | 0.904865722606518 | 0.491463670972575 |
| CCDC86 | -1.575723409 | -0.575723409 | -16.31265592 | 4.41761712637221 | -0.992811085 | 0.389883103568467 |
| CCDC88A | 0.0465801195606776 | 0.0465801195606776 | -11.41084066 | 11.785350457577 | 0.0279423802360203 | 0.998435102357591 |
| CCDC88B | -36.38499648 | -35.38499648 | -18.50485598 | 0.557299503756087 | -2.1187485 | 0.1803694486231 |
| CCDC9 | 0.0221156951105413 | 0.0221156951105413 | -0.057391556 | 0.481725840704449 | 1.50869250022139 | 0.254492945096847 |
| CCDC90B | -0.073543508 | 0.926456492 | -11.51209303 | 10.5392993698679 | -0.076374672 | 0.973519201696352 |
| CCDC93 | 0.00687570633839473 | 0.00687570633839473 | -0.177536738 | 0.30608867371263 | 0.461557769377593 | 0.652187811666889 |
| CCL18 | -36.29901657 | -35.29901657 | -17.49182567 | 0.582635936921074 | -2.105249218 | 0.180564492983725 |
| CCL19 | -36.33056367 | -35.33056367 | -17.83215056 | 0.549139046358785 | -2.115872669 | 0.1803694486231 |
| CCL22 | 0 | 1 | NA | NA | NA | NA |
| CCL26 | 0 | 1 | NA | NA | NA | NA |
| CCL5 | 0.947340936683373 | 0.947340936683373 | -8.465409067 | 14.16618697 | 0.437184106253535 | 0.662694734519085 |
| CCM2 | -1.953247611 | -0.953247611 | -18.47797974 | 0.924937250953187 | -2.027411398 | 0.187940717985324 |
| CCN5 | -34.81849198 | -33.81849198 | -12.67187448 | 6.61232293826534 | -0.707106781 | 0.539497285386707 |
| CCNDBP1 | -36.2801808 | -35.2801808 | -17.21284696 | 0.522988125697126 | -2.117615507 | 0.1803694486231 |
| CCNH | -0.103124997 | 0.896875003 | -10.45051891 | 10.0284664953565 | -0.035702414 | 0.997044355619252 |
| CCNL1 | -34.69082739 | -33.69082739 | -11.59871809 | 6.05233817751172 | -0.707106781 | 0.539497285386707 |
| CCNL2 | -34.66017602 | -33.66017602 | -11.35489209 | 5.92510709951841 | -0.707106781 | 0.539497285386707 |
| CCNT1 | -2.042191786 | -1.042191786 | -18.09245743 | 0.173260542528278 | -2.203367452 | 0.1803694486231 |
| CCNY | 0.0798948628632811 | 0.0798948628632811 | -10.17766275 | 11.2024535171665 | 0.0829944114138529 | 0.969455517331549 |
| CCS | -0.016089251 | 0.983910749 | -0.63028252 | 0.308516738392342 | -0.59432826 | 0.570925215708035 |
| CCT2 | 0.0193130539614288 | 0.0193130539614288 | -0.208895685 | 0.624717373543162 | 1.07220884982223 | 0.400909502654508 |
| CCT3 | 0.00870118154718733 | 0.00870118154718733 | -0.137893571 | 0.331829881793707 | 0.898237201490368 | 0.490002201456186 |
| CCT4 | 0.0128731155148683 | 0.0128731155148683 | -0.19578827 | 0.474667343615425 | 0.894522934648311 | 0.490002201456186 |
| CCT5 | 0.027941651734003 | 0.027941651734003 | -0.151389948 | 0.72280894373522 | 1.44699535 | 0.320031554276127 |
| CCT6A | 0.0129062694567973 | 0.0129062694567973 | -0.122106614 | 0.40986914382561 | 1.04723846842496 | 0.390098642797282 |
| CCT6B | -0.007603961 | 0.992396039 | -13.74098409 | 13.6686757224123 | -0.004564478 | 0.999130434024431 |
| CCT7 | 0.0133335887529594 | 0.0133335887529594 | -0.119932012 | 0.395483136311264 | 1.12054891702544 | 0.379541983738255 |
| CCT8 | 0.0172993887689907 | 0.0172993887689907 | -0.115114834 | 0.494116490240378 | 1.34873226949137 | 0.345139853904083 |
| CD109 | 0.133135827501755 | 0.133135827501755 | 0.721062001514917 | 1.82559421307352 | 4.07440093761159 | 0.0276221875942476 |
| CD14 | -0.029898774 | 0.970101226 | -0.93666938 | 0.322587461613808 | -0.844460571 | 0.476427474715893 |
| CD151 | 0.0667096855514117 | 0.0667096855514117 | -0.320991522 | 1.61337910439502 | 1.36754710357182 | 0.323808473447231 |
| CD163 | -0.046051334 | 0.953948666 | -1.068626795 | 0.134092852890616 | -1.364792997 | 0.272846819133432 |
| CD163L1 | -36.77295903 | -35.77295903 | -12.46202746 | -11.02314179 | -36.7293711 | 0.000878607494471725 |
| CD177 | -0.688885308 | 0.311114692 | -15.41411657 | 8.00414423927718 | -0.548129592 | 0.600843962365439 |
| CD180 | -35.78448256 | -34.78448256 | -16.79331792 | 4.95656381736169 | -1.224677708 | 0.366275438800159 |
| CD19 | -1.914471132 | -0.914471132 | -20.76935316 | 1.40733832290407 | -1.953535093 | 0.199742525014748 |
| CD1A | 0 | 1 | NA | NA | NA | NA |
| CD2 | -36.25877445 | -35.25877445 | -16.94974365 | 0.505696611649524 | -2.119942055 | 0.1803694486231 |
| CD200 | -0.003382958 | 0.996617042 | -13.14045847 | 13.1096576114687 | -0.002030184 | 0.999376962148702 |
| CD207 | 0 | 1 | NA | NA | NA | NA |
| CD209 | 1.55059203512555 | 1.55059203512555 | -4.567880024 | 16.3796092348627 | 0.975755278561497 | 0.397805341813219 |
| CD248 | 0.0850721285405123 | 0.0850721285405123 | 0.235893434235616 | 1.38524562375308 | 2.76767826638059 | 0.0968442459833395 |
| CD27 | -36.96301378 | -35.96301378 | -13.84088467 | -12.95116087 | -67.76364423 | 0.000260229699772871 |
| CD276 | 0.100955620984456 | 0.100955620984456 | 0.328737203090053 | 1.59692414796023 | 3.14555532641793 | 0.0879091406932419 |
| CD2AP | -2.062545275 | -1.062545275 | -20.53430284 | 0.013242143950824 | -2.246011222 | 0.179533257798002 |
| CD2BP2 | 0.0168413099419672 | 0.0168413099419672 | -0.14753339 | 0.468191631165379 | 1.03417711745814 | 0.400909502654508 |
| CD300A | -1.549531578 | -0.549531578 | -15.63294 | 4.36560176886225 | -0.974956874 | 0.397941901307404 |
| CD300LF | -35.09935612 | -34.09935612 | -15.39534386 | 8.03345909793901 | -0.707106781 | 0.539497285386707 |
| CD302 | 1.0165892885155 | 1.0165892885155 | -8.768790403 | 15.2628589703464 | 0.470290592287804 | 0.648276453301243 |
| CD34 | -1.010737987 | -0.010737987 | -18.31982353 | 5.24911233710127 | -1.239045446 | 0.366275438800159 |
| CD36 | 0.462792955364164 | 0.462792955364164 | -6.761967414 | 14.2454872705836 | 0.800820771556087 | 0.537659715841318 |
| CD37 | -2.105823832 | -1.105823832 | -21.67024361 | -0.421684423 | -2.334153515 | 0.172098822373015 |
| CD38 | -1.240552215 | -0.240552215 | -20.86494137 | 3.07878654533346 | -1.667146243 | 0.262299994913958 |
| CD3D | -1.06861592 | -0.06861592 | -18.16078375 | 4.54859914554054 | -1.339369356 | 0.354826113544616 |
| CD40 | -1.053600366 | -0.053600366 | -19.07241365 | 4.98681202259758 | -1.317385197 | 0.361060998583172 |
| CD44 | -0.089645263 | 0.910354737 | -1.687519864 | -0.27255821 | -2.396807883 | 0.0876189570736668 |
| CD46 | -1.036121477 | -0.036121477 | -19.74166695 | 5.37873098780333 | -1.2852476 | 0.366275438800159 |
| CD47 | -0.00018424 | 0.99981576 | -0.410759012 | 0.406906893456278 | -0.008183694 | 0.999130434024431 |
| CD48 | -36.99349212 | -35.99349212 | -14.02652671 | -13.33754808 | -89.37603904 | 0.000220658446544722 |
| CD5 | -2.113650528 | -1.113650528 | -19.42182968 | -0.463297842 | -2.347395564 | 0.170314078628347 |
| CD53 | -1.181606004 | -0.181606004 | -19.96311365 | 3.65585072778738 | -1.55278732 | 0.294043510574195 |
| CD55 | 0.0281166897779008 | 0.0281166897779008 | -0.19186156 | 0.747818483014716 | 1.07733421909994 | 0.368125003866442 |
| CD58 | -1.027364541 | -0.027364541 | -19.01038618 | 5.28178424877776 | -1.271535112 | 0.366275438800159 |
| CD59 | 0.000477154003557344 | 0.000477154003557344 | -0.427963522 | 0.438222038131727 | 0.0233481400510443 | 0.999130434024431 |
| CD5L | 0.0880014960254183 | 0.0880014960254183 | 0.247552941250729 | 1.63922824250815 | 2.49311018418063 | 0.0993457544474214 |
| CD6 | -1.06310452 | -0.06310452 | -17.42708957 | 4.40749228842941 | -1.327712553 | 0.356675249847261 |
| CD63 | -0.031316611 | 0.968683389 | -0.791212536 | 0.142089852750937 | -1.341439047 | 0.314483864815475 |
| CD68 | -1.051803554 | -0.051803554 | -17.11907774 | 4.47701154864722 | -1.311424071 | 0.36189259424674 |
| CD74 | -0.077522814 | 0.922477186 | -1.588808323 | 0.0248598035188528 | -1.694384244 | 0.1803694486231 |
| CD79A | -37.09223866 | -36.09223866 | -15.12735204 | -14.17526697 | -69.25908809 | 0.000260229699772871 |
| CD81 | 0.0306353335722437 | 0.0306353335722437 | -0.370333574 | 0.955385466667353 | 0.894725393164894 | 0.478062822881238 |
| CD82 | -0.078189408 | 0.921810592 | -1.295999845 | -0.150513682 | -2.359600343 | 0.115248223390755 |
| CD84 | -35.86618987 | -34.86618987 | -17.77321783 | 5.24673995873639 | -1.22453313 | 0.366275438800159 |
| CD8A | -2.00999671 | -1.00999671 | -19.30471472 | 0.480613282398475 | -2.140975272 | 0.1803694486231 |
| CD9 | 0.0346085510195567 | 0.0346085510195567 | -0.152555405 | 0.864403785531752 | 1.34993360421526 | 0.311502981040924 |
| CD93 | -1.006811447 | -0.006811447 | -18.84074307 | 5.47045032766309 | -1.235216171 | 0.366275438800159 |
| CD99 | 0.0428760688105439 | 0.0428760688105439 | -0.219998233 | 1.16163424109142 | 1.34258150383176 | 0.320823156369851 |
| CD99L2 | 0.477153252185942 | 0.477153252185942 | -6.090663587 | 13.2453324320024 | 0.830310744151988 | 0.526597799254599 |
| CDA | -1.991367113 | -0.991367113 | -19.10797358 | 0.599717765494089 | -2.097249777 | 0.1803694486231 |
| CDC16 | -0.559536214 | 0.440463786 | -14.11472499 | 8.40052336910849 | -0.441844217 | 0.660075326925812 |
| CDC23 | -0.043654714 | 0.956345286 | -10.85628922 | 10.3016468350776 | -0.045366884 | 0.992845174114801 |
| CDC26 | -35.85696194 | -34.85696194 | -17.66530378 | 5.21869345263401 | -1.223954557 | 0.366275438800159 |
| CDC34 | 0 | 1 | NA | NA | NA | NA |
| CDC37 | -0.010737778 | 0.989262222 | -0.260292466 | 0.0325313160136157 | -1.363411773 | 0.272753432133392 |
| CDC40 | 0.00744397725186701 | 0.00744397725186701 | -0.645954094 | 0.781894346212558 | 0.169914846654392 | 0.894771937297282 |
| CDC42 | -0.011768919 | 0.988231081 | -0.418650569 | 0.173856276042305 | -0.768320584 | 0.531441347278013 |
| CDC42BPB | 0.0642425824573915 | 0.0642425824573915 | 0.228071298062838 | 0.946028419060758 | 2.83042607911242 | 0.0591167895318091 |
| CDC42BPG | -35.006983 | -34.006983 | -14.44050277 | 7.53521255548498 | -0.707106781 | 0.539497285386707 |
| CDC42EP1 | 0.472494019308761 | 0.472494019308761 | -6.871365183 | 14.7613504365859 | 0.81925112245424 | 0.531441347278013 |
| CDC42EP4 | 1.98363832893919 | 1.98363832893919 | -0.750868853 | 20.5352294318671 | 2.08771922786566 | 0.181386796687267 |
| CDC42EP5 | 1.64330906452692 | 1.64330906452692 | -4.388754118 | 17.6034061965102 | 1.04007705897265 | 0.371925820869984 |
| CDC5L | -0.023920876 | 0.976079124 | -0.550641417 | 0.0121056961954712 | -1.741274522 | 0.1803694486231 |
| CDC73 | -0.022934106 | 0.977065894 | -0.449327212 | 0.0195734103131049 | -1.603270512 | 0.19263877608386 |
| CDCP1 | 0 | 1 | NA | NA | NA | NA |
| CDH1 | 0.0325239549125748 | 0.0325239549125748 | -0.640762533 | 1.24082842789209 | 0.662087110043652 | 0.553503966695302 |
| CDH11 | 0.0853889023615129 | 0.0853889023615129 | 0.205511234755732 | 1.47115707755778 | 2.6650623397983 | 0.110889026522763 |
| CDH13 | 0.0269494762178472 | 0.0269494762178472 | -0.665615794 | 1.15976602828842 | 0.533301001788188 | 0.621617284366183 |
| CDH3 | -1.225474435 | -0.225474435 | -14.92098569 | 7.70742742169421 | -0.563150426 | 0.592942304265312 |
| CDH5 | 0.0570259481892018 | 0.0570259481892018 | -0.115006463 | 1.14816902539823 | 1.41971712634782 | 0.247398005265032 |
| CDIPT | 0.0279096544135855 | 0.0279096544135855 | -0.070876903 | 0.633956989984576 | 1.40242973562424 | 0.258285935495512 |
| CDK1 | -34.79484144 | -33.79484144 | -12.46583405 | 6.50480878478432 | -0.707106781 | 0.539497285386707 |
| CDK17 | -0.54198279 | 0.45801721 | -15.78064313 | 9.56626398479922 | -0.427251528 | 0.668435905102326 |
| CDK2 | -34.80641463 | -33.80641463 | -12.56623625 | 6.55719975324335 | -0.707106781 | 0.539497285386707 |
| CDK2AP1 | 34.7273740070147 | 34.7273740070147 | -6.207615578 | 11.8962921447014 | 0.707106781186548 | 0.539497285386707 |
| CDK4 | 0.048987798617916 | 0.048987798617916 | -0.06912408 | 0.930756632041478 | 1.57426549884041 | 0.216602981026048 |
| CDK5 | 0.390185252693495 | 0.390185252693495 | -7.02411793 | 12.8415853697268 | 0.658422714582137 | 0.561394998415846 |
| CDK5RAP3 | -0.087665441 | 0.912334559 | -0.999473363 | -0.705523735 | -10.03784109 | 0.000497992726450439 |
| CDK6 | 0.0373984866768653 | 0.0373984866768653 | -0.373013668 | 1.03553097306801 | 0.898026021750842 | 0.464950524194327 |
| CDK7 | -1.593251251 | -0.593251251 | -17.2909021 | 4.5799696242083 | -1.005584348 | 0.384282553981175 |
| CDK9 | -0.490183888 | 0.509816112 | -12.69866101 | 5.724626127 | -0.851528765 | 0.517642586759047 |
| CDKAL1 | -36.43963175 | -35.43963175 | -19.20743011 | 0.567160819752988 | -2.121244655 | 0.1803694486231 |
| CDKN1B | -0.531976695 | 0.468023305 | -13.39696181 | 5.49025816859594 | -0.941320058 | 0.473444566548856 |
| CDKN2A | 1.632808401 | 2.632808401 | -4.296345895 | 17.0094328822225 | 1.0327061706379 | 0.374430460168872 |
| CDKN2AIP | -0.352381647 | 0.647618353 | -14.32345953 | 8.40687954585888 | -0.585738844 | 0.601226729384732 |
| CDKN2B | 0 | 1 | NA | NA | NA | NA |
| CDKN2C | -0.019297704 | 0.980702296 | -0.572260681 | 0.207931100071639 | -0.818368743 | 0.494510273520826 |
| CDS2 | -0.043937991 | 0.956062009 | -0.787755301 | -0.037057026 | -2.017632528 | 0.152974682593197 |
| CDSN | 34.8311508469278 | 34.8311508469278 | -6.670597801 | 12.7835525941361 | 0.707106781186547 | 0.539497285386707 |
| CDV3 | -0.033941178 | 0.966058822 | -0.701912796 | -0.006822467 | -1.795525335 | 0.174773300129766 |
| CEACAM1 | 0 | 1 | NA | NA | NA | NA |
| CEACAM5 | 0 | 1 | NA | NA | NA | NA |
| CEACAM6 | -36.97516325 | -35.97516325 | -14.2436878 | -12.77493626 | -41.39624801 | 0.000642824954077901 |
| CEACAM8 | -36.75002391 | -35.75002391 | -12.47665321 | -10.6381145 | -28.29194735 | 0.0017055451325429 |
| CEBPB | -2.040558618 | -1.040558618 | -20.47798886 | 0.210756857121194 | -2.199962193 | 0.1803694486231 |
| CEBPZ | -1.022881619 | -0.022881619 | -17.91962959 | 5.02746908019735 | -1.26308117 | 0.366275438800159 |
| CELF1 | -0.411661938 | 0.588338062 | -14.31582372 | 7.51461132593676 | -0.699146131 | 0.542872872129736 |
| CELF2 | -0.130863931 | 0.869136069 | -1.874656733 | -0.521352913 | -3.527687433 | 0.0648842992292444 |
| CELSR2 | -0.04736969 | 0.95263031 | -9.817209961 | 9.63275069977158 | -0.016413398 | 0.999130434024431 |
| CEMIP | 0 | 1 | NA | NA | NA | NA |
| CEMIP2 | 0.896365931366981 | 0.896365931366981 | -9.825492222 | 16.0141855723865 | 0.414915396464205 | 0.677370666860611 |
| CENPB | -36.93667156 | -35.93667156 | -13.39622644 | -12.91106137 | -122.0204533 | 0.000156652813393394 |
| CENPC | -2.130630038 | -1.130630038 | -18.09424717 | -0.551401771 | -2.38745271 | 0.167509566752535 |
| CENPV | 0.696961947998108 | 0.696961947998108 | -7.561984884 | 14.6946041666671 | 0.555057393766128 | 0.596296453214701 |
| CEP170 | 0.0939135700992061 | 0.0939135700992061 | 0.474491764144731 | 1.14526167609152 | 4.41038277533728 | 0.0279939804710251 |
| CEP170B | 0 | 1 | NA | NA | NA | NA |
| CEP89 | 0 | 1 | NA | NA | NA | NA |
| CEPT1 | -0.05821147 | 0.94178853 | -0.935320847 | -0.059577905 | -1.997344599 | 0.139853840716483 |
| CERCAM | 0.560318745240197 | 0.560318745240197 | -5.805392937 | 15.1764694997379 | 1.00451353270777 | 0.441560186942964 |
| CERS2 | -0.001110411 | 0.998889589 | -0.262040895 | 0.241403827488704 | -0.07393984 | 0.975711635982561 |
| CERS6 | -35.65361992 | -34.65361992 | -15.34582708 | 4.53549473279265 | -1.223601154 | 0.366275438800159 |
| CERT1 | -0.020767223 | 0.979232777 | -0.538687053 | 0.169861188680701 | -0.901038048 | 0.44050140954373 |
| CES1 | -1.003542809 | -0.003542809 | -19.73629134 | 5.70741994511174 | -1.221966422 | 0.366275438800159 |
| CES2 | 0.940696984152271 | 0.940696984152271 | -8.416980949 | 14.0295063713022 | 0.433882058505451 | 0.664367338267869 |
| CETP | 0.125890759639831 | 0.125890759639831 | -11.00300007 | 12.0022992300557 | 0.0752993212239536 | 0.97429540885804 |
| CFAP20 | 0.0357447200880256 | 0.0357447200880256 | 0.0162848921030386 | 0.67886713244837 | 1.84792377715025 | 0.167106013143295 |
| CFAP410 | 0.099992663099705 | 0.099992663099705 | 0.0526459735370865 | 1.64272742559124 | 2.31547270329209 | 0.166464953611858 |
| CFB | 0.0764880871502543 | 0.0764880871502543 | 0.275821342808844 | 1.40426251906231 | 2.82195513581496 | 0.0841851891763818 |
| CFD | 0.0527520995986598 | 0.0527520995986598 | -0.208543876 | 1.20688596938249 | 1.2211393770367 | 0.327645414009843 |
| CFDP1 | 0.562638064764428 | 0.562638064764428 | -9.030820823 | 15.2173442928577 | 0.444161781052456 | 0.658317574188362 |
| CFH | 0.106138818985478 | 0.106138818985478 | 0.632556646842811 | 1.67830607935898 | 4.33990949911567 | 0.0418806032498226 |
| CFHR1 | 0.065391410488613 | 0.065391410488613 | -0.076887625 | 1.3244929262605 | 1.82024657680268 | 0.197368473432119 |
| CFHR2 | 0.102773596348076 | 0.102773596348076 | -0.468112743 | 2.278906041 | 1.18616425486455 | 0.35364169857735 |
| CFI | 0.070295633737695 | 0.070295633737695 | 0.352143052396522 | 1.08831094017072 | 3.8828539334678 | 0.0538679412155676 |
| CFL1 | -0.000679938 | 0.999320062 | -0.308356903 | 0.29287347184127 | -0.044629084 | 0.993220185640509 |
| CFL2 | 0.0354526888056228 | 0.0354526888056228 | 0.0370091846351228 | 0.622385982770323 | 2.41738241333766 | 0.151746908426006 |
| CFP | 0.0364614805105825 | 0.0364614805105825 | 0.179199969511913 | 0.511809187551481 | 3.66605160530064 | 0.0357523135237048 |
| CGAS | -1.032047727 | -0.032047727 | -18.26128211 | 5.01771987805749 | -1.279401695 | 0.366275438800159 |
| CGGBP1 | -0.424260281 | 0.575739719 | -14.09315074 | 7.22176942645053 | -0.724540941 | 0.539497285386707 |
| CGN | 1.11140973847042 | 1.11140973847042 | -8.287192813 | 15.1636689111921 | 0.51324042913978 | 0.623880968411888 |
| CGREF1 | 1.78850667003721 | 1.78850667003721 | -3.51824364 | 16.8449097801997 | 1.13779021104262 | 0.361578179655194 |
| CHAD | 0.157491928706969 | 0.157491928706969 | 0.812524085491846 | 2.36432975824113 | 3.87167923006396 | 0.0444925432867386 |
| CHAF1B | 0 | 1 | NA | NA | NA | NA |
| CHAMP1 | 0.376396806691662 | 0.376396806691662 | -7.062787839 | 12.5890633227378 | 0.63051797724767 | 0.575186153229801 |
| CHCHD1 | -1.144631234 | -0.144631234 | -17.90711055 | 3.69907968376469 | -1.474866774 | 0.315001123360638 |
| CHCHD2 | -0.013924268 | 0.986075732 | -0.453922821 | 0.189293360215204 | -0.866218921 | 0.5000399473548 |
| CHCHD3 | -0.028948869 | 0.971051131 | -0.482100254 | -0.123800102 | -3.013317446 | 0.0574056423986682 |
| CHCHD4 | -0.097255042 | 0.902744958 | -1.207659573 | -0.564295614 | -4.789113818 | 0.0144289042301664 |
| CHCHD6 | -0.388132494 | 0.611867506 | -13.38265007 | 7.3467482428392 | -0.653579281 | 0.563028958254002 |
| CHD1L | -34.52155764 | -33.52155764 | -10.31465533 | 5.38230015911406 | -0.707106781 | 0.539497285386707 |
| CHD3 | 0.0266271699693854 | 0.0266271699693854 | -10.24493724 | 10.577857599225 | 0.0276654739948362 | 0.998435102357591 |
| CHD4 | 0.00558190306217389 | 0.00558190306217389 | -0.365881185 | 0.473641924505683 | 0.224493598394404 | 0.842309962811171 |
| CHERP | -0.03504116 | 0.96495884 | -0.615555491 | -0.024934409 | -1.88392198 | 0.155466238836064 |
| CHGA | 0 | 1 | NA | NA | NA | NA |
| CHI3L1 | -36.56880066 | -35.56880066 | -21.01888953 | 0.632707410547711 | -2.11881019 | 0.1803694486231 |
| CHID1 | -0.063365875 | 0.936634125 | -1.233664685 | -0.025849952 | -2.268180541 | 0.170935276601406 |
| CHIT1 | -34.91556241 | -33.91556241 | -13.55382812 | 7.07253600985482 | -0.707106781 | 0.539497285386707 |
| CHKB | 0.0454989051950639 | 0.0454989051950639 | -9.839791807 | 10.3911660035312 | 0.0471665278392627 | 0.99228196233491 |
| CHMP1A | 0.0226692733440552 | 0.0226692733440552 | -0.02839138 | 0.483291120397829 | 1.73793048722683 | 0.199742525014748 |
| CHMP1B | 0.00338711344441167 | 0.00338711344441167 | -0.131977983 | 0.198124681257984 | 0.370173238795425 | 0.718222338271579 |
| CHMP2A | 8.94530644665328e-06 | 8.94530644665328e-07 | -0.219976024 | 0.220154381923515 | 0.000703163942220239 | 0.999577493567163 |
| CHMP2B | -0.003343181 | 0.996656819 | -0.429259758 | 0.367322623152805 | -0.166009945 | 0.902427899166895 |
| CHMP3 | 0.0475814867394668 | 0.0475814867394668 | 0.10197015870584 | 0.819247203165602 | 2.54614161961921 | 0.115722268902765 |
| CHMP4A | -0.062324847 | 0.937675153 | -1.375878424 | 0.264594907779521 | -1.470425096 | 0.306939460163683 |
| CHMP4B | 0.0011234746074056 | 0.0011234746074056 | -0.15544891 | 0.17839406497578 | 0.120462634345465 | 0.93704981977343 |
| CHMP5 | 0.0133886873080689 | 0.0133886873080689 | -0.220433832 | 0.460285610042103 | 0.6297853254897 | 0.555441009809081 |
| CHMP6 | 0.0308079021207932 | 0.0308079021207932 | 0.0469646461444402 | 0.568065739124771 | 2.04261708503616 | 0.127486195662268 |
| CHMP7 | -1.965441286 | -0.965441286 | -19.2741851 | 0.857539319746717 | -2.050981874 | 0.185146238018798 |
| CHORDC1 | -36.82603394 | -35.82603394 | -12.37225257 | -11.99299517 | -144.5715553 | 0.000135517346157358 |
| CHP1 | 0.00715994265848161 | 0.00715994265848161 | -0.277223505 | 0.414436659589996 | 0.36149855665501 | 0.724657663497786 |
| CHPF | 0.491487771125139 | 0.491487771125139 | -6.234690967 | 13.9641698674048 | 0.860061546998612 | 0.513307103063848 |
| CHPF2 | 0.977825530498258 | 0.977825530498258 | -5.333167636 | 17.2532890027101 | 1.18491569947318 | 0.372046232587338 |
| CHPT1 | 0.638307222338124 | 0.638307222338124 | -8.36523517 | 15.2681641784026 | 0.506776396038469 | 0.627358090184273 |
| CHRAC1 | -0.497933486 | 0.502066514 | -14.36930153 | 6.31139354199455 | -0.870851648 | 0.507417984352627 |
| CHST14 | 0.130584324568354 | 0.130584324568354 | 0.504896980310391 | 1.80717689475955 | 3.69771724955823 | 0.0693365999576725 |
| CHST2 | -36.87886482 | -35.87886482 | -12.97764695 | -12.29638403 | -83.48450005 | 0.000220658446544722 |
| CHTOP | -0.049199504 | 0.950800496 | -1.295564428 | 0.38168394656767 | -1.027964675 | 0.392756414023024 |
| CHUK | 0.0364271645076219 | 0.0364271645076219 | -0.087631493 | 0.707681042949247 | 1.42494587699846 | 0.265540972734776 |
| CIAO1 | -1.013890379 | -0.013890379 | -18.02997581 | 5.16025652221395 | -1.247685729 | 0.366275438800159 |
| CIAO2A | -0.624452804 | 0.375547196 | -15.2030717 | 8.45321093051938 | -0.495314444 | 0.633775138389975 |
| CIAPIN1 | -0.002119974 | 0.997880026 | -0.222943344 | 0.182065818430141 | -0.209710242 | 0.862456771764846 |
| CIB1 | -36.91019662 | -35.91019662 | -13.49780428 | -12.33112031 | -49.81947912 | 0.00044044858460524 |
| CIC | -0.395200098 | 0.604799902 | -11.74936081 | 6.36129351730348 | -0.666263675 | 0.558116976763512 |
| CILP | 0.92813125263245 | 0.92813125263245 | -9.486649613 | 15.7319772345158 | 0.429425808369578 | 0.667181703153712 |
| CILP2 | 35.922116635313 | 35.922116635313 | -5.458526859 | 18.4801346594602 | 1.22408290732532 | 0.366275438800159 |
| CIRBP | 0.0350624712226543 | 0.0350624712226543 | -0.105456629 | 0.819578779933325 | 1.56868721729903 | 0.256640101335578 |
| CISD1 | -0.0081795 | 0.9918205 | -0.638778976 | 0.480004440969619 | -0.247193402 | 0.821512918961126 |
| CISD2 | -0.09807906 | 0.90192094 | -1.401855017 | -0.609474101 | -4.47819481 | 0.0203094444224222 |
| CKAP4 | 0.0454404941504524 | 0.0454404941504524 | -0.052450041 | 1.07833030232188 | 1.77339419595195 | 0.191795561840914 |
| CKAP5 | 0.0145477539440229 | 0.0145477539440229 | -0.123339077 | 0.40536049842527 | 0.925950900399603 | 0.426010674809809 |
| CKB | 0.0320249026253629 | 0.0320249026253629 | -0.417503262 | 1.03985770116531 | 0.847805742497456 | 0.5000399473548 |
| CKM | -0.299763501 | 0.700236499 | -13.68408217 | 8.76434849363062 | -0.485864135 | 0.653199750821584 |
| CKMT1B | -1.616537956 | -0.616537956 | -17.78218886 | 4.61335947052864 | -1.017614039 | 0.379841861092033 |
| CKMT2 | 34.9654386994405 | 34.9654386994405 | -7.321320645 | 14.0305997016363 | 0.707106781186547 | 0.539497285386707 |
| CLASP1 | 0.0467218890114445 | 0.0467218890114445 | -0.683778072 | 1.53097720784547 | 0.703159938691949 | 0.539497285386707 |
| CLASP2 | 0.0194794820535928 | 0.0194794820535928 | -0.104203451 | 0.465261172234458 | 1.11126957865554 | 0.366275438800159 |
| CLC | -1.998986365 | -0.998986365 | -18.75160791 | 0.523820685462322 | -2.112333698 | 0.1803694486231 |
| CLCA2 | 0.0305374543174685 | 0.0305374543174685 | -10.7482612 | 10.8805337076598 | 0.0105824874362791 | 0.999130434024431 |
| CLCA4 | -36.01210133 | -35.01210133 | -19.66971367 | 5.81004959647763 | -1.224061963 | 0.366275438800159 |
| CLCC1 | -0.425842323 | 0.574157677 | -14.13417408 | 7.2187271058221 | -0.727706821 | 0.539497285386707 |
| CLCN7 | -0.008916307 | 0.991083693 | -0.209578665 | 0.0402373172734851 | -1.172968305 | 0.348478991839611 |
| CLDN1 | 0.988645403394676 | 0.988645403394676 | -8.932089494 | 15.3176616668244 | 0.457643600518683 | 0.653774096383628 |
| CLDN4 | 0 | 1 | NA | NA | NA | NA |
| CLEC11A | 0.145475288733275 | 0.145475288733275 | 0.333586879690875 | 2.5613287106488 | 2.8711353427411 | 0.120250924847184 |
| CLEC14A | 0.0556782569376311 | 0.0556782569376311 | -10.05795706 | 10.7529033125327 | 0.05779777642975 | 0.987185871906287 |
| CLEC2B | -36.43005939 | -35.43005939 | -19.08141589 | 0.564416390249591 | -2.121027356 | 0.1803694486231 |
| CLEC3B | 0.138034710011697 | 0.138034710011697 | 0.83239662539258 | 1.9549817708587 | 4.77052936386101 | 0.0316815233111557 |
| CLIC1 | -0.026327037 | 0.973672963 | -0.547964067 | -0.031585855 | -1.999651807 | 0.144393296415223 |
| CLIC2 | -0.011319442 | 0.988680558 | -0.449516941 | 0.238483009673004 | -0.566334077 | 0.595197901360214 |
| CLIC3 | -35.90803999 | -34.90803999 | -18.29563142 | 5.40046000705346 | -1.224606301 | 0.366275438800159 |
| CLIC4 | -0.019890479 | 0.980109521 | -0.514268744 | 0.111170785556974 | -1.117750084 | 0.366275438800159 |
| CLIC5 | 34.8415198946998 | 34.8415198946998 | -6.718713935 | 12.8757624898677 | 0.707106781186547 | 0.539497285386707 |
| CLIC6 | -0.313153216 | 0.686846784 | -11.99573183 | 7.4840093942274 | -0.507653117 | 0.642390206958208 |
| CLINT1 | -0.058532023 | 0.941467977 | -0.908607336 | -0.205813785 | -2.801955584 | 0.0670680593256903 |
| CLIP1 | 0.0126073794399828 | 0.0126073794399828 | -0.39464887 | 0.624893217861371 | 0.464543167465261 | 0.657423743030449 |
| CLIP2 | 0.0458457257837484 | 0.0458457257837484 | -10.530745 | 11.1270618178321 | 0.0476510522516257 | 0.992135986860447 |
| CLMN | -1.579758843 | -0.579758843 | -15.76709477 | 4.24766877948697 | -0.995828027 | 0.388604115269661 |
| CLN6 | -0.351012139 | 0.648987861 | -13.47009296 | 7.92289037853922 | -0.581304237 | 0.603688346125979 |
| CLNS1A | -0.005160246 | 0.994839754 | -0.254302351 | 0.157982370857891 | -0.405664988 | 0.684352245564589 |
| CLPB | -0.089493942 | 0.910506058 | -1.130156254 | -0.516752033 | -4.840742985 | 0.0196663010362462 |
| CLPP | -0.082707557 | 0.917292443 | -1.133628754 | -0.430847488 | -3.979617188 | 0.0316815233111557 |
| CLPTM1 | -0.433456588 | 0.566543412 | -14.75160551 | 7.42329858510428 | -0.743108137 | 0.539497285386707 |
| CLPTM1L | -0.533414342 | 0.466585658 | -14.95437893 | 6.08226117224002 | -0.94880288 | 0.470050973006295 |
| CLPX | -0.526457734 | 0.473542266 | -13.12028869 | 5.41588038877868 | -0.931747527 | 0.477554849105054 |
| CLSTN1 | 2.14048083899396 | 2.14048083899396 | 0.689738502819085 | 19.0109904648836 | 2.40320726635846 | 0.164823616087717 |
| CLTA | 0.0173926898581036 | 0.0173926898581036 | -0.013129056 | 0.413070760283836 | 1.69305020294076 | 0.183086734459121 |
| CLTB | 0.00999159097678801 | 0.00999159097678801 | -0.165807463 | 0.378424079763466 | 0.684516234666878 | 0.539497285386707 |
| CLTC | 0.0219793844988733 | 0.0219793844988733 | -0.06620819 | 0.562419061278305 | 1.39217061865404 | 0.264347105058371 |
| CLU | 0.0601506675060871 | 0.0601506675060871 | 0.181666976198485 | 1.10706334606358 | 2.7628019062348 | 0.100763636811655 |
| CLUH | -1.635603006 | -0.635603006 | -17.30273165 | 4.36399753353418 | -1.033595306 | 0.374281845155447 |
| CLYBL | -36.91130935 | -35.91130935 | -13.44266397 | -12.40618974 | -56.12144814 | 0.00036478881262549 |
| CMA1 | -0.424560716 | 0.575439284 | -16.09735095 | 8.22382582036231 | -0.717736567 | 0.539497285386707 |
| CMAS | 0.00312558481928873 | 0.00312558481928873 | -0.208944219 | 0.269701292068132 | 0.232373252615377 | 0.836744175813969 |
| CMBL | 0.103339618978511 | 0.103339618978511 | 0.783192355043188 | 1.22517993974851 | 7.8620803494008 | 0.00157876589764 |
| CMC2 | -0.000671149 | 0.999328851 | -10.91627555 | 10.9074752941572 | -0.000697701 | 0.999577493567163 |
| CMC4 | -1.558622882 | -0.558622882 | -17.72748567 | 4.89516066604567 | -0.981519453 | 0.394612911428318 |
| CMIP | 0 | 1 | NA | NA | NA | NA |
| CMPK1 | -0.004559302 | 0.995440698 | -0.565698276 | 0.471586875325128 | -0.166012576 | 0.898921202692492 |
| CMPK2 | 0.413609901240501 | 0.413609901240501 | -7.491892349 | 14.3147416940381 | 0.702206784591534 | 0.541375911932484 |
| CMTM3 | 1.07590168539481 | 1.07590168539481 | -5.122270147 | 20.6259053891838 | 1.35455277405184 | 0.352314095058106 |
| CMTM6 | -35.95459774 | -34.95459774 | -18.89556551 | 5.57746163964135 | -1.224618435 | 0.366275438800159 |
| CMTR1 | 0.00544772095155079 | 0.00544772095155079 | -0.312571701 | 0.410881201603879 | 0.242363859879721 | 0.82695243198817 |
| CNBP | -0.071695251 | 0.928304749 | -1.073144343 | -0.353763938 | -4.167143692 | 0.0567649960882649 |
| CNDP2 | -0.055998264 | 0.944001736 | -0.929982829 | -0.326119097 | -3.991767105 | 0.0444925432867386 |
| CNFN | 0 | 1 | NA | NA | NA | NA |
| CNIH4 | -0.667189743 | 0.332810257 | -14.52567574 | 7.715161812 | -0.53078944 | 0.613035598848993 |
| CNN1 | 1.03456028001279 | 1.03456028001279 | -5.457549095 | 20.0300108021462 | 1.28195985346042 | 0.366275438800159 |
| CNN2 | 0.0610478149429202 | 0.0610478149429202 | 0.128062891407592 | 1.20823530251363 | 2.46687954341041 | 0.124726261846778 |
| CNN3 | 0.0997897779860161 | 0.0997897779860161 | 0.378820693801914 | 1.83783566803006 | 3.00575615479744 | 0.0846619331307277 |
| CNNM3 | -1.09853594 | -0.09853594 | -17.59748732 | 4.11087161897993 | -1.397582829 | 0.340245200893141 |
| CNOT1 | -0.002359779 | 0.997640221 | -0.276592528 | 0.230943536372822 | -0.156119253 | 0.905956371366286 |
| CNOT10 | 0.0289914705806549 | 0.0289914705806549 | -11.0673832 | 11.4596630003735 | 0.0301325522356483 | 0.998435102357591 |
| CNOT11 | -1.624394667 | -0.624394667 | -16.33820629 | 4.17016330305747 | -1.026778085 | 0.375998813722474 |
| CNOT2 | -0.029554506 | 0.970445494 | -0.469303929 | -0.044853979 | -2.09591469 | 0.119877716277189 |
| CNOT3 | 0.00712283243580717 | 0.00712283243580717 | -0.313231964 | 0.439973344571927 | 0.30554813728999 | 0.771773850480919 |
| CNOT6L | -36.42471699 | -35.42471699 | -19.01318211 | 0.564625473399789 | -2.12053002 | 0.1803694486231 |
| CNOT7 | -0.431855189 | 0.568144811 | -12.82502872 | 6.47369571814878 | -0.739784789 | 0.539497285386707 |
| CNOT9 | -0.012865866 | 0.987134134 | -0.448879498 | 0.217140337821225 | -0.679695369 | 0.539497285386707 |
| CNP | 0.0285406510832836 | 0.0285406510832836 | 0.0500380106043842 | 0.559160120873487 | 2.1504498103094 | 0.126358852687972 |
| CNPY2 | -0.056340267 | 0.943659733 | -0.970506607 | -0.190211554 | -2.633322851 | 0.0779899623978774 |
| CNPY3 | -0.004308467 | 0.995691533 | -0.484293818 | 0.405216625990273 | -0.15544556 | 0.906799670646499 |
| CNPY4 | 0.117908254160694 | 0.117908254160694 | 0.794092530420131 | 1.43354627224549 | 6.24866054175618 | 0.00805555443857398 |
| CNRIP1 | 0.0785028813035253 | 0.0785028813035253 | 0.422415869164898 | 1.06562961172158 | 4.04652444674894 | 0.0260030167431236 |
| CNST | -1.012857558 | -0.012857558 | -12.56834733 | 7.24257465514216 | -0.467858437 | 0.648994611354729 |
| CNTN1 | 36.9821793315989 | 36.9821793315989 | 13.1262837832602 | 14.0240566497975 | 68.0541934106389 | 0.000260229699772871 |
| CNTN4 | 0 | 1 | NA | NA | NA | NA |
| CNTNAP1 | 0.556742877957387 | 0.556742877957387 | -5.405483869 | 14.0813308215381 | 0.996148902454137 | 0.444676083634273 |
| CNTNAP4 | -0.010476385 | 0.989523615 | -0.309126383 | 0.0822498199331101 | -1.00300523 | 0.385148992080337 |
| COA3 | -0.457670603 | 0.542329397 | -13.69896041 | 6.56719686822265 | -0.791654214 | 0.539497285386707 |
| COA4 | -35.60185832 | -34.60185832 | -14.83492742 | 4.4055771755024 | -1.219795093 | 0.366275438800159 |
| COA6 | -36.99529318 | -35.99529318 | -14.16625684 | -13.23200058 | -65.99378089 | 0.000260229699772871 |
| COA7 | -36.43391815 | -35.43391815 | -19.14924235 | 0.582649351037929 | -2.117432532 | 0.1803694486231 |
| COASY | -0.01438744 | 0.98561256 | -0.263259538 | -0.012113302 | -1.905135129 | 0.151564715628381 |
| COBL | 0 | 1 | NA | NA | NA | NA |
| COBLL1 | -35.87761545 | -34.87761545 | -17.91300883 | 5.28693224524955 | -1.224694097 | 0.366275438800159 |
| COCH | 35.9009039186706 | 35.9009039186706 | -5.388020075 | 18.2195649353052 | 1.22313240267177 | 0.366275438800159 |
| COG1 | 0.00312138403661977 | 0.00312138403661977 | -0.707701902 | 0.764906807115128 | 0.0799418206303159 | 0.972765179053006 |
| COG2 | -0.048110447 | 0.951889553 | -0.807596284 | -0.05808605 | -1.998409488 | 0.13308716885642 |
| COG3 | -0.107551078 | 0.892448922 | -11.09735682 | 9.75919705569664 | -0.111121152 | 0.94480414398014 |
| COG4 | -0.049326989 | 0.950673011 | -0.747829943 | -0.130170448 | -2.495994794 | 0.0845612712882046 |
| COG5 | 0.00518793764236503 | 0.00518793764236503 | -0.300217643 | 0.398680792981444 | 0.273407361174986 | 0.803032046011227 |
| COG6 | -0.01206805 | 0.98793195 | -0.606036883 | 0.380834061553696 | -0.463180543 | 0.657423743030449 |
| COG7 | 0.00912275015083697 | 0.00912275015083697 | -0.40411294 | 0.576744153133419 | 0.305687421487607 | 0.769649708445333 |
| COG8 | -0.068107647 | 0.931892353 | -1.119981419 | -0.103421718 | -2.229413813 | 0.126711791969931 |
| COIL | -0.140291746 | 0.859708254 | -1.758974197 | -0.751530962 | -5.15200683 | 0.0386628031379461 |
| COL10A1 | 35.9903363525074 | 35.9903363525074 | -5.717699285 | 19.3698411255516 | 1.22458460070278 | 0.366275438800159 |
| COL11A1 | 0.171209816852792 | 0.171209816852792 | 0.889273399082315 | 3.09392358886509 | 3.51364514020518 | 0.0593825127399959 |
| COL11A2 | 0.100989468762543 | 0.100989468762543 | 0.408363832517064 | 1.58869275529799 | 2.92746170785988 | 0.0548717713947671 |
| COL12A1 | 0.192896200851231 | 0.192896200851231 | 1.44854539224468 | 3.3113152612036 | 4.59221287382876 | 0.0222042334472824 |
| COL14A1 | 0.0952417685408737 | 0.0952417685408737 | 0.484698847297009 | 1.84499040245869 | 3.2739682965764 | 0.0647820440466605 |
| COL15A1 | -0.013254772 | 0.986745228 | -0.605935688 | 0.342255704823069 | -0.500417212 | 0.633597723009551 |
| COL16A1 | 0.1410195408872 | 0.1410195408872 | 0.580250360540526 | 2.383473206 | 3.62546414468256 | 0.0836546650355817 |
| COL17A1 | -35.90452158 | -34.90452158 | -18.25109998 | 5.38733875409245 | -1.224602857 | 0.366275438800159 |
| COL18A1 | 0.0602486851398093 | 0.0602486851398093 | 0.147871601504675 | 1.13995867432889 | 2.25293074614732 | 0.103481070395769 |
| COL1A1 | 0.119812970636615 | 0.119812970636615 | 0.781509260670278 | 2.58247715095199 | 3.825570546 | 0.0612277012636593 |
| COL1A2 | 0.118116942105573 | 0.118116942105573 | 0.789949923216638 | 2.48806060141725 | 3.66636745122502 | 0.0508730095968037 |
| COL21A1 | 36.5865750787613 | 36.5865750787613 | -0.656294015 | 21.2951933584714 | 2.11577103756698 | 0.1803694486231 |
| COL26A1 | 1.18389033657944 | 1.18389033657944 | -3.455789188 | 19.3644829949947 | 1.54670686210237 | 0.291397222441925 |
| COL28A1 | 35.8699324352072 | 35.8699324352072 | -5.292368937 | 17.851384571625 | 1.22114684836708 | 0.366275438800159 |
| COL2A1 | 1.11629733312299 | 1.11629733312299 | -4.809643788 | 21.6054425471539 | 1.4298004744677 | 0.329699705770463 |
| COL3A1 | 0.115287267913044 | 0.115287267913044 | 0.792213013543236 | 2.18962083157616 | 3.9390110666566 | 0.0389117109105226 |
| COL4A1 | 0.0433316203465445 | 0.0433316203465445 | -0.195598425 | 0.911456450261992 | 1.13931921190292 | 0.363549164993598 |
| COL4A2 | 0.0479608406763028 | 0.0479608406763028 | -0.133517295 | 1.13289309199658 | 1.43123401547198 | 0.260738681674259 |
| COL5A1 | 0.135088127750276 | 0.135088127750276 | 0.429389988714586 | 2.70339174980266 | 2.8645263063496 | 0.106098499797615 |
| COL5A2 | 0.150253462898729 | 0.150253462898729 | 0.55412646817303 | 2.57040782682719 | 3.0207301693463 | 0.0811180182352244 |
| COL5A3 | 0.067955975651733 | 0.067955975651733 | -0.215269344 | 1.53731439107817 | 1.35786320686774 | 0.284555793678466 |
| COL6A1 | 0.146988781518096 | 0.146988781518096 | 1.09244787468271 | 2.54121804907731 | 4.64769185295376 | 0.0270870760891318 |
| COL6A2 | 0.153197839859792 | 0.153197839859792 | 1.17116606238965 | 2.58757308243709 | 4.90198310159893 | 0.0229629824313062 |
| COL6A3 | 0.146942505436706 | 0.146942505436706 | 1.11544855347377 | 2.60732388451355 | 4.42729252249791 | 0.0222042334472824 |
| COL6A5 | 0 | 1 | NA | NA | NA | NA |
| COL6A6 | -35.01158787 | -34.01158787 | -14.48666841 | 7.55930229296355 | -0.707106781 | 0.539497285386707 |
| COL7A1 | 0.49862557241091 | 0.49862557241091 | -6.288362682 | 14.3266236538571 | 0.874251572022358 | 0.506106824045559 |
| COL8A1 | 0.628422101212792 | 0.628422101212792 | -5.143811514 | 16.1544642567548 | 1.15203645624122 | 0.379841861092033 |
| COL8A2 | 1.09661049096432 | 1.09661049096432 | -4.680081373 | 19.9886404119509 | 1.39206575224742 | 0.3412183162817 |
| COLEC12 | 0.448067928919352 | 0.448067928919352 | -7.363969008 | 15.0415963495998 | 0.768491801612244 | 0.539497285386707 |
| COLGALT1 | 0.0419311824325996 | 0.0419311824325996 | 0.0209222140890567 | 0.868925844106337 | 1.90785321481549 | 0.167509566752535 |
| COLGALT2 | 0.0396990413116874 | 0.0396990413116874 | -0.043695053 | 0.760201511532781 | 1.54510410274542 | 0.205852646469262 |
| COMMD1 | -36.65880065 | -35.65880065 | -22.36193165 | 0.663487456470606 | -2.120640926 | 0.1803694486231 |
| COMMD10 | 0.0169940473629509 | 0.0169940473629509 | -0.471603019 | 0.778369607305561 | 0.465534492410351 | 0.654211894695707 |
| COMMD2 | 0.00685763376876745 | 0.00685763376876745 | -0.176914675 | 0.313114037988171 | 0.518968657406223 | 0.625689848851569 |
| COMMD3 | 0.0524125728610509 | 0.0524125728610509 | -0.529538478 | 1.44070180559886 | 0.908834431474633 | 0.464844165288807 |
| COMMD4 | -0.434328313 | 0.565671687 | -13.50687721 | 6.76976006535946 | -0.743145589 | 0.539497285386707 |
| COMMD5 | 0.0120006950215603 | 0.0120006950215603 | -0.318037048 | 0.536739481553867 | 0.51236784253845 | 0.634031727574291 |
| COMMD6 | 0.00221425778963713 | 0.00221425778963713 | -1.088303184 | 1.12734153331222 | 0.036801001429055 | 0.997044355619252 |
| COMMD7 | -0.558608401 | 0.441391599 | -16.0789824 | 9.57808899586214 | -0.441159527 | 0.66029657356452 |
| COMMD8 | -0.557573446 | 0.442426554 | -14.10430925 | 8.41102177544345 | -0.440284005 | 0.660811863371745 |
| COMMD9 | -0.004851141 | 0.995148859 | -0.601800199 | 0.515213222867922 | -0.153034648 | 0.911663955067371 |
| COMP | 0.497935544990109 | 0.497935544990109 | -6.382747099 | 14.5143124715733 | 0.871921984941973 | 0.507329132034982 |
| COMT | 0.0171474200912519 | 0.0171474200912519 | -0.591843214 | 0.925113225453653 | 0.487170370577544 | 0.652187811666889 |
| COMTD1 | -1.104711996 | -0.104711996 | -19.20337456 | 4.40845516281322 | -1.407623726 | 0.336395164305236 |
| COPA | -0.016245325 | 0.983754675 | -0.621054161 | 0.259379011772477 | -0.736750377 | 0.539497285386707 |
| COPB1 | -0.014138949 | 0.985861051 | -0.631736632 | 0.311739686857299 | -0.618383027 | 0.562186501283842 |
| COPB2 | -0.008967855 | 0.991032145 | -0.682546217 | 0.479387278432386 | -0.352494027 | 0.738663043597405 |
| COPE | 0.000343846357505722 | 0.000343846357505722 | -0.669966256 | 0.677385875386681 | 0.0123905053755938 | 0.999130434024431 |
| COPG1 | -0.015802775 | 0.984197225 | -0.588831374 | 0.239830324991642 | -0.788027946 | 0.523139705644887 |
| COPG2 | 0.0180734676687985 | 0.0180734676687985 | -0.21261312 | 0.561192362634388 | 0.944456632504813 | 0.458975962378967 |
| COPS2 | 0.0261254628670295 | 0.0261254628670295 | -0.073349862 | 0.588350864580062 | 1.52369888587546 | 0.257267458304079 |
| COPS3 | 0.00913079335250497 | 0.00913079335250497 | -0.055881769 | 0.232980949089269 | 1.08078672689014 | 0.366275438800159 |
| COPS4 | 0.0169558994809473 | 0.0169558994809473 | 0.00414955966692807 | 0.332389968894734 | 2.13631965572718 | 0.174665065873632 |
| COPS5 | 0.0291775384732838 | 0.0291775384732838 | -0.057017257 | 0.611420184813386 | 1.49067992850767 | 0.235998473152946 |
| COPS6 | 0.0363437336169569 | 0.0363437336169569 | -0.081737507 | 0.792631383251319 | 1.71635620076666 | 0.229599963554651 |
| COPS7A | 0.0206052194432216 | 0.0206052194432216 | -0.164266092 | 0.563286387310033 | 1.04737292818302 | 0.387543403611319 |
| COPS7B | -0.306477126 | 0.693522874 | -12.57421827 | 7.98749110414982 | -0.500382615 | 0.647379845419524 |
| COPS8 | 0.00432400990185448 | 0.00432400990185448 | -0.184669183 | 0.266355294894907 | 0.31849476347102 | 0.759152568141338 |
| COPZ1 | -0.051046169 | 0.948953831 | -0.917280693 | -0.123719349 | -2.28285399 | 0.100975155016721 |
| COPZ2 | 0.101828561813009 | 0.101828561813009 | 0.367556648956634 | 1.6435247544041 | 3.40944946842366 | 0.0864895828009813 |
| COQ10A | -0.571872569 | 0.428127431 | -16.14471063 | 9.48531372411009 | -0.452076773 | 0.655753631330814 |
| COQ10B | 0 | 1 | NA | NA | NA | NA |
| COQ3 | -34.71067139 | -33.71067139 | -11.75935853 | 6.13616211710915 | -0.707106781 | 0.539497285386707 |
| COQ5 | -2.022685019 | -1.022685019 | -19.98609489 | 0.370726359629338 | -2.164380907 | 0.1803694486231 |
| COQ6 | -0.031456721 | 0.968543279 | -1.138370233 | 0.57979541844744 | -0.575333521 | 0.585410762254481 |
| COQ7 | -0.445137346 | 0.554862654 | -13.41115306 | 6.59502299963092 | -0.765416054 | 0.539497285386707 |
| COQ8A | -0.049283055 | 0.950716945 | -0.777022254 | -0.088934555 | -2.389614747 | 0.117528060177276 |
| COQ9 | -0.454997164 | 0.545002836 | -14.02713038 | 6.75841836391786 | -0.785873159 | 0.539497285386707 |
| CORO1A | -0.106088193 | 0.893911807 | -1.820798 | -0.528674163 | -3.781558034 | 0.0660749195938009 |
| CORO1B | 0.00453353915831832 | 0.00453353915831832 | -0.245432108 | 0.339932555701364 | 0.323490020017394 | 0.761561174944822 |
| CORO1C | 0.0500761420494989 | 0.0500761420494989 | 0.324490212967742 | 0.696099767208523 | 4.96697787626972 | 0.0187515300021514 |
| CORO2A | -1.120258544 | -0.120258544 | -14.56069636 | 7.92138666656481 | -0.517212154 | 0.621704967022904 |
| CORO2B | 0.0611213151401153 | 0.0611213151401153 | -0.044381265 | 1.19989765290247 | 1.63871033691835 | 0.187211943197938 |
| CORO6 | 0 | 1 | NA | NA | NA | NA |
| CORO7 | -0.059374295 | 0.940625705 | -0.983573476 | -0.179277923 | -2.550507186 | 0.082420509168267 |
| COTL1 | -0.029261565 | 0.970738435 | -0.734516403 | 0.0874664437032822 | -1.383418448 | 0.265815462779191 |
| COX11 | -34.77241959 | -33.77241959 | -12.27359232 | 6.40449494360097 | -0.707106781 | 0.539497285386707 |
| COX14 | 0 | 1 | NA | NA | NA | NA |
| COX17 | -0.541772109 | 0.458227891 | -14.46205556 | 5.77042854944901 | -0.965667081 | 0.461642070669539 |
| COX19 | -1.024625787 | -0.024625787 | -17.75107636 | 4.95148801404158 | -1.265300932 | 0.366275438800159 |
| COX20 | -0.392145283 | 0.607854717 | -12.4155954 | 6.74203958218243 | -0.659423089 | 0.560961264806121 |
| COX4I1 | -0.049974503 | 0.950025497 | -0.79483892 | -0.269147292 | -3.505181116 | 0.0357653827046287 |
| COX5A | -0.044356799 | 0.955643201 | -0.724452175 | -0.265678967 | -4.579155771 | 0.0515046474290599 |
| COX5B | -0.041927376 | 0.958072624 | -0.79642375 | -0.078034058 | -2.613510164 | 0.131425840811869 |
| COX6B1 | -0.040796685 | 0.959203315 | -0.794365212 | -0.061098449 | -2.027504265 | 0.130438701726166 |
| COX6C | -0.038152687 | 0.961847313 | -0.665733247 | -0.19001165 | -3.344613238 | 0.0553885422324435 |
| COX7A1 | 0.0668624230545071 | 0.0668624230545071 | 0.229657519507516 | 0.978607956857644 | 2.90139742191261 | 0.0665100994548111 |
| COX7A2 | -0.073167521 | 0.926832479 | -1.262971953 | -0.321273099 | -3.156689944 | 0.0648842992292444 |
| COX7A2L | -2.033133789 | -1.033133789 | -21.1479255 | 0.279013766450192 | -2.183282397 | 0.1803694486231 |
| COX7B | -36.91934355 | -35.91934355 | -13.43935304 | -12.55385155 | -66.05666106 | 0.000260229699772871 |
| COX7C | -0.526562383 | 0.473437617 | -14.39411227 | 5.94401364963171 | -0.933886219 | 0.477095057556613 |
| CP | 0.0635132895455931 | 0.0635132895455931 | 0.35232859784249 | 1.02647134937011 | 3.53916230749902 | 0.0350150008153323 |
| CPA3 | -0.060245695 | 0.939754305 | -2.883085757 | 1.58546755617339 | -0.52970845 | 0.618050310462089 |
| CPA4 | 0 | 1 | NA | NA | NA | NA |
| CPB2 | 0.098016262110881 | 0.098016262110881 | 0.618725963015576 | 1.23159660653749 | 5.24749038490679 | 0.0102101330381638 |
| CPD | -36.39272207 | -35.39272207 | -18.59431689 | 0.55039404600155 | -2.120939279 | 0.1803694486231 |
| CPM | -35.87100121 | -34.87100121 | -17.94048648 | 5.37216343764161 | -1.213198263 | 0.366735091564231 |
| CPN1 | 0.0681106230353627 | 0.0681106230353627 | 0.0902051972348 | 1.17655200209992 | 2.14365381684782 | 0.134033116286307 |
| CPN2 | 1.05973219503331 | 1.05973219503331 | -5.066228831 | 19.743886454764 | 1.32586079298991 | 0.35880858304931 |
| CPNE1 | 0.0465453412603975 | 0.0465453412603975 | 0.278997759460583 | 0.708694580982357 | 4.03828314510769 | 0.0270693975625301 |
| CPNE2 | 0.0679760788647091 | 0.0679760788647091 | 0.116335965430236 | 1.16006184435358 | 2.44955809560368 | 0.127493113256436 |
| CPNE3 | -0.004389815 | 0.995610185 | -0.231895891 | 0.133707702800231 | -0.489803214 | 0.640292274015903 |
| CPNE6 | 0 | 1 | NA | NA | NA | NA |
| CPOX | -0.005519261 | 0.994480739 | -0.411278311 | 0.299269437824707 | -0.341430313 | 0.752130837242416 |
| CPPED1 | 0.0195691216184612 | 0.0195691216184612 | -0.27423313 | 0.647618254449546 | 0.822741822160197 | 0.517642586759047 |
| CPQ | 0.102364361326701 | 0.102364361326701 | 0.486674735124447 | 1.50155557708129 | 3.54761011578567 | 0.0437718992653409 |
| CPS1 | 36.8378039711937 | 36.8378039711937 | 11.8985692400614 | 12.666272209186 | 72.0056926174423 | 0.000254048186324466 |
| CPSF1 | -0.049604573 | 0.950395427 | -0.765407473 | -0.169898566 | -3.229458472 | 0.0839280070110771 |
| CPSF2 | -0.080933487 | 0.919066513 | -1.143865009 | -0.260288337 | -3.239014696 | 0.0813439485664402 |
| CPSF3 | -0.058860297 | 0.941139703 | -0.779983674 | -0.321775641 | -4.280980282 | 0.0255994622577781 |
| CPSF4 | -36.77461691 | -35.77461691 | -12.93709953 | -10.57507323 | -22.40031477 | 0.00306401850664402 |
| CPSF6 | -0.021721728 | 0.978278272 | -0.488578028 | 0.0452914527306367 | -1.437070353 | 0.239662739711551 |
| CPSF7 | -0.027368243 | 0.972631757 | -0.592764323 | 0.0534786738338976 | -1.508990639 | 0.232207825901483 |
| CPT1A | -0.02954763 | 0.97045237 | -0.529669893 | -0.042255655 | -2.106206636 | 0.130705233401285 |
| CPT1B | 0 | 1 | NA | NA | NA | NA |
| CPT2 | -0.073827889 | 0.926172111 | -1.121568195 | -0.297917846 | -3.465458888 | 0.0695970603154579 |
| CPVL | -0.045468595 | 0.954531405 | -1.424234999 | 0.511003680091646 | -0.938740227 | 0.450866419056598 |
| CPXM1 | 0.103855400541049 | 0.103855400541049 | 0.396199419985045 | 1.64441828085827 | 2.89132698520143 | 0.0619159459285198 |
| CPXM2 | 0.498761004455163 | 0.498761004455163 | -6.651427422 | 15.144202304449 | 0.874890441507961 | 0.506088775093368 |
| CPZ | -0.120825727 | 0.879174273 | -10.98345636 | 10.4659709374307 | -0.041813945 | 0.994613821823136 |
| CR1 | -36.38940423 | -35.38940423 | -18.6423987 | 0.639924634455472 | -2.100965496 | 0.180716766229804 |
| CRABP1 | 0.0817321510012841 | 0.0817321510012841 | -1.086458153 | 2.62518657047839 | 0.788358888528233 | 0.524475843328539 |
| CRABP2 | 0.0904655113386666 | 0.0904655113386666 | -0.124026732 | 1.85617797423285 | 1.64098183394032 | 0.208700918814415 |
| CRADD | -0.640159193 | 0.359840807 | -13.88363381 | 7.60517520299785 | -0.506902875 | 0.627348453175941 |
| CRAT | -0.00275718 | 0.99724282 | -0.385709797 | 0.333131791402853 | -0.129387049 | 0.929355021297957 |
| CREB1 | -0.038035816 | 0.961964184 | -0.726803938 | 0.0279510993380834 | -1.679963835 | 0.187211943197938 |
| CREBBP | 0.0833789691715752 | 0.0833789691715752 | -9.033642719 | 9.34004502931542 | 0.028876860724795 | 0.998435102357591 |
| CREG1 | -0.608912437 | 0.391087563 | -14.36691502 | 8.13216990090446 | -0.481154366 | 0.642062788922739 |
| CRELD1 | 0.0633443860137981 | 0.0633443860137981 | -0.445085563 | 1.62085688623245 | 1.14701156699345 | 0.366735091564231 |
| CRELD2 | -0.119621461 | 0.880378539 | -2.003821541 | -0.32091714 | -2.788479468 | 0.103481070395769 |
| CRIP1 | 0.019567948650652 | 0.019567948650652 | -0.472419453 | 0.927182535684849 | 0.612875820026722 | 0.568897620936761 |
| CRIP2 | 0.0522542946114139 | 0.0522542946114139 | 0.111052628753859 | 0.926912625911145 | 2.20793305898409 | 0.108199037689272 |
| CRISP3 | -2.153279944 | -1.153279944 | -18.85204825 | -0.801656767 | -2.428800337 | 0.161680819566986 |
| CRISPLD2 | 1.08709298958207 | 1.08709298958207 | -4.312388251 | 17.9521458968907 | 1.37572828044454 | 0.345464653630088 |
| CRK | 0.0253888378038095 | 0.0253888378038095 | -0.021477897 | 0.517168824215429 | 1.64540775603444 | 0.189983954626358 |
| CRKL | -0.004687621 | 0.995312379 | -0.304514263 | 0.212360606294602 | -0.363682784 | 0.730113530415138 |
| CRLF3 | -0.446447179 | 0.553552821 | -14.01625261 | 6.87447731659548 | -0.769139171 | 0.539497285386707 |
| CRMP1 | 0.500356914020712 | 0.500356914020712 | -5.942064609 | 13.7153338584658 | 0.868137115729333 | 0.506088775093368 |
| CRNKL1 | -0.440371825 | 0.559628175 | -13.32922315 | 6.623604618 | -0.755557339 | 0.539497285386707 |
| CRNN | 1.1889358471118 | 1.1889358471118 | -8.951424058 | 16.8686940312248 | 0.541155668633879 | 0.608391382020405 |
| CROCC | 0.974361269028546 | 0.974361269028546 | -6.060447013 | 19.5600332581283 | 1.17808468906732 | 0.37350198937203 |
| CROT | -0.01758586 | 0.98241414 | -0.492762173 | 0.1889775748618 | -0.771042474 | 0.520251905198183 |
| CRP | -34.79570059 | -33.79570059 | -12.4732599 | 6.50868367553937 | -0.707106781 | 0.539497285386707 |
| CRTAC1 | 0.219417332045034 | 0.219417332045034 | -9.972754798 | 12.9569418060045 | 0.226386178412045 | 0.840669963706941 |
| CRTAP | 0.0954143696810689 | 0.0954143696810689 | 0.377760282032332 | 1.65311744761547 | 3.29618653060547 | 0.082420509168267 |
| CRTC1 | 0.0471957949684316 | 0.0471957949684316 | 0.0684586982293528 | 0.791120732301711 | 2.58277962070795 | 0.138119690893487 |
| CRTC2 | -34.7038874 | -33.7038874 | -11.70419225 | 6.10737574982662 | -0.707106781 | 0.539497285386707 |
| CRYAB | 0.116819028262736 | 0.116819028262736 | 0.37507842014949 | 1.8865440338608 | 2.95144105802062 | 0.0864895828009813 |
| CRYBG1 | 0.0478186610349725 | 0.0478186610349725 | -10.94422839 | 11.5910031875681 | 0.0496716626067452 | 0.991496031344742 |
| CRYBG2 | 0 | 1 | NA | NA | NA | NA |
| CRYL1 | -0.001307761 | 0.998692239 | -0.223997653 | 0.198414735871588 | -0.108591002 | 0.947016998104297 |
| CRYZ | 0.0469001850169588 | 0.0469001850169588 | 0.182882000421062 | 0.866049260161849 | 2.65688733818499 | 0.0698765466924951 |
| CRYZL1 | -0.014638747 | 0.985361253 | -11.83540659 | 11.6291076660695 | -0.015212423 | 0.999130434024431 |
| CS | -0.062157037 | 0.937842963 | -0.998252904 | -0.369238167 | -4.531936097 | 0.0493141768719727 |
| CSDE1 | -0.032639251 | 0.967360749 | -0.471112635 | -0.176820046 | -4.236364212 | 0.0404784941376624 |
| CSE1L | 0.00280972187963155 | 0.00280972187963155 | -0.09672521 | 0.153768500705627 | 0.407156972925942 | 0.68507047821426 |
| CSF1R | 0.0337274145257241 | 0.0337274145257241 | -0.315582406 | 0.89254768393024 | 0.968683056285739 | 0.437871424197525 |
| CSGALNACT2 | 1.62043061774278 | 1.62043061774278 | -4.338738764 | 16.9220376220641 | 1.02420139010187 | 0.377033418126296 |
| CSK | -0.041798342 | 0.958201658 | -0.642719648 | -0.203815922 | -3.337499458 | 0.0402744596888282 |
| CSNK1A1 | 0.046398485702716 | 0.046398485702716 | -0.28883594 | 1.16348771013081 | 1.24239917307016 | 0.363817806593495 |
| CSNK1G2 | 1.56061195392908 | 1.56061195392908 | -4.526492639 | 16.4314947509378 | 0.982905801084614 | 0.393933863628188 |
| CSNK2A1 | 0.0484417856761243 | 0.0484417856761243 | -0.16149131 | 0.99161695257588 | 1.34777055612108 | 0.302238855639596 |
| CSNK2A2 | -0.000136957 | 0.999863043 | -0.378259896 | 0.375744100730104 | -0.006783387 | 0.999130434024431 |
| CSNK2B | 0.0293765724205994 | 0.0293765724205994 | -0.139502176 | 0.70506104359903 | 1.16533167066789 | 0.352569768252778 |
| CSPG4 | 0.535457873507806 | 0.535457873507806 | -6.033008363 | 14.9011352862181 | 0.951395059527454 | 0.468322577380277 |
| CSRP1 | 0.0756110307556676 | 0.0756110307556676 | 0.430778252710484 | 1.12956365814047 | 4.24301096134899 | 0.0383041704369688 |
| CSRP2 | 0.114922099431067 | 0.114922099431067 | 0.497501924108857 | 1.89382169516123 | 2.96504727932531 | 0.0537719121043574 |
| CSRP3 | 0 | 1 | NA | NA | NA | NA |
| CST2 | 0 | 1 | NA | NA | NA | NA |
| CST3 | 0.0396112018396058 | 0.0396112018396058 | 0.0859119359950983 | 0.674272893896825 | 2.28207427029541 | 0.106098499797615 |
| CST6 | -34.86381487 | -33.86381487 | -13.07628609 | 6.82334934713934 | -0.707106781 | 0.539497285386707 |
| CSTA | -0.469768348 | 0.530231652 | -13.9529921 | 6.51877320422814 | -0.815759241 | 0.532353730394718 |
| CSTB | -0.027090207 | 0.972909793 | -0.845779183 | 0.270221385627049 | -1.03107252 | 0.403567666451163 |
| CSTF1 | -0.026869534 | 0.973130466 | -0.448965877 | -0.036241616 | -2.120081867 | 0.130567854759618 |
| CSTF2 | -0.0570888 | 0.9429112 | -0.991308979 | -0.115621125 | -2.263703325 | 0.112158244508424 |
| CSTF2T | -2.04019167 | -1.04019167 | -18.58234664 | 0.189348426559074 | -2.198268503 | 0.1803694486231 |
| CSTF3 | -0.021438105 | 0.978561895 | -0.469504511 | 0.0548014609520155 | -1.387957478 | 0.263974040065859 |
| CTBP1 | -0.007931149 | 0.992068851 | -0.376891259 | 0.219403079004688 | -0.457252356 | 0.653774096383628 |
| CTBP2 | 0.067096533305027 | 0.067096533305027 | -0.614243919 | 1.89201273672557 | 0.886061341634006 | 0.451108497783615 |
| CTBS | -1.055463967 | -0.055463967 | -20.79817249 | 5.41382496678985 | -1.320650494 | 0.360282869162183 |
| CTCF | 0.568748630318555 | 0.568748630318555 | -9.047720758 | 15.3460276662651 | 0.449262640714054 | 0.656682440358441 |
| CTDNEP1 | -0.362447055 | 0.637552945 | -13.73861473 | 7.91064783720196 | -0.605357678 | 0.589740665249948 |
| CTDP1 | -0.499395122 | 0.500604878 | -13.50580788 | 5.92680340403968 | -0.876438918 | 0.50575395903854 |
| CTDSP1 | 0.00121384718533586 | 0.00121384718533586 | -0.354451701 | 0.375630117776097 | 0.0517769809364767 | 0.990756959288016 |
| CTDSPL2 | -0.951257102 | 0.048742898 | -16.89151757 | 5.50610835237364 | -1.142249479 | 0.383831171138254 |
| CTH | -36.95841379 | -35.95841379 | -13.44779133 | -13.25896472 | -318.2760916 | 0.000135517346157358 |
| CTHRC1 | 0.00487031114185223 | 0.00487031114185223 | -0.909653586 | 1.01096873189818 | 0.0917772217796049 | 0.9623155826899 |
| CTNNA1 | 0.072618012168401 | 0.072618012168401 | 0.285006647261425 | 1.18981069618308 | 3.03803929051624 | 0.0693365999576725 |
| CTNNA2 | -35.85585177 | -34.85585177 | -17.64824775 | 5.21121154867467 | -1.224325886 | 0.366275438800159 |
| CTNNB1 | 0.0957762169312285 | 0.0957762169312285 | 0.314931913445523 | 1.59992147061944 | 2.72968400279817 | 0.0811824560348127 |
| CTNNBIP1 | -0.437730587 | 0.562269413 | -14.26888788 | 9.61484650789239 | -0.34195323 | 0.739477926908747 |
| CTNNBL1 | -0.005875549 | 0.994124451 | -0.456445972 | 0.347452684189687 | -0.253549258 | 0.818866023169525 |
| CTNND1 | 0.0625530232671602 | 0.0625530232671602 | 0.29159812342199 | 0.962897845420906 | 3.37099108872134 | 0.0489433019350404 |
| CTPS1 | 0.0646827898502186 | 0.0646827898502186 | 0.32225939174219 | 0.896913359640212 | 3.73718088578889 | 0.033716702615266 |
| CTPS2 | 0.47278810744695 | 0.47278810744695 | -6.644044815 | 14.2993390012415 | 0.822324112853545 | 0.530364745744375 |
| CTR9 | -36.8967101 | -35.8967101 | -13.24961444 | -12.3389827 | -63.23387256 | 0.000273271710161686 |
| CTSA | -0.049490079 | 0.950509921 | -0.85707636 | -0.117206514 | -2.290784854 | 0.100720852088896 |
| CTSB | -0.144590239 | 0.855409761 | -2.662135005 | -0.637573633 | -3.240990032 | 0.0757988675671794 |
| CTSC | -0.089974871 | 0.910025129 | -1.450586014 | -0.34494257 | -3.024156888 | 0.0695970603154579 |
| CTSD | -0.087199244 | 0.912800756 | -2.1089248 | 0.0982572233542119 | -1.901367429 | 0.187940717985324 |
| CTSG | -0.103030784 | 0.896969216 | -3.27061219 | 0.780560422382686 | -1.22754525 | 0.362428697203791 |
| CTSH | -0.06222738 | 0.93777262 | -1.210704732 | -0.020672627 | -1.998284862 | 0.171637099443428 |
| CTSK | 0.0518918592101848 | 0.0518918592101848 | -0.241095831 | 1.25855999053462 | 1.24730004276922 | 0.336370257762061 |
| CTSL | -1.069849322 | -0.069849322 | -19.26697522 | 4.8389126487894 | -1.344765179 | 0.354738855177865 |
| CTSS | -0.134946182 | 0.865053818 | -2.046179479 | -0.740901867 | -3.914587457 | 0.0381981169492808 |
| CTSV | 0 | 1 | NA | NA | NA | NA |
| CTSZ | -0.092248423 | 0.907751577 | -1.940439875 | -0.103226108 | -2.088271806 | 0.149920297231595 |
| CTTN | 0.0402325391227301 | 0.0402325391227301 | 0.0406609653968598 | 0.778262153413032 | 2.46128998950454 | 0.157117829321625 |
| CTTNBP2NL | -1.026748663 | -0.026748663 | -15.11182122 | 8.63603051098512 | -0.474834028 | 0.645841531946401 |
| CTU1 | -34.91797658 | -33.91797658 | -13.57652781 | 7.08438095434586 | -0.707106781 | 0.539497285386707 |
| CUL1 | 0.00827204802392919 | 0.00827204802392919 | -0.307275846 | 0.46018293597257 | 0.351595474473238 | 0.731308459489582 |
| CUL2 | 0.0336817063068118 | 0.0336817063068118 | -0.15695741 | 0.806693943072341 | 1.38887515697652 | 0.31778775238987 |
| CUL3 | 0.02883761077419 | 0.02883761077419 | -0.03425952 | 0.578150886550541 | 1.54373007576158 | 0.20725263393776 |
| CUL4A | 0.0563865314722735 | 0.0563865314722735 | -0.29188749 | 1.29624852883782 | 1.37832609020372 | 0.337769090879761 |
| CUL4B | 0.0124591783279063 | 0.0124591783279063 | -0.167671587 | 0.395757505452099 | 0.700919368659512 | 0.539497285386707 |
| CUL5 | 0.0326858768508971 | 0.0326858768508971 | 0.034699780891888 | 0.592304491450701 | 1.97551485455773 | 0.142813069729428 |
| CUL7 | 36.8605002213707 | 36.8605002213707 | 11.826442725288 | 13.1279047140431 | 43.1480145160731 | 0.000583895136039144 |
| CUTA | -0.041750261 | 0.958249739 | -0.619789914 | -0.222510902 | -3.6874586 | 0.032294204946929 |
| CUTC | -0.043010308 | 0.956989692 | -0.641146052 | -0.181323242 | -3.105898893 | 0.0489656305565222 |
| CUX1 | -0.442149518 | 0.557850482 | -13.52925602 | 6.687998026 | -0.7601703 | 0.539497285386707 |
| CWC15 | -0.408218725 | 0.591781275 | -14.2134169 | 7.51974758927556 | -0.692389974 | 0.546638926108471 |
| CWC22 | -0.391060603 | 0.608939397 | -12.73567397 | 6.9541270707588 | -0.660003159 | 0.561015630932865 |
| CWC27 | -1.625618931 | -0.625618931 | -16.12400517 | 4.10608179029207 | -1.028060698 | 0.375653536348272 |
| CWF19L1 | -0.950630978 | 0.049369022 | -17.37643367 | 5.6408766207051 | -1.132977364 | 0.384764475182212 |
| CXADR | 0 | 1 | NA | NA | NA | NA |
| CXCL1 | -34.99604626 | -33.99604626 | -14.33144656 | 7.47830583172277 | -0.707106781 | 0.539497285386707 |
| CXCL10 | 0 | 1 | NA | NA | NA | NA |
| CXCL12 | 0.0173311482640565 | 0.0173311482640565 | -0.417748236 | 0.718634580060696 | 0.469431448676379 | 0.649304231210266 |
| CXCL13 | -1.035186585 | -0.035186585 | -18.83296583 | 5.12586176796964 | -1.283274793 | 0.366275438800159 |
| CXCL14 | 1.89727372450306 | 1.89727372450306 | -3.419654484 | 18.7033855233927 | 1.20862142194458 | 0.336681562276805 |
| CXCL17 | 0 | 1 | NA | NA | NA | NA |
| CXCL6 | 0 | 1 | NA | NA | NA | NA |
| CXorf38 | -0.012617542 | 0.987382458 | -0.367324001 | 0.133524692585954 | -0.820948332 | 0.493474404826194 |
| CYB561D2 | -35.78350136 | -34.78350136 | -16.78178901 | 4.95308252102477 | -1.224690264 | 0.366275438800159 |
| CYB5A | 0.0200442444492489 | 0.0200442444492489 | -0.149924471 | 0.56233423454247 | 1.1309921316339 | 0.366735091564231 |
| CYB5B | 0.00446542157717475 | 0.00446542157717475 | -0.553730157 | 0.643291264897386 | 0.130375864462201 | 0.928328518499981 |
| CYB5R1 | -0.022787117 | 0.977212883 | -0.492347781 | 0.0184918143988102 | -1.630444173 | 0.187539194882395 |
| CYB5R2 | 0.44176457634552 | 0.44176457634552 | -7.152807636 | 14.4486716042721 | 0.759836308324574 | 0.539497285386707 |
| CYB5R3 | 0.0637002445420347 | 0.0637002445420347 | 0.241827523780016 | 1.17335610238433 | 2.63196565522359 | 0.072020993150171 |
| CYBA | -1.08783727 | -0.08783727 | -20.08976406 | 4.82009779519149 | -1.377404316 | 0.345139853904083 |
| CYBB | -0.114504161 | 0.885495839 | -1.488047796 | -0.701946112 | -5.154140939 | 0.0202513372867071 |
| CYBC1 | -0.094876498 | 0.905123502 | -1.240039474 | -0.617693359 | -5.172597064 | 0.0102762020199873 |
| CYBRD1 | 0.0760128777123528 | 0.0760128777123528 | 0.160204988660405 | 1.45603292600307 | 2.33153186081593 | 0.118381386844144 |
| CYC1 | -0.044250652 | 0.955749348 | -0.725608642 | -0.18075883 | -3.152407357 | 0.0680915154796618 |
| CYCS | -0.073942243 | 0.926057757 | -1.285337136 | -0.267597483 | -3.242401643 | 0.0896916084940673 |
| CYFIP1 | 0.0207149787055892 | 0.0207149787055892 | -0.031127048 | 0.446621694448453 | 1.71223094246198 | 0.207918045420449 |
| CYFIP2 | -0.404212083 | 0.595787917 | -13.12158124 | 6.98987331369701 | -0.685165389 | 0.550025408354715 |
| CYGB | -0.476543391 | 0.523456609 | -13.6066798 | 6.25320719489143 | -0.82772323 | 0.52730639626287 |
| CYLD | -0.594968357 | 0.405031643 | -15.17012359 | 8.70056481310691 | -0.471039575 | 0.647660636368325 |
| CYP1B1 | -0.519642209 | 0.480357791 | -15.98345096 | 9.91587369167687 | -0.408757213 | 0.682359803325453 |
| CYP20A1 | -0.046966319 | 0.953033681 | -0.918037508 | 0.0112024555389361 | -1.897853775 | 0.1803694486231 |
| CYP27A1 | -0.045874401 | 0.954125599 | -1.839931725 | 0.952138521379255 | -0.65787362 | 0.555383755208474 |
| CYP2S1 | -0.03046933 | 0.96953067 | -0.90558695 | 0.383487982613089 | -0.704455827 | 0.539497285386707 |
| CYP4F11 | 0 | 1 | NA | NA | NA | NA |
| CYP4F22 | 0 | 1 | NA | NA | NA | NA |
| CYP4F3 | -34.84621914 | -33.84621914 | -12.91777066 | 6.74063425610473 | -0.707106781 | 0.539497285386707 |
| CYP4X1 | -34.96420643 | -33.96420643 | -14.01862066 | 7.31506984935096 | -0.707106781 | 0.539497285386707 |
| CYP51A1 | -0.426766338 | 0.573233662 | -13.49191896 | 6.87807443735505 | -0.729540743 | 0.539497285386707 |
| CYP7B1 | -0.029552859 | 0.970447141 | -0.856937868 | 0.30362279328074 | -0.986912249 | 0.432839309433579 |
| CYRIA | -0.483761827 | 0.516238173 | -13.70305559 | 6.22129798691962 | -0.844515919 | 0.520938784373714 |
| CYRIB | -0.057771831 | 0.942228169 | -0.997591203 | -0.108041528 | -2.505827069 | 0.124570854411906 |
| CZIB | 0.0541717218446013 | 0.0541717218446013 | 0.155363595815758 | 0.916175623374641 | 2.43880587878521 | 0.0846619331307277 |
| D2HGDH | 0.00905814771314956 | 0.00905814771314956 | -10.80278325 | 10.9209688687062 | 0.00941318242543668 | 0.999130434024431 |
| DAB2 | 0.0137579235720126 | 0.0137579235720126 | -0.171803403 | 0.442741877148603 | 0.868812477791634 | 0.488463954357916 |
| DAB2IP | 0 | 1 | NA | NA | NA | NA |
| DAD1 | -0.06655939 | 0.93344061 | -1.022249595 | -0.497153907 | -5.642443992 | 0.0236568001512545 |
| DAG1 | 0.0910126206453671 | 0.0910126206453671 | 0.38244665445208 | 1.42756277790996 | 3.47793734156327 | 0.0688482766944037 |
| DAGLB | 0.518982451329412 | 0.518982451329412 | -9.297188791 | 14.9786808012983 | 0.408401501619272 | 0.682516504408689 |
| DAP | -0.375849548 | 0.624150452 | -13.34419668 | 7.48285938948656 | -0.629622932 | 0.575409334330215 |
| DAP3 | -0.105014588 | 0.894985412 | -1.641203184 | -0.313825513 | -3.138824734 | 0.0961074340736676 |
| DAPK3 | 0.507223547959654 | 0.507223547959654 | -6.030271008 | 13.9499410848874 | 0.891102168965409 | 0.497964265646538 |
| DARS1 | -0.029691435 | 0.970308565 | -0.702773087 | 0.073126695666195 | -1.74650282 | 0.228864939907129 |
| DARS2 | -0.061233374 | 0.938766626 | -0.871758539 | -0.293885167 | -3.646585219 | 0.0407587993982122 |
| DAZAP1 | -0.024217153 | 0.975782847 | -0.604298015 | 0.125541533910831 | -1.144139991 | 0.360245704961941 |
| DBI | 0.0177076708378627 | 0.0177076708378627 | -0.109996267 | 0.460517976515976 | 1.06463611 | 0.366275438800159 |
| DBN1 | 0.0864005498794463 | 0.0864005498794463 | 0.450907840138881 | 1.37734446714981 | 4.00971881631485 | 0.0548717713947671 |
| DBNL | -0.011033471 | 0.988966529 | -0.280356705 | 0.0608903224964001 | -1.121984556 | 0.366275438800159 |
| DBR1 | -0.660992874 | 0.339007126 | -15.26163212 | 8.16128482982706 | -0.525536198 | 0.616550083670876 |
| DBT | -0.03953109 | 0.96046891 | -0.976710895 | 0.228449646666888 | -1.156773212 | 0.366275438800159 |
| DCAF1 | -0.445863306 | 0.554136694 | -13.98344771 | 6.86352677162515 | -0.767859509 | 0.539497285386707 |
| DCAF11 | -1.121209673 | -0.121209673 | -14.51906758 | 7.90434656929043 | -0.516819382 | 0.621704967022904 |
| DCAF13 | -34.83846003 | -33.83846003 | -12.8484828 | 6.70447909188138 | -0.707106781 | 0.539497285386707 |
| DCAF7 | -0.004122759 | 0.995877241 | -0.32765827 | 0.248748182006163 | -0.281721444 | 0.798257295858548 |
| DCAF8 | -1.572342222 | -0.572342222 | -16.27658181 | 4.42504373949626 | -0.99055657 | 0.390630988606417 |
| DCAKD | 0.0341940826429511 | 0.0341940826429511 | -0.162587221 | 0.808168313131486 | 1.1747452608855 | 0.352931421519169 |
| DCD | -0.01714344 | 0.98285656 | -1.110100728 | 0.776228648916068 | -0.346730879 | 0.741612098703883 |
| DCHS1 | 2.11728714188738 | 2.11728714188738 | 0.509663652973741 | 20.8001218673206 | 2.35887310397428 | 0.170127248166302 |
| DCK | -0.446135999 | 0.553864001 | -13.35072035 | 6.55293323902158 | -0.766690247 | 0.539497285386707 |
| DCLK1 | 0.0601719368981609 | 0.0601719368981609 | 0.0304321773673939 | 1.09362964575209 | 1.87093836733034 | 0.164702566587332 |
| DCN | 0.108259911128787 | 0.108259911128787 | 0.571585662463919 | 2.1081482553415 | 3.10993902722931 | 0.0548717713947671 |
| DCP1A | -0.840822813 | 0.159177187 | -13.26013643 | 8.39487839270132 | -0.38883864 | 0.697580737886459 |
| DCPS | -0.030566336 | 0.969433664 | -0.739291575 | 0.0931885914564625 | -1.342902777 | 0.275680790309397 |
| DCTD | 0.0397539727475675 | 0.0397539727475675 | 0.000542504093079059 | 0.763556549764861 | 1.76049167193041 | 0.179117559639718 |
| DCTN1 | 0.0364933195936511 | 0.0364933195936511 | 0.0591216883881247 | 0.660016029626337 | 2.2007475439818 | 0.127493113256436 |
| DCTN2 | 0.0288814741228924 | 0.0288814741228924 | 0.0585116861693175 | 0.550209753793468 | 2.1599372137606 | 0.114859418901757 |
| DCTN3 | 0.0376429818699674 | 0.0376429818699674 | 0.108520262025103 | 0.660821471183318 | 2.41851840953042 | 0.0868066317151366 |
| DCTN4 | 0.0224111078235301 | 0.0224111078235301 | -0.210420771 | 0.629939961906068 | 0.864053613540393 | 0.464168969199592 |
| DCTN5 | 0.884437510077132 | 0.884437510077132 | -8.997725698 | 14.5644678022523 | 0.409189549259266 | 0.681576293714366 |
| DCTN6 | 0.0422240159773172 | 0.0422240159773172 | -0.100431784 | 0.882988347822122 | 1.52918688538061 | 0.24853721137945 |
| DCTPP1 | -36.42733356 | -35.42733356 | -19.05280396 | 0.570757374057598 | -2.119426307 | 0.1803694486231 |
| DCUN1D1 | 0.0222063668458734 | 0.0222063668458734 | -0.086736327 | 0.498099896242809 | 1.2175312356181 | 0.329477610805232 |
| DCUN1D5 | 0.510840110778653 | 0.510840110778653 | -7.937275833 | 12.6586670835458 | 0.400199253891149 | 0.688779559347009 |
| DCXR | -0.071073916 | 0.928926084 | -1.153460481 | -0.300520929 | -3.669867444 | 0.0768138743236135 |
| DDAH1 | 0.081286373748201 | 0.081286373748201 | 0.431040895420075 | 1.09684590356114 | 4.41957115835302 | 0.037807978546348 |
| DDAH2 | 0.0511331585585052 | 0.0511331585585052 | 0.259325024231814 | 0.844159846002924 | 3.26532005290603 | 0.0423142442798193 |
| DDB1 | 0.00456468612516177 | 0.00456468612516177 | -0.234313161 | 0.331317616390073 | 0.341734256442256 | 0.747014066450092 |
| DDB2 | -0.038150618 | 0.961849382 | -13.60293225 | 13.247653437452 | -0.022897731 | 0.999130434024431 |
| DDI2 | -0.001461244 | 0.998538756 | -0.704645566 | 0.675416711345123 | -0.040938109 | 0.995327285436105 |
| DDOST | -0.066506561 | 0.933493439 | -1.299998791 | -0.262717187 | -3.200870305 | 0.0919270569052632 |
| DDRGK1 | -0.085758306 | 0.914241694 | -1.226521941 | -0.573258781 | -4.904315022 | 0.0163865667283959 |
| DDT | -0.007558549 | 0.992441451 | -0.568733174 | 0.42463973796134 | -0.250977763 | 0.818092393826308 |
| DDX1 | -0.004910064 | 0.995089936 | -0.476521149 | 0.369682962490513 | -0.258453439 | 0.818267552758893 |
| DDX17 | -0.021138248 | 0.978861752 | -0.406072677 | -0.043021064 | -2.159232425 | 0.114919247158557 |
| DDX18 | -0.456920649 | 0.543079351 | -13.36033151 | 6.40846818646787 | -0.789672329 | 0.539497285386707 |
| DDX19A | 0 | 1 | NA | NA | NA | NA |
| DDX19B | 0 | 1 | NA | NA | NA | NA |
| DDX21 | -0.076300637 | 0.923699363 | -1.185894259 | -0.355254665 | -3.219239963 | 0.0444366797071688 |
| DDX23 | -0.030407159 | 0.969592841 | -0.486390525 | -0.127388077 | -2.958336423 | 0.0538679412155676 |
| DDX24 | -2.094116035 | -1.094116035 | -19.28146049 | -0.26694227 | -2.310776671 | 0.174626078133574 |
| DDX27 | -0.022293847 | 0.977706153 | -0.733542049 | 0.31208929994331 | -0.710926457 | 0.539497285386707 |
| DDX31 | 0.963057561451296 | 0.963057561451296 | -7.805909514 | 13.2012575183775 | 0.445880073336456 | 0.657887841997856 |
| DDX39A | -36.91310275 | -35.91310275 | -13.14196615 | -12.7390401 | -144.5446064 | 0.000135517346157358 |
| DDX39B | -0.010387659 | 0.989612341 | -0.536345697 | 0.328475401653005 | -0.424489015 | 0.671970886340401 |
| DDX3X | -0.010924127 | 0.989075873 | -0.440776501 | 0.228954806372747 | -0.573957579 | 0.589099143327268 |
| DDX3Y | 0.00153123567323382 | 0.00153123567323382 | -13.62742331 | 13.6419042202346 | 0.000918808792363488 | 0.999577493567163 |
| DDX41 | -0.029945038 | 0.970054962 | -0.576408568 | 0.052804359721471 | -1.744018963 | 0.22130836730508 |
| DDX42 | -0.002864917 | 0.997135083 | -0.265796867 | 0.209206540317953 | -0.211100308 | 0.855883134600213 |
| DDX46 | -0.042606301 | 0.957393699 | -0.563521652 | -0.275047466 | -5.660442709 | 0.0232301628989628 |
| DDX47 | -0.036294464 | 0.963705536 | -0.847351163 | 0.203176180724294 | -1.077855486 | 0.366275438800159 |
| DDX5 | -0.035915915 | 0.964084085 | -0.662327115 | -0.074456293 | -2.581992093 | 0.124138605311285 |
| DDX50 | -0.018827835 | 0.981172165 | -0.638440997 | 0.286704229625236 | -0.738209145 | 0.539497285386707 |
| DDX51 | -1.984047893 | -0.984047893 | -18.89105344 | 0.678800673348332 | -2.087024491 | 0.181184707940497 |
| DDX52 | -0.629546099 | 0.370453901 | -15.61520265 | 8.63620936233917 | -0.49941398 | 0.631332353576646 |
| DDX54 | -0.008810194 | 0.991189806 | -0.369678372 | 0.204982514063828 | -0.505428986 | 0.628849803965199 |
| DDX55 | -0.392789709 | 0.607210291 | -13.64823756 | 7.42909957248946 | -0.658664 | 0.561015630932865 |
| DDX56 | -0.425245294 | 0.574754706 | -13.39138935 | 6.84171133991802 | -0.725579163 | 0.539497285386707 |
| DDX58 | -0.402452362 | 0.597547638 | -14.14044056 | 7.55814118651831 | -0.682405413 | 0.551061525652585 |
| DDX59 | -34.72505807 | -33.72505807 | -11.87721053 | 6.19765858318791 | -0.707106781 | 0.539497285386707 |
| DDX6 | -0.01347409 | 0.98652591 | -0.398807068 | 0.12390782174926 | -0.97887814 | 0.412610536628868 |
| DDX60 | 0.430631257132281 | 0.430631257132281 | -7.723962283 | 15.291201868289 | 0.732446454608585 | 0.539497285386707 |
| DDX60L | 34.773304080641 | 34.773304080641 | -6.408422612 | 12.2811193165852 | 0.707106781186547 | 0.539497285386707 |
| DECR1 | -0.028806199 | 0.971193801 | -0.526942615 | -0.083051995 | -2.515417597 | 0.095357341084507 |
| DECR2 | -0.989134565 | 0.010865435 | -18.40594478 | 5.52778108841684 | -1.202618697 | 0.36731973075278 |
| DEF6 | -2.09426365 | -1.09426365 | -20.20857082 | -0.276204321 | -2.311981174 | 0.174665065873632 |
| DEFA1B | -35.92661574 | -34.92661574 | -18.54101209 | 5.47873244813162 | -1.223761201 | 0.366275438800159 |
| DEFA3 | -36.95670001 | -35.95670001 | -14.51372472 | -12.16132508 | -25.51762334 | 0.00221572006546813 |
| DEFA4 | -0.770089467 | 0.229910533 | -16.08239445 | 7.66064458154858 | -0.613735488 | 0.560965177743621 |
| DEFA5 | 0 | 1 | NA | NA | NA | NA |
| DEFB4B | 0 | 1 | NA | NA | NA | NA |
| DEK | -0.011439666 | 0.988560334 | -0.48492517 | 0.261808058667693 | -0.623199609 | 0.572353578183855 |
| DENND10 | 0 | 1 | NA | NA | NA | NA |
| DENND3 | -1.704801181 | -0.704801181 | -16.19938968 | 3.74727294640634 | -1.081942106 | 0.366275438800159 |
| DENND4C | -0.721004062 | 0.278995938 | -15.39170767 | 7.71884043431788 | -0.574803544 | 0.58331334336005 |
| DENR | -0.006230265 | 0.993769735 | -0.335143674 | 0.215634929725363 | -0.376847134 | 0.70792343851302 |
| DEPTOR | -0.57047011 | 0.42952989 | -14.4019895 | 8.47619760327924 | -0.450689648 | 0.656161617379267 |
| DERA | 0.00811442419712567 | 0.00811442419712567 | -0.056034454 | 0.21415432100292 | 1.03102602837249 | 0.378609149301149 |
| DERL1 | -0.124685275 | 0.875314725 | -1.58865795 | -0.895496516 | -7.213638701 | 0.0163865667283959 |
| DERL3 | -37.14788902 | -36.14788902 | -15.91552202 | -14.53949638 | -49.80562868 | 0.00044044858460524 |
| DERPC | -0.996766383 | 0.003233617 | -19.05084546 | 5.65935956355948 | -1.218409841 | 0.366275438800159 |
| DES | 0.0818463522385647 | 0.0818463522385647 | -0.400512287 | 2.16186191289253 | 1.19375832426952 | 0.3412183162817 |
| DFFA | 0.00828398368297808 | 0.00828398368297808 | -0.327372623 | 0.472607612731683 | 0.316413362626979 | 0.760373960359508 |
| DGAT2L6 | 0 | 1 | NA | NA | NA | NA |
| DGKA | 0.0296166685466573 | 0.0296166685466573 | 0.0430475120814399 | 0.527482683519477 | 2.039044356 | 0.127997839858642 |
| DGKQ | 34.6446803118642 | 34.6446803118642 | -5.861807222 | 11.2335840276238 | 0.707106781186547 | 0.539497285386707 |
| DGLUCY | -0.002384339 | 0.997615661 | -0.318552309 | 0.270113141002314 | -0.163158787 | 0.90292026073532 |
| DGUOK | -1.043809102 | -0.043809102 | -17.83153102 | 4.77276276637971 | -1.299753191 | 0.364559456625742 |
| DHCR24 | -35.95975618 | -34.95975618 | -18.9640673 | 5.5982585226097 | -1.224536804 | 0.366275438800159 |
| DHCR7 | -0.03146317 | 0.96853683 | -0.83804901 | 0.240154961575853 | -1.123432687 | 0.373099048271877 |
| DHODH | -1.96838819 | -0.96838819 | -20.14919707 | 0.872357955302525 | -2.057194844 | 0.184643860681916 |
| DHRS1 | -0.025202814 | 0.974797186 | -0.638057465 | 0.134567967079061 | -1.226368906 | 0.350680114094073 |
| DHRS11 | 0.0270927123090555 | 0.0270927123090555 | -10.91520316 | 11.2763688097233 | 0.0281616353031831 | 0.998435102357591 |
| DHRS4 | -0.076426824 | 0.923573176 | -2.125599025 | 0.776167674762983 | -0.805432169 | 0.500030138248588 |
| DHRS7 | -0.053473592 | 0.946526408 | -0.948950345 | -0.191537449 | -2.940689503 | 0.0847737972621843 |
| DHRS7B | -0.002273685 | 0.997726315 | -0.57446987 | 0.532713749102863 | -0.07475156 | 0.975895096866426 |
| DHRS7C | 34.9619105184224 | 34.9619105184224 | -7.303437874 | 13.9963291087043 | 0.707106781186548 | 0.539497285386707 |
| DHRS9 | -34.85506965 | -33.85506965 | -12.99726099 | 6.78211318412454 | -0.707106781 | 0.539497285386707 |
| DHRSX | -1.573153349 | -0.573153349 | -16.34075042 | 4.44001998238889 | -0.990876374 | 0.39062478731047 |
| DHTKD1 | -1.123943579 | -0.123943579 | -18.57498012 | 4.04845822158851 | -1.443979997 | 0.325280869222481 |
| DHX15 | -0.03629784 | 0.96370216 | -0.560203678 | -0.190256687 | -3.768092695 | 0.04345047369545 |
| DHX16 | -0.387546162 | 0.612453838 | -14.04747724 | 7.71383471382467 | -0.652711965 | 0.563384306397679 |
| DHX29 | -0.017218462 | 0.982781538 | -0.439543464 | 0.126592751941999 | -1.142678578 | 0.370420782721663 |
| DHX30 | -0.013792756 | 0.986207244 | -0.607700272 | 0.347329717592594 | -0.575102315 | 0.602253180989618 |
| DHX32 | 34.5205174877197 | 34.5205174877197 | -5.37842104 | 10.3072213750538 | 0.707106781186547 | 0.539497285386707 |
| DHX36 | -0.413064052 | 0.586935948 | -13.42777065 | 7.01960796763942 | -0.7011632 | 0.54154545688419 |
| DHX37 | -36.83781844 | -35.83781844 | -12.69390494 | -11.87118286 | -67.19102309 | 0.000260229699772871 |
| DHX38 | -0.030159492 | 0.969840508 | -0.557684389 | -0.013613725 | -2.05431855 | 0.168190760193245 |
| DHX57 | 0.350306456768057 | 0.350306456768057 | -7.080833138 | 12.0324642655906 | 0.582883735566304 | 0.602987071525983 |
| DHX58 | 34.7671180132782 | 34.7671180132782 | -6.381003052 | 12.2285723931659 | 0.707106781186547 | 0.539497285386707 |
| DHX8 | -1.129672498 | -0.129672498 | -13.91733872 | 7.5366213744157 | -0.521267032 | 0.619760639471857 |
| DHX9 | -0.022153946 | 0.977846054 | -0.453976275 | -0.035699436 | -2.19200847 | 0.134033116286307 |
| DIABLO | -0.001999772 | 0.998000228 | -0.328699167 | 0.288582163680359 | -0.123403649 | 0.936060640159654 |
| DIAPH1 | -0.013933532 | 0.986066468 | -0.354700463 | 0.0900800979549971 | -1.052351824 | 0.371925820869984 |
| DIAPH2 | 0.0489446587950442 | 0.0489446587950442 | -10.77651697 | 11.4290545919478 | 0.0508594852857466 | 0.990919784807717 |
| DICER1 | -36.43795847 | -35.43795847 | -19.18712448 | 0.568462169276411 | -2.12082401 | 0.1803694486231 |
| DIDO1 | -0.372454542 | 0.627545458 | -13.98491677 | 7.91720830895555 | -0.622989994 | 0.579161231845404 |
| DIMT1 | -1.037755288 | -0.037755288 | -16.93064558 | 4.54016873762557 | -1.282400342 | 0.366275438800159 |
| DIP2A | 36.4278068583387 | 36.4278068583387 | -0.57550793 | 19.0636187742064 | 2.11844138143756 | 0.1803694486231 |
| DIP2B | -1.064279383 | -0.064279383 | -17.29955519 | 4.40899906793927 | -1.336081239 | 0.356621571555239 |
| DIPK2A | 0.54061420193737 | 0.54061420193737 | -5.631976996 | 14.0663346360883 | 0.96340301647014 | 0.462668474276794 |
| DIS3 | -0.030061482 | 0.969938518 | -0.484536307 | -0.088991614 | -2.625459337 | 0.0841851891763818 |
| DIS3L2 | -1.555605277 | -0.555605277 | -17.44819052 | 4.83728699084799 | -0.979191393 | 0.396018957882372 |
| DKC1 | -0.046325362 | 0.953674638 | -0.878442483 | -0.041025554 | -2.060535135 | 0.154106165589444 |
| DKK2 | 0 | 1 | NA | NA | NA | NA |
| DLAT | -0.055915481 | 0.944084519 | -0.810798808 | -0.37598741 | -4.769274804 | 0.015412611548611 |
| DLD | -0.022612506 | 0.977387494 | -0.435832938 | -0.038547082 | -2.068615755 | 0.124570854411906 |
| DLG1 | 0.0652941391563211 | 0.0652941391563211 | -0.385974215 | 1.63587182789095 | 1.27872356761146 | 0.355515289923087 |
| DLGAP4 | 0.0472210801907479 | 0.0472210801907479 | -0.020557987 | 0.887527762838686 | 1.70198148853386 | 0.1803694486231 |
| DLGAP5 | 0 | 1 | NA | NA | NA | NA |
| DLST | 0.00574998834010399 | 0.00574998834010399 | -0.193164444 | 0.323422428486259 | 0.458181154594054 | 0.655380640029902 |
| DMAC2L | -0.469242778 | 0.530757222 | -13.7777584 | 6.44702430363165 | -0.814728531 | 0.532766709927747 |
| DMAP1 | -1.032781832 | -0.032781832 | -17.72120123 | 4.86502278549756 | -1.280016137 | 0.366275438800159 |
| DMBT1 | 35.9150401630916 | 35.9150401630916 | -5.442046424 | 18.3999391497164 | 1.22303263688702 | 0.366275438800159 |
| DMD | 0.0405226243977109 | 0.0405226243977109 | -0.286916806 | 1.07535671398076 | 1.13913653519872 | 0.366275438800159 |
| DMKN | 35.0306137566044 | 35.0306137566044 | -7.659652634 | 14.6789800891159 | 0.707106781186547 | 0.539497285386707 |
| DMP1 | -1.082413777 | -0.082413777 | -16.78120695 | 9.30869183146244 | -0.500324204 | 0.631332353576646 |
| DMPK | 0 | 1 | NA | NA | NA | NA |
| DMRT2 | -0.464040624 | 0.535959376 | -15.13184204 | 11.8937118120797 | -0.209853819 | 0.856633712253738 |
| DMTN | -0.004157481 | 0.995842519 | -1.148766797 | 1.06309132633055 | -0.067344289 | 0.980196893396618 |
| DMXL2 | -0.084520815 | 0.915479185 | -10.92754633 | 10.5642528147833 | -0.029271758 | 0.998435102357591 |
| DNAAF5 | 0.041602645903263 | 0.041602645903263 | 0.00974708314538028 | 0.707917955678075 | 1.77916037503049 | 0.172277500107103 |
| DNAH17 | 0.099237457807658 | 0.099237457807658 | 0.528279684414474 | 1.68664133489351 | 3.89651478216445 | 0.0574056423986682 |
| DNAJA1 | 0.0324145458201421 | 0.0324145458201421 | -0.041609543 | 0.666808391723019 | 1.64893195302276 | 0.205508992663539 |
| DNAJA2 | 0.00254244243869885 | 0.00254244243869885 | -0.087791353 | 0.140387662358457 | 0.454936939195996 | 0.660075326925812 |
| DNAJA3 | -0.039498178 | 0.960501822 | -0.617887056 | -0.122851531 | -3.233664987 | 0.0943191190788667 |
| DNAJA4 | 34.7327946472202 | 34.7327946472202 | -6.230983333 | 11.9410741778449 | 0.707106781186548 | 0.539497285386707 |
| DNAJB1 | 0.00145010630442321 | 0.00145010630442321 | -0.202861688 | 0.229890082637062 | 0.110008474119269 | 0.945587956124106 |
| DNAJB11 | -0.096614064 | 0.903385936 | -1.304379806 | -0.786401549 | -7.051356656 | 0.0032628612408553 |
| DNAJB12 | 0.0278952424491463 | 0.0278952424491463 | -0.519852207 | 1.00617459790319 | 0.593748270260454 | 0.57827242635708 |
| DNAJB2 | 0.0394555329498082 | 0.0394555329498082 | 0.00980618176650527 | 0.700510130958628 | 1.78168278694924 | 0.17224744541596 |
| DNAJB4 | -0.027283279 | 0.972716721 | -1.022598164 | 0.546814591819702 | -0.525606806 | 0.616550083670876 |
| DNAJB6 | -0.993572106 | 0.006427894 | -19.39181277 | 5.79711079 | -1.213103951 | 0.366735091564231 |
| DNAJB9 | -34.92050821 | -33.92050821 | -13.60037268 | 7.09682347160436 | -0.707106781 | 0.539497285386707 |
| DNAJC1 | -0.106424001 | 0.893575999 | -1.579989563 | -0.477650626 | -3.375996857 | 0.0489656305565222 |
| DNAJC10 | -0.01073773 | 0.98926227 | -0.436708157 | 0.236554835932665 | -0.568217027 | 0.597133145505151 |
| DNAJC11 | 0.00162646019303211 | 0.00162646019303211 | -0.217540779 | 0.249032080386909 | 0.116845148494301 | 0.94009972124765 |
| DNAJC13 | 0.027579640467508 | 0.027579640467508 | -0.008546148 | 0.531255548262711 | 1.69087422894296 | 0.1803694486231 |
| DNAJC17 | -35.89491555 | -34.89491555 | -18.1526859 | 5.37429212341111 | -1.222239197 | 0.366275438800159 |
| DNAJC19 | -1.000561031 | -0.000561031 | -18.76482735 | 5.53013999826512 | -1.225281972 | 0.366275438800159 |
| DNAJC2 | -34.71579442 | -33.71579442 | -11.80119044 | 6.15799046839688 | -0.707106781 | 0.539497285386707 |
| DNAJC25 | -0.006388828 | 0.993611172 | -13.89242673 | 13.8309808175934 | -0.003834879 | 0.999130434024431 |
| DNAJC3 | -0.067485215 | 0.932514785 | -1.017874187 | -0.344646484 | -3.547434374 | 0.0378017385946239 |
| DNAJC30 | 0 | 1 | NA | NA | NA | NA |
| DNAJC5 | -36.34704871 | -35.34704871 | -18.01331024 | 0.531680818253229 | -2.121296467 | 0.1803694486231 |
| DNAJC7 | -0.460197413 | 0.539802587 | -13.905881 | 6.63055434576309 | -0.796855096 | 0.538825992065788 |
| DNAJC8 | -0.04788052 | 0.95211948 | -0.835039636 | -0.068754325 | -2.355635064 | 0.135713069910199 |
| DNAJC9 | -1.078402079 | -0.078402079 | -18.76197659 | 4.60940920580168 | -1.360491935 | 0.350426488005461 |
| DNASE1L1 | -0.414765968 | 0.585234032 | -13.38438002 | 6.98556277418946 | -0.705169289 | 0.539958598407219 |
| DNASE2 | -0.543946213 | 0.456053787 | -15.60122826 | 6.19746938547455 | -0.967174757 | 0.460697898437451 |
| DNM1 | 0.1176846609994 | 0.1176846609994 | 0.355820304117188 | 1.92650260809488 | 3.04413635305104 | 0.0971038181262111 |
| DNM1L | -0.001497221 | 0.998502779 | -0.259896222 | 0.229354713653412 | -0.117799786 | 0.940777264296504 |
| DNM1P34 | 0.550650710795337 | 0.550650710795337 | -6.311167471 | 16.1495128342826 | 0.984738643267472 | 0.451274415166418 |
| DNM2 | -0.013053442 | 0.986946558 | -0.298367403 | 0.0436595860489422 | -1.602735922 | 0.265815462779191 |
| DNM3 | -0.000187142 | 0.999812858 | -0.720493626 | 0.717043189180747 | -0.004695087 | 0.999130434024431 |
| DNMT1 | -0.502340516 | 0.497659484 | -13.2512204 | 5.78350574575469 | -0.881329439 | 0.503368350005806 |
| DNPEP | -0.007760333 | 0.992239667 | -0.303662205 | 0.143610665611452 | -0.633846548 | 0.552716415991246 |
| DNPH1 | 0.00820319281096362 | 0.00820319281096362 | -0.795625918 | 0.956623617308341 | 0.16087961027044 | 0.902427899166895 |
| DNTTIP1 | 0.58520269108749 | 0.58520269108749 | -8.206470892 | 14.1642744786001 | 0.463076799597679 | 0.652016273769328 |
| DNTTIP2 | -0.978107972 | 0.021892028 | -17.0971391 | 5.27139499959808 | -1.18628459 | 0.371925820869984 |
| DOCK1 | 0.506608722298429 | 0.506608722298429 | -6.300041709 | 14.5849896268405 | 0.891504222164215 | 0.497859011640708 |
| DOCK10 | -0.448365712 | 0.551634288 | -14.06642496 | 6.8728320120535 | -0.772932818 | 0.539497285386707 |
| DOCK11 | -0.999740397 | 0.000259603 | -17.6205907 | 5.19991363347774 | -1.223907085 | 0.366275438800159 |
| DOCK2 | -0.087725056 | 0.912274944 | -1.094419519 | -0.596410801 | -5.975993274 | 0.00742123975108202 |
| DOCK5 | 36.2629869619038 | 36.2629869619038 | -0.52193466 | 17.0140667135488 | 2.1163735238868 | 0.1803694486231 |
| DOCK6 | 36.2860375896716 | 36.2860375896716 | -0.530374178 | 17.2881252132385 | 2.11636560331046 | 0.1803694486231 |
| DOCK7 | 0.500185827535939 | 0.500185827535939 | -6.070909567 | 13.8503679777771 | 0.878613159944844 | 0.505105500054318 |
| DOCK8 | -0.530084114 | 0.469915886 | -14.79459474 | 6.06581106970302 | -0.941387736 | 0.473444566548856 |
| DOCK9 | -0.57144264 | 0.42855736 | -15.38676939 | 9.04561676810149 | -0.451585902 | 0.655781420491697 |
| DOHH | 1.48300651715199 | 1.48300651715199 | -4.773523777 | 15.8055424238432 | 0.928650920186332 | 0.424267295692596 |
| DOK2 | -0.494270696 | 0.505729304 | -13.61820963 | 6.05460935795241 | -0.862633484 | 0.511754197162799 |
| DOK3 | -2.162304696 | -1.162304696 | -19.97033739 | -0.881768327 | -2.458015949 | 0.161304083203444 |
| DOLPP1 | -35.87470693 | -34.87470693 | -17.87659056 | 5.27594288015975 | -1.224730224 | 0.366275438800159 |
| DOP1A | 0 | 1 | NA | NA | NA | NA |
| DOP1B | 0 | 1 | NA | NA | NA | NA |
| DPAGT1 | -2.112099843 | -1.112099843 | -19.27716023 | -0.455921663 | -2.34220949 | 0.170314078628347 |
| DPEP1 | -0.142872257 | 0.857127743 | -13.58547347 | 11.4491774809897 | -0.147988013 | 0.912368055659318 |
| DPF2 | -0.452066617 | 0.547933383 | -14.15213685 | 6.85459978333835 | -0.779732783 | 0.539497285386707 |
| DPH2 | -0.562936414 | 0.437063586 | -14.37132005 | 8.52228789234816 | -0.44473215 | 0.658224492461674 |
| DPM1 | -0.054699107 | 0.945300893 | -0.730406858 | -0.402518249 | -6.638750421 | 0.0143440996769497 |
| DPM3 | -0.087223771 | 0.912776229 | -1.437585785 | -0.343896923 | -3.254469352 | 0.0761918355955649 |
| DPP3 | -0.034480313 | 0.965519687 | -0.76966525 | 0.0877298599802438 | -1.598313859 | 0.244457704982357 |
| DPP4 | 0.0227593376576796 | 0.0227593376576796 | -0.055802219 | 0.47734259850499 | 1.38297822022915 | 0.264433826910479 |
| DPP7 | -0.15726513 | 0.84273487 | -1.960692893 | -1.342236774 | -10.50881799 | 0.00505975111662158 |
| DPP9 | -0.022719541 | 0.977280459 | -0.444774852 | 0.0320647425194053 | -1.524617768 | 0.217517133624183 |
| DPT | 0.0529801992515008 | 0.0529801992515008 | -0.160869706 | 1.36782786179234 | 1.37254802745084 | 0.265815462779191 |
| DPY19L1 | -0.032400193 | 0.967599807 | -1.08006848 | 0.550596895482616 | -0.7116333 | 0.539497285386707 |
| DPY19L3 | 0.461426102176374 | 0.461426102176374 | -6.739851227 | 14.1700872325082 | 0.798785572744859 | 0.537923342132161 |
| DPY30 | -0.055830527 | 0.944169473 | -1.284382699 | 0.133430565544713 | -1.710419538 | 0.230248322668736 |
| DPYD | -0.023771007 | 0.976228993 | -0.769550487 | 0.332847698668503 | -0.810481321 | 0.5238430353643 |
| DPYS | 1.14665897779128 | 1.14665897779128 | -3.708327637 | 18.1530926291607 | 1.4868148831198 | 0.312367842894804 |
| DPYSL2 | 0.0610891810102584 | 0.0610891810102584 | 0.434111371348869 | 0.945530588182685 | 4.74327333150685 | 0.0163802625627815 |
| DPYSL3 | 0.110588191019812 | 0.110588191019812 | 0.784799961298003 | 1.72624230341749 | 4.6410763276174 | 0.0159729447348982 |
| DPYSL4 | 0.0516765386873854 | 0.0516765386873854 | -0.078111654 | 1.07508974337206 | 1.67231892678825 | 0.211294118489054 |
| DPYSL5 | 1.20519258714906 | 1.20519258714906 | -3.434583706 | 22.2399624117149 | 1.55342273540205 | 0.275138545491146 |
| DR1 | -0.003260752 | 0.996739248 | -0.526286025 | 0.461742299412135 | -0.129946269 | 0.930753380899663 |
| DRAP1 | 0.0124701980419406 | 0.0124701980419406 | -0.230438454 | 0.484055212366387 | 0.771155324724659 | 0.539497285386707 |
| DRG1 | -0.032533936 | 0.967466064 | -0.593494889 | -0.002111326 | -1.987825052 | 0.177846384633647 |
| DRG2 | 0.00836553645210561 | 0.00836553645210561 | -0.258302099 | 0.42060684515746 | 0.427587866986412 | 0.670184726509629 |
| DSC1 | 0.107687475149722 | 0.107687475149722 | -11.13032216 | 11.6198228358341 | 0.0372783865439542 | 0.996444558886932 |
| DSC2 | 0.115032547438961 | 0.115032547438961 | -12.27444587 | 12.8517413324583 | 0.0398145402346913 | 0.995327285436105 |
| DSC3 | 0.174555254563956 | 0.174555254563956 | -12.19268245 | 13.0702070545134 | 0.0603125511049029 | 0.986245618759808 |
| DSG1 | 0.587493299212605 | 0.587493299212605 | -8.606778862 | 14.8825633427107 | 0.464443066233928 | 0.651481689963059 |
| DSG2 | -34.94242653 | -33.94242653 | -13.80857558 | 7.20546602798934 | -0.707106781 | 0.539497285386707 |
| DSG3 | 0.5525386673131 | 0.5525386673131 | -8.878720928 | 14.7915123342837 | 0.435176706503239 | 0.663729018975284 |
| DSP | -0.00586055 | 0.99413945 | -1.19290161 | 1.07992371917841 | -0.086774631 | 0.967099093409502 |
| DSPP | 35.9317458166677 | 35.9317458166677 | -5.488573584 | 18.597384013601 | 1.22474437261702 | 0.366275438800159 |
| DST | 0.506462990564682 | 0.506462990564682 | -5.969851143 | 13.7946026631339 | 0.888897332229241 | 0.498769147875224 |
| DSTN | 0.0627876446905612 | 0.0627876446905612 | 0.226352885263612 | 1.06712752146398 | 2.81158916305177 | 0.0757988675671794 |
| DTD1 | 0.0172729763108647 | 0.0172729763108647 | -0.214753625 | 0.545454869864336 | 0.772544612574667 | 0.523387983067527 |
| DTNA | 35.7576466500182 | 35.7576466500182 | -4.864662701 | 16.4832737071573 | 1.22474139921754 | 0.366275438800159 |
| DTNBP1 | 0.571420850114713 | 0.571420850114713 | -8.920721743 | 15.1767487679385 | 0.451715732411327 | 0.655781420491697 |
| DTX3L | -0.410348646 | 0.589651354 | -14.08025998 | 7.41192342750625 | -0.697509842 | 0.543715986631775 |
| DTYMK | 0.00850315029341286 | 0.00850315029341286 | -0.203521528 | 0.377072579388155 | 0.517255927159381 | 0.621057728482762 |
| DUOX1 | 0.554743524660256 | 0.554743524660256 | -5.240459795 | 13.4454060499568 | 0.986952577234409 | 0.450754258845013 |
| DUOX2 | 0 | 1 | NA | NA | NA | NA |
| DUOXA2 | 0 | 1 | NA | NA | NA | NA |
| DUSP14 | 0 | 1 | NA | NA | NA | NA |
| DUSP22 | -1.014527971 | -0.014527971 | -14.33235193 | 8.24210348404308 | -0.46945403 | 0.648356244122926 |
| DUSP23 | 0.00447772839807018 | 0.00447772839807018 | -0.206638564 | 0.29324525912262 | 0.302262094258419 | 0.772588728688433 |
| DUSP3 | 0.00740554956267331 | 0.00740554956267331 | -0.250048168 | 0.390135196263755 | 0.378715035337284 | 0.705941037662548 |
| DUT | 0.0174274036311257 | 0.0174274036311257 | -0.182491731 | 0.520782318068571 | 0.896952663574568 | 0.461366804240842 |
| DVL2 | 0.988899535539694 | 0.988899535539694 | -5.722427474 | 18.9228179590262 | 1.20427614365117 | 0.36731973075278 |
| DYNC1H1 | 0.0374067111715542 | 0.0374067111715542 | 0.135613129534413 | 0.668202718864613 | 2.64225465162246 | 0.0740912809027584 |
| DYNC1I2 | 0.0506324083841743 | 0.0506324083841743 | 0.344108790451594 | 0.711450577404007 | 5.09547595906609 | 0.0144289042301664 |
| DYNC1LI1 | 0.00357535085960412 | 0.00357535085960412 | -0.182753022 | 0.250741386590729 | 0.283419364210827 | 0.790956301899392 |
| DYNC1LI2 | 0.0413745212235463 | 0.0413745212235463 | 0.201634590886204 | 0.584490328501158 | 4.1429996986385 | 0.051055672178882 |
| DYNC2H1 | 0.416921918410163 | 0.416921918410163 | -6.950664867 | 13.3735686569478 | 0.710305086610607 | 0.539497285386707 |
| DYNLL1 | 0.0196892719516613 | 0.0196892719516613 | -0.252449957 | 0.621601003928078 | 0.735325475579806 | 0.537529052247127 |
| DYNLL2 | 0.0129364565935718 | 0.0129364565935718 | -0.118397779 | 0.38267457383764 | 1.10581090406407 | 0.38381760326338 |
| DYNLRB1 | 0.427426851777372 | 0.427426851777372 | -7.175117811 | 14.0908115212534 | 0.731018320562903 | 0.539497285386707 |
| DYNLT1 | 0.0525929413378131 | 0.0525929413378131 | -9.901603898 | 10.5461427844028 | 0.0545534867746396 | 0.988843825961911 |
| DYNLT3 | 0.0371795828274526 | 0.0371795828274526 | 0.0877653847423056 | 0.590653643286486 | 2.33934609426622 | 0.0934460762426867 |
| DYSF | -0.064005958 | 0.935994042 | -1.111249178 | -0.088131811 | -2.0321047 | 0.129238487032554 |
| EARS2 | -1.058069081 | -0.058069081 | -19.23256668 | 4.97789181883277 | -1.323107068 | 0.359878701134842 |
| EBNA1BP2 | -0.04211005 | 0.95788995 | -0.789978648 | 0.00528589486276 | -1.707301827 | 0.1803694486231 |
| EBP | -0.058059175 | 0.941940825 | -0.862020134 | -0.294924924 | -3.53193413 | 0.0352452130660486 |
| ECE1 | -0.392093705 | 0.607906295 | -13.54117853 | 7.37477823564899 | -0.661690471 | 0.56094112803985 |
| ECH1 | -0.043728207 | 0.956271793 | -0.791884393 | -0.137495372 | -2.672407943 | 0.0912221724666125 |
| ECHDC1 | 0.00560018436402956 | 0.00560018436402956 | -0.128235561 | 0.238428572250319 | 0.522959955202174 | 0.618161624117539 |
| ECHDC2 | -1.047673403 | -0.047673403 | -16.36159382 | 4.32397558967198 | -1.305033857 | 0.363301226496658 |
| ECHDC3 | -35.8731495 | -34.8731495 | -17.88918721 | 5.30213502553383 | -1.221362579 | 0.366275438800159 |
| ECHS1 | -0.042089403 | 0.957910597 | -0.679499582 | -0.168220057 | -3.217606567 | 0.0708079654595296 |
| ECI1 | -0.085529523 | 0.914470477 | -1.196308313 | -0.57562697 | -4.950100623 | 0.0123542078611028 |
| ECI2 | 0.00659664804028837 | 0.00659664804028837 | -0.235913994 | 0.358404037201085 | 0.407839904479023 | 0.691260480224731 |
| ECM1 | 0.118729844166763 | 0.118729844166763 | 0.489796196631363 | 1.95355275540329 | 3.32333491061904 | 0.0729045590054497 |
| ECM2 | 1.07462428149097 | 1.07462428149097 | -4.753949999 | 19.1293638045644 | 1.35353411621087 | 0.35243829593768 |
| ECPAS | -0.030453054 | 0.969546946 | -0.516579439 | -0.060827549 | -2.19209001 | 0.108654489227253 |
| ECSIT | -2.004415632 | -1.004415632 | -16.88724428 | 0.45060774617229 | -2.127306308 | 0.1803694486231 |
| EDARADD | 0 | 1 | NA | NA | NA | NA |
| EDC3 | 0.998282898986352 | 0.998282898986352 | -8.527946702 | 14.7020649378214 | 0.462111934501427 | 0.652187811666889 |
| EDC4 | -0.003289192 | 0.996710808 | -0.254614189 | 0.191522328341518 | -0.244863792 | 0.823514424811469 |
| EDF1 | -0.055817865 | 0.944182135 | -1.0974059 | -0.011542961 | -1.767761035 | 0.174416417808595 |
| EDIL3 | 1.19351656141752 | 1.19351656141752 | -3.344014112 | 18.80248283 | 1.56980583818646 | 0.288686171854954 |
| EEA1 | 0.0605791217121278 | 0.0605791217121278 | 0.132310149325174 | 1.01032019064848 | 2.39130466984702 | 0.107763782998397 |
| EEF1A2 | 34.9719717492133 | 34.9719717492133 | -7.354549436 | 14.0942794524436 | 0.707106781186547 | 0.539497285386707 |
| EEF1B2 | -0.061616864 | 0.938383136 | -0.911653155 | -0.473883768 | -5.890374745 | 0.0148074334961127 |
| EEF1D | -0.038077973 | 0.961922027 | -0.857599654 | -0.042728818 | -1.955984532 | 0.149435481381314 |
| EEF1E1 | -0.431225419 | 0.568774581 | -14.04651127 | 7.09900231812592 | -0.738356958 | 0.539497285386707 |
| EEF1G | -0.045410598 | 0.954589402 | -0.966693663 | -0.100676134 | -2.697334518 | 0.130235766873387 |
| EEF2 | -0.053585355 | 0.946414645 | -1.073988262 | -0.207868321 | -3.158416824 | 0.0952861252231188 |
| EEF2K | -34.84023619 | -33.84023619 | -12.86431074 | 6.71273828713198 | -0.707106781 | 0.539497285386707 |
| EEFSEC | 0.00381252929515372 | 0.00381252929515372 | -0.150249984 | 0.224722147346602 | 0.343794402088083 | 0.73720623088191 |
| EFEMP1 | 0.0836068902287346 | 0.0836068902287346 | 0.373612001330434 | 1.45576413771264 | 2.97109476641712 | 0.0563690518057547 |
| EFEMP2 | 0.130294732561873 | 0.130294732561873 | 0.931678629191397 | 1.73694635187284 | 5.76571810285034 | 0.00732524583180956 |
| EFHD1 | 0.494106260206002 | 0.494106260206002 | -5.735621748 | 12.9303454459391 | 0.864973922048944 | 0.510541571566814 |
| EFHD2 | -0.050073805 | 0.949926195 | -0.841474522 | -0.195546959 | -2.795631567 | 0.0627253866892858 |
| EFL1 | 0.0111504611000884 | 0.0111504611000884 | -0.16570361 | 0.363133848283212 | 0.726478152332889 | 0.539497285386707 |
| EFNB1 | 0.0638655122323698 | 0.0638655122323698 | 0.135459394856986 | 0.96390287907141 | 2.31352177207374 | 0.0987975051200024 |
| EFNB2 | 0.540902370840638 | 0.540902370840638 | -4.929401655 | 12.3862545348767 | 0.958353993917474 | 0.462938981503828 |
| EFR3A | 0.0885275197368059 | 0.0885275197368059 | 0.0981889219266893 | 1.46757348804692 | 2.34875227966109 | 0.14495359600462 |
| EFS | 2.1795209670013 | 2.1795209670013 | 1.02146332008641 | 19.6769099109645 | 2.49559354691756 | 0.157321139924891 |
| EFTUD2 | -0.026710777 | 0.973289223 | -0.546993227 | 0.00817240484041505 | -1.779214893 | 0.1803694486231 |
| EGFR | 0.0287941081199233 | 0.0287941081199233 | -0.095589492 | 0.614363326751259 | 1.44414851456487 | 0.286306271894801 |
| EGLN1 | -0.996993335 | 0.003006665 | -17.29911674 | 5.1316899448286 | -1.218724786 | 0.366275438800159 |
| EHBP1L1 | 0.0245859450013597 | 0.0245859450013597 | -0.669611926 | 1.13905337990493 | 0.561508868195298 | 0.613035598848993 |
| EHD1 | -0.003891074 | 0.996108926 | -0.283597526 | 0.204008019978735 | -0.289258808 | 0.784975376088116 |
| EHD2 | 0.0567769141808725 | 0.0567769141808725 | 0.130837916917813 | 1.08915136097989 | 2.30224059218924 | 0.111433033216688 |
| EHD3 | 0.0864266292174917 | 0.0864266292174917 | 0.448508171310783 | 1.27911969786912 | 3.98299668137944 | 0.0444725549358167 |
| EHD4 | -0.016276676 | 0.983723324 | -0.443653912 | 0.110354247864271 | -1.187247637 | 0.366275438800159 |
| EHHADH | -0.992474245 | 0.007525755 | -18.11605397 | 5.43017548416218 | -1.209520902 | 0.36683700319119 |
| EHMT1 | 0.0106704840263009 | 0.0106704840263009 | -10.45344423 | 10.5882025571403 | 0.0110811260160912 | 0.999130434024431 |
| EIF1 | -1.027813677 | -0.027813677 | -13.51778675 | 7.72273057371801 | -0.475131041 | 0.645841531946401 |
| EIF1AX | -0.018248842 | 0.981751158 | -0.86121508 | 0.519856650196535 | -0.436606403 | 0.663729018975284 |
| EIF1AY | -0.127509417 | 0.872490583 | -13.88198664 | 12.7100555541198 | -0.076394172 | 0.973519201696352 |
| EIF2A | -0.045276621 | 0.954723379 | -0.574702879 | -0.316574238 | -6.51402192 | 0.0126163971432531 |
| EIF2AK2 | 0.0020458131379569 | 0.0020458131379569 | -0.124125131 | 0.16138232656102 | 0.247684676395641 | 0.824436169835719 |
| EIF2AK4 | 0.00811185589338467 | 0.00811185589338467 | -0.46486565 | 0.608627039791748 | 0.232833579472257 | 0.834132057836303 |
| EIF2B1 | -0.027083363 | 0.972916637 | -0.493738243 | -0.000577164 | -1.743273662 | 0.179117559639718 |
| EIF2B2 | -0.013691332 | 0.986308668 | -0.472586631 | 0.221870045082236 | -0.697242099 | 0.539497285386707 |
| EIF2B3 | -0.011468863 | 0.988531137 | -0.46861261 | 0.257685001039644 | -0.52252002 | 0.621057728482762 |
| EIF2B4 | -0.058086441 | 0.941913559 | -0.78650795 | -0.344031362 | -5.094194484 | 0.0333552624724531 |
| EIF2B5 | -0.031798532 | 0.968201468 | -0.60459379 | -0.024279581 | -1.962388072 | 0.157321139924891 |
| EIF2D | -0.007930612 | 0.992069388 | -0.327577418 | 0.188672670461445 | -0.531746064 | 0.622155058401711 |
| EIF2S1 | -0.029648923 | 0.970351077 | -0.562713683 | -0.089100511 | -2.410543919 | 0.0900859252365181 |
| EIF2S2 | -0.040952075 | 0.959047925 | -0.619277074 | -0.198766371 | -3.714702063 | 0.0508050551389419 |
| EIF2S3 | -0.046172642 | 0.953827358 | -0.756187122 | -0.170823324 | -3.517225266 | 0.0876930912577496 |
| EIF3A | -0.031208161 | 0.968791839 | -0.727555542 | 0.075849575501763 | -1.564399979 | 0.239423383399016 |
| EIF3B | -0.036555318 | 0.963444682 | -0.735466153 | -0.015484457 | -1.921653285 | 0.169111987184949 |
| EIF3C | -0.034440601 | 0.965559399 | -11.28455291 | 11.0181915515148 | -0.020666829 | 0.999130434024431 |
| EIF3D | -0.037188071 | 0.962811929 | -0.664163336 | -0.110967083 | -2.477190783 | 0.0876189570736668 |
| EIF3E | -0.033659376 | 0.966340624 | -0.71475688 | 0.00190697404053092 | -1.834672684 | 0.1803694486231 |
| EIF3F | -0.034994788 | 0.965005212 | -0.619082158 | -0.109568949 | -2.681841286 | 0.0896004791925918 |
| EIF3G | -0.054478089 | 0.945521911 | -0.805040707 | -0.29302561 | -4.007729176 | 0.0399095149599659 |
| EIF3H | -0.03289335 | 0.96710665 | -0.587619863 | -0.053669508 | -2.201411457 | 0.126559938102032 |
| EIF3I | -0.043217508 | 0.956782492 | -0.815772428 | -0.048930709 | -1.954672972 | 0.141320308193179 |
| EIF3J | -0.031335151 | 0.968664849 | -0.594619129 | -0.021218365 | -2.045612002 | 0.161562534314472 |
| EIF3K | -0.032229205 | 0.967770795 | -0.599447306 | -0.064766154 | -2.325043706 | 0.11946029296065 |
| EIF3L | -0.038461881 | 0.961538119 | -0.637540517 | -0.154544995 | -2.839429474 | 0.0585244565226582 |
| EIF3M | -0.022854389 | 0.977145611 | -0.34290442 | -0.086699594 | -2.90720744 | 0.056017830176136 |
| EIF4A1 | -0.023202743 | 0.976797257 | -0.489186926 | -0.036837997 | -2.012286588 | 0.130705233401285 |
| EIF4A2 | -0.001503727 | 0.998496273 | -0.350315631 | 0.319106416706339 | -0.083154906 | 0.969583819335316 |
| EIF4A3 | -0.019451156 | 0.980548844 | -0.539525236 | 0.124280131741395 | -1.230475946 | 0.359103891620818 |
| EIF4B | -0.05185296 | 0.94814704 | -0.89684627 | -0.221497325 | -2.879783978 | 0.0574056423986682 |
| EIF4E | -0.005868562 | 0.994131438 | -0.579505344 | 0.46393341761299 | -0.191645155 | 0.872650912257848 |
| EIF4E2 | 0.559098407920555 | 0.559098407920555 | -8.366851774 | 14.0511218233229 | 0.441443001614882 | 0.66029657356452 |
| EIF4ENIF1 | -34.63244717 | -33.63244717 | -11.13873303 | 5.81231293000837 | -0.707106781 | 0.539497285386707 |
| EIF4G1 | -0.032742679 | 0.967257321 | -0.679691915 | 0.0110522114917955 | -2.011023434 | 0.1803694486231 |
| EIF4G2 | -0.005547116 | 0.994452884 | -0.339426168 | 0.229679199278476 | -0.335671555 | 0.744149029651997 |
| EIF4G3 | 0.0520549053916896 | 0.0520549053916896 | 0.297934545374227 | 0.666694877386194 | 4.52700549444501 | 0.0162947112456901 |
| EIF4H | -0.001488809 | 0.998511191 | -0.183303986 | 0.152199176270512 | -0.168631331 | 0.896391696905685 |
| EIF5 | -0.038588352 | 0.961411648 | -0.526429084 | -0.26930379 | -6.10861065 | 0.0214618783472991 |
| EIF5A | -0.069458879 | 0.930541121 | -1.102501925 | -0.35342618 | -3.384615941 | 0.0402744596888282 |
| EIF5B | -0.046616437 | 0.953383563 | -0.969104116 | 0.0411491884111115 | -1.866420415 | 0.186653518389882 |
| EIF6 | -0.037807793 | 0.962192207 | -0.621396162 | -0.167105092 | -3.319099248 | 0.0627253866892858 |
| EIPR1 | -0.014695351 | 0.985304649 | -0.510353449 | 0.236798213084994 | -0.799948092 | 0.535425881029537 |
| ELAC2 | -0.080257467 | 0.919742533 | -10.72221642 | 9.73803131696596 | -0.083284535 | 0.969455517331549 |
| ELANE | -0.200919472 | 0.799080528 | -3.085760169 | -1.571603209 | -5.512382953 | 0.0123934097720643 |
| ELAVL1 | -0.027065636 | 0.972934364 | -0.497593553 | -0.08893454 | -2.564883355 | 0.0845612712882046 |
| ELF1 | -0.481186587 | 0.518813413 | -13.56708792 | 6.19245201617447 | -0.839189722 | 0.523341947688131 |
| ELL | -36.46025914 | -35.46025914 | -19.4849968 | 0.576297845928089 | -2.121039605 | 0.1803694486231 |
| ELMO1 | -0.051964128 | 0.948035872 | -1.032097878 | 0.0737236691719438 | -1.695354817 | 0.209406676476696 |
| ELMO2 | 0.0521171436975106 | 0.0521171436975106 | 0.115495742655543 | 0.839704830610544 | 2.41235781449225 | 0.104333150555025 |
| ELMO3 | 0 | 1 | NA | NA | NA | NA |
| ELMOD2 | -0.000153268 | 0.999846732 | -0.292503599 | 0.289513284121402 | -0.010744471 | 0.999130434024431 |
| ELN | -35.19151184 | -34.19151184 | -16.41084835 | 8.56336046488684 | -0.707106781 | 0.539497285386707 |
| ELOA | -0.976630092 | 0.023369908 | -12.80204136 | 7.51833784317673 | -0.451707504 | 0.655753631330814 |
| ELOB | -0.009779787 | 0.990220213 | -0.462334077 | 0.253796365199783 | -0.523384523 | 0.620813677631159 |
| ELOC | 0.000776854114668629 | 0.000776854114668629 | -0.573067207 | 0.587598333177573 | 0.0245979733129519 | 0.999130434024431 |
| ELOVL1 | -1.544477409 | -0.544477409 | -17.88387461 | 5.02148549689335 | -0.971784498 | 0.399778344600192 |
| ELOVL5 | 34.8670710219163 | 34.8670710219163 | -6.83876698 | 13.1058324865178 | 0.707106781186548 | 0.539497285386707 |
| ELP1 | 0.43264632518272 | 0.43264632518272 | -6.903888256 | 13.6974902225453 | 0.741459837550363 | 0.539497285386707 |
| ELP2 | -0.958844933 | 0.041155067 | -18.04034166 | 5.78561573544864 | -1.153560518 | 0.380452616321765 |
| ELP3 | 0.0228277437283266 | 0.0228277437283266 | 0.0214645365742304 | 0.405493581552546 | 2.02194010594819 | 0.148635096810518 |
| ELP4 | -34.73300541 | -33.73300541 | -11.9428188 | 6.23189369497158 | -0.707106781 | 0.539497285386707 |
| EMC1 | -0.048239483 | 0.951760517 | -0.649581503 | -0.330965945 | -6.87698048 | 0.032609142045817 |
| EMC10 | -0.464224356 | 0.535775644 | -13.57569824 | 6.42172059537303 | -0.804819971 | 0.536784365749205 |
| EMC2 | -0.055515787 | 0.944484213 | -0.94794028 | -0.148442269 | -2.710655889 | 0.103361193276205 |
| EMC3 | -0.063544844 | 0.936455156 | -1.130444919 | -0.112397611 | -2.66854016 | 0.131752695143835 |
| EMC4 | -1.60242885 | -0.60242885 | -17.59223655 | 4.60825941361195 | -1.011962575 | 0.381825642867288 |
| EMC6 | -0.623572069 | 0.376427931 | -16.01238427 | 8.91411365597288 | -0.494277745 | 0.634031727574291 |
| EMC7 | -0.025927551 | 0.974072449 | -0.562135014 | 0.072108615262292 | -1.438308539 | 0.267465200468248 |
| EMC8 | -0.025063989 | 0.974936011 | -0.499601721 | 0.00396712242252477 | -1.725300014 | 0.1803694486231 |
| EMD | 0.0042921053034647 | 0.0042921053034647 | -0.187308318 | 0.277615391260742 | 0.345028393308167 | 0.737456893686127 |
| EMG1 | -1.695033722 | -0.695033722 | -17.48401808 | 4.10185140281259 | -1.074249693 | 0.366275438800159 |
| EMILIN1 | 0.101886652919926 | 0.101886652919926 | 0.563610183858795 | 1.70341538700893 | 3.46100971968375 | 0.0383041704369688 |
| EMILIN2 | 0.164639234706999 | 0.164639234706999 | 0.814093759160019 | 2.20389305483457 | 4.02099822835579 | 0.037807978546348 |
| EMILIN3 | 0 | 1 | NA | NA | NA | NA |
| EML1 | 0.0451590790212052 | 0.0451590790212052 | 0.276850777615274 | 0.573221798954477 | 5.62924544363686 | 0.0251233961386856 |
| EML2 | -0.092114886 | 0.907885114 | -1.366631614 | -0.380883668 | -3.636695822 | 0.0679500740251509 |
| EML3 | 0.00486892447748379 | 0.00486892447748379 | -0.272648795 | 0.364231662405456 | 0.273488556927656 | 0.802142144248918 |
| EML4 | 0.0224826227864762 | 0.0224826227864762 | -0.011426512 | 0.478770499297031 | 1.69660248508185 | 0.1803694486231 |
| EML6 | -1.032246411 | -0.032246411 | -14.6346224 | 8.33638255641016 | -0.477546054 | 0.644233855679381 |
| ENAH | 0.0968745653635661 | 0.0968745653635661 | 0.417445408134375 | 1.61044263365962 | 3.76304728285957 | 0.0796176030128218 |
| ENDOD1 | 0.0911250101237758 | 0.0911250101237758 | 0.347118336520761 | 1.41197581190056 | 2.92951607299065 | 0.0609548408040319 |
| ENDOG | 0.0336293121168511 | 0.0336293121168511 | -11.13503688 | 11.5941208099473 | 0.0349516503736704 | 0.997450932115599 |
| ENDOU | 0 | 1 | NA | NA | NA | NA |
| ENG | 0.0263313465939288 | 0.0263313465939288 | -0.398127603 | 0.894976326603656 | 0.701724067466896 | 0.539497285386707 |
| ENGASE | -0.422910492 | 0.577089508 | -13.68064722 | 7.02797122796881 | -0.722272684 | 0.539497285386707 |
| ENO1 | -0.00208573 | 0.99791427 | -0.237684042 | 0.188503005848202 | -0.203508195 | 0.862599301392591 |
| ENO2 | 0.00386856661600128 | 0.00386856661600128 | -0.518313913 | 0.59447847385078 | 0.140633532267644 | 0.922676528177908 |
| ENO3 | -0.121245616 | 0.878754384 | -12.58713195 | 10.9024724503961 | -0.124203289 | 0.933451345791332 |
| ENOPH1 | -0.013018532 | 0.986981468 | -0.40901712 | 0.171482565566392 | -0.746346776 | 0.5377743564556 |
| ENOSF1 | -34.84848592 | -33.84848592 | -12.93808319 | 6.7512335573924 | -0.707106781 | 0.539497285386707 |
| ENPEP | 0.0765732783761181 | 0.0765732783761181 | 0.129566757221714 | 1.39167040648162 | 2.08976063164337 | 0.121338600562669 |
| ENPP1 | 1.05138990142101 | 1.05138990142101 | -8.665698507 | 15.3698054860148 | 0.486317972617299 | 0.638818830709466 |
| ENPP4 | -0.044432068 | 0.955567932 | -0.908676836 | 0.146838788848643 | -1.290179124 | 0.309260370229649 |
| ENSA | -0.004725882 | 0.995274118 | -0.646748257 | 0.557125430934114 | -0.13050685 | 0.928328518499981 |
| ENTPD1 | 0.0121291565875474 | 0.0121291565875474 | -0.395484474 | 0.659118912729635 | 0.442984822965132 | 0.66029657356452 |
| ENTPD3 | 1.14438885408656 | 1.14438885408656 | -3.763512465 | 18.2979789612712 | 1.48229025076546 | 0.313713786103829 |
| ENTPD4 | -36.9306367 | -35.9306367 | -13.47087584 | -12.72659715 | -79.2081562 | 0.00023176824691923 |
| ENY2 | 0.0208357791867831 | 0.0208357791867831 | -11.01185047 | 11.2908572451358 | 0.0216460357883409 | 0.999130434024431 |
| EOGT | -0.034432679 | 0.965567321 | -0.766434235 | 0.121724647367748 | -1.477338981 | 0.284878956001337 |
| EP400 | 0.00613814412665911 | 0.00613814412665911 | -0.808488059 | 0.915854548277502 | 0.117167433834172 | 0.94126289739034 |
| EPB41 | -0.006649635 | 0.993350365 | -0.977890738 | 0.842440052956743 | -0.128938801 | 0.929355021297957 |
| EPB41L1 | -0.427656927 | 0.572343073 | -13.85355852 | 7.0562620889229 | -0.731082807 | 0.539497285386707 |
| EPB41L2 | 0.0845852727676667 | 0.0845852727676667 | 0.600683471314597 | 1.19808129343584 | 5.21702999762383 | 0.010031921642431 |
| EPB41L3 | 0.0456631213885975 | 0.0456631213885975 | -0.181358392 | 1.06225333395324 | 1.22608283954954 | 0.325280869222481 |
| EPB42 | 0.0287331243400213 | 0.0287331243400213 | -0.719532713 | 1.3621826201363 | 0.535201275238364 | 0.61076113623048 |
| EPCAM | 0 | 1 | NA | NA | NA | NA |
| EPDR1 | 0.0592239321826707 | 0.0592239321826707 | -0.222025625 | 1.36162122604821 | 1.421990931 | 0.294403235671687 |
| EPHA2 | 36.9859512611374 | 36.9859512611374 | 13.3072379944681 | 13.9141799424358 | 100.927537299469 | 0.000182450656380933 |
| EPHA4 | 36.8924261193744 | 36.8924261193744 | 12.3799571666561 | 13.1327691592087 | 76.2634465909967 | 0.000243867488171237 |
| EPHA7 | 0 | 1 | NA | NA | NA | NA |
| EPHB2 | 0.65841616761058 | 0.65841616761058 | -7.691397653 | 14.3403449404444 | 0.523261275297014 | 0.617640756478743 |
| EPHB3 | 36.7774200326753 | 36.7774200326753 | 11.1919578616977 | 12.3659429355877 | 45.1564820364269 | 0.000541280339075164 |
| EPHB4 | 36.435472049561 | 36.435472049561 | -0.566878849 | 19.1534803945736 | 2.12095402036223 | 0.1803694486231 |
| EPHX1 | 0.0357404931963058 | 0.0357404931963058 | -0.061436389 | 0.84738152567564 | 1.65607970764241 | 0.211683558741771 |
| EPHX2 | 0.987932840915625 | 0.987932840915625 | -5.520089929 | 18.1960817479097 | 1.20108694747725 | 0.367529743405878 |
| EPM2A | 0 | 1 | NA | NA | NA | NA |
| EPM2AIP1 | -0.373534373 | 0.626465627 | -13.72810822 | 7.74409043200415 | -0.626648412 | 0.577777235685786 |
| EPN1 | -0.006030021 | 0.993969979 | -0.420263122 | 0.304758629259627 | -0.295193904 | 0.782037498628257 |
| EPN2 | 1.58002245363941 | 1.58002245363941 | -4.353254887 | 16.1156422964921 | 0.99427041172813 | 0.389202761398707 |
| EPN3 | 0.0830931659563713 | 0.0830931659563713 | -9.037158511 | 9.34261482171724 | 0.0287780146041735 | 0.998435102357591 |
| EPPK1 | 0.903817044063668 | 0.903817044063668 | -6.336145735 | 17.8489082057034 | 1.06317043020908 | 0.411849317348522 |
| EPRS1 | -0.026714775 | 0.973285225 | -0.745235378 | 0.182755239339086 | -1.29495682 | 0.357254797084829 |
| EPS15 | -0.002598185 | 0.997401815 | -0.341832765 | 0.290756665513442 | -0.163653304 | 0.90292026073532 |
| EPS15L1 | 0.0293980798721794 | 0.0293980798721794 | -0.037102345 | 0.609499006812887 | 1.96673279441617 | 0.195631715815276 |
| EPS8 | -0.013350382 | 0.986649618 | -0.697233394 | 0.463255905304845 | -0.35878772 | 0.725368063426545 |
| EPS8L1 | 0 | 1 | NA | NA | NA | NA |
| EPS8L2 | 0.549879664417435 | 0.549879664417435 | -9.362982099 | 15.5722538329169 | 0.43379348912652 | 0.664644836948523 |
| EPSTI1 | -34.64556721 | -33.64556721 | -11.24049197 | 5.86541186198874 | -0.707106781 | 0.539497285386707 |
| EPX | 0.00204037347347859 | 0.00204037347347859 | -0.731623748 | 0.76993796917846 | 0.044170304205959 | 0.993220185640509 |
| ERAP1 | -0.024947864 | 0.975052136 | -0.474730557 | -0.047887492 | -2.197296804 | 0.119866482067661 |
| ERAP2 | 0.0248607096244536 | 0.0248607096244536 | -11.89233909 | 12.2524358584974 | 0.0258069631156077 | 0.998861061793165 |
| ERBB2 | 0 | 1 | NA | NA | NA | NA |
| ERBIN | -0.006284832 | 0.993715168 | -0.199715468 | 0.0879850761901106 | -0.680339849 | 0.539497285386707 |
| ERC1 | 0.0669232934528551 | 0.0669232934528551 | 0.20913665452312 | 1.00058584669945 | 2.77860042759262 | 0.0761918355955649 |
| ERCC2 | -0.05292593 | 0.94707407 | -1.485742661 | 0.543304939274696 | -1.00854578 | 0.43191166093357 |
| ERCC3 | -0.37901931 | 0.62098069 | -13.37890998 | 7.47092701021648 | -0.636969198 | 0.57180551099007 |
| ERCC4 | -0.568843888 | 0.431156112 | -13.52088525 | 7.97159293785746 | -0.449256016 | 0.656682440358441 |
| ERG28 | -0.46333762 | 0.53666238 | -15.21137798 | 7.20715443464647 | -0.803339329 | 0.537046163482827 |
| ERGIC1 | -0.034979039 | 0.965020961 | -0.808903341 | 0.0676359153170634 | -1.489332844 | 0.228718315617715 |
| ERGIC2 | -0.511103144 | 0.488896856 | -15.38530311 | 6.5908423923842 | -0.899883638 | 0.49365039845455 |
| ERGIC3 | -0.109356817 | 0.890643183 | -1.275642269 | -0.921492695 | -10.99141452 | 0.000583895136039144 |
| ERH | -0.030929303 | 0.969070697 | -0.777049807 | 0.193404733945237 | -1.286273555 | 0.360193338177673 |
| ERI3 | 0.0125596866085985 | 0.0125596866085985 | -0.182744821 | 0.427715341456871 | 0.742379555851733 | 0.539497285386707 |
| ERLEC1 | -0.109732665 | 0.890267335 | -1.479597856 | -0.599743332 | -4.429566588 | 0.0324314269886733 |
| ERLIN1 | 0.0703159696461637 | 0.0703159696461637 | 0.3577495661648 | 0.894238461976449 | 4.17047853195234 | 0.0279763107606235 |
| ERLIN2 | 0.081087618905432 | 0.081087618905432 | 0.359680685693837 | 1.26346015524585 | 3.21813946675438 | 0.0525595373037303 |
| ERMAP | 0.0298821539140572 | 0.0298821539140572 | -10.96326948 | 11.0952764759914 | 0.0103554398504319 | 0.999130434024431 |
| ERMP1 | 0.022246851869322 | 0.022246851869322 | -0.183199301 | 0.615650429337766 | 0.960049363148539 | 0.411335306976434 |
| ERO1A | -0.085675798 | 0.914324202 | -1.421440607 | -0.318441623 | -3.502066644 | 0.0879091406932419 |
| ERO1B | -1.225824983 | -0.225824983 | -17.03086303 | 6.87274623329181 | -0.74575508 | 0.534281958550569 |
| ERP29 | -0.073034243 | 0.926965757 | -1.0600747 | -0.43312013 | -4.130636542 | 0.0226234395622484 |
| ERP44 | -0.019256026 | 0.980743974 | -0.456614215 | 0.028900644356103 | -1.646005377 | 0.206184342487605 |
| ESAM | -0.029590417 | 0.970409583 | -14.37949802 | 14.0875199829952 | -0.017748106 | 0.999130434024431 |
| ESD | 0.0565536984996058 | 0.0565536984996058 | 0.125530279129601 | 1.11530198028159 | 2.5086488870388 | 0.121651765302156 |
| ESRP1 | -34.76417168 | -33.76417168 | -12.20362415 | 6.36798478562352 | -0.707106781 | 0.539497285386707 |
| ESRP2 | 1.00365128524533 | 1.00365128524533 | -5.609937751 | 19.1652600457216 | 1.23072575994079 | 0.366275438800159 |
| ESRRA | -34.56958097 | -33.56958097 | -10.66378014 | 5.56447730989912 | -0.707106781 | 0.539497285386707 |
| ESYT1 | -0.001227889 | 0.998772111 | -0.24016008 | 0.214085442629379 | -0.115429954 | 0.943295035661147 |
| ESYT2 | 0.0435383445924734 | 0.0435383445924734 | 0.13415466994019 | 0.750501888595363 | 2.52730539579244 | 0.0838488333459855 |
| ETF1 | -0.03413752 | 0.96586248 | -0.53547054 | -0.140648592 | -3.441676628 | 0.0702796333134765 |
| ETFA | -0.043090045 | 0.956909955 | -0.626837416 | -0.295270251 | -5.394033499 | 0.0256475213740026 |
| ETFB | -0.043776898 | 0.956223102 | -0.686839313 | -0.305441693 | -5.348816074 | 0.0353501415719168 |
| ETFDH | -0.015897018 | 0.984102982 | -0.645044191 | 0.342935010536105 | -0.644801229 | 0.562031639448264 |
| ETFRF1 | -0.984469311 | 0.015530689 | -13.69781216 | 8.01295585159972 | -0.455061401 | 0.654500626880749 |
| ETHE1 | -0.019052772 | 0.980947228 | -0.532253453 | 0.121993899452389 | -1.306738244 | 0.349220975890688 |
| ETS1 | -34.872702 | -33.872702 | -13.15708581 | 6.86551152479197 | -0.707106781 | 0.539497285386707 |
| ETV6 | -2.010080081 | -1.010080081 | -20.68978759 | 0.511717527204017 | -2.140715588 | 0.1803694486231 |
| EVA1B | -35.73575519 | -34.73575519 | -16.23580038 | 4.79215939093074 | -1.224653348 | 0.366275438800159 |
| EVI2B | -0.579025888 | 0.420974112 | -13.82195901 | 5.03953717953656 | -1.047140886 | 0.421362924942437 |
| EVL | -0.092385802 | 0.907614198 | -1.701985713 | -0.00166212 | -1.984161383 | 0.179117559639718 |
| EVPL | 0.0404974733703015 | 0.0404974733703015 | -13.60631128 | 13.9934905634968 | 0.0242773439689067 | 0.999130434024431 |
| EWSR1 | -0.020633469 | 0.979366531 | -0.654399531 | 0.229781976893707 | -0.831548127 | 0.484539614595117 |
| EXD2 | -0.441724133 | 0.558275867 | -13.26701111 | 6.5669945299916 | -0.759604948 | 0.539497285386707 |
| EXOC1 | 0.0420733746292362 | 0.0420733746292362 | -0.023257025 | 0.781229482598257 | 1.6309711253153 | 0.182746044686501 |
| EXOC2 | 0.0475393716173125 | 0.0475393716173125 | 0.148815276231836 | 0.725320992728645 | 2.90886709851819 | 0.0829291783160025 |
| EXOC3 | 0.0390506389224451 | 0.0390506389224451 | 0.122437863151168 | 0.627396255552268 | 2.82001531258423 | 0.0845612712882046 |
| EXOC4 | 0.0277397320286244 | 0.0277397320286244 | -0.066732117 | 0.600151609643742 | 1.67142571827866 | 0.237330029143195 |
| EXOC5 | 0.0538956360764014 | 0.0538956360764014 | 0.238880726584448 | 0.760517208362998 | 3.41043261673785 | 0.0441140669696835 |
| EXOC6B | 0.125856249743011 | 0.125856249743011 | 0.421581169696787 | 1.7137132829025 | 3.11164091468885 | 0.0684325982231265 |
| EXOC7 | 0.052600309706815 | 0.052600309706815 | 0.322210818662611 | 0.667414834624449 | 4.97461316281128 | 0.0122496344082122 |
| EXOC8 | 0.0452437864974856 | 0.0452437864974856 | -0.066783639 | 0.91154169056813 | 1.62160339421622 | 0.213937911794673 |
| EXOG | 0.0392591212202408 | 0.0392591212202408 | -0.177571922 | 0.847598040999643 | 1.19038225794114 | 0.354826113544616 |
| EXOSC1 | -1.069632593 | -0.069632593 | -17.3230451 | 4.37043786734323 | -1.342833656 | 0.354826113544616 |
| EXOSC10 | -0.472582252 | 0.527417748 | -13.63267024 | 6.33540449902588 | -0.821564434 | 0.530560205205794 |
| EXOSC2 | -0.060447459 | 0.939552541 | -1.057058981 | -0.046215907 | -2.273610302 | 0.159395442620735 |
| EXOSC3 | 0 | 1 | NA | NA | NA | NA |
| EXOSC4 | -0.427576435 | 0.572423565 | -15.23427655 | 7.75839412168433 | -0.731312856 | 0.539497285386707 |
| EXOSC5 | -34.8189352 | -33.8189352 | -12.67576814 | 6.61435469269802 | -0.707106781 | 0.539497285386707 |
| EXOSC6 | -0.072117824 | 0.927882176 | -1.270034794 | -0.084635038 | -2.204398091 | 0.142706257318893 |
| EXOSC7 | -0.042247774 | 0.957752226 | -10.86500198 | 10.3276971503418 | -0.043875729 | 0.993356560314692 |
| EXOSC8 | -2.061613882 | -1.061613882 | -20.48056779 | 0.0232852823188763 | -2.244346513 | 0.179533257798002 |
| EXOSC9 | -0.011912417 | 0.988087583 | -0.45834724 | 0.241979548223129 | -0.575925152 | 0.590284403565099 |
| EZR | -0.090810357 | 0.909189643 | -1.245627501 | -0.670690482 | -5.785472284 | 0.00710261704363371 |
| F10 | 0.0372932493349493 | 0.0372932493349493 | -0.282130089 | 1.00612129770061 | 1.15581446161587 | 0.367224657481449 |
| F11 | 1.09148120280547 | 1.09148120280547 | -4.472053095 | 18.7818797118674 | 1.38467105366664 | 0.343931617922055 |
| F11R | -0.074958413 | 0.925041587 | -1.018488832 | -0.407745448 | -4.252653737 | 0.0299294879224496 |
| F12 | 0.134736577891167 | 0.134736577891167 | 0.367957870842269 | 2.41852462317118 | 2.36413509206707 | 0.0924871407422864 |
| F13A1 | 0.0152720733372946 | 0.0152720733372946 | -0.342408671 | 0.686448792964988 | 0.582715675148956 | 0.57827242635708 |
| F13B | 2.08206058845832 | 2.08206058845832 | 0.167599708717732 | 18.8862009002804 | 2.28386566310013 | 0.175707003568027 |
| F2 | 0.035306538797389 | 0.035306538797389 | 0.0145072904983114 | 0.757406911768293 | 1.80314087060031 | 0.169333108591434 |
| F3 | 0 | 1 | NA | NA | NA | NA |
| F5 | 0.458590132879956 | 0.458590132879956 | -6.690740475 | 13.9664101449217 | 0.792548594034858 | 0.539497285386707 |
| F7 | 1.57633024551842 | 1.57633024551842 | -4.472874904 | 16.5394448864264 | 0.993610046956072 | 0.389405986790154 |
| F8A3 | -35.54768044 | -34.54768044 | -14.25441923 | 4.20946216945317 | -1.22425298 | 0.366275438800159 |
| F9 | 0.0360620980444799 | 0.0360620980444799 | -0.093991351 | 0.784732219553969 | 1.38388165689357 | 0.266139820992796 |
| FAAH | 35.7616910686522 | 35.7616910686522 | -4.895115678 | 16.5463437328366 | 1.22282257505534 | 0.366275438800159 |
| FABP1 | 34.9355869605018 | 34.9355869605018 | -7.171386987 | 13.7432664131624 | 0.707106781186548 | 0.539497285386707 |
| FABP3 | -0.017667158 | 0.982332842 | -1.765092916 | 1.40122443766197 | -0.219440265 | 0.850970260062849 |
| FABP4 | 0.467455807752698 | 0.467455807752698 | -7.55456286 | 16.0972354664606 | 0.80920397916025 | 0.534470832811491 |
| FABP5 | -0.040980841 | 0.959019159 | -1.666288704 | 0.749760034567578 | -0.773427176 | 0.538122477278722 |
| FABP7 | 0 | 1 | NA | NA | NA | NA |
| FABP9 | 0 | 1 | NA | NA | NA | NA |
| FADD | -35.90763335 | -34.90763335 | -18.28917453 | 5.39763729696446 | -1.224740796 | 0.366275438800159 |
| FADS2 | 0.902998788865283 | 0.902998788865283 | -8.193538118 | 13.3929628869155 | 0.417385816237929 | 0.67602315637146 |
| FAF1 | -0.013377486 | 0.986622514 | -0.322805406 | 0.068801560536692 | -1.122688567 | 0.364688913286397 |
| FAF2 | -0.010629723 | 0.989370277 | -0.288780054 | 0.0699631557907663 | -1.081127758 | 0.366275438800159 |
| FAH | -0.026231288 | 0.973768712 | -0.43623118 | -0.079198803 | -2.582323675 | 0.0841851891763818 |
| FAHD1 | 0.0112861140942717 | 0.0112861140942717 | -0.262621995 | 0.468209996566143 | 0.592522817477253 | 0.591508080590907 |
| FAHD2A | -0.051174348 | 0.948825652 | -0.762858974 | -0.217753406 | -3.14775487 | 0.049204903310836 |
| FAM107B | -1.094444782 | -0.094444782 | -17.33360349 | 4.09491413290955 | -1.388838434 | 0.342589676346385 |
| FAM114A1 | 0.466073633477022 | 0.466073633477022 | -7.142687212 | 15.1599452531676 | 0.808848050752161 | 0.535226954164563 |
| FAM114A2 | -0.03576427 | 0.96423573 | -0.828016461 | 0.14595543377521 | -1.28578004 | 0.320042285685488 |
| FAM120A | 0.0035219618292347 | 0.0035219618292347 | -0.143165345 | 0.211978414745448 | 0.338847877398883 | 0.741612098703883 |
| FAM120B | -0.014195862 | 0.985804138 | -0.622881895 | 0.383226074631855 | -0.42087558 | 0.674602519987979 |
| FAM120C | 0.460192620461766 | 0.460192620461766 | -6.493301617 | 13.5796893453963 | 0.792484756681447 | 0.539497285386707 |
| FAM126A | 0.0481914716254827 | 0.0481914716254827 | -0.137941992 | 1.00226117450428 | 1.50501730746347 | 0.267874728789652 |
| FAM136A | -0.429795643 | 0.570204357 | -14.06608087 | 7.12597719431014 | -0.735557662 | 0.539497285386707 |
| FAM162A | 0.00132286263751335 | 0.00132286263751335 | -0.687298302 | 0.713023297118713 | 0.0398819628384837 | 0.996073677353712 |
| FAM169A | 1.6605334753536 | 1.6605334753536 | -4.028269122 | 16.4866467619429 | 1.05146398515095 | 0.367529743405878 |
| FAM174A | -0.063611943 | 0.936388057 | -0.894279468 | -0.320016863 | -4.28432628 | 0.0489656305565222 |
| FAM177A1 | -0.394963922 | 0.605036078 | -13.07641908 | 7.08803643336527 | -0.664681566 | 0.558631800775978 |
| FAM180B | 0 | 1 | NA | NA | NA | NA |
| FAM210B | 0.96268085749456 | 0.96268085749456 | -7.804505478 | 13.1960673721038 | 0.445701037491154 | 0.657887841997856 |
| FAM234A | 0.00639370081346357 | 0.00639370081346357 | -12.26322709 | 12.3177259511839 | 0.00383614312008419 | 0.999130434024431 |
| FAM240C | 0 | 1 | NA | NA | NA | NA |
| FAM3C | -0.053956584 | 0.946043416 | -1.144168293 | 0.0808681997032849 | -1.885109467 | 0.203561147190231 |
| FAM50A | -0.837641134 | 0.162358866 | -14.520292 | 9.20928664465496 | -0.387341142 | 0.698637708927531 |
| FAM83A | 0 | 1 | NA | NA | NA | NA |
| FAM83B | 0 | 1 | NA | NA | NA | NA |
| FAM83C | 0 | 1 | NA | NA | NA | NA |
| FAM83G | 34.806999100317 | 34.806999100317 | -6.559856752 | 12.5713281335252 | 0.707106781186547 | 0.539497285386707 |
| FAM83H | -0.628120767 | 0.371879233 | -15.94214341 | 8.86175779579387 | -0.495688971 | 0.633636391695793 |
| FAM8A1 | 0.0301632133700411 | 0.0301632133700411 | -0.133129741 | 0.684939352741709 | 1.17929622003282 | 0.349003315577836 |
| FAM91A1 | -0.455524132 | 0.544475868 | -12.83170487 | 6.17402231154589 | -0.786103813 | 0.539497285386707 |
| FAM98A | 0.0131394365210662 | 0.0131394365210662 | -0.309957617 | 0.557360275620387 | 0.502533905182767 | 0.630816747271883 |
| FAM98B | -0.030604884 | 0.969395116 | -0.64932634 | 0.0559233428433846 | -1.702743692 | 0.21933344441598 |
| FAM98C | 35.875513901273 | 35.875513901273 | -5.279282251 | 17.8869800317036 | 1.2246892560103 | 0.366275438800159 |
| FAP | 0.00861717168760933 | 0.00861717168760933 | -0.509162559 | 0.67411956172765 | 0.251365708651102 | 0.819259951808132 |
| FAR2 | -34.93648505 | -33.93648505 | -13.75182442 | 7.17585264580178 | -0.707106781 | 0.539497285386707 |
| FARP1 | 0.106975830905779 | 0.106975830905779 | 0.633476843146676 | 1.43274128057148 | 4.60511936756343 | 0.0203094444224222 |
| FARS2 | -0.516014468 | 0.483985532 | -13.14073525 | 5.47354179351689 | -0.899469653 | 0.490002201456186 |
| FARSA | -0.056406499 | 0.943593501 | -0.884893724 | -0.18756759 | -2.882005841 | 0.0783502795687516 |
| FARSB | -0.051292653 | 0.948707347 | -0.753204496 | -0.279728935 | -4.049162006 | 0.037807978546348 |
| FAS | -0.397561152 | 0.602438848 | -12.12134952 | 6.52931141239215 | -0.671395841 | 0.555441009809081 |
| FASN | 7.8284717684342e-05 | 7.8284717684342e-06 | -0.263436515 | 0.264976729573859 | 0.00523106641667278 | 0.999130434024431 |
| FASTKD2 | 0 | 1 | NA | NA | NA | NA |
| FAT4 | 2.14877683746083 | 2.14877683746083 | 0.758102356275893 | 19.7500271488391 | 2.42744869663431 | 0.164111864996312 |
| FAU | -0.062262697 | 0.937737303 | -1.051774884 | -0.341671998 | -3.543793174 | 0.04345047369545 |
| FBL | -0.05374518 | 0.94625482 | -0.944490288 | -0.23524602 | -3.160028504 | 0.0684325982231265 |
| FBLIM1 | 0.054225836405755 | 0.054225836405755 | 0.143197734418723 | 0.934872357999414 | 2.7714431110514 | 0.106714207935712 |
| FBLL1 | 35.9204714188177 | 35.9204714188177 | -5.446656131 | 18.4534228504213 | 1.22466160439007 | 0.366275438800159 |
| FBLN1 | 0.15067091706306 | 0.15067091706306 | 1.3375185125268 | 2.20680315957247 | 7.3636098 | 0.00505975111662158 |
| FBLN2 | 0.0399034445095817 | 0.0399034445095817 | -0.385560369 | 1.265544038 | 0.973498933894362 | 0.411215167582234 |
| FBLN5 | 0.106073133901873 | 0.106073133901873 | 0.118287469114565 | 2.07297824550728 | 2.0504434202892 | 0.145604600156276 |
| FBN1 | 0.129897656886609 | 0.129897656886609 | 0.805343401952834 | 2.36199410847008 | 3.85918418423273 | 0.0453049930855873 |
| FBN2 | 0.604304468844526 | 0.604304468844526 | -5.155700776 | 15.2980969945347 | 1.0933017269152 | 0.39736509500738 |
| FBP1 | -0.04050303 | 0.95949697 | -0.666323758 | -0.140317361 | -2.937528343 | 0.0810869445560843 |
| FBP2 | -36.96304603 | -35.96304603 | -13.76379949 | -13.02884496 | -82.0353807 | 0.000220658446544722 |
| FBXL18 | 0.719400054541945 | 0.719400054541945 | -7.032459782 | 13.9867219462916 | 0.572842622971468 | 0.584631415539537 |
| FBXL8 | 0.374274957756132 | 0.374274957756132 | -8.093731625 | 14.3668159539586 | 0.627738688128417 | 0.576973974932153 |
| FBXO2 | 0.119163730020412 | 0.119163730020412 | -10.73127603 | 12.3805914008371 | 0.123678109621521 | 0.933909495003884 |
| FBXO22 | 0.0154950908361642 | 0.0154950908361642 | -0.197183149 | 0.486077883247199 | 0.747058996015335 | 0.534470832811491 |
| FBXO3 | 0.440739070473629 | 0.440739070473629 | -6.469204581 | 13.0520965308567 | 0.756861347153391 | 0.539497285386707 |
| FBXO30 | 0.623300435040131 | 0.623300435040131 | -8.42498881 | 15.1300157037874 | 0.494106249731449 | 0.634031727574291 |
| FBXO45 | -34.73530666 | -33.73530666 | -11.96188402 | 6.24184213772464 | -0.707106781 | 0.539497285386707 |
| FBXO6 | -34.81243127 | -33.81243127 | -12.61875207 | 6.58460308286014 | -0.707106781 | 0.539497285386707 |
| FBXO7 | 0.0262139060210918 | 0.0262139060210918 | -0.489347933 | 1.00065862882757 | 0.594222185294242 | 0.570914625414931 |
| FBXW11 | -0.682034003 | 0.317965997 | -13.8402487 | 7.24150041522563 | -0.542283192 | 0.605320766728735 |
| FCER1G | -0.492866706 | 0.507133294 | -15.27185947 | 6.77695144216133 | -0.860197777 | 0.512154775182943 |
| FCF1 | -0.542638562 | 0.457361438 | -15.00578899 | 9.0889212673475 | -0.427927085 | 0.668270163327605 |
| FCGBP | 0.0396459959788477 | 0.0396459959788477 | -0.238275178 | 0.990649900338166 | 1.06044012475923 | 0.366735091564231 |
| FCGR1A | -0.103813812 | 0.896186188 | -2.292737051 | 0.342322413179661 | -1.331389169 | 0.295113106149497 |
| FCGR2B | -37.18440473 | -36.18440473 | -16.0884245 | -15.14727181 | -74.68565438 | 0.000243867488171237 |
| FCGR3A | -35.90306472 | -34.90306472 | -18.23167056 | 5.38089276678409 | -1.224707466 | 0.366275438800159 |
| FCGRT | 0.0593454110682445 | 0.0593454110682445 | 0.285759367243496 | 0.848070867298756 | 4.19848840945087 | 0.0549675445616915 |
| FCHO2 | -1.577260997 | -0.577260997 | -18.39384107 | 4.9706692091562 | -0.994039297 | 0.389202761398707 |
| FCHSD1 | -34.68260392 | -33.68260392 | -11.53279261 | 6.01793753825563 | -0.707106781 | 0.539497285386707 |
| FCN1 | -36.90931056 | -35.90931056 | -13.31514629 | -12.49791984 | -71.07932052 | 0.000259029764524189 |
| FCN3 | 0.0440613313113313 | 0.0440613313113313 | -0.73992829 | 1.59458565343489 | 0.794034522608785 | 0.537046163482827 |
| FCRL5 | -36.344227 | -35.344227 | -17.99991637 | 0.552445098083826 | -2.116310475 | 0.1803694486231 |
| FCSK | 0.458201995625154 | 0.458201995625154 | -6.32121976 | 13.2368932936723 | 0.789649507873764 | 0.539497285386707 |
| FDCSP | 0.0070683197524556 | 0.0070683197524556 | -1.097042822 | 1.26224869209947 | 0.128434580336531 | 0.930694298204222 |
| FDFT1 | 0.0413772939124375 | 0.0413772939124375 | -9.954423046 | 10.1207461069607 | 0.0143378204311383 | 0.999130434024431 |
| FDPS | -0.027722029 | 0.972277971 | -1.103305483 | 0.562133621989761 | -0.631821909 | 0.561683424906805 |
| FDX1 | -0.470016798 | 0.529983202 | -15.54389079 | 7.27248512539871 | -0.814763717 | 0.532766709927747 |
| FDXR | 0.00622669076403717 | 0.00622669076403717 | -0.309022649 | 0.435748885526126 | 0.294590493576224 | 0.779722152552842 |
| FECH | -0.011076071 | 0.988923929 | -0.375007015 | 0.17105014803058 | -0.646694864 | 0.543036874716367 |
| FEN1 | -0.103279041 | 0.896720959 | -1.540131908 | -0.53455322 | -4.374913156 | 0.0548717713947671 |
| FERMT1 | 0 | 1 | NA | NA | NA | NA |
| FERMT2 | 0.0909817366030383 | 0.0909817366030383 | 0.467458868597272 | 1.2902506175322 | 3.93770428792799 | 0.0385666087989108 |
| FERMT3 | -0.066464018 | 0.933535982 | -1.16624765 | -0.251137481 | -2.800712352 | 0.0735932337194676 |
| FES | -0.040076855 | 0.959923145 | -11.61098685 | 11.0661998359684 | -0.041572301 | 0.994613821823136 |
| FETUB | 0.63890795827635 | 0.63890795827635 | -8.05541318 | 14.7113564779006 | 0.507199589704923 | 0.627296062254955 |
| FGA | 0.0204101032237072 | 0.0204101032237072 | -0.582321088 | 1.08387583203033 | 0.562056531144096 | 0.599312548531877 |
| FGB | 0.0543521089091866 | 0.0543521089091866 | -0.118684977 | 1.4988374288633 | 1.49117595097933 | 0.225026215650574 |
| FGD3 | 0.0474401687455627 | 0.0474401687455627 | 0.145003695600613 | 0.727731248481701 | 2.657060778 | 0.0768138743236135 |
| FGFBP1 | 0 | 1 | NA | NA | NA | NA |
| FGFR1OP2 | -36.24710942 | -35.24710942 | -16.82793013 | 0.516306707811748 | -2.116352855 | 0.1803694486231 |
| FGG | 0.0746507364210422 | 0.0746507364210422 | 0.23792882113132 | 1.56881871834532 | 2.48536874667235 | 0.0985312713076194 |
| FGL2 | 0.660595009751318 | 0.660595009751318 | -7.809465883 | 14.5995994246402 | 0.525329056044932 | 0.616550083670876 |
| FGR | -2.214693386 | -1.214693386 | -18.02153783 | -1.215455077 | -2.574634478 | 0.149053627887546 |
| FH | -0.042061548 | 0.957938452 | -0.638952845 | -0.240387274 | -3.827583468 | 0.0286320824170219 |
| FHIP2A | 0.0173395732967528 | 0.0173395732967528 | -0.190385635 | 0.512141493440234 | 0.859823300908604 | 0.48376182222999 |
| FHIT | -0.017863642 | 0.982136358 | -0.460337492 | 0.141986394785382 | -0.971990977 | 0.413255578212935 |
| FHL1 | 0.124047582392419 | 0.124047582392419 | 0.761864683148237 | 1.78065178118013 | 4.63206681811906 | 0.0277616496067295 |
| FHL2 | 0.0877479861102027 | 0.0877479861102027 | 0.52320768075391 | 1.27752235640489 | 4.85197768787603 | 0.0400336586898355 |
| FHL3 | 0.0762640541730158 | 0.0762640541730158 | 0.145127113649881 | 1.37613194581641 | 2.71282303748659 | 0.129697093168504 |
| FHOD1 | 0.0480441779043986 | 0.0480441779043986 | 0.149072364378603 | 0.724864855123334 | 2.72184572495985 | 0.0759721863380357 |
| FIBP | 0.00309085816480126 | 0.00309085816480126 | -10.34930579 | 10.3878152138076 | 0.0032130855300468 | 0.999376962148702 |
| FILIP1L | -0.024748884 | 0.975251116 | -10.78142756 | 10.4668599479128 | -0.025618968 | 0.998861061793165 |
| FIP1L1 | -0.063277847 | 0.936722153 | -0.913517354 | -0.221346177 | -2.97514343 | 0.0648842992292444 |
| FIS1 | -0.031956794 | 0.968043206 | -0.670142499 | 0.0491037487635159 | -1.754884777 | 0.208859935161997 |
| FITM1 | 0 | 1 | NA | NA | NA | NA |
| FKBP10 | 0.116106406499929 | 0.116106406499929 | 0.506861313408661 | 2.05449049126465 | 3.46958472195779 | 0.0783538412934913 |
| FKBP11 | -0.111931553 | 0.888068447 | -1.660115407 | -0.911603626 | -6.297589356 | 0.0102762020199873 |
| FKBP14 | 0.500150774488202 | 0.500150774488202 | -6.284446021 | 14.3276715358978 | 0.878077955840252 | 0.505407233967041 |
| FKBP15 | -0.020703677 | 0.979296323 | -0.637011743 | 0.268686078435956 | -0.710773696 | 0.539497285386707 |
| FKBP1A | 0.00389538878533472 | 0.00389538878533472 | -0.38009323 | 0.462534070380179 | 0.171768792612366 | 0.893039998536056 |
| FKBP2 | -0.119997514 | 0.880002486 | -1.683053814 | -0.926104048 | -6.071970029 | 0.00721761206846586 |
| FKBP3 | -0.043809989 | 0.956190011 | -0.586233386 | -0.324415528 | -6.847916368 | 0.0159729447348982 |
| FKBP4 | 0.0147268687532199 | 0.0147268687532199 | -0.344711518 | 0.634157346690132 | 0.655450077157169 | 0.561545253145876 |
| FKBP5 | -0.0233868 | 0.9766132 | -0.540731456 | 0.0965605183750324 | -1.231111356 | 0.330128930179926 |
| FKBP7 | 0.121721972288784 | 0.121721972288784 | 0.425605369875674 | 1.932922696 | 2.87846640233852 | 0.0736890301461125 |
| FKBP8 | -0.053820535 | 0.946179465 | -1.060895637 | 0.0333919409780907 | -1.645746298 | 0.183650685850336 |
| FKBP9 | 0.107743915543058 | 0.107743915543058 | 0.5915400133454 | 1.81691824759484 | 3.99722304227115 | 0.0551284097584765 |
| FLAD1 | -0.003069825 | 0.996930175 | -0.709656004 | 0.655844092448069 | -0.077418169 | 0.973968913166085 |
| FLG | 34.7907517664987 | 34.7907517664987 | -6.486395429 | 12.430546644408 | 0.707106781186547 | 0.539497285386707 |
| FLG2 | 1.08482203835043 | 1.08482203835043 | -8.004575465 | 14.4483002627555 | 0.501406240639432 | 0.630816747271883 |
| FLII | -0.006160838 | 0.993839162 | -0.266493603 | 0.150189947969597 | -0.549870848 | 0.612278741072293 |
| FLNA | 0.0725745454745109 | 0.0725745454745109 | 0.31093631248569 | 1.45925969508065 | 3.07651680249446 | 0.0839280070110771 |
| FLNB | 0.0865748110887876 | 0.0865748110887876 | 0.329568062230041 | 1.59677124357197 | 2.6474442859782 | 0.0726372369744417 |
| FLNC | 0.149606219564384 | 0.149606219564384 | 0.308626519766256 | 2.58889415634886 | 2.64543509865347 | 0.121104377820964 |
| FLOT1 | -0.031351527 | 0.968648473 | -0.678219531 | 0.0191040627785121 | -1.713051901 | 0.181184707940497 |
| FLOT2 | -0.040765952 | 0.959234048 | -0.731388584 | -0.111064197 | -2.374685575 | 0.0926613647437377 |
| FLVCR2 | -34.92205927 | -33.92205927 | -13.6150025 | 7.1044574694214 | -0.707106781 | 0.539497285386707 |
| FLYWCH2 | -1.958852833 | -0.958852833 | -19.21497092 | 0.933491393377791 | -2.041773773 | 0.187211943197938 |
| FMNL1 | -0.499865566 | 0.500134434 | -15.09381112 | 6.61696762454104 | -0.877468336 | 0.50542243586687 |
| FMNL2 | 0.103500298481361 | 0.103500298481361 | 0.354423294181228 | 1.33029182290798 | 3.09921239690417 | 0.0563223029448156 |
| FMNL3 | 0.522223148021613 | 0.522223148021613 | -5.805767876 | 13.882174446077 | 0.920684728464894 | 0.483749664205478 |
| FMO1 | -0.44582518 | 0.55417482 | -13.09628954 | 6.41609910218895 | -0.766264936 | 0.539497285386707 |
| FMO3 | 0.0531254514297395 | 0.0531254514297395 | -0.353143197 | 1.34877161349558 | 1.03024863110406 | 0.378670181966966 |
| FMOD | 0.100329626716832 | 0.100329626716832 | 0.0185112189391386 | 2.43789153888623 | 1.87465344184326 | 0.175707003568027 |
| FMR1 | 0.0132020758300061 | 0.0132020758300061 | -0.268523079 | 0.500403322570664 | 0.595947754238583 | 0.583352535252264 |
| FN1 | 0.0697623156707945 | 0.0697623156707945 | 0.0396515786000242 | 1.59219158804554 | 1.95475019859515 | 0.167509566752535 |
| FN3K | 0.0379175196318007 | 0.0379175196318007 | 0.102737180176535 | 0.595606442916447 | 2.46269765782312 | 0.0841851891763818 |
| FN3KRP | 0.0170000140530895 | 0.0170000140530895 | -0.41222822 | 0.732641061178127 | 0.501296244630244 | 0.632590957913431 |
| FNBP1 | -0.452446872 | 0.547553128 | -14.25611793 | 6.90460347270597 | -0.780924334 | 0.539497285386707 |
| FNBP4 | -36.35304356 | -35.35304356 | -18.10317147 | 0.548749142977759 | -2.117917658 | 0.1803694486231 |
| FNDC1 | 0.512962554548369 | 0.512962554548369 | -6.399371886 | 15.0155304851781 | 0.903994402779001 | 0.491705776536746 |
| FNDC3A | -0.085189297 | 0.914810703 | -1.221758401 | -0.485397662 | -4.158768903 | 0.0288778128885194 |
| FNDC3B | -0.009454656 | 0.990545344 | -0.551104169 | 0.369946966772406 | -0.343795578 | 0.737456893686127 |
| FNTA | 0.0242436447998345 | 0.0242436447998345 | 0.0778531857167364 | 0.381045024453575 | 2.85808390384585 | 0.0816570208654121 |
| FNTB | 0.0972055935637009 | 0.0972055935637009 | -10.93684708 | 11.6978718041199 | 0.0582333597850708 | 0.987160144644494 |
| FOCAD | -0.042556842 | 0.957443158 | -0.771655361 | -0.029600119 | -1.97847824 | 0.158835588885167 |
| FOLR2 | -0.002927156 | 0.997072844 | -0.41554454 | 0.355018437473041 | -0.152461225 | 0.911675472891325 |
| FOLR3 | -35.06980803 | -34.06980803 | -15.08323628 | 7.87059794445555 | -0.707106781 | 0.539497285386707 |
| FOS | 36.3418285271498 | 36.3418285271498 | -0.564241821 | 17.98273086 | 2.11340891155337 | 0.1803694486231 |
| FOSL2 | 0.924277156144939 | 0.924277156144939 | -8.111024552 | 13.4171811919502 | 0.427363006077123 | 0.668270163327605 |
| FOXE1 | 0 | 1 | NA | NA | NA | NA |
| FOXK1 | 0.414858801253762 | 0.414858801253762 | -7.028776307 | 13.4658084681667 | 0.706029176146914 | 0.539786809961107 |
| FOXRED1 | -0.440219217 | 0.559780783 | -13.69239532 | 6.8015716020042 | -0.756544035 | 0.539497285386707 |
| FPGS | 0 | 1 | NA | NA | NA | NA |
| FREM2 | 36.1073383234857 | 36.1073383234857 | -6.199123993 | 21.0045836285587 | 1.22472866633519 | 0.366275438800159 |
| FRG1 | -0.068271159 | 0.931728841 | -1.415675226 | 0.263022685970211 | -1.236296016 | 0.334509726783066 |
| FRMD6 | 36.8414088878666 | 36.8414088878666 | 11.6281269790283 | 12.9981723203375 | 40.4492338684574 | 0.000682566960680831 |
| FRMD8 | -0.016212742 | 0.983787258 | -0.588656672 | 0.309227032744354 | -0.539785135 | 0.607362336036313 |
| FRMPD1 | 0.168892221262437 | 0.168892221262437 | 1.38422650394369 | 2.20380652550544 | 8.44553658985685 | 0.00745505134779027 |
| FRY | 34.4460243286403 | 34.4460243286403 | -5.107755755 | 9.78851765384404 | 0.707106781186548 | 0.539497285386707 |
| FRYL | -0.093544604 | 0.906455396 | -1.032874441 | -0.555414199 | -5.81035998 | 0.00742123975108202 |
| FRZB | 0.14585369774019 | 0.14585369774019 | 0.873238011678005 | 2.10880412148116 | 4.43911213103256 | 0.0288778128885194 |
| FSCN1 | 0.117218042773891 | 0.117218042773891 | 0.90793200673368 | 1.67684757716471 | 6.79793227309261 | 0.0198686046776442 |
| FST | 0 | 1 | NA | NA | NA | NA |
| FSTL1 | 0.0514145044668086 | 0.0514145044668086 | -0.162455587 | 1.04635542009796 | 1.2773003613591 | 0.306338462175826 |
| FTH1 | -0.083364843 | 0.916635157 | -1.698706047 | -0.187996985 | -2.167955869 | 0.112658214048308 |
| FTL | -0.118803184 | 0.881196816 | -2.510273397 | -0.260765833 | -2.28554564 | 0.120767935051272 |
| FTO | -0.015785285 | 0.984214715 | -0.441970911 | 0.153952028491175 | -1.006146534 | 0.42457532978048 |
| FTSJ3 | -0.364530492 | 0.635469508 | -14.057143 | 8.07640647865864 | -0.606771914 | 0.589099143327268 |
| FUBP1 | -0.041921343 | 0.958078657 | -0.617620304 | -0.286595087 | -4.811604962 | 0.0159780933511772 |
| FUBP3 | -0.003551578 | 0.996448422 | -0.39964042 | 0.329731693208709 | -0.210439597 | 0.863217189048696 |
| FUCA1 | -0.081828153 | 0.918171847 | -1.423484646 | -0.157038953 | -2.331849605 | 0.118310015598945 |
| FUCA2 | -36.39095246 | -35.39095246 | -18.59732334 | 0.575519587841946 | -2.115231127 | 0.1803694486231 |
| FUNDC2 | -0.049993539 | 0.950006461 | -1.110894348 | 0.142505199136143 | -1.493682291 | 0.262891724110501 |
| FUS | 0.012288406882764 | 0.012288406882764 | -0.181067656 | 0.439697463268272 | 0.749054349735815 | 0.535425881029537 |
| FUT8 | -0.093693385 | 0.906306615 | -1.183198803 | -0.608169197 | -5.651899346 | 0.0130261633012374 |
| FXN | 0.0304306538109091 | 0.0304306538109091 | -0.448630075 | 1.02738065970361 | 0.689894273026551 | 0.539497285386707 |
| FXR1 | -0.022920129 | 0.977079871 | -0.54639689 | 0.118180280033929 | -1.135249384 | 0.364559456625742 |
| FXR2 | -0.419895881 | 0.580104119 | -13.50984528 | 6.98417053159004 | -0.716255156 | 0.539497285386707 |
| FXYD1 | 0.512986084062062 | 0.512986084062062 | -5.718016157 | 13.4113114565026 | 0.902314819972105 | 0.492111525301096 |
| FXYD3 | -34.82725589 | -33.82725589 | -12.74908641 | 6.65261296627272 | -0.707106781 | 0.539497285386707 |
| FYB1 | -1.627057744 | -0.627057744 | -18.18119973 | 4.6250007592998 | -1.028684944 | 0.375560518269785 |
| FYCO1 | 0.110352017026152 | 0.110352017026152 | 0.45635791149876 | 1.54967962731364 | 3.74036146971447 | 0.0625959367903568 |
| FYN | 0.568193701780691 | 0.568193701780691 | -7.437179121 | 12.5880567607982 | 0.447335426908568 | 0.657423743030449 |
| FYTTD1 | -0.98820339 | 0.01179661 | -18.42817254 | 5.57138473988246 | -1.203967162 | 0.36731973075278 |
| FZD1 | 1.73305909128775 | 1.73305909128775 | -3.678096964 | 16.3998685375102 | 1.09925898264937 | 0.366275438800159 |
| FZD7 | 0.159413639307874 | 0.159413639307874 | 0.307107698116813 | 2.27315051539393 | 2.27998516701736 | 0.100854539292597 |
| G3BP1 | -0.017913211 | 0.982086789 | -0.470492936 | 0.11560498607144 | -1.061981459 | 0.36731973075278 |
| G3BP2 | -0.04393961 | 0.95606039 | -0.798747686 | 0.00130524436699986 | -2.019577311 | 0.179832306528571 |
| G6PD | -0.015212343 | 0.984787657 | -0.266904966 | -0.054707328 | -3.039284761 | 0.0859803986121198 |
| GAA | -0.022260287 | 0.977739713 | -0.82287876 | 0.37284061552445 | -0.651936921 | 0.540130662224582 |
| GAB1 | -0.971171412 | 0.028828588 | -17.24261501 | 5.38683629031883 | -1.173599719 | 0.374572457284349 |
| GABARAPL1 | 0.121280554789145 | 0.121280554789145 | 0.282460230593835 | 2.31597283991521 | 2.22644246900944 | 0.107543581937135 |
| GABARAPL2 | 0.412559771586004 | 0.412559771586004 | -6.401825479 | 12.2118185244021 | 0.700815780030954 | 0.541981380751507 |
| GABPA | -0.445824554 | 0.554175446 | -13.87596062 | 6.81340302487185 | -0.76764369 | 0.539497285386707 |
| GABPB1 | -36.92900551 | -35.92900551 | -13.63314667 | -12.53472282 | -53.60983858 | 0.00040238102253647 |
| GADD45GIP1 | -0.461589658 | 0.538410342 | -12.94522901 | 6.15902324005658 | -0.799066355 | 0.537923342132161 |
| GAK | -0.446867428 | 0.553132572 | -14.82797789 | 7.26305834906505 | -0.769796599 | 0.539497285386707 |
| GALC | -0.005158287 | 0.994841713 | -0.413369582 | 0.315691842133971 | -0.235533896 | 0.832539751576661 |
| GALE | -0.028012221 | 0.971987779 | -0.963967082 | 0.46658274966125 | -0.676860454 | 0.539786809961107 |
| GALK1 | -0.004729376 | 0.995270624 | -0.268544485 | 0.171186121131447 | -0.393767963 | 0.695535903600177 |
| GALK2 | -0.04781034 | 0.95218966 | -0.799696689 | -0.071888852 | -2.291214569 | 0.129450833435746 |
| GALM | 0.0441404940175901 | 0.0441404940175901 | 0.0636871517394853 | 0.773064937625486 | 2.52276607472814 | 0.13962809743939 |
| GALNS | 0.0332252860075992 | 0.0332252860075992 | -0.620044948 | 1.19642582412153 | 0.586466393788801 | 0.582482178881234 |
| GALNT1 | 0.108007884916489 | 0.108007884916489 | 0.456399433414949 | 1.61637679884457 | 3.36567286136278 | 0.0571409867172479 |
| GALNT2 | -0.054938076 | 0.945061924 | -0.784352697 | -0.337753275 | -4.457456485 | 0.0218015022926752 |
| GALNT3 | 0 | 1 | NA | NA | NA | NA |
| GALNT5 | 0.494966308060661 | 0.494966308060661 | -6.158605851 | 13.8945264329358 | 0.866745135263147 | 0.50995353643859 |
| GALNT7 | -36.90268834 | -35.90268834 | -13.00784635 | -12.68700478 | -180.219407 | 0.000135517346157358 |
| GALT | -0.015080102 | 0.984919898 | -0.331159765 | 0.0509612662201287 | -1.289012307 | 0.303628347410128 |
| GAMT | 1.58815891589804 | 1.58815891589804 | -4.72900544 | 17.7482288167415 | 1.00218080929654 | 0.38541466403847 |
| GANAB | -0.026994185 | 0.973005815 | -0.562747916 | -0.079816401 | -2.307835102 | 0.0972989234850956 |
| GAPDH | 0.0096231360591434 | 0.0096231360591434 | -0.452988722 | 0.691258939227106 | 0.425008348481149 | 0.680927421634294 |
| GAPVD1 | 0.00148847599252786 | 0.00148847599252786 | -0.211263521 | 0.23964570799571 | 0.113520248339108 | 0.943295035661147 |
| GAR1 | -0.037820864 | 0.962179136 | -0.662509532 | -0.104952432 | -2.39973007 | 0.0896916084940673 |
| GARS1 | -0.029835002 | 0.970164998 | -0.760344542 | 0.148397972727676 | -1.467779607 | 0.309260370229649 |
| GART | -0.004564532 | 0.995435468 | -0.279357058 | 0.185657493796778 | -0.361249022 | 0.723886380723939 |
| GASK1B | 0.0712470259400068 | 0.0712470259400068 | -11.36511317 | 12.3776222053158 | 0.0738197559009746 | 0.975431641972397 |
| GATAD2A | -0.104054078 | 0.895945922 | -1.580145822 | -0.218579905 | -2.816735107 | 0.114919247158557 |
| GATAD2B | 0.0377034557404212 | 0.0377034557404212 | 0.0926396011236205 | 0.602591612179582 | 2.76110800558991 | 0.106098499797615 |
| GATC | -2.07428231 | -1.07428231 | -19.58348554 | -0.090947724 | -2.270320907 | 0.17758933085722 |
| GATD1 | 0.0265006662454327 | 0.0265006662454327 | -0.134145489 | 0.682854851587253 | 1.16865500387577 | 0.351294549216458 |
| GATM | -0.423726832 | 0.576273168 | -13.15834372 | 6.74997933328459 | -0.724013866 | 0.539497285386707 |
| GBA | -0.024059189 | 0.975940811 | -0.680878135 | 0.22470710709607 | -0.894385967 | 0.451083219116073 |
| GBE1 | 0.00273197288976176 | 0.00273197288976176 | -0.104658585 | 0.158001174994359 | 0.374301618340115 | 0.71463840664295 |
| GBF1 | -0.040058118 | 0.959941882 | -0.829298718 | 0.0181779322373239 | -2.031976087 | 0.1803694486231 |
| GBP1 | 0.0396048795944808 | 0.0396048795944808 | 0.164861185283058 | 0.617294073679052 | 3.01259269131233 | 0.0533963898117674 |
| GBP2 | 0.0321451273139417 | 0.0321451273139417 | 0.108370050977048 | 0.526053712732462 | 3.20546762026586 | 0.0896916084940673 |
| GBP4 | 0 | 1 | NA | NA | NA | NA |
| GBP5 | 0.983891571611584 | 0.983891571611584 | -5.670577429 | 18.8436111495463 | 1.18550666238383 | 0.368508989355442 |
| GBP6 | 0.0130394617085578 | 0.0130394617085578 | -11.85049764 | 12.0371190378877 | 0.01351739650206 | 0.999130434024431 |
| GBP7 | 34.7298944864338 | 34.7298944864338 | -6.218470154 | 11.9170938846463 | 0.707106781186547 | 0.539497285386707 |
| GC | 0.0856953515073526 | 0.0856953515073526 | 0.222683519463166 | 1.68670047591243 | 2.44014525533264 | 0.108627734565813 |
| GCA | -0.12204647 | 0.87795353 | -1.871356999 | -0.561122427 | -3.489430498 | 0.0537719121043574 |
| GCC1 | -0.426342584 | 0.573657416 | -13.54436751 | 6.91521248748469 | -0.728039056 | 0.539497285386707 |
| GCDH | -0.046602142 | 0.953397858 | -0.954938704 | 0.0187289491546023 | -1.791316349 | 0.1803694486231 |
| GCHFR | -0.460447942 | 0.539552058 | -13.74153024 | 6.49261010769789 | -0.788193768 | 0.539497285386707 |
| GCLC | -0.007675797 | 0.992324203 | -0.52881115 | 0.37802232869341 | -0.28787828 | 0.784975376088116 |
| GCLM | 0.00430004578578692 | 0.00430004578578692 | -0.53624318 | 0.615370468723493 | 0.120186648310929 | 0.937155251472286 |
| GCN1 | -0.038341484 | 0.961658516 | -0.72483431 | -0.075951182 | -2.157254102 | 0.115722268902765 |
| GDAP1 | 0 | 1 | NA | NA | NA | NA |
| GDE1 | -0.446691298 | 0.553308702 | -13.41238454 | 6.57431675984749 | -0.769067259 | 0.539497285386707 |
| GDF10 | 0.99542889049309 | 0.99542889049309 | -9.499427439 | 16.351620438728 | 0.460803124888407 | 0.652187811666889 |
| GDI1 | 0.0434720974289726 | 0.0434720974289726 | 0.198944218532836 | 0.695449300790359 | 3.40905190363676 | 0.0565936076207959 |
| GDI2 | 0.00781391992732477 | 0.00781391992732477 | -0.261334832 | 0.429918986179218 | 0.484651945664418 | 0.648356244122926 |
| GDPD3 | 0 | 1 | NA | NA | NA | NA |
| GEMIN5 | -0.391159737 | 0.608840263 | -14.02289347 | 7.65328563782249 | -0.659952609 | 0.561015630932865 |
| GET3 | -0.004021599 | 0.995978401 | -0.303274348 | 0.219341538962351 | -0.333467394 | 0.755737904002073 |
| GET4 | 0.00491402483929729 | 0.00491402483929729 | -0.357226366 | 0.449868589665604 | 0.199241243807296 | 0.86584752864707 |
| GFAP | -0.033270568 | 0.966729432 | -10.74768457 | 10.6054159141422 | -0.011529423 | 0.999130434024431 |
| GFER | -1.205745807 | -0.205745807 | -17.23008919 | 2.90839422297991 | -1.599693717 | 0.28043206850922 |
| GFM1 | -0.467524096 | 0.532475904 | -15.54043855 | 7.30070643756089 | -0.811490503 | 0.534281958550569 |
| GFM2 | -1.07056397 | -0.07056397 | -18.86497301 | 4.73206430540126 | -1.345223182 | 0.354726159265742 |
| GFOD1 | 1.6214325662722 | 1.6214325662722 | -4.466315226 | 17.4479441360682 | 1.02511776840596 | 0.376604261704483 |
| GFPT1 | -0.024592804 | 0.975407196 | -0.801331786 | 0.313911187698721 | -0.880019824 | 0.485764639951488 |
| GFRA1 | 0.151045850443992 | 0.151045850443992 | -9.537285172 | 10.1293858102032 | 0.0522293525863886 | 0.990470984138181 |
| GFUS | 0.00152229131551463 | 0.00152229131551463 | -0.501893069 | 0.531866818639049 | 0.0502929835707285 | 0.991188924057953 |
| GGA1 | -36.34491878 | -35.34491878 | -18.04782216 | 0.591982754796646 | -2.107392655 | 0.18042925372161 |
| GGA2 | -2.103512609 | -1.103512609 | -19.73739376 | -0.372152118 | -2.32763666 | 0.1722731964937 |
| GGACT | -0.603009246 | 0.396990754 | -13.95385998 | 7.9380439427356 | -0.477375046 | 0.644233855679381 |
| GGCT | -0.421310832 | 0.578689168 | -13.33826018 | 6.86568796640165 | -0.71581131 | 0.539497285386707 |
| GGCX | 0.590496722140012 | 0.590496722140012 | -8.848372955 | 15.357348814731 | 0.467452882723904 | 0.649150111336054 |
| GGH | -0.014862074 | 0.985137926 | -0.558071927 | 0.270713821128716 | -0.657223385 | 0.54733731056867 |
| GGPS1 | 0.514590731580095 | 0.514590731580095 | -5.259234818 | 12.409706922654 | 0.904043890329622 | 0.49124461671169 |
| GGT5 | -0.001964141 | 0.998035859 | -0.498450044 | 0.458228480963201 | -0.07864845 | 0.972765179053006 |
| GHDC | -0.079487321 | 0.920512679 | -1.055929039 | -0.528720184 | -5.315607283 | 0.0122282448667971 |
| GHITM | -0.013536493 | 0.986463507 | -0.40411993 | 0.134720395936219 | -0.870161472 | 0.462072596778855 |
| GID8 | 1.56105630638725 | 1.56105630638725 | -4.607381457 | 16.7192278501594 | 0.982688543710421 | 0.39396092296319 |
| GIGYF2 | -0.049241852 | 0.950758148 | -1.085802992 | 0.184217056016256 | -1.48321586 | 0.292213987153759 |
| GIMAP1 | -0.040779872 | 0.959220128 | -0.992379348 | 0.208688307011937 | -1.193348829 | 0.354826113544616 |
| GIMAP2 | -2.016833762 | -1.016833762 | -17.40771324 | 0.35839234849384 | -2.150500815 | 0.1803694486231 |
| GIMAP4 | -0.029918254 | 0.970081746 | -0.545779075 | -0.066391102 | -2.211652555 | 0.107311310221535 |
| GIMAP5 | -0.495783526 | 0.504216474 | -13.69996008 | 6.05983645760882 | -0.866472087 | 0.509932647461076 |
| GIMAP7 | -0.043986709 | 0.956013291 | -11.49269003 | 11.147400457435 | -0.026393915 | 0.998787170483424 |
| GIMAP8 | -0.008184139 | 0.991815861 | -0.456166028 | 0.316556186776845 | -0.320677616 | 0.757847952376997 |
| GINS3 | 0 | 1 | NA | NA | NA | NA |
| GIPC1 | 0.121963454424709 | 0.121963454424709 | 0.168942630554375 | 1.97643293365952 | 2.65489312947456 | 0.14001882095693 |
| GIT1 | 0.0218756301082146 | 0.0218756301082146 | 0.0400048323101468 | 0.364534053825912 | 2.20593103063221 | 0.114381125871646 |
| GIT2 | 0.0163343159411172 | 0.0163343159411172 | -0.008369638 | 0.328840909608516 | 1.75699100918549 | 0.1803694486231 |
| GJA1 | 0.120230898024686 | 0.120230898024686 | -0.214706141 | 2.51870795593873 | 1.80245885885657 | 0.214787651786783 |
| GJB2 | 0 | 1 | NA | NA | NA | NA |
| GLA | -0.019233949 | 0.980766051 | -10.68052589 | 10.4365787815178 | -0.019988577 | 0.999130434024431 |
| GLB1 | -0.00121923 | 0.99878077 | -0.262175594 | 0.2380924343217 | -0.084684254 | 0.968616145870256 |
| GLE1 | -35.74110164 | -34.74110164 | -16.29583755 | 4.80970910210875 | -1.22468148 | 0.366275438800159 |
| GLG1 | 0.00376173998400025 | 0.00376173998400025 | -0.22948515 | 0.311489810403103 | 0.273227066162416 | 0.799715374157191 |
| GLI2 | 1.64065729868694 | 1.64065729868694 | -4.818539446 | 19.2696402877531 | 1.03838257605922 | 0.37248434920495 |
| GLIPR2 | -0.009435544 | 0.990564456 | -0.447238908 | 0.258773012300987 | -0.491869069 | 0.639580078990356 |
| GLMN | -34.93729842 | -33.93729842 | -13.75957964 | 7.17989940738802 | -0.707106781 | 0.539497285386707 |
| GLO1 | 0.0430830906149236 | 0.0430830906149236 | 0.0180144639330439 | 0.877141835981943 | 2.18521206700091 | 0.170774187741151 |
| GLOD4 | -0.001677344 | 0.998322656 | -0.30092392 | 0.2667770902386 | -0.111641161 | 0.945186096678396 |
| GLRX | -0.136063788 | 0.863936212 | -1.804242115 | -1.015284853 | -6.376287357 | 0.00721761206846586 |
| GLRX3 | 0.00389317875568256 | 0.00389317875568256 | -0.504269254 | 0.578089114071533 | 0.149252044543113 | 0.915662793409887 |
| GLRX5 | -0.407320671 | 0.592679329 | -14.104413 | 7.46682705968211 | -0.691684023 | 0.54689616521613 |
| GLS | -0.032839481 | 0.967160519 | -0.712232168 | 0.0187740517438769 | -1.646579611 | 0.181184707940497 |
| GLS2 | 0.407604049239559 | 0.407604049239559 | -7.259994082 | 13.7115352408876 | 0.690050691683563 | 0.54733731056867 |
| GLT8D1 | -0.018728677 | 0.981271323 | -0.588476255 | 0.230914959924992 | -0.802947999 | 0.512154775182943 |
| GLT8D2 | 0.574480109608433 | 0.574480109608433 | -4.841042232 | 13.1278784824514 | 1.03248639079189 | 0.426877638881485 |
| GLTP | -0.461592923 | 0.538407077 | -13.23185651 | 6.28368992716819 | -0.798258001 | 0.537923342132161 |
| GLUD1 | 0.0109773506421821 | 0.0109773506421821 | -0.409630405 | 0.647516688604116 | 0.475616225554407 | 0.654554258595878 |
| GLUL | -0.502640541 | 0.497359459 | -14.77119523 | 6.43245182135918 | -0.882574323 | 0.502462760248914 |
| GLYR1 | 0.000571857608598473 | 0.000571857608598473 | -0.203584703 | 0.214746496788462 | 0.0480017237914086 | 0.992135986860447 |
| GM2A | -0.116808223 | 0.883191777 | -1.747396219 | -0.588753942 | -4.051470863 | 0.0525595373037303 |
| GMDS | -0.047085876 | 0.952914124 | -1.069684617 | 0.105391983093021 | -1.834088532 | 0.222112602842161 |
| GMEB1 | -0.052749747 | 0.947250253 | -12.69762724 | 12.2415679713929 | -0.031650463 | 0.998435102357591 |
| GMFB | 0.0238459801363628 | 0.0238459801363628 | -0.217225463 | 0.654279054179648 | 0.867827957526665 | 0.462072596778855 |
| GMFG | -0.081254384 | 0.918745616 | -1.01261383 | -0.517350506 | -5.45869228 | 0.0107826594792155 |
| GMIP | -36.89671701 | -35.89671701 | -13.08039373 | -12.508326 | -100.6577455 | 0.000182450656380933 |
| GMPPA | -0.073227184 | 0.926772816 | -1.284735847 | -0.203240362 | -2.914044849 | 0.10761361335075 |
| GMPPB | -0.100509411 | 0.899490589 | -1.811398728 | -0.332103911 | -3.125363666 | 0.100605018467369 |
| GMPR | -0.896958422 | 0.103041578 | -15.41796506 | 9.45825104919284 | -0.415053854 | 0.677370666860611 |
| GMPR2 | -0.008478253 | 0.991521747 | -0.689354153 | 0.52806188719164 | -0.26524824 | 0.811740301830937 |
| GMPS | -0.00054842 | 0.99945158 | -0.220400569 | 0.209547823906558 | -0.055744168 | 0.989487050150791 |
| GNA11 | 0.0770815342387271 | 0.0770815342387271 | 0.393439908210442 | 1.08853923345084 | 3.72454895013066 | 0.0323586606723677 |
| GNA12 | -0.025386545 | 0.974613455 | -11.93232874 | 11.7240124433738 | -0.015237435 | 0.999130434024431 |
| GNA13 | -0.013373693 | 0.986626307 | -0.40122533 | 0.141333788059247 | -0.925231648 | 0.451630680056826 |
| GNA14 | -0.69969581 | 0.30030419 | -11.93224305 | 8.19232182893709 | -0.321859149 | 0.755737904002073 |
| GNA15 | -0.988406636 | 0.011593364 | -17.25764381 | 5.21358636574964 | -1.204063194 | 0.36731973075278 |
| GNAI1 | 0.0723468008419443 | 0.0723468008419443 | 0.0713143329048109 | 1.31341049871279 | 2.17377115132266 | 0.150937151869948 |
| GNAI2 | 0.0104743216848616 | 0.0104743216848616 | -0.231692466 | 0.474152833207674 | 0.693746925321453 | 0.539497285386707 |
| GNAI3 | 0.00491391994977906 | 0.00491391994977906 | -0.217254664 | 0.321051943510715 | 0.337533622542716 | 0.74297107829644 |
| GNAO1 | 0.0108956876060705 | 0.0108956876060705 | -11.17069905 | 11.3175654940692 | 0.0113000411207626 | 0.999130434024431 |
| GNAQ | 0.062964088277905 | 0.062964088277905 | 0.187442001702804 | 1.05820782510007 | 3.21819082167292 | 0.104948996268386 |
| GNAS | 0.00607034565412285 | 0.00607034565412285 | -0.167146477 | 0.293951075380644 | 0.491202244835439 | 0.637535164767707 |
| GNAZ | -34.79574844 | -33.79574844 | -12.47367359 | 6.50889954133171 | -0.707106781 | 0.539497285386707 |
| GNB1 | 0.0173980043650175 | 0.0173980043650175 | -0.313900164 | 0.685989649822557 | 0.803064073255018 | 0.533032388260122 |
| GNB1L | -1.031246204 | -0.031246204 | -17.50080319 | 4.81409919260324 | -1.27651409 | 0.366275438800159 |
| GNB2 | 0.00830298056096037 | 0.00830298056096037 | -0.415801844 | 0.592041160360691 | 0.332190474503271 | 0.752425159425364 |
| GNB4 | 0.039901638180966 | 0.039901638180966 | -0.061232146 | 0.851119927891879 | 1.55481452033348 | 0.215920656179098 |
| GNE | -0.42301098 | 0.57698902 | -13.04850597 | 6.69699557079596 | -0.720907146 | 0.539497285386707 |
| GNG10 | -0.011862377 | 0.988137623 | -0.816587057 | 0.57162755111403 | -0.327301689 | 0.755442027220271 |
| GNG12 | 0.0485652546147368 | 0.0485652546147368 | -0.023697947 | 1.01552415763153 | 1.77597941581838 | 0.1803694486231 |
| GNG2 | 0.075340869275154 | 0.075340869275154 | 0.464796224318817 | 1.09472087040048 | 4.41351779209593 | 0.0232301628989628 |
| GNG4 | 35.7939233830227 | 35.7939233830227 | -4.992363396 | 16.9068297846698 | 1.22431330419078 | 0.366275438800159 |
| GNG5 | -0.023465005 | 0.976534995 | -0.589763574 | 0.150755351061232 | -1.109248521 | 0.366275438800159 |
| GNG7 | -0.520385489 | 0.479614511 | -14.35072363 | 6.00938894867024 | -0.920819037 | 0.48376182222999 |
| GNL1 | 0.0276346458629944 | 0.0276346458629944 | 0.108418859238568 | 0.402340063120705 | 3.01328292149274 | 0.0525595373037303 |
| GNL3 | -0.017458734 | 0.982541266 | -0.343205425 | 0.0166656755825322 | -1.620995773 | 0.196284152371001 |
| GNPAT | -0.314935433 | 0.685064567 | -12.53907461 | 7.846469354 | -0.516940034 | 0.638269825193503 |
| GNPDA1 | 0.0196285282318502 | 0.0196285282318502 | -0.103968378 | 0.501798710641473 | 1.39694774667697 | 0.325081185755388 |
| GNPDA2 | 0.0878264246517454 | 0.0878264246517454 | 0.208455537881904 | 1.3424460306333 | 2.79475307839435 | 0.106103077041777 |
| GNPNAT1 | 0.00742320143420989 | 0.00742320143420989 | -0.18799225 | 0.314753031594599 | 0.505876647652605 | 0.637428610542048 |
| GNS | -0.012276943 | 0.987723057 | -0.573334456 | 0.331111452719643 | -0.550384617 | 0.615716804180357 |
| GOLGA1 | -35.80573966 | -34.80573966 | -17.04405798 | 5.03160636843357 | -1.224514568 | 0.366275438800159 |
| GOLGA2 | -0.057695032 | 0.942304968 | -0.862764983 | -0.235550718 | -3.127439146 | 0.0545433511671234 |
| GOLGA3 | 0.0262001437675852 | 0.0262001437675852 | -0.232262766 | 0.748021756519306 | 0.951788396938338 | 0.421374606651904 |
| GOLGA4 | -0.361675439 | 0.638324561 | -13.49412015 | 7.79461399445026 | -0.60037086 | 0.592126758280289 |
| GOLGA5 | -0.036161478 | 0.963838522 | -0.57203998 | -0.086677702 | -2.522305443 | 0.10032605369018 |
| GOLGA7 | -0.39835541 | 0.60164459 | -13.4743027 | 7.25789550261805 | -0.674394859 | 0.554686898603132 |
| GOLGB1 | -0.024195293 | 0.975804707 | -0.572906495 | 0.102607386894053 | -1.356189632 | 0.312367517497055 |
| GOLIM4 | 0.0156221856991346 | 0.0156221856991346 | -0.115853638 | 0.422255598104126 | 0.988438217071553 | 0.392686887778417 |
| GOLM1 | 0.0109064600522779 | 0.0109064600522779 | -0.843181506 | 1.03588726571818 | 0.178152781927253 | 0.886317453822251 |
| GOLM2 | -0.015517968 | 0.984482032 | -0.31517518 | 0.0228629704875982 | -1.641352542 | 0.212290017230865 |
| GOLPH3 | -0.114956205 | 0.885043795 | -1.591970881 | -0.500389394 | -3.743099724 | 0.0542596324813655 |
| GOLPH3L | -1.017151776 | -0.017151776 | -18.2077566 | 5.17453666367134 | -1.253812064 | 0.366275438800159 |
| GOLT1B | 0.0190671070852899 | 0.0190671070852899 | -0.60727669 | 0.973762243416842 | 0.421370914635538 | 0.676369299432988 |
| GON7 | -0.036271317 | 0.963728683 | -11.33305768 | 10.8498459536077 | -0.037695255 | 0.996395923375039 |
| GOPC | 0.00762407457910663 | 0.00762407457910663 | -0.18217214 | 0.328315643511801 | 0.536346048519742 | 0.616550083670876 |
| GORASP2 | -0.053849122 | 0.946150878 | -0.944038487 | -0.133175195 | -2.374263044 | 0.100854235355595 |
| GOSR1 | -0.105068658 | 0.894931342 | -1.191899774 | -0.684386902 | -6.535247767 | 0.00570303541694555 |
| GOSR2 | -0.061801637 | 0.938198363 | -0.862152642 | -0.275176657 | -3.444789182 | 0.0428059353100352 |
| GOT1 | -0.023627522 | 0.976372478 | -0.821757732 | 0.336569629420568 | -0.743846419 | 0.536512897960712 |
| GOT2 | -0.029626756 | 0.970373244 | -0.55055008 | -0.109313952 | -2.591933359 | 0.074529101345398 |
| GP1BA | -0.012550581 | 0.987449419 | -1.034648983 | 0.79547480114134 | -0.240120784 | 0.83032566426907 |
| GP1BB | -0.022618618 | 0.977381382 | -1.180485096 | 0.74510450051276 | -0.408757927 | 0.684593869265198 |
| GP5 | -0.016521579 | 0.983478421 | -0.918486569 | 0.620459185130203 | -0.340831844 | 0.74045127354562 |
| GP9 | -0.01231279 | 0.98768721 | -1.273145763 | 1.02883629237685 | -0.190847558 | 0.874442070429676 |
| GPAA1 | 0.0519039167366647 | 0.0519039167366647 | 0.165970761292333 | 0.818627936510746 | 2.73586273975866 | 0.0781558085553345 |
| GPAM | 1.09370317561905 | 1.09370317561905 | -4.896727433 | 20.7431384692516 | 1.38429596745326 | 0.342870130939439 |
| GPAT3 | -1.035611679 | -0.035611679 | -15.81627007 | 4.27150630234852 | -1.279973576 | 0.366275438800159 |
| GPAT4 | -1.586237655 | -0.586237655 | -15.81174829 | 4.22539493854571 | -1.000494219 | 0.386284743929022 |
| GPC1 | 0.0870716539940946 | 0.0870716539940946 | 0.168428921852972 | 1.4089217061601 | 2.37683839437197 | 0.114209650550459 |
| GPC4 | 0.626320638939409 | 0.626320638939409 | -7.523596578 | 13.5571999516498 | 0.496765797514431 | 0.632907789449222 |
| GPD1 | 0 | 1 | NA | NA | NA | NA |
| GPD1L | 0.0160343437907641 | 0.0160343437907641 | -0.256581687 | 0.564153806799635 | 0.813110182540976 | 0.530227332284313 |
| GPD2 | -0.064350781 | 0.935649219 | -0.834331911 | -0.448618765 | -5.772211231 | 0.00711048753060642 |
| GPHN | 0.0156625825008976 | 0.0156625825008976 | -0.214307815 | 0.498681146244267 | 0.779843729536951 | 0.532053314571769 |
| GPI | 0.0137794722585194 | 0.0137794722585194 | -0.127421598 | 0.436884045940362 | 1.02332483730877 | 0.392686887778417 |
| GPKOW | -0.674617473 | 0.325382527 | -15.67113948 | 8.26226012522413 | -0.536508101 | 0.609890353960562 |
| GPLD1 | 0.0329796319110453 | 0.0329796319110453 | 0.0822636084106153 | 0.509347923711582 | 2.493507053 | 0.0919270569052632 |
| GPN1 | -0.517691141 | 0.482308859 | -13.70264045 | 8.51757861448629 | -0.407171593 | 0.683505240507025 |
| GPNMB | -0.062117086 | 0.937882914 | -2.918689648 | 1.5040821363665 | -0.70844285 | 0.539497285386707 |
| GPR180 | -1.987414363 | -0.987414363 | -17.87365194 | 0.614018626231516 | -2.093481102 | 0.180716766229804 |
| GPR183 | -35.78177029 | -34.78177029 | -16.76729853 | 4.95277668752172 | -1.224054942 | 0.366275438800159 |
| GPRC5B | 2.0709719634373 | 2.0709719634373 | 0.0630587611596723 | 19.2452172466438 | 2.2630022620624 | 0.178364820794953 |
| GPRIN3 | -36.85945997 | -35.85945997 | -13.11369923 | -11.82266137 | -43.46504973 | 0.00058288378693748 |
| GPS1 | 0.019032327870719 | 0.019032327870719 | -0.120660386 | 0.499593684784281 | 1.09289178547369 | 0.366275438800159 |
| GPSM1 | 35.7466830480001 | 35.7466830480001 | -4.850333052 | 16.3809843472296 | 1.22214569632111 | 0.366275438800159 |
| GPSM3 | -0.562119179 | 0.437880821 | -13.807837 | 5.25367693883695 | -1.007489325 | 0.439615861134292 |
| GPT | 0 | 1 | NA | NA | NA | NA |
| GPT2 | 0 | 1 | NA | NA | NA | NA |
| GPX1 | -0.035670545 | 0.964329455 | -0.852448613 | 0.0762862729221009 | -1.5046696 | 0.232023128273538 |
| GPX2 | 0 | 1 | NA | NA | NA | NA |
| GPX3 | 0.137461927166362 | 0.137461927166362 | 0.354105942904757 | 2.57499534579028 | 2.48915960885867 | 0.107311310221535 |
| GPX4 | -0.028664472 | 0.971335528 | -0.532096622 | -0.03549601 | -2.211621205 | 0.142781177075778 |
| GPX7 | 0.108723068662818 | 0.108723068662818 | 0.428099113204391 | 1.68019729476647 | 3.27984786502359 | 0.0695145969750744 |
| GPX8 | 0.127265494187065 | 0.127265494187065 | 0.513947982339879 | 2.11478688940813 | 3.44239635094028 | 0.0795772810243883 |
| GRAMD4 | -1.962630004 | -0.962630004 | -17.84734395 | 0.826009894738412 | -2.047208744 | 0.186250725889806 |
| GRAP2 | -0.636686154 | 0.363313846 | -15.36735835 | 8.43334159128305 | -0.505502765 | 0.627898998475351 |
| GRB14 | 0 | 1 | NA | NA | NA | NA |
| GRB2 | -0.064500922 | 0.935499078 | -1.058423062 | -0.282602147 | -2.99989531 | 0.0531501602223687 |
| GREM1 | 0 | 1 | NA | NA | NA | NA |
| GRHL1 | 0 | 1 | NA | NA | NA | NA |
| GRHPR | 0.0096639247949092 | 0.0096639247949092 | -0.090837626 | 0.290353414538869 | 0.917842895271911 | 0.433294790280052 |
| GRIP1 | -0.016212058 | 0.983787942 | -0.939489226 | 0.636368161063068 | -0.348155403 | 0.73614944378499 |
| GRIPAP1 | -0.021862501 | 0.978137499 | -0.422302378 | 0.0367358487767626 | -1.568318786 | 0.226552045592464 |
| GRK2 | -0.04250851 | 0.95749149 | -0.723241016 | -0.092296946 | -2.249211281 | 0.104333150555025 |
| GRK6 | -36.94949121 | -35.94949121 | -13.49175579 | -13.0503377 | -135.3103875 | 0.000139499733991025 |
| GRN | -0.045126016 | 0.954873984 | -1.041345424 | 0.0744759232889908 | -1.551353882 | 0.215813106586992 |
| GRPEL1 | -0.060973175 | 0.939026825 | -0.754816318 | -0.463344838 | -7.233314013 | 0.00235686980595526 |
| GRSF1 | -0.460009316 | 0.539990684 | -13.76715943 | 6.56608053348702 | -0.79571481 | 0.539074876138249 |
| GRWD1 | -0.071339756 | 0.928660244 | -0.918153522 | -0.29499822 | -3.417362547 | 0.0407524281451239 |
| GSDMA | 0.0341422009930587 | 0.0341422009930587 | -10.46945264 | 10.6136002783821 | 0.011831404774882 | 0.999130434024431 |
| GSDMC | 0 | 1 | NA | NA | NA | NA |
| GSDMD | 0.0056204641535791 | 0.0056204641535791 | -0.289335875 | 0.39721558312128 | 0.303057422213132 | 0.777689584711742 |
| GSDME | 0.0347598870876336 | 0.0347598870876336 | -0.136653156 | 0.800784681291812 | 1.22881173400242 | 0.325081185755388 |
| GSK3A | 0.00620581855176888 | 0.00620581855176888 | -0.341546935 | 0.45399605720937 | 0.260741844434333 | 0.811991625860354 |
| GSK3B | -0.43908893 | 0.56091107 | -14.22073347 | 7.07192779646305 | -0.753798636 | 0.539497285386707 |
| GSKIP | -35.8732219 | -34.8732219 | -17.85861994 | 5.27093604824046 | -1.224685623 | 0.366275438800159 |
| GSN | 0.0493487478457693 | 0.0493487478457693 | 0.374239213096695 | 0.840721598471473 | 5.37978684571907 | 0.0356286051155568 |
| GSPT1 | 0.00097859903383723 | 0.00097859903383723 | -0.368061098 | 0.38757398487455 | 0.051053463154743 | 0.991332244908879 |
| GSPT2 | 0.36330856724833 | 0.36330856724833 | -7.833831827 | 13.6529907598476 | 0.605856367053036 | 0.589099143327268 |
| GSR | 0.0122720261258181 | 0.0122720261258181 | -0.227554726 | 0.497555801496644 | 0.786248550598215 | 0.537375417204127 |
| GSS | 0.00476268031006578 | 0.00476268031006578 | -0.256020244 | 0.358990226336245 | 0.310681656087772 | 0.768886837772054 |
| GSTA1 | 0 | 1 | NA | NA | NA | NA |
| GSTA4 | 36.9602098822512 | 36.9602098822512 | 13.1067819252208 | 13.6332435239038 | 114.298677419529 | 0.000170529874772727 |
| GSTK1 | -0.063044767 | 0.936955233 | -1.224925664 | -0.056871548 | -2.306682571 | 0.158294098139884 |
| GSTM1 | -36.89161531 | -35.89161531 | -13.09012979 | -12.40826208 | -84.15089741 | 0.000220658446544722 |
| GSTM2 | 0.0360866257405259 | 0.0360866257405259 | -0.030813741 | 0.70799849912759 | 1.58601502739412 | 0.193105206646518 |
| GSTM3 | 0.131628506442082 | 0.131628506442082 | 0.653992845734399 | 1.83808855070413 | 4.55791818137214 | 0.0548717713947671 |
| GSTM4 | -0.941770928 | 0.058229072 | -14.38116001 | 8.60360070137062 | -0.436043076 | 0.662981888106935 |
| GSTM5 | 1.06367971575751 | 1.06367971575751 | -4.539441907 | 17.9287661930987 | 1.3305816235211 | 0.356621571555239 |
| GSTO1 | -0.003713126 | 0.996286874 | -0.4930344 | 0.410441957754252 | -0.15880497 | 0.903227424927662 |
| GSTP1 | -0.003493675 | 0.996506325 | -0.328006336 | 0.247556915499665 | -0.261793579 | 0.811931297973326 |
| GSTT1 | 0.0271179736056368 | 0.0271179736056368 | -11.420198 | 11.7980866495861 | 0.0281630032485909 | 0.998435102357591 |
| GSTZ1 | -0.048115731 | 0.951884269 | -1.155652665 | 0.314655336546241 | -1.001201928 | 0.388690732112018 |
| GTF2A1 | -0.032989066 | 0.967010934 | -0.668491461 | 0.0747595848618576 | -1.537033944 | 0.247131879512359 |
| GTF2A2 | -0.039844819 | 0.960155181 | -0.620063242 | -0.125998577 | -2.847765292 | 0.0818565574308722 |
| GTF2B | -0.507397464 | 0.492602536 | -13.88257936 | 5.99067358202897 | -0.89356129 | 0.497019270930624 |
| GTF2E1 | -36.78966015 | -35.78966015 | -12.48981537 | -11.26880563 | -43.78730231 | 0.00057581819891853 |
| GTF2F1 | -1.058024556 | -0.058024556 | -17.80244002 | 4.60518698678302 | -1.325198553 | 0.359451193776443 |
| GTF2F2 | -0.479316972 | 0.520683028 | -13.54345347 | 6.20771926641223 | -0.835564387 | 0.524386993255536 |
| GTF2H4 | -0.536096272 | 0.463903728 | -14.30139088 | 8.72580332181054 | -0.422017668 | 0.672688064287923 |
| GTF2I | 0.0254575517182516 | 0.0254575517182516 | -0.050210174 | 0.555702116618381 | 1.45948097133164 | 0.235998473152946 |
| GTF3C1 | -1.078660382 | -0.078660382 | -18.5887181 | 4.54426131332611 | -1.358627569 | 0.350303616970168 |
| GTF3C2 | -0.321837288 | 0.678162712 | -12.77305658 | 7.9000361997866 | -0.528756106 | 0.632590957913431 |
| GTF3C3 | -0.009761869 | 0.990238131 | -0.448351452 | 0.278244131849546 | -0.41761844 | 0.677370666860611 |
| GTF3C4 | -0.001066822 | 0.998933178 | -0.223894584 | 0.204292877673725 | -0.081472496 | 0.970298134767008 |
| GTF3C5 | -0.018571595 | 0.981428405 | -0.413693318 | 0.0701615109903419 | -1.377128193 | 0.303206953234416 |
| GTPBP1 | -0.013937645 | 0.986062355 | -0.436086678 | 0.182377145086145 | -0.797239373 | 0.523419603040466 |
| GTPBP3 | -36.50451173 | -35.50451173 | -20.10213748 | 0.604454551017787 | -2.118948804 | 0.1803694486231 |
| GTPBP4 | -0.066465017 | 0.933534983 | -1.248009248 | 0.0454625998312554 | -1.795066438 | 0.184584486541437 |
| GTPBP6 | -2.036009473 | -1.036009473 | -19.17407972 | 0.217456440060921 | -2.18714456 | 0.1803694486231 |
| GUCY1A1 | 0.0276974844951721 | 0.0276974844951721 | -0.403484136 | 0.896384290385775 | 0.823777021071738 | 0.525114988876483 |
| GUCY1B1 | 1.03135058009443 | 1.03135058009443 | -4.663201354 | 17.0015689552274 | 1.2754119223834 | 0.366275438800159 |
| GUF1 | -1.150491409 | -0.150491409 | -12.3215756 | 6.61108699351377 | -0.529626823 | 0.615155534697666 |
| GUK1 | 0.0381638327476621 | 0.0381638327476621 | -0.209494035 | 0.968017311373431 | 1.15027203983687 | 0.36309241194026 |
| GULP1 | 2.07656192527726 | 2.07656192527726 | 0.123215160623233 | 19.359958689055 | 2.27254051554756 | 0.177014650065806 |
| GUSB | -0.105195457 | 0.894804543 | -1.579564956 | -0.668066211 | -4.267856662 | 0.0200603409546028 |
| GXYLT2 | 0.550973589110895 | 0.550973589110895 | -5.247795028 | 13.3680757791395 | 0.979433593068332 | 0.454142967601296 |
| GYG1 | 0.00239625501443426 | 0.00239625501443426 | -0.26177921 | 0.311275669915335 | 0.173790060443393 | 0.894472909651872 |
| GYPA | -1.083433594 | -0.083433594 | -15.09083522 | 8.36677855359948 | -0.5007666 | 0.631075157183946 |
| GYPC | 0.0106923192793492 | 0.0106923192793492 | -0.974146268 | 1.15774352340337 | 0.153159422598621 | 0.909455278857403 |
| GYS1 | -0.009681869 | 0.990318131 | -0.386758851 | 0.200359811467932 | -0.563718539 | 0.593037441469552 |
| GZMA | -1.031906551 | -0.031906551 | -18.28258287 | 5.02032306357343 | -1.277585671 | 0.366275438800159 |
| GZMK | -0.113856157 | 0.886143843 | -2.654079843 | 0.475681223215121 | -1.436049375 | 0.302485340561127 |
| H1-0 | 0.0526433113672517 | 0.0526433113672517 | 0.214370379758019 | 1.06808562623512 | 2.71169515904566 | 0.0783024065441912 |
| H1-1 | -1.034014853 | -0.034014853 | -17.99639816 | 4.92662179202419 | -1.28183823 | 0.366275438800159 |
| H1-10 | -0.037787539 | 0.962212461 | -0.986360592 | 0.149191625455918 | -1.549469305 | 0.273270742670169 |
| H1-2 | -0.96964176 | 0.03035824 | -18.65993552 | 5.85461686358775 | -1.165352084 | 0.375817581209462 |
| H1-3 | -1.10502495 | -0.10502495 | -17.40435824 | 4.03099142445108 | -1.397262455 | 0.339321903969437 |
| H1-4 | -1.078562237 | -0.078562237 | -22.21377307 | 5.45902959077122 | -1.359881806 | 0.350426488005461 |
| H1-5 | -0.111567677 | 0.888432323 | -2.116015132 | -0.483762209 | -3.389803983 | 0.0841851891763818 |
| H2AC21 | 0.0134503726408765 | 0.0134503726408765 | -0.666828305 | 0.984999604743543 | 0.333855599940739 | 0.745522104138966 |
| H2AX | -0.066695053 | 0.933304947 | -1.21543997 | -0.106121999 | -2.061270473 | 0.124783997152714 |
| H2BC1 | 0.00093384730152904 | 0.00093384730152904 | -0.866426684 | 0.888071453140166 | 0.0253344128441276 | 0.999130434024431 |
| H3-3B | -34.90741385 | -33.90741385 | -13.47748985 | 7.03270186488297 | -0.707106781 | 0.539497285386707 |
| H3-7 | -0.030498423 | 0.969501577 | -0.933149529 | 0.198348731324816 | -1.134013725 | 0.363549164993598 |
| H4-16 | -0.029971807 | 0.970028193 | -0.971204136 | 0.125412142891912 | -1.609716381 | 0.253736524296101 |
| H6PD | 0.0755481660138855 | 0.0755481660138855 | 0.145917151592886 | 1.36798303332769 | 2.47229083915711 | 0.124570854411906 |
| HAAO | 0.0880753814157954 | 0.0880753814157954 | 0.07190231762905 | 1.54178403596967 | 2.0367183180904 | 0.153593928357353 |
| HABP2 | 0.0461484502372307 | 0.0461484502372307 | -0.019654016 | 0.912357250665676 | 1.85358118240654 | 0.1803694486231 |
| HACD2 | -34.67137562 | -33.67137562 | -11.4433828 | 5.97128252171002 | -0.707106781 | 0.539497285386707 |
| HACD3 | -0.008107848 | 0.991892152 | -0.281567979 | 0.11665560240754 | -0.802532474 | 0.520874511364647 |
| HACD4 | 0.0284015849823532 | 0.0284015849823532 | -0.281536318 | 0.790509099455284 | 0.869387491534285 | 0.473406154067073 |
| HADH | 0.00601544729370537 | 0.00601544729370537 | -0.126612489 | 0.247572710995481 | 0.559453645176671 | 0.593071742097792 |
| HADHA | -0.016619458 | 0.983380542 | -0.416892693 | 0.0545254760230174 | -1.515185738 | 0.262891724110501 |
| HADHB | -0.0101508 | 0.9898492 | -0.366970617 | 0.138400075062436 | -0.811434259 | 0.503411547358543 |
| HAGH | -0.010998395 | 0.989001605 | -0.711049775 | 0.482995737966819 | -0.335264296 | 0.745089812636821 |
| HAL | 0 | 1 | NA | NA | NA | NA |
| HAPLN1 | 0.314862118732175 | 0.314862118732175 | 2.66280925389471 | 4.25183846152721 | 7.83276010477073 | 0.00366810752162307 |
| HAPLN3 | 0.205052598142363 | 0.205052598142363 | 1.43924096274275 | 2.74470848977424 | 5.54540390715168 | 0.00766580505806797 |
| HARS1 | -0.031741475 | 0.968258525 | -0.583058466 | -0.073602263 | -2.532153807 | 0.114548183597488 |
| HARS2 | -1.044815244 | -0.044815244 | -18.64920551 | 4.97979981962403 | -1.3012017 | 0.3645121701785 |
| HAT1 | -0.069250598 | 0.930749402 | -1.196884927 | -0.023174281 | -1.881527635 | 0.169877955404372 |
| HAVCR2 | -36.76880567 | -35.76880567 | -13.15910062 | -10.25855456 | -18.16810899 | 0.00518157838812004 |
| HAX1 | -1.766081152 | -0.766081152 | -17.08810041 | 3.66644706994284 | -1.123157805 | 0.365207566604811 |
| HBA2 | 0.0117055652905445 | 0.0117055652905445 | -1.22272088 | 1.53764792815254 | 0.205564834999134 | 0.861611846009304 |
| HBB | 0.0117441747377333 | 0.0117441747377333 | -0.778677836 | 1.07821183348933 | 0.286146932319692 | 0.787679860377166 |
| HBD | 0.00756026001089647 | 0.00756026001089647 | -1.305350674 | 1.4947507249188 | 0.119226731890751 | 0.938107997816854 |
| HBE1 | 0.0691096075255524 | 0.0691096075255524 | -1.115904716 | 2.45650996292222 | 0.67357811828587 | 0.539497285386707 |
| HBG1 | 0.0363291203873613 | 0.0363291203873613 | -2.533995909 | 3.39795123392621 | 0.252146494949513 | 0.81745713797012 |
| HBG2 | 0.100638331409339 | 0.100638331409339 | -3.335835604 | 5.6514910709353 | 0.495869208194924 | 0.640477884824356 |
| HBM | 0.496450283862219 | 0.496450283862219 | -6.247652202 | 14.3595590658704 | 0.856073742511518 | 0.510263089466306 |
| HBQ1 | 0.0751496134158563 | 0.0751496134158563 | -0.497265353 | 1.90060992548978 | 1.0435685753666 | 0.375745937349651 |
| HBS1L | -0.021128781 | 0.978871219 | -0.439382509 | 0.0626505102877743 | -1.383949282 | 0.283708019792457 |
| HBZ | -0.360102304 | 0.639897696 | -14.01256448 | 8.11255916177818 | -0.597151865 | 0.593775163912624 |
| HCCS | 0.0320211835807346 | 0.0320211835807346 | -0.733873615 | 1.33943206527178 | 0.580734831369405 | 0.593071742097792 |
| HCFC1 | -0.031687136 | 0.968312864 | -0.598782103 | -0.048530248 | -2.492391517 | 0.139730274552958 |
| HCK | -1.65269618 | -0.65269618 | -18.41773126 | 4.54330887046806 | -1.046089112 | 0.369272929954607 |
| HCLS1 | -0.10071732 | 0.89928268 | -1.60418709 | -0.467826512 | -3.472593898 | 0.0563223029448156 |
| HDAC1 | -0.026152068 | 0.973847932 | -0.364543738 | -0.148697273 | -4.883428338 | 0.0407524281451239 |
| HDAC2 | 0.0336788524173892 | 0.0336788524173892 | -0.008145318 | 0.637305642967105 | 1.80457980902735 | 0.1803694486231 |
| HDAC3 | 0.0526432871472852 | 0.0526432871472852 | 0.11358371209151 | 0.782951249391041 | 2.72685105127899 | 0.109465529715811 |
| HDAC4 | 1.10012911679281 | 1.10012911679281 | -3.797712382 | 16.3290680966432 | 1.39932753794988 | 0.33940826533785 |
| HDAC6 | 0.0162513860853018 | 0.0162513860853018 | -0.262859682 | 0.564086765938856 | 0.633094375932869 | 0.551061525652585 |
| HDAC7 | 0.437049880297364 | 0.437049880297364 | -6.783881142 | 13.5712210251712 | 0.749811314694139 | 0.539497285386707 |
| HDDC2 | -0.029471881 | 0.970528119 | -1.022820893 | 0.52398573865877 | -0.587074147 | 0.58049773749138 |
| HDDC3 | -0.064029416 | 0.935970584 | -1.296450751 | 0.0590426353697197 | -1.887174384 | 0.187606087491659 |
| HDGF | -0.026264763 | 0.973735237 | -0.48848902 | -0.069536977 | -2.47375242 | 0.103748773709721 |
| HDGFL2 | -0.003511852 | 0.996488148 | -0.27652351 | 0.210914781996715 | -0.236772358 | 0.831478615590815 |
| HDGFL3 | 0.0662539406297435 | 0.0662539406297435 | 0.325308994562753 | 0.997971709991685 | 3.52587801016119 | 0.0423142442798193 |
| HDHD2 | -0.002745291 | 0.997254709 | -0.816551641 | 0.76781467371854 | -0.053347373 | 0.989700356754673 |
| HDHD3 | -0.061396957 | 0.938603043 | -0.867915219 | -0.307322612 | -3.627309348 | 0.0324314269886733 |
| HDHD5 | -0.040389394 | 0.959610606 | -0.541312288 | -0.213089542 | -3.99190101 | 0.0256475213740026 |
| HDLBP | -0.031873731 | 0.968126269 | -0.866006735 | 0.154468271778374 | -1.46768802 | 0.298735355454758 |
| HEATR1 | -0.068562091 | 0.931437909 | -1.118293489 | -0.142382451 | -2.351764495 | 0.108527000377308 |
| HEATR3 | -0.078035946 | 0.921964054 | -1.108913491 | -0.245837266 | -3.118230598 | 0.0810869445560843 |
| HEATR5B | -0.564052361 | 0.435947639 | -14.21327573 | 8.42205696327177 | -0.44530443 | 0.657979673972922 |
| HEATR6 | 0.941135548567463 | 0.941135548567463 | -8.378643086 | 14.0005049007375 | 0.435763433300109 | 0.663048878273489 |
| HEBP1 | 0.00169491219393913 | 0.00169491219393913 | -0.37004723 | 0.405617495333646 | 0.0875216977052125 | 0.967308634406604 |
| HEBP2 | -0.006210433 | 0.993789567 | -0.373875952 | 0.257375803154969 | -0.319557955 | 0.757707468540326 |
| HECTD1 | 0.0893051948385503 | 0.0893051948385503 | -0.633979699 | 2.23440015852376 | 1.13859567304386 | 0.369183133494433 |
| HECTD3 | -0.028752398 | 0.971247602 | -0.555549492 | 0.0221926021776772 | -1.598854642 | 0.189983954626358 |
| HELZ2 | -2.156429498 | -1.156429498 | -19.68017926 | -0.998061751 | -2.405899097 | 0.157235814550523 |
| HEPH | 0.147610076648317 | 0.147610076648317 | 0.847509563424182 | 1.79826411162671 | 6.1950535771232 | 0.0383041704369688 |
| HERC4 | 0.0268408247744196 | 0.0268408247744196 | 0.0163791518103336 | 0.468426285037676 | 1.87654515014429 | 0.159425140216955 |
| HERC6 | 0 | 1 | NA | NA | NA | NA |
| HERPUD1 | -1.284673729 | -0.284673729 | -19.27277028 | 2.36041671200903 | -1.75606908 | 0.240240827503584 |
| HEXA | 0.00906078348833972 | 0.00906078348833972 | -0.158778235 | 0.347990638294266 | 0.646658288598988 | 0.543036874716367 |
| HEXB | -0.012928009 | 0.987071991 | -0.468905108 | 0.191344994426378 | -0.766945867 | 0.529808963818589 |
| HEXIM1 | 0.030380764713757 | 0.030380764713757 | -0.439963228 | 0.976598034687254 | 0.670439589722349 | 0.539497285386707 |
| HFE | 0.0401605530915413 | 0.0401605530915413 | -10.35966973 | 10.8716074919123 | 0.0417282338333785 | 0.994613821823136 |
| HGFAC | 0.668798937986859 | 0.668798937986859 | -8.648977739 | 16.3111376028451 | 0.532080505748376 | 0.612233031829465 |
| HGH1 | 0.0521855388277483 | 0.0521855388277483 | 0.0323023264594641 | 0.903919913310291 | 2.32046919003362 | 0.164681896550719 |
| HGS | 0.0240605724123793 | 0.0240605724123793 | -0.087721962 | 0.562611860672154 | 1.34896602653046 | 0.297577442908415 |
| HGSNAT | 0.0781969047456155 | 0.0781969047456155 | -11.70861265 | 12.3606729774516 | 0.0469068782544657 | 0.99228196233491 |
| HHATL | 35.0281151357041 | 35.0281151357041 | -7.646398271 | 14.6535793891151 | 0.707106781186547 | 0.539497285386707 |
| HIBADH | -0.004458989 | 0.995541011 | -0.297494078 | 0.211400217232416 | -0.335342292 | 0.752425159425364 |
| HIBCH | -0.019745115 | 0.980254885 | -0.560349507 | 0.158042996803688 | -1.091753776 | 0.375653536348272 |
| HID1 | -2.207184836 | -1.207184836 | -21.22367507 | -1.365072777 | -2.556870011 | 0.150816725211032 |
| HIDE1 | -34.80912085 | -33.80912085 | -12.58983017 | 6.56951131753005 | -0.707106781 | 0.539497285386707 |
| HIF1AN | 0.00917332242574918 | 0.00917332242574918 | -10.35326362 | 10.4680153714311 | 0.0095358467907677 | 0.999130434024431 |
| HIKESHI | 0.008079115002004 | 0.008079115002004 | -0.429444407 | 0.568722265347083 | 0.283096483093178 | 0.796961956140589 |
| HINT1 | 0.0131095198435655 | 0.0131095198435655 | -0.226555909 | 0.508558783213702 | 0.710056471455916 | 0.539497285386707 |
| HINT2 | -0.577679577 | 0.422320423 | -15.75391325 | 5.8057016002942 | -1.038138037 | 0.425277260224837 |
| HINT3 | -0.000663017 | 0.999336983 | -11.28269381 | 11.2737107050099 | -0.000689065 | 0.999577493567163 |
| HIP1 | 0.0721087544904366 | 0.0721087544904366 | 0.127397308111762 | 1.13951921834897 | 2.17883458702397 | 0.112158244508424 |
| HIP1R | -1.760882538 | -0.760882538 | -17.57614672 | 3.79524754992982 | -1.119697122 | 0.366275438800159 |
| HK1 | 0.00603960510913286 | 0.00603960510913286 | -0.063318725 | 0.196437801849578 | 0.963530752891021 | 0.424267295692596 |
| HK2 | -1.980885223 | -0.980885223 | -20.93275577 | 0.798932458418483 | -2.083476616 | 0.182063758237489 |
| HK3 | -2.088213638 | -1.088213638 | -21.02159425 | -0.244757676 | -2.296722115 | 0.175024732685753 |
| HLA-A | -0.030727541 | 0.969272459 | -1.278694762 | 0.611413448069092 | -0.676272311 | 0.539497285386707 |
| HLA-B | -0.079321676 | 0.920678324 | -1.795345308 | 0.108836940458854 | -1.8407515 | 0.198354309528674 |
| HLA-C | -0.062619068 | 0.937380932 | -1.252403334 | -0.057158527 | -1.971426081 | 0.153033087846879 |
| HLA-DMB | -1.051127897 | -0.051127897 | -18.15740294 | 4.75918240094475 | -1.30963103 | 0.3619762450857 |
| HLA-DPA1 | -0.017879479 | 0.982120521 | -0.68575988 | 0.34637379495977 | -0.709249104 | 0.539497285386707 |
| HLA-DPB1 | 0.0211785890178948 | 0.0211785890178948 | -0.681580868 | 1.0769237200788 | 0.392532934764051 | 0.695329037615816 |
| HLA-DQA1 | -0.057236198 | 0.942763802 | -1.831352257 | 0.86462245395831 | -0.628402245 | 0.554353627193476 |
| HLA-DQB1 | -0.055051769 | 0.944948231 | -1.645977481 | 0.533055153032347 | -1.012931673 | 0.410037204425534 |
| HLA-DRA | -0.034381274 | 0.965618726 | -1.024758273 | 0.306185855411872 | -0.934158099 | 0.420656783472752 |
| HLA-DRB1 | -0.057386889 | 0.942613111 | -13.23958758 | 12.3583039142258 | -0.059591761 | 0.986728246797556 |
| HLA-DRB3 | -0.442113475 | 0.557886525 | -14.59345104 | 7.21935616474714 | -0.7566691 | 0.539497285386707 |
| HLA-DRB4 | -0.031493033 | 0.968506967 | -12.68841684 | 12.2177700448001 | -0.032698923 | 0.998068903333744 |
| HLA-DRB5 | 0 | 1 | NA | NA | NA | NA |
| HLA-E | -0.017965209 | 0.982034791 | -0.682710783 | 0.349197460234187 | -0.619894839 | 0.566398099030184 |
| HLA-F | 0.392594408573186 | 0.392594408573186 | -7.428207733 | 13.6720551297297 | 0.661084919399877 | 0.560880133528299 |
| HLA-G | -0.023318717 | 0.976681283 | -13.4648264 | 13.3396412428036 | -0.008081212 | 0.999130434024431 |
| HLA-H | 0.305903342873418 | 0.305903342873418 | -8.974282723 | 14.1218396745593 | 0.499867470117419 | 0.647590537137961 |
| HM13 | -0.141209738 | 0.858790262 | -1.676403274 | -1.246072956 | -11.75138786 | 0.000273271710161686 |
| HMBS | 0.0262696609816779 | 0.0262696609816779 | -0.410802229 | 0.926740980315671 | 0.704492656583654 | 0.539497285386707 |
| HMCES | -2.104828191 | -1.104828191 | -18.40550901 | -0.350397783 | -2.331951484 | 0.17224744541596 |
| HMCN1 | 0.102293087078394 | 0.102293087078394 | 0.426927260031525 | 1.58346882232658 | 3.03585007631238 | 0.0531501602223687 |
| HMG20A | -0.056197506 | 0.943802494 | -13.11539137 | 12.6143380706929 | -0.033705652 | 0.99766937227783 |
| HMG20B | -34.64998628 | -33.64998628 | -11.27497517 | 5.88340557441445 | -0.707106781 | 0.539497285386707 |
| HMGA1 | -0.601225479 | 0.398774521 | -16.19868119 | 5.59310759027966 | -1.093346877 | 0.400909502654508 |
| HMGB1 | -0.076698031 | 0.923301969 | -1.35158916 | -0.148122714 | -2.66533209 | 0.127190155013202 |
| HMGB2 | -0.090029324 | 0.909970676 | -1.437808738 | -0.397696819 | -3.309394068 | 0.0579571478363869 |
| HMGB3 | -0.960814646 | 0.039185354 | -19.2783638 | 6.16576538483737 | -1.157858926 | 0.379662749797453 |
| HMGCL | -0.022810509 | 0.977189491 | -0.374991349 | -0.054427492 | -2.446059604 | 0.100973219460144 |
| HMGCR | 0 | 1 | NA | NA | NA | NA |
| HMGCS1 | 0.119438746777722 | 0.119438746777722 | -13.21347516 | 14.3476440632536 | 0.0713077245550498 | 0.977309112895896 |
| HMGN1 | -0.069055946 | 0.930944054 | -1.247941084 | -0.015927286 | -1.788917052 | 0.172764906484805 |
| HMGN2 | -1.522033252 | -0.522033252 | -15.40912149 | 4.44702301767796 | -0.955634408 | 0.407742429843282 |
| HMGN4 | -0.082141551 | 0.917858449 | -1.518039789 | -0.185983438 | -2.465730728 | 0.11469818241699 |
| HMGN5 | 0.985484983680738 | 0.985484983680738 | -5.284689006 | 17.4048994566176 | 1.19868169651859 | 0.36789104110623 |
| HMOX1 | -0.116431123 | 0.883568877 | -1.957241313 | -0.181375913 | -2.250323026 | 0.127174561584654 |
| HMOX2 | -0.027480329 | 0.972519671 | -0.989531592 | 0.41578887399451 | -0.89598046 | 0.491705776536746 |
| HNMT | 0.0100717856631639 | 0.0100717856631639 | -0.205878256 | 0.40364912376155 | 0.702401062557513 | 0.539497285386707 |
| HNRNPA0 | 0.0148070621439402 | 0.0148070621439402 | -0.128436492 | 0.430895505926675 | 0.99936529516524 | 0.401213750626867 |
| HNRNPA1 | -0.064032374 | 0.935967626 | -1.066740326 | -0.280106958 | -3.154842496 | 0.0603462894706014 |
| HNRNPA2B1 | -0.032116175 | 0.967883825 | -0.704156229 | -0.067249202 | -2.249125176 | 0.12509114118925 |
| HNRNPA3 | -0.014855638 | 0.985144362 | -0.581554325 | 0.254690754027253 | -0.677108728 | 0.539497285386707 |
| HNRNPAB | -0.009026961 | 0.990973039 | -0.407092542 | 0.241867655702517 | -0.516626293 | 0.632590957913431 |
| HNRNPC | -0.031823437 | 0.968176563 | -0.809739519 | 0.0551588094171063 | -1.514445483 | 0.215065608732433 |
| HNRNPD | -0.033356547 | 0.966643453 | -0.674320472 | -0.067261087 | -2.114126811 | 0.117985257479168 |
| HNRNPDL | 0.011013586853012 | 0.011013586853012 | -0.232999809 | 0.473360672519418 | 0.600851781480406 | 0.569057716596462 |
| HNRNPF | -0.034126503 | 0.965873497 | -0.682219325 | -0.063708535 | -2.14543714 | 0.123694468406154 |
| HNRNPH1 | -0.041609099 | 0.958390901 | -0.777403662 | -0.072925121 | -2.089177625 | 0.120980763005907 |
| HNRNPH2 | -0.002759294 | 0.997240706 | -0.617820802 | 0.56326087857262 | -0.096996429 | 0.960140269786056 |
| HNRNPH3 | -0.014258711 | 0.985741289 | -0.564049397 | 0.26670311028262 | -0.707286602 | 0.539497285386707 |
| HNRNPK | -0.0164621 | 0.9835379 | -0.509780603 | 0.141615329038802 | -1.083438155 | 0.375560518269785 |
| HNRNPL | -0.021469404 | 0.978530596 | -0.54982904 | 0.0909729795482504 | -1.252570473 | 0.317164124655671 |
| HNRNPLL | 0.045003026908677 | 0.045003026908677 | -0.119244683 | 0.912452860751614 | 1.48510307079156 | 0.264948297539786 |
| HNRNPM | -0.027139495 | 0.972860505 | -0.798057625 | 0.194301770786565 | -1.059129846 | 0.36731973075278 |
| HNRNPR | -0.003604801 | 0.996395199 | -0.250212007 | 0.17236347601611 | -0.328316223 | 0.752425159425364 |
| HNRNPU | -0.022356566 | 0.977643434 | -0.533005793 | 0.0201023802054905 | -1.772446202 | 0.185286070433541 |
| HNRNPUL1 | -0.042435806 | 0.957564194 | -0.61702975 | -0.212260102 | -4.256760517 | 0.0538679412155676 |
| HNRNPUL2 | -0.028903734 | 0.971096266 | -0.553609925 | -0.048741038 | -2.247539599 | 0.129450833435746 |
| HOMER3 | -35.79473686 | -34.79473686 | -16.91634211 | 4.99515577850789 | -1.224315918 | 0.366275438800159 |
| HOOK1 | -36.46658074 | -35.46658074 | -19.67001127 | 0.678276451903566 | -2.100307363 | 0.180716766229804 |
| HOOK3 | 0.0409653038315576 | 0.0409653038315576 | 0.0342009728698353 | 0.723336025237623 | 2.25129973288128 | 0.157235814550523 |
| HOPX | -0.930633045 | 0.069366955 | -15.40643354 | 9.27378511829573 | -0.430886336 | 0.66648894933017 |
| HP | 0.0432742250059759 | 0.0432742250059759 | -0.550134709 | 1.6175280123129 | 0.853140784463695 | 0.471297098899403 |
| HP1BP3 | 0.0263303511391367 | 0.0263303511391367 | -0.033920008 | 0.636535254823786 | 1.66083823771164 | 0.198354309528674 |
| HPCAL1 | -0.367161858 | 0.632838142 | -13.1396195 | 7.49702117434473 | -0.611372656 | 0.585817099552445 |
| HPF1 | -1.029861607 | -0.029861607 | -17.26862891 | 4.76570621276722 | -1.274574475 | 0.366275438800159 |
| HPGD | -5.39E-05 | 1.00E+00 | -12.18170562 | 12.1809166330201 | -5.60E-05 | 0.999939341811618 |
| HPGDS | -1.497435316 | -0.497435316 | -17.06541287 | 5.08677331529293 | -0.936286645 | 0.419491455638605 |
| HPR | 0.0406820876950496 | 0.0406820876950496 | -0.550986447 | 1.29077639451808 | 0.699832249655426 | 0.539497285386707 |
| HPRT1 | 0.0291480471362981 | 0.0291480471362981 | -0.165468493 | 0.731140192266798 | 1.16606278916806 | 0.364559456625742 |
| HPS5 | 0.400683258298164 | 0.400683258298164 | -6.762152151 | 12.6091540241542 | 0.678806296689334 | 0.552716415991246 |
| HPS6 | 1.03620142870921 | 1.03620142870921 | -4.121648899 | 15.1577247185072 | 1.28587410525557 | 0.366275438800159 |
| HPSE | 0 | 1 | NA | NA | NA | NA |
| HPX | 0.066016351370128 | 0.066016351370128 | -0.024422287 | 1.56940370612891 | 1.81302579468977 | 0.1803694486231 |
| HRAS | 0.623765326551197 | 0.623765326551197 | -8.353474541 | 15.0162188259269 | 0.494918205830527 | 0.634031727574291 |
| HRC | 0 | 1 | NA | NA | NA | NA |
| HRG | 0.095419515662963 | 0.095419515662963 | 0.503096207584917 | 1.51312672029785 | 3.5502982131734 | 0.0402744596888282 |
| HRNR | 1.11266271992794 | 1.11266271992794 | -7.636193992 | 13.9824980966273 | 0.513849898187879 | 0.623527893026044 |
| HS1BP3 | 0.00229112706594167 | 0.00229112706594167 | -0.249063354 | 0.292824053236361 | 0.140210789397888 | 0.91890140379679 |
| HSBP1 | 0.0611418636237685 | 0.0611418636237685 | 0.0503852980071797 | 1.15036433124977 | 2.01301461891417 | 0.155466238836064 |
| HSCB | -2.006677488 | -1.006677488 | -20.76363796 | 0.535217580322169 | -2.132220219 | 0.1803694486231 |
| HSD11B1 | -34.81590154 | -33.81590154 | -12.64914183 | 6.60046079394258 | -0.707106781 | 0.539497285386707 |
| HSD17B10 | -0.048934969 | 0.951065031 | -0.892095154 | -0.188410825 | -3.387022459 | 0.0919573023622265 |
| HSD17B11 | -0.055372971 | 0.944627029 | -0.79557998 | -0.275476984 | -3.776444045 | 0.0407413914760697 |
| HSD17B12 | -0.03125775 | 0.96874225 | -0.68869413 | 0.0729140167387183 | -1.637342741 | 0.235998473152946 |
| HSD17B14 | -34.86238339 | -33.86238339 | -13.06331783 | 6.81658236659151 | -0.707106781 | 0.539497285386707 |
| HSD17B4 | -0.042529889 | 0.957470111 | -0.747890629 | -0.169933648 | -2.896300196 | 0.0704375074851147 |
| HSD17B7 | -1.702094252 | -0.702094252 | -16.98055757 | 3.94047349193109 | -1.080175375 | 0.366275438800159 |
| HSD17B8 | -0.534630524 | 0.465369476 | -14.61404126 | 5.92656750391751 | -0.951264914 | 0.468527460568712 |
| HSD3B1 | 0 | 1 | NA | NA | NA | NA |
| HSDL1 | -0.001354428 | 0.998645572 | -0.408567476 | 0.383071999827264 | -0.05585469 | 0.987958171023924 |
| HSDL2 | 0.0057653992031031 | 0.0057653992031031 | -0.195504995 | 0.309512121243424 | 0.479064205225258 | 0.653774096383628 |
| HSF1 | -0.589207946 | 0.410792054 | -14.84399271 | 8.56442080043186 | -0.466361486 | 0.649896707856798 |
| HSP90AA1 | 0.000913065528062695 | 0.000913065528062695 | -0.46606574 | 0.485878455476852 | 0.0428118525388874 | 0.994613821823136 |
| HSP90AA4P | -35.86706139 | -34.86706139 | -17.78211033 | 5.24806303442169 | -1.224729585 | 0.366275438800159 |
| HSP90AB1 | 0.00484199650216849 | 0.00484199650216849 | -0.532571471 | 0.643623155775542 | 0.204402321440684 | 0.868201417463026 |
| HSP90AB2P | 0.401896560311541 | 0.401896560311541 | -7.19034851 | 13.438465838813 | 0.679523798322289 | 0.552358417618403 |
| HSP90AB4P | -0.38256311 | 0.61743689 | -13.66616021 | 7.58091004526547 | -0.64354094 | 0.568601960142693 |
| HSP90B1 | -0.104540542 | 0.895459458 | -1.768728773 | -0.863906066 | -5.064408723 | 0.0119780099521387 |
| HSPA12A | 0.0988176270800312 | 0.0988176270800312 | 0.222982577233143 | 1.61759629697303 | 2.2838413519173 | 0.0993457544474214 |
| HSPA12B | 0.131851588179098 | 0.131851588179098 | 0.861379052026563 | 1.83906863356285 | 5.08477186373251 | 0.0202272866524185 |
| HSPA13 | -0.155636535 | 0.844363465 | -1.965911307 | -1.065907675 | -6.089119049 | 0.00984451386004276 |
| HSPA14 | 0.0251911265668434 | 0.0251911265668434 | -0.102664171 | 0.587115964653237 | 1.25764391720563 | 0.323635202081789 |
| HSPA2 | 0.127358832838138 | 0.127358832838138 | 0.889790852921775 | 1.55012340613281 | 6.80823644693992 | 0.00850003363500947 |
| HSPA4 | 0.00207800372657644 | 0.00207800372657644 | -0.270693196 | 0.3120556449676 | 0.151393612352687 | 0.913463238008119 |
| HSPA4L | 1.7031951973973 | 1.7031951973973 | -4.122579851 | 17.7656500049016 | 1.08026612407487 | 0.366275438800159 |
| HSPA5 | -0.090984786 | 0.909015214 | -1.504879471 | -0.811590675 | -5.864903564 | 0.00758996613728495 |
| HSPA8 | 0.00889609889301551 | 0.00889609889301551 | -0.269251646 | 0.481852797293625 | 0.614249957083992 | 0.581107383260531 |
| HSPA9 | -0.046989253 | 0.953010747 | -0.833798725 | -0.224539709 | -3.45989391 | 0.0674940977926953 |
| HSPB1 | 0.0326759383784359 | 0.0326759383784359 | -0.279112833 | 1.03320671418888 | 1.03911005323676 | 0.380452616321765 |
| HSPB11 | -1.566942709 | -0.566942709 | -16.92821234 | 4.62677275656835 | -0.987479067 | 0.39247027574547 |
| HSPB2 | 0 | 1 | NA | NA | NA | NA |
| HSPB3 | 34.9972287851752 | 34.9972287851752 | -7.484438033 | 14.343198328093 | 0.707106781186547 | 0.539497285386707 |
| HSPB6 | -0.380681788 | 0.619318212 | -14.49464788 | 8.0788011551141 | -0.637469151 | 0.57118509852219 |
| HSPB7 | 0 | 1 | NA | NA | NA | NA |
| HSPB8 | 0 | 1 | NA | NA | NA | NA |
| HSPBP1 | 0.0189424400210647 | 0.0189424400210647 | -0.158462152 | 0.504625783019175 | 0.987162748712562 | 0.411929838964859 |
| HSPD1 | -0.051413838 | 0.948586162 | -0.888922724 | -0.31324243 | -3.652249181 | 0.0343284158775903 |
| HSPE1 | -0.065948685 | 0.934051315 | -1.111518509 | -0.401661761 | -4.358395074 | 0.0491647079324609 |
| HSPG2 | 0.0992107631315714 | 0.0992107631315714 | 0.491382565946063 | 1.66452564266881 | 3.3874932826201 | 0.0531018349927428 |
| HSPH1 | -0.029090708 | 0.970909292 | -0.509270758 | -0.031776215 | -2.016873429 | 0.141261291798785 |
| HTATIP2 | -1.023219342 | -0.023219342 | -19.11600683 | 5.35647079338442 | -1.263853455 | 0.366275438800159 |
| HTATSF1 | -0.025346302 | 0.974653698 | -0.700327173 | 0.23276990431316 | -1.036166074 | 0.407945710828718 |
| HTRA1 | 0.122690450692951 | 0.122690450692951 | 0.163996548886554 | 2.16632762347643 | 2.34708590058266 | 0.14001882095693 |
| HTRA2 | 0.0143070934009147 | 0.0143070934009147 | -0.336624984 | 0.59962758228987 | 0.526211899129831 | 0.621704967022904 |
| HTRA3 | 0 | 1 | NA | NA | NA | NA |
| HTT | -0.406563865 | 0.593436135 | -13.76747694 | 7.30006133161569 | -0.690176366 | 0.54733731056867 |
| HUWE1 | 0.00410948860348441 | 0.00410948860348441 | -0.217755494 | 0.298400634186494 | 0.299913532704445 | 0.780215147617436 |
| HVCN1 | -0.085425384 | 0.914574616 | -1.379637407 | -0.312455663 | -2.744173278 | 0.0642688422145678 |
| HYI | -0.000349289 | 0.999650711 | -0.8834728 | 0.877115466620286 | -0.006948519 | 0.999130434024431 |
| HYOU1 | -0.111458041 | 0.888541959 | -1.563278038 | -0.831919605 | -5.677435954 | 0.00736042678840189 |
| HYPK | -0.405837916 | 0.594162084 | -12.32002812 | 6.53378021916149 | -0.686283363 | 0.548813468638075 |
| IAH1 | 0.0261161325730299 | 0.0261161325730299 | -0.107877803 | 0.626747699293722 | 1.30677466248661 | 0.31415740132249 |
| IARS1 | -0.027675062 | 0.972324938 | -0.859044245 | 0.232388251268891 | -1.110250438 | 0.368346702519642 |
| IARS2 | -0.01884622 | 0.98115378 | -0.513778593 | 0.134516755894037 | -1.277715225 | 0.364688913286397 |
| IBA57 | -0.032960618 | 0.967039382 | -0.570645913 | -0.077244776 | -2.35718276 | 0.103748773709721 |
| IBSP | 0 | 1 | NA | NA | NA | NA |
| ICAM1 | -0.090048934 | 0.909951066 | -1.356246903 | -0.434752985 | -3.89043134 | 0.0548717713947671 |
| ICAM2 | -0.992335968 | 0.007664032 | -15.61966064 | 9.08961535792244 | -0.459367521 | 0.65324940354771 |
| ICAM3 | -1.137364571 | -0.137364571 | -20.33902004 | 4.26963138323272 | -1.469213529 | 0.317420650050733 |
| ICAM4 | 0.923106255880902 | 0.923106255880902 | -7.735803995 | 12.7976450592284 | 0.4273756979575 | 0.668270163327605 |
| ICMT | 0.00594093163278339 | 0.00594093163278339 | -0.226687806 | 0.327235729634861 | 0.319134396642688 | 0.758716629421548 |
| IDE | -0.06915146 | 0.93084854 | -0.903832182 | -0.431307557 | -4.899659948 | 0.0127799126650757 |
| IDH1 | 0.0116201873685181 | 0.0116201873685181 | -0.048322764 | 0.300382419722663 | 1.26487470117077 | 0.312367517497055 |
| IDH2 | -0.04597029 | 0.95402971 | -0.8703802 | -0.143352729 | -2.894575957 | 0.103748773709721 |
| IDH3A | -0.079108625 | 0.920891375 | -1.195559851 | -0.433098431 | -4.775233998 | 0.056017830176136 |
| IDH3B | -0.080722256 | 0.919277744 | -1.135827305 | -0.502019199 | -4.93952695 | 0.0285249016734406 |
| IDH3G | -0.505179219 | 0.494820781 | -15.36548247 | 6.66214375753388 | -0.888764135 | 0.499173160405125 |
| IDI1 | -36.37333242 | -35.37333242 | -18.34774961 | 0.544712546036326 | -2.120565161 | 0.1803694486231 |
| IDO1 | 0 | 1 | NA | NA | NA | NA |
| IDUA | -0.019511332 | 0.980488668 | -0.74154819 | 0.352911758675904 | -0.672633718 | 0.539497285386707 |
| IER3IP1 | 0.00905910184392182 | 0.00905910184392182 | -0.358825918 | 0.537218357947116 | 0.400817063027262 | 0.698085798188918 |
| IFI16 | -0.021930291 | 0.978069709 | -0.472759503 | 0.0331841893450347 | -1.740038095 | 0.207474063571298 |
| IFI27 | -35.03224407 | -34.03224407 | -14.69557743 | 7.66831331065759 | -0.707106781 | 0.539497285386707 |
| IFI30 | -1.069750328 | -0.069750328 | -19.90375053 | 4.98446623023054 | -1.343045675 | 0.354726159265742 |
| IFI35 | -0.02663444 | 0.97336556 | -0.427141013 | -0.070512141 | -2.515050519 | 0.0900859252365181 |
| IFI44 | 1.91144939759219 | 1.91144939759219 | -1.384918939 | 20.0682580510102 | 1.94742940891518 | 0.200644118003916 |
| IFI44L | 0.565893416732723 | 0.565893416732723 | -9.267814645 | 15.6754197359634 | 0.447087121377497 | 0.657423743030449 |
| IFIH1 | -1.254829545 | -0.254829545 | -18.11872459 | 7.0978324095601 | -0.76498005 | 0.524475843328539 |
| IFIT1 | 1.04059679591578 | 1.04059679591578 | -5.209640943 | 19.2983691322332 | 1.29184322118703 | 0.366275438800159 |
| IFIT2 | 1.5858953080678 | 1.5858953080678 | -5.151303671 | 19.2806141487185 | 1.00061449362999 | 0.386284743929022 |
| IFIT3 | 0.0634606629317818 | 0.0634606629317818 | -0.603847603 | 1.75611316794041 | 0.89913877381309 | 0.456902874401019 |
| IFIT5 | 0.0303665903427299 | 0.0303665903427299 | 0.0550318705323978 | 0.526285659193972 | 2.6373567981761 | 0.129182555040176 |
| IFITM3 | 0.0188287594462429 | 0.0188287594462429 | -9.760968213 | 9.98333417187247 | 0.0194864804862305 | 0.999130434024431 |
| IFITM5 | -34.93971305 | -33.93971305 | -13.78262829 | 7.19192644326037 | -0.707106781 | 0.539497285386707 |
| IFT27 | -0.431759536 | 0.568240464 | -13.77264531 | 6.95351916296835 | -0.739322529 | 0.539497285386707 |
| IGBP1 | -0.012791278 | 0.987208722 | -0.302538055 | 0.0646523036992938 | -1.14860518 | 0.3619762450857 |
| IGDCC4 | 0.642592146285526 | 0.642592146285526 | -8.491868381 | 15.5707784081169 | 0.510347398764261 | 0.625102839632804 |
| IGF1 | 1.11674157584877 | 1.11674157584877 | -3.546722522 | 16.2652825854834 | 1.42010696250745 | 0.328418638149916 |
| IGF2 | 0.0471739321156766 | 0.0471739321156766 | -0.213525691 | 1.17479582589456 | 1.26984624267442 | 0.326086253195408 |
| IGF2BP1 | 0 | 1 | NA | NA | NA | NA |
| IGF2BP2 | 1.09949379818053 | 1.09949379818053 | -4.289214533 | 18.4625117709709 | 1.39781863171076 | 0.33940826533785 |
| IGF2BP3 | 0.64842857789066 | 0.64842857789066 | -7.350838538 | 13.5043116097617 | 0.511868497158014 | 0.623941853775364 |
| IGF2R | 0.00524662309701698 | 0.00524662309701698 | -0.302403758 | 0.40713385070069 | 0.267202171090373 | 0.80544667182555 |
| IGFALS | 0.0862406508331576 | 0.0862406508331576 | 0.475159781330801 | 1.22408857750264 | 3.92932218339501 | 0.0262489120838967 |
| IGFBP2 | 0.607473832179094 | 0.607473832179094 | -8.508622081 | 15.0340273163487 | 0.481470212499517 | 0.64191931012684 |
| IGFBP3 | 0.0892885259413987 | 0.0892885259413987 | 0.220007950706503 | 1.46765827321496 | 2.34485977993503 | 0.0926613647437377 |
| IGFBP5 | 0.159141746259978 | 0.159141746259978 | 0.653085396396163 | 2.59003195019258 | 3.17228201755045 | 0.0670680593256903 |
| IGFBP6 | 34.8705252239919 | 34.8705252239919 | -6.855160455 | 13.1372489884923 | 0.707106781186548 | 0.539497285386707 |
| IGFBP7 | 0.0324728852301744 | 0.0324728852301744 | -0.108188656 | 0.703956409971099 | 1.28200947224669 | 0.304409095912808 |
| IGFL1 | 0 | 1 | NA | NA | NA | NA |
| IGFLR1 | -1.636842547 | -0.636842547 | -16.98509325 | 4.26929356781414 | -1.035473394 | 0.37351506440868 |
| IGHA1 | -0.002456231 | 0.997543769 | -0.99076217 | 0.929149515480064 | -0.060128966 | 0.986728246797556 |
| IGHA2 | -0.010242179 | 0.989757821 | -1.44968291 | 1.24290717802515 | -0.1347688 | 0.924453979548372 |
| IGHD | 0.173723852825519 | 0.173723852825519 | 0.401804788815913 | 2.91709710288934 | 2.83721858996971 | 0.115722268902765 |
| IGHG1 | -0.012464203 | 0.987535797 | -0.919107742 | 0.583331554521079 | -0.401274035 | 0.689884853141824 |
| IGHG2 | -0.001014688 | 0.998985312 | -1.22279354 | 1.19638072498506 | -0.02445414 | 0.999130434024431 |
| IGHG3 | 0.0427870934479761 | 0.0427870934479761 | -0.696127151 | 1.74514825199581 | 0.95155420373234 | 0.465233794098861 |
| IGHG4 | 0.0236615168990246 | 0.0236615168990246 | -1.344813281 | 1.87947303887779 | 0.327920200659937 | 0.757707468540326 |
| IGHM | 0.0339610265353434 | 0.0339610265353434 | -0.336167519 | 1.14127552548394 | 0.991185554645589 | 0.401634162957962 |
| IGHV1-18 | 0.0520564291099166 | 0.0520564291099166 | -0.541828173 | 1.50612678068591 | 0.887921729491617 | 0.468423818184853 |
| IGHV1-2 | -0.732820058 | 0.267179942 | -16.16695234 | 8.02914831970863 | -0.582061184 | 0.57827242635708 |
| IGHV1-3 | 0.031554558101294 | 0.031554558101294 | -0.553088598 | 1.1187500942937 | 0.68905584856595 | 0.539497285386707 |
| IGHV1-45 | -0.380658514 | 0.619341486 | -12.3269943 | 6.78714875638408 | -0.628486378 | 0.573246848336326 |
| IGHV1-46 | -0.061435868 | 0.938564132 | -1.567571164 | 0.478905104543057 | -1.122156139 | 0.379841861092033 |
| IGHV1-58 | 0.602235880936511 | 0.602235880936511 | -8.352198735 | 14.6616091426451 | 0.476416440063087 | 0.644860960250888 |
| IGHV1-69 | 0.0666949788243316 | 0.0666949788243316 | -1.033118511 | 2.2876177539391 | 0.816412041447492 | 0.528014169078056 |
| IGHV1-69D | 34.8646111232919 | 34.8646111232919 | -6.827116327 | 13.0835051414541 | 0.707106781186548 | 0.539497285386707 |
| IGHV1-8 | 0 | 1 | NA | NA | NA | NA |
| IGHV2-26 | 0.480548145371895 | 0.480548145371895 | -6.797085606 | 14.7372642206263 | 0.829381331304245 | 0.52730639626287 |
| IGHV2-5 | 0.396665300044694 | 0.396665300044694 | -7.365428393 | 13.6077171565421 | 0.665977408867424 | 0.558116976763512 |
| IGHV2-70D | 0.47213735827747 | 0.47213735827747 | -7.62303372 | 16.3805415705058 | 0.793572386804497 | 0.537375417204127 |
| IGHV3-13 | 0.0520084237119325 | 0.0520084237119325 | -0.22311286 | 1.23464298187695 | 1.21523576835364 | 0.334509726783066 |
| IGHV3-15 | -0.005500846 | 0.994499154 | -1.174327737 | 1.07369765747581 | -0.080999417 | 0.970803346798997 |
| IGHV3-35 | -0.000280986 | 0.999719014 | -1.341958297 | 1.33605649291938 | -0.004182566 | 0.999130434024431 |
| IGHV3-38 | -0.019784234 | 0.980215766 | -1.20501053 | 0.825754803259497 | -0.362240831 | 0.728167240765905 |
| IGHV3-43 | 0.381530294468574 | 0.381530294468574 | -7.512763145 | 13.5052383147484 | 0.641127000874383 | 0.569664374168477 |
| IGHV3-49 | 0.0259697876869072 | 0.0259697876869072 | -1.46066335 | 1.98404529259437 | 0.291255608641969 | 0.78705086035764 |
| IGHV3-64 | 0.609456647348265 | 0.609456647348265 | -8.64047578 | 15.2928929223315 | 0.482817278687153 | 0.64104460116308 |
| IGHV3-64D | -0.01284655 | 0.98715345 | -1.028080223 | 0.783406087535457 | -0.234134929 | 0.832925925519797 |
| IGHV3-7 | -0.01081976 | 0.98918024 | -1.519099181 | 1.28383927750554 | -0.169497269 | 0.898252563509827 |
| IGHV3-72 | 1.0298327189589 | 1.0298327189589 | -5.144716108 | 18.8745398660012 | 1.26634219714162 | 0.366275438800159 |
| IGHV3-74 | -0.044334749 | 0.955665251 | -1.574296184 | 0.707863426391151 | -0.781510547 | 0.536872666939829 |
| IGHV4-28 | 0.0144517119644074 | 0.0144517119644074 | -0.792757113 | 1.09780824790779 | 0.296956653542347 | 0.780524805218151 |
| IGHV4-4 | 0.0184258466048071 | 0.0184258466048071 | -2.244024723 | 2.60010756579145 | 0.157577006418145 | 0.909648966486998 |
| IGHV5-51 | -0.036782519 | 0.963217481 | -1.957439799 | 1.25078804705022 | -0.469988571 | 0.656930483488738 |
| IGHV6-1 | 0.0428080263273089 | 0.0428080263273089 | -0.834804408 | 1.78811333578405 | 0.665202480745074 | 0.539497285386707 |
| IGKC | -0.01392974 | 0.98607026 | -0.787926753 | 0.434634185008207 | -0.555653741 | 0.606541685300831 |
| IGKJ1 | 0.00784188508754019 | 0.00784188508754019 | -0.862260131 | 1.01953023566657 | 0.151780554133494 | 0.910854154984341 |
| IGKV1-16 | 0.0316201064565479 | 0.0316201064565479 | -0.519715677 | 1.10147527669689 | 0.72842324237224 | 0.539497285386707 |
| IGKV1-17 | -0.02762586 | 0.97237414 | -1.090811692 | 0.521735303324614 | -0.610754485 | 0.561683424906805 |
| IGKV1-27 | -35.93972015 | -34.93972015 | -18.73200954 | 5.55054101771494 | -1.221562191 | 0.366275438800159 |
| IGKV1-5 | -0.018946184 | 0.981053816 | -1.03237386 | 0.612589510554446 | -0.441754467 | 0.65992821134264 |
| IGKV1-8 | -34.89005099 | -33.89005099 | -13.31626017 | 6.94857044890936 | -0.707106781 | 0.539497285386707 |
| IGKV1D-16 | 0.974517566747085 | 0.974517566747085 | -6.143222001 | 19.7680737922386 | 1.18072454899831 | 0.37350198937203 |
| IGKV2-29 | -36.52772228 | -35.52772228 | -20.44140664 | 0.627501641024374 | -2.116284889 | 0.1803694486231 |
| IGKV2-30 | -1.657637419 | -0.657637419 | -19.62546166 | 4.81444461572021 | -1.049230679 | 0.368125003866442 |
| IGKV2D-29 | 0.0129776469571035 | 0.0129776469571035 | -11.40751218 | 11.5867157992305 | 0.0134846259357742 | 0.999130434024431 |
| IGKV3-20 | -0.029101871 | 0.970898129 | -1.565949664 | 0.834084478132514 | -0.547100835 | 0.605507690553716 |
| IGKV3D-15 | 0.00936510410376965 | 0.00936510410376965 | -2.3708144 | 2.57689628120995 | 0.088959444197215 | 0.967308634406604 |
| IGKV3D-20 | -0.037699773 | 0.962300227 | -1.309708946 | 0.45055794565809 | -0.854892769 | 0.472617674700726 |
| IGKV4-1 | -0.039285157 | 0.960714843 | -1.040300848 | 0.228938387097538 | -1.204819095 | 0.357404285547567 |
| IGKV6-21 | 0.586685459893107 | 0.586685459893107 | -8.908140177 | 15.3820914466231 | 0.463299582327244 | 0.652016273769328 |
| IGLC7 | -0.496010111 | 0.503989889 | -13.98029486 | 6.11664433051572 | -0.860004468 | 0.50995353643859 |
| IGLL1 | 0.440405879681474 | 0.440405879681474 | -6.647304632 | 13.3915365042122 | 0.754286444324065 | 0.539497285386707 |
| IGLL5 | -0.01307647 | 0.98692353 | -0.816891127 | 0.513119100643902 | -0.395270627 | 0.691968313323585 |
| IGLV1-36 | 0.011513232277222 | 0.011513232277222 | -0.545235822 | 0.738232374711016 | 0.2957482600363 | 0.784565673749586 |
| IGLV1-40 | -0.003433033 | 0.996566967 | -1.578906956 | 1.51280921453138 | -0.040444951 | 0.995327285436105 |
| IGLV1-47 | -0.008249843 | 0.991750157 | -0.726877053 | 0.536581745051309 | -0.267270127 | 0.804396581371602 |
| IGLV1-51 | -0.048763749 | 0.951236251 | -1.198321783 | 0.129170141324699 | -1.461891903 | 0.249425916482577 |
| IGLV10-54 | 1.12369964406759 | 1.12369964406759 | -3.712315228 | 16.9282906142753 | 1.43613203318538 | 0.326967877556403 |
| IGLV2-11 | -0.148531158 | 0.851468842 | -2.135623936 | -0.70214634 | -3.473829464 | 0.0398192221657769 |
| IGLV2-14 | 0 | 1 | NA | NA | NA | NA |
| IGLV2-18 | -0.026169418 | 0.973830582 | -1.651249288 | 1.16725721781453 | -0.332747744 | 0.753052916356947 |
| IGLV2-23 | -0.469083863 | 0.530916137 | -14.35009363 | 6.69290509349959 | -0.809824193 | 0.533898790187029 |
| IGLV2-8 | -0.571161204 | 0.428838796 | -16.65566042 | 9.80448727972993 | -0.450441595 | 0.656201994982807 |
| IGLV3-1 | 0 | 1 | NA | NA | NA | NA |
| IGLV3-10 | 0.0591186588940951 | 0.0591186588940951 | -10.70207219 | 11.4883642597917 | 0.0613302300833296 | 0.985643503572262 |
| IGLV3-12 | -35.79119955 | -34.79119955 | -16.92985512 | 5.03786226303785 | -1.218192143 | 0.366357598549604 |
| IGLV3-16 | -1.546201262 | -0.546201262 | -18.75258264 | 5.26451363024563 | -0.971840192 | 0.399778344600192 |
| IGLV3-19 | -0.050768259 | 0.949231741 | -1.697644077 | 0.587068134611196 | -1.064630772 | 0.40728216732969 |
| IGLV3-21 | -0.469796001 | 0.530203999 | -15.12857375 | 7.05544890714111 | -0.812595376 | 0.532840186984085 |
| IGLV3-25 | 35.0085367224031 | 35.0085367224031 | -7.543332049 | 14.4560629885782 | 0.707106781186548 | 0.539497285386707 |
| IGLV3-9 | 0.0886343605137891 | 0.0886343605137891 | -0.94180802 | 2.69032264553935 | 0.940935790444159 | 0.445522961338631 |
| IGLV4-60 | 0.332424657044334 | 0.332424657044334 | -7.657304051 | 12.6316195437305 | 0.544464830570026 | 0.623585169643218 |
| IGLV4-69 | -0.050780286 | 0.949219714 | -2.44085571 | 1.41660030443085 | -0.526336255 | 0.625689848851569 |
| IGLV5-39 | 35.0618715980005 | 35.0618715980005 | -7.827419731 | 15.0004894292904 | 0.707106781186547 | 0.539497285386707 |
| IGLV5-45 | -0.307043415 | 0.692956585 | -13.79011335 | 8.67119558634159 | -0.493764914 | 0.648403356778023 |
| IGLV5-52 | 34.7744528318396 | 34.7744528318396 | -6.413527374 | 12.2909020969923 | 0.707106781186548 | 0.539497285386707 |
| IGLV6-57 | 0.0590549662923238 | 0.0590549662923238 | -0.756137482 | 1.85043198474904 | 0.797893634103788 | 0.520251905198183 |
| IGLV7-43 | 0.521883639337774 | 0.521883639337774 | -5.390740736 | 12.9650487866682 | 0.917403301575632 | 0.483749664205478 |
| IGLV7-46 | 0.0146107868933497 | 0.0146107868933497 | -12.69919787 | 12.8282974896923 | 0.00875068854609636 | 0.999130434024431 |
| IGLV8-61 | 0.571758588132306 | 0.571758588132306 | -6.053642447 | 16.5066808296045 | 1.01248669762144 | 0.431481015871607 |
| IGLV9-49 | -0.339116387 | 0.660883613 | -15.0033572 | 9.00247522175245 | -0.560149371 | 0.616550083670876 |
| IGSF10 | 35.800657542588 | 35.800657542588 | -5.022597959 | 16.99280826 | 1.22354843042442 | 0.366275438800159 |
| IGSF3 | -34.8656551 | -33.8656551 | -13.09297614 | 6.83205839834191 | -0.707106781 | 0.539497285386707 |
| IGSF8 | 0.00447964925130656 | 0.00447964925130656 | -0.293019438 | 0.374513838974144 | 0.222648907851948 | 0.845851627629491 |
| IK | -0.017742912 | 0.982257088 | -0.416657328 | 0.0809333875184784 | -1.20747945 | 0.344111735183475 |
| IKBIP | 0.0646001022322847 | 0.0646001022322847 | 0.164165275456625 | 1.17426259950341 | 2.83191869224753 | 0.114657219638757 |
| IKBKB | -0.571043729 | 0.428956271 | -15.17344433 | 8.92269196637298 | -0.451360595 | 0.655781420491697 |
| IKBKG | -36.53469265 | -35.53469265 | -20.6113436 | 0.70147626897247 | -2.102197614 | 0.180616181545238 |
| IKZF1 | -36.91651955 | -35.91651955 | -13.17413491 | -12.76823907 | -143.8272578 | 0.000135517346157358 |
| IKZF3 | -36.87618276 | -35.87618276 | -12.96039008 | -12.26669843 | -81.83644486 | 0.000220658446544722 |
| IL13RA2 | 0 | 1 | NA | NA | NA | NA |
| IL16 | -0.578365097 | 0.421634903 | -14.83573514 | 5.45307628746638 | -1.03885068 | 0.424772789828451 |
| IL17D | 0.695940089604214 | 0.695940089604214 | -9.007538443 | 17.4777976534857 | 0.553951593657403 | 0.596908615806043 |
| IL18 | -37.00144918 | -36.00144918 | -14.75570487 | -12.75971112 | -31.02151419 | 0.00135930837203558 |
| IL1RN | -0.044496814 | 0.955503186 | -11.43503477 | 11.2330817535814 | -0.015418365 | 0.999130434024431 |
| IL36A | 0 | 1 | NA | NA | NA | NA |
| IL36G | 0 | 1 | NA | NA | NA | NA |
| IL36RN | 0 | 1 | NA | NA | NA | NA |
| IL4I1 | -0.082239923 | 0.917760077 | -2.039418872 | 0.561475338666139 | -1.124451462 | 0.368125003866442 |
| ILF2 | -0.026534697 | 0.973465303 | -0.573658163 | -0.020220273 | -1.949286423 | 0.16038335819584 |
| ILF3 | -0.025169487 | 0.974830513 | -0.500797184 | -0.056276974 | -2.278226304 | 0.114919247158557 |
| ILK | 0.0498396260607511 | 0.0498396260607511 | 0.0953642463739794 | 0.934951020548169 | 2.48999409796728 | 0.127493113256436 |
| ILKAP | -36.58993539 | -35.58993539 | -21.31647836 | 0.629451118935408 | -2.121241886 | 0.1803694486231 |
| ILVBL | 0.0352808608206992 | 0.0352808608206992 | -0.109417076 | 0.768442051846472 | 1.38087194227064 | 0.283708019792457 |
| IMMT | -0.013529652 | 0.986470348 | -0.359937804 | 0.0709950948578055 | -1.169430935 | 0.351599596135942 |
| IMP3 | -0.441413204 | 0.558586796 | -13.6216568 | 6.74999394902517 | -0.758703986 | 0.539497285386707 |
| IMP4 | -1.990431664 | -0.990431664 | -19.30985564 | 0.627728418324448 | -2.097811714 | 0.18042925372161 |
| IMPA1 | -0.016395879 | 0.983604121 | -0.530843626 | 0.198209715962437 | -0.789665769 | 0.509607148662422 |
| IMPA2 | 0 | 1 | NA | NA | NA | NA |
| IMPACT | 0.0085860355854745 | 0.0085860355854745 | -0.138290245 | 0.293562339888567 | 0.628415871898449 | 0.554342150065539 |
| IMPDH1 | -0.457900092 | 0.542099908 | -14.09478656 | 6.75617223405318 | -0.791242059 | 0.539497285386707 |
| IMPDH2 | -0.006510423 | 0.993489577 | -0.343173083 | 0.206148837953527 | -0.440059116 | 0.66180921540292 |
| IMUP | 0 | 1 | NA | NA | NA | NA |
| INF2 | 0.029201097610704 | 0.029201097610704 | -0.078044005 | 0.621018280761884 | 1.48066365692511 | 0.262299994913958 |
| ING1 | 0.0298859724352401 | 0.0298859724352401 | -12.02804487 | 12.2799835825609 | 0.0179346831520274 | 0.999130434024431 |
| INIP | 0.0707860762257519 | 0.0707860762257519 | -10.61801406 | 11.1510295614726 | 0.0423926240983815 | 0.994613821823136 |
| INPP1 | -0.004986056 | 0.995013944 | -0.808178342 | 0.721666818270404 | -0.110358099 | 0.946943550418751 |
| INPP4A | -0.015505528 | 0.984494472 | -0.659006834 | 0.380233667149672 | -0.47600174 | 0.646398999780997 |
| INPP5D | -1.045824581 | -0.045824581 | -18.65575998 | 4.96069440609315 | -1.302694164 | 0.363812765896965 |
| INPP5K | 0.0695360794120911 | 0.0695360794120911 | -0.163643614 | 1.37146956377613 | 1.67379042870032 | 0.24307867089863 |
| INPPL1 | 0.0118622906371876 | 0.0118622906371876 | -0.22626201 | 0.440110791840677 | 0.585180273640255 | 0.581827289940927 |
| INSR | -35.73608558 | -34.73608558 | -16.24303171 | 4.79676970922141 | -1.22424442 | 0.366275438800159 |
| INTS1 | -0.035393309 | 0.964606691 | -0.618658392 | -0.010412699 | -1.802748863 | 0.170592731777117 |
| INTS10 | -0.032092327 | 0.967907673 | -0.583919061 | 0.0021697116856385 | -1.803042146 | 0.1803694486231 |
| INTS11 | -0.044974532 | 0.955025468 | -0.795583731 | -0.028770239 | -1.898553339 | 0.159120006280923 |
| INTS13 | -0.003123398 | 0.996876602 | -0.359274921 | 0.303421034690212 | -0.152131236 | 0.910745108584665 |
| INTS14 | -34.70216836 | -33.70216836 | -11.69025446 | 6.1001028568765 | -0.707106781 | 0.539497285386707 |
| INTS2 | -1.589229393 | -0.589229393 | -15.32276763 | 4.08299395427526 | -1.002149219 | 0.38541466403847 |
| INTS3 | -0.043501947 | 0.956498053 | -1.610318758 | 0.870127061417423 | -0.655236035 | 0.561015630932865 |
| INTS4 | -2.108156265 | -1.108156265 | -18.01573982 | -0.37384214 | -2.338172008 | 0.171399939255817 |
| INTS5 | -0.053777514 | 0.946222486 | -0.862342268 | -0.020609762 | -1.925814659 | 0.167509566752535 |
| INTS6 | -34.91931214 | -33.91931214 | -13.58910192 | 7.09094226685596 | -0.707106781 | 0.539497285386707 |
| INTS7 | -1.991827469 | -0.991827469 | -18.92431823 | 0.611259509701486 | -2.102019791 | 0.18042925372161 |
| INTS9 | -0.988194556 | 0.011805444 | -14.42993949 | 8.41585873642194 | -0.457491557 | 0.653774096383628 |
| IPO11 | -0.989464868 | 0.010535132 | -16.46639959 | 4.93076098681661 | -1.201266451 | 0.36731973075278 |
| IPO13 | 0.555326494232807 | 0.555326494232807 | -8.623776588 | 14.4264857222861 | 0.438369214357027 | 0.662035268543307 |
| IPO4 | -0.016690857 | 0.983309143 | -0.715453942 | 0.42150787986868 | -0.452783776 | 0.655753631330814 |
| IPO5 | -0.025050836 | 0.974949164 | -0.695995587 | 0.197457245253952 | -1.203263619 | 0.366275438800159 |
| IPO7 | 0.00575744585814037 | 0.00575744585814037 | -0.251865591 | 0.367799534461386 | 0.378242211102094 | 0.717299881785849 |
| IPO8 | 0.395750685035806 | 0.395750685035806 | -6.737864799 | 12.4377288100977 | 0.668316552668451 | 0.557497737387282 |
| IPO9 | 0.0236535806541105 | 0.0236535806541105 | -0.021265321 | 0.462959891411169 | 1.70455772580345 | 0.1915940163361 |
| IQCD | 36.4737251625047 | 36.4737251625047 | -0.594842734 | 19.6808604746136 | 2.11829033538452 | 0.1803694486231 |
| IQGAP1 | 0.00438074351332812 | 0.00438074351332812 | -0.066093341 | 0.164227675710082 | 0.750446660916695 | 0.532491104424462 |
| IQGAP2 | -0.020519758 | 0.979480242 | -0.555219182 | 0.15071303487414 | -1.047265961 | 0.379603314376294 |
| IQSEC1 | 0.473240258194271 | 0.473240258194271 | -6.512847635 | 14.0455206284814 | 0.822465261493503 | 0.530227332284313 |
| IRAG1 | 0.0810774438612218 | 0.0810774438612218 | 0.141066485165712 | 1.2749327581901 | 2.57389219175695 | 0.124684545530739 |
| IRAG2 | -2.153863332 | -1.153863332 | -20.51195991 | -0.830295207 | -2.439208743 | 0.163145168170835 |
| IRAK4 | 0.389487540659982 | 0.389487540659982 | -7.605331381 | 13.876579904775 | 0.656697993027706 | 0.562031639448264 |
| IREB2 | 0.62358511681093 | 0.62358511681093 | -7.946980946 | 14.2749553258758 | 0.494336544174293 | 0.634031727574291 |
| IRF2BP1 | 0.0156559479475028 | 0.0156559479475028 | -0.151143434 | 0.428590716633458 | 0.901215148666583 | 0.461121200688429 |
| IRF2BP2 | 0.00452956385893418 | 0.00452956385893418 | -0.134976913 | 0.225850270741424 | 0.469073337045421 | 0.652187811666889 |
| IRF2BPL | -0.43935557 | 0.56064443 | -12.89945827 | 6.41254385387656 | -0.754236838 | 0.539497285386707 |
| IRF3 | -0.039552979 | 0.960447021 | -0.854977607 | 0.0963278378904598 | -1.44095285 | 0.255413410793726 |
| IRF4 | -2.108062761 | -1.108062761 | -21.62923748 | -0.432981921 | -2.340561894 | 0.171755377781913 |
| IRF5 | -35.73193191 | -34.73193191 | -16.22270572 | 4.80935130991439 | -1.221174254 | 0.366275438800159 |
| IRF6 | -34.81741931 | -33.81741931 | -12.6624563 | 6.6074084280171 | -0.707106781 | 0.539497285386707 |
| IRF7 | 0 | 1 | NA | NA | NA | NA |
| IRF8 | -34.77689335 | -33.77689335 | -12.31171146 | 6.42438592765568 | -0.707106781 | 0.539497285386707 |
| IRF9 | 0.0474480547435739 | 0.0474480547435739 | 0.142751701382547 | 0.761078388511329 | 2.97153495790825 | 0.0926613647437377 |
| IRGQ | 0.0389026566792201 | 0.0389026566792201 | -0.055083329 | 0.792216019792494 | 1.5297132698914 | 0.215395426601888 |
| IRS1 | 35.7827700623543 | 35.7827700623543 | -4.95049564 | 16.7732076949005 | 1.22469892360448 | 0.366275438800159 |
| ISCA2 | -0.38550098 | 0.61449902 | -13.61596562 | 7.51292072399532 | -0.649589148 | 0.56568869640334 |
| ISCU | 0.544969217734112 | 0.544969217734112 | -8.358135735 | 13.8314037157913 | 0.429745554965482 | 0.667191447813252 |
| ISG15 | 0.0514865765172163 | 0.0514865765172163 | -0.366793705 | 1.36412303433431 | 1.19195434697355 | 0.366275438800159 |
| ISG20 | -0.627145495 | 0.372854505 | -14.48264748 | 4.64261840176351 | -1.151363724 | 0.380647142332623 |
| ISLR | 0.151430496446454 | 0.151430496446454 | 1.03691898455561 | 2.06499163908196 | 5.55017453963051 | 0.0158873075776561 |
| ISOC1 | -0.019554494 | 0.980445506 | -0.470649582 | 0.0902538443233333 | -1.183831482 | 0.346015552988529 |
| ISOC2 | -0.007985349 | 0.992014651 | -0.703480876 | 0.55183302246907 | -0.213170341 | 0.853973629015084 |
| IST1 | -0.0196457 | 0.9803543 | -0.461500104 | 0.0610942720565025 | -1.518929931 | 0.263546001045689 |
| ISY1 | -0.011014774 | 0.988985226 | -0.476691811 | 0.284283741763882 | -0.439860613 | 0.661026237313678 |
| ISYNA1 | 0.0557634290927415 | 0.0557634290927415 | 0.104168545852645 | 0.969257452328487 | 2.44589999334953 | 0.123637539973339 |
| ITCH | -0.432239754 | 0.567760246 | -14.10623768 | 7.11794199118335 | -0.740835591 | 0.539497285386707 |
| ITGA1 | 0.0967118061195755 | 0.0967118061195755 | 0.443367515691719 | 1.43742801169441 | 3.44959811680725 | 0.0488911633452712 |
| ITGA10 | 34.8396685783743 | 34.8396685783743 | -6.710097778 | 12.8592504614322 | 0.707106781186547 | 0.539497285386707 |
| ITGA11 | 0.126150861388269 | 0.126150861388269 | 0.685854649701482 | 1.82407363765848 | 4.62196726415351 | 0.0489656305565222 |
| ITGA2 | 1.06479010890148 | 1.06479010890148 | -4.682678595 | 18.4133547173273 | 1.33659553942236 | 0.356478906085352 |
| ITGA2B | -0.004283866 | 0.995716134 | -1.047717375 | 0.957065424108713 | -0.080726022 | 0.970803346798997 |
| ITGA3 | -0.921195294 | 0.078804706 | -18.96715571 | 6.54507469854367 | -1.093524778 | 0.400909502654508 |
| ITGA4 | -0.030977228 | 0.969022772 | -0.935347558 | 0.35115089126029 | -1.009726304 | 0.435944213720461 |
| ITGA5 | 0.0685032421870867 | 0.0685032421870867 | 0.227914877259832 | 1.15248950454376 | 2.70091153762603 | 0.0796176030128218 |
| ITGA6 | -0.015970001 | 0.984029999 | -0.575186683 | 0.248274477981628 | -0.691773989 | 0.539497285386707 |
| ITGA7 | -0.36708365 | 0.63291635 | -13.17582959 | 7.5162384599286 | -0.613447053 | 0.584826420924461 |
| ITGA9 | 1.21917442403962 | 1.21917442403962 | -3.007308899 | 18.7635525135695 | 1.62365183960585 | 0.272904858412467 |
| ITGAL | -1.019343169 | -0.019343169 | -19.37127157 | 5.47508168399109 | -1.257114577 | 0.366275438800159 |
| ITGAM | -0.114964092 | 0.885035908 | -1.776165385 | -0.528131284 | -3.231575901 | 0.0461919266370588 |
| ITGAV | 0.101541918312173 | 0.101541918312173 | 0.744424568599706 | 1.41984940894115 | 5.56170498239743 | 0.0079071835597774 |
| ITGAX | -1.077376035 | -0.077376035 | -19.21639326 | 4.71692917451628 | -1.356228356 | 0.350680114094073 |
| ITGB1 | 0.0490224069119635 | 0.0490224069119635 | 0.145886258574366 | 0.85681165968853 | 2.51760315407355 | 0.0872617597809388 |
| ITGB2 | -0.115651038 | 0.884348962 | -1.910916405 | -0.443860981 | -2.782651589 | 0.0623026754878798 |
| ITGB3 | 0.0178253834088372 | 0.0178253834088372 | -0.796302358 | 1.14055935600257 | 0.338020657285063 | 0.747275118469679 |
| ITGB4 | 0.047838615500811 | 0.047838615500811 | -0.107275105 | 1.04304640814501 | 1.41201152243962 | 0.24976923379291 |
| ITGB5 | 0.101916724331861 | 0.101916724331861 | 0.533419388174885 | 1.55073901268802 | 4.07630051461478 | 0.0501864839417429 |
| ITGB6 | 0.0284738166822671 | 0.0284738166822671 | -1.84355897 | 2.34621123608357 | 0.261936020846216 | 0.818049856834345 |
| ITGBL1 | 0.546939047926307 | 0.546939047926307 | -5.704032869 | 14.4747736535263 | 0.977569849351032 | 0.455327499849187 |
| ITIH1 | 0.10399482406931 | 0.10399482406931 | 0.5171331120784 | 1.91378976809405 | 3.06017138034998 | 0.0536782776480021 |
| ITIH2 | 0.086483561963279 | 0.086483561963279 | 0.510978720797858 | 1.49949142411675 | 3.51960344505121 | 0.0353667134923509 |
| ITIH3 | 0.149626078185739 | 0.149626078185739 | 0.210846951152138 | 2.60543757991708 | 2.45000104817752 | 0.139101976398134 |
| ITIH4 | 0.0546549510792333 | 0.0546549510792333 | -0.090697934 | 1.2854660725427 | 1.63388735441907 | 0.211294118489054 |
| ITIH5 | 36.4650899660456 | 36.4650899660456 | -0.578867492 | 19.5509878014383 | 2.12090543098789 | 0.1803694486231 |
| ITM2B | -0.044622686 | 0.955377314 | -10.29533134 | 10.1129971642914 | -0.015461963 | 0.999130434024431 |
| ITM2C | -2.129365019 | -1.129365019 | -19.66850018 | -0.624626712 | -2.377541686 | 0.167106013143295 |
| ITPA | 0.439167651387794 | 0.439167651387794 | -6.69605959 | 13.4291068445041 | 0.752209754337402 | 0.539497285386707 |
| ITPK1 | -35.90539001 | -34.90539001 | -18.27881261 | 5.40730566721948 | -1.222873655 | 0.366275438800159 |
| ITPR1 | -0.365017476 | 0.634982524 | -13.04610836 | 7.48076405071076 | -0.609780263 | 0.587270323007015 |
| ITPR2 | -0.467286688 | 0.532713312 | -12.55579726 | 5.900071658 | -0.811271388 | 0.534281958550569 |
| ITPR3 | -0.407812001 | 0.592187999 | -13.8404857 | 7.33257909080777 | -0.691046308 | 0.547266939638037 |
| ITPRID2 | -0.984413388 | 0.015586612 | -15.37798447 | 8.98822986911353 | -0.455671995 | 0.654211894695707 |
| ITPRIP | -0.997820729 | 0.002179271 | -17.33093568 | 5.13566439055595 | -1.219986964 | 0.366275438800159 |
| ITSN1 | 36.9987768366021 | 36.9987768366021 | 13.5260674489948 | 13.9384280860695 | 149.878904997447 | 0.000135517346157358 |
| ITSN2 | -36.38471745 | -35.38471745 | -18.49054887 | 0.546463293448231 | -2.121136569 | 0.1803694486231 |
| IVD | -0.000684881 | 0.999315119 | -0.488137671 | 0.474158250213145 | -0.028249553 | 0.998435102357591 |
| IVL | 35.1511937010577 | 35.1511937010577 | -8.327358369 | 15.9585732567744 | 0.707106781186548 | 0.539497285386707 |
| IVNS1ABP | -35.8041269 | -34.8041269 | -17.07402901 | 5.07499838825806 | -1.219095107 | 0.366275438800159 |
| IWS1 | 0.00649940560897645 | 0.00649940560897645 | -13.34248097 | 13.402787670706 | 0.00390143230509561 | 0.999130434024431 |
| JAG1 | 0 | 1 | NA | NA | NA | NA |
| JAGN1 | -1.018934171 | -0.018934171 | -17.2305605 | 4.8735714932921 | -1.256377488 | 0.366275438800159 |
| JAK1 | -0.983903891 | 0.016096109 | -18.18065954 | 5.55141832927298 | -1.197193357 | 0.368508989355442 |
| JAM2 | 0.125096645845713 | 0.125096645845713 | 0.335052310240685 | 1.702853462 | 2.57839357211953 | 0.0751894512272061 |
| JAM3 | -0.003634499 | 0.996365501 | -10.65376774 | 10.6073473119226 | -0.003777695 | 0.999130434024431 |
| JCAD | 1.66118468981075 | 1.66118468981075 | -4.524674582 | 18.5267600517039 | 1.05173431932056 | 0.367529743405878 |
| JCHAIN | 0.0415447715052818 | 0.0415447715052818 | -0.216556788 | 1.18243904040833 | 1.30933605219858 | 0.321437437138493 |
| JMJD6 | -36.36838994 | -35.36838994 | -18.2924785 | 0.55032784172416 | -2.118881968 | 0.1803694486231 |
| JMJD7 | 0 | 1 | NA | NA | NA | NA |
| JOSD2 | 0.0577630489152746 | 0.0577630489152746 | -10.79190817 | 11.0443767843033 | 0.02001246465686 | 0.999130434024431 |
| JPH1 | 0 | 1 | NA | NA | NA | NA |
| JPH2 | 0 | 1 | NA | NA | NA | NA |
| JPT1 | -0.016209631 | 0.983790369 | -0.642909102 | 0.338173452265112 | -0.637755181 | 0.563130836110916 |
| JPT2 | 0.0333217916154944 | 0.0333217916154944 | -0.430809883 | 1.03551870948518 | 0.741912324097637 | 0.537923342132161 |
| JSRP1 | -1.880587923 | -0.880587923 | -18.59679831 | 3.47299559090648 | -1.197575836 | 0.341345038376949 |
| JTB | 0.0176667850029782 | 0.0176667850029782 | -0.368411157 | 0.690342982568735 | 0.537965593718432 | 0.61079104937064 |
| JUN | 1.90488781959946 | 1.90488781959946 | -2.79838844 | 15.4421034943651 | 1.2133379226353 | 0.334942767234843 |
| JUNB | 0.91496434248434 | 0.91496434248434 | -9.003340653 | 14.8003799648045 | 0.42221218606486 | 0.67220752984697 |
| JUP | 0.045108099442942 | 0.045108099442942 | -0.900229413 | 1.71900885850529 | 0.648388528061801 | 0.560618525135692 |
| KANK1 | 0.55250927995503 | 0.55250927995503 | -5.449724985 | 13.9518226040739 | 0.985152302107907 | 0.451119084316298 |
| KANK2 | 0.120482980000449 | 0.120482980000449 | 0.505742680422208 | 1.88871655276348 | 3.43779528911709 | 0.067599734050344 |
| KANK3 | -1.459218407 | -0.459218407 | -17.33911781 | 5.38600397395774 | -0.911718508 | 0.43406228052404 |
| KANK4 | 34.9589620050082 | 34.9589620050082 | -7.288526688 | 13.9677532693987 | 0.707106781186547 | 0.539497285386707 |
| KARS1 | -0.036268752 | 0.963731248 | -0.96105001 | 0.209621298040991 | -1.357588178 | 0.336395164305236 |
| KAT7 | -1.039084092 | -0.039084092 | -16.66685385 | 4.50812510848973 | -1.289206623 | 0.366275438800159 |
| KAZALD1 | 0 | 1 | NA | NA | NA | NA |
| KBTBD11 | 0.158714339273733 | 0.158714339273733 | 0.669080336601569 | 2.23785253742806 | 3.42843574874258 | 0.0525595373037303 |
| KCMF1 | 0.391290788357603 | 0.391290788357603 | -6.775621227 | 12.5397596558478 | 0.651382623239139 | 0.562031639448264 |
| KCNA3 | -36.83161841 | -35.83161841 | -13.87448927 | -10.58525571 | -16.73413623 | 0.00629441630560795 |
| KCNAB2 | -0.442607955 | 0.557392045 | -14.50582171 | 7.16781698885173 | -0.761386077 | 0.539497285386707 |
| KCNN4 | -0.065981174 | 0.934018826 | -9.358916435 | 8.65102325943334 | -0.068023213 | 0.979715024510606 |
| KCTD10 | 0.0490948822929947 | 0.0490948822929947 | -0.127788772 | 0.98218221433384 | 1.58533622164942 | 0.256501558948442 |
| KCTD12 | 0.0491333679011874 | 0.0491333679011874 | 0.167961214163099 | 0.90869875690347 | 2.54625074348938 | 0.0810869445560843 |
| KCTD15 | 2.08747919578686 | 2.08747919578686 | 0.20803757619738 | 19.7875804489357 | 2.29811614828951 | 0.17552300612974 |
| KDELR3 | 0.372876558298767 | 0.372876558298767 | -7.359852203 | 13.0452054474199 | 0.623991276537865 | 0.57827242635708 |
| KDM1A | 0.0132773336055413 | 0.0132773336055413 | -0.571812353 | 0.812666002663092 | 0.373459039120859 | 0.724657663497786 |
| KDM2A | -0.028127342 | 0.971872658 | -11.14506259 | 10.7747095820903 | -0.029236271 | 0.998435102357591 |
| KDM3B | -2.084899003 | -1.084899003 | -19.0644762 | -0.190113856 | -2.290451487 | 0.175707003568027 |
| KDSR | 0.0570935269874292 | 0.0570935269874292 | 0.210857115024747 | 0.858367241016898 | 3.02640809311396 | 0.0648842992292444 |
| KEAP1 | -0.407917029 | 0.592082971 | -13.36034207 | 7.06731656100313 | -0.692220342 | 0.546638926108471 |
| KEL | 0.0792969945710491 | 0.0792969945710491 | -11.6322978 | 12.0072688278273 | 0.0274649627181082 | 0.998435102357591 |
| KERA | 34.8833987052769 | 34.8833987052769 | -6.916604271 | 13.2550000934059 | 0.707106781186548 | 0.539497285386707 |
| KHDRBS1 | 0.0094551432023295 | 0.0094551432023295 | -0.502877565 | 0.694984451922654 | 0.2790585537779 | 0.793345075734739 |
| KHDRBS3 | -0.037810271 | 0.962189729 | -10.53164355 | 10.0641610913803 | -0.039278536 | 0.995578370294886 |
| KHSRP | -0.024425994 | 0.975574006 | -0.465706295 | -0.064253158 | -2.366425347 | 0.102621388225085 |
| KIAA0930 | -2.048793796 | -1.048793796 | -18.68799282 | 0.128815342649919 | -2.217902427 | 0.1803694486231 |
| KIAA1191 | 0.569787193018287 | 0.569787193018287 | -7.71627154 | 13.102070672979 | 0.450142642074396 | 0.656403662616607 |
| KIAA1217 | 0.0717869205859223 | 0.0717869205859223 | 0.279420397351951 | 1.15049505471597 | 3.38515326438124 | 0.0781558085553345 |
| KIAA1522 | -34.82841383 | -33.82841383 | -12.75932316 | 6.65795461841764 | -0.707106781 | 0.539497285386707 |
| KIAA1614 | 0.130691614639502 | 0.130691614639502 | 0.566461218527351 | 2.2213184778874 | 2.93929545511216 | 0.0562202894362036 |
| KIAA2013 | -0.475380718 | 0.524619282 | -13.08217819 | 6.04074428984498 | -0.826569583 | 0.528014169078056 |
| KIDINS220 | 34.6631741695732 | 34.6631741695732 | -5.937433213 | 11.3785138926153 | 0.707106781186547 | 0.539497285386707 |
| KIF13A | 0.972769606217254 | 0.972769606217254 | -5.585406896 | 18.0029999119163 | 1.17359968091569 | 0.374103691020701 |
| KIF13B | -0.055772381 | 0.944227619 | -0.969714849 | -0.084205139 | -2.586845084 | 0.138062043939893 |
| KIF1B | -1.606768315 | -0.606768315 | -16.08921795 | 4.19222483389684 | -1.015000518 | 0.380776553328625 |
| KIF21A | -1.952915141 | -0.952915141 | -19.4389754 | 0.994075009491229 | -2.029808946 | 0.188528137102147 |
| KIF2A | -0.001977702 | 0.998022298 | -0.191176713 | 0.153110169873827 | -0.216434691 | 0.854416103164352 |
| KIF5B | 0.0262828846528852 | 0.0262828846528852 | 0.0114051194034407 | 0.511087161096595 | 1.83151353970283 | 0.167580633021715 |
| KIFAP3 | -35.56903161 | -34.56903161 | -14.46541127 | 4.27068836863333 | -1.224453581 | 0.366275438800159 |
| KIFBP | 0.59934118711862 | 0.59934118711862 | -8.550924453 | 14.9780656434968 | 0.47464727722477 | 0.645841531946401 |
| KIRREL1 | 0.116267270699894 | 0.116267270699894 | 0.338667845857635 | 1.60756486494877 | 2.8094890740141 | 0.0765517955754613 |
| KLC1 | 0.0178440702641209 | 0.0178440702641209 | 0.000225002979102585 | 0.328386862091311 | 1.75284893212455 | 0.179117559639718 |
| KLC2 | -35.90011556 | -34.90011556 | -18.20352869 | 5.37899370676632 | -1.223763394 | 0.366275438800159 |
| KLC3 | 0 | 1 | NA | NA | NA | NA |
| KLC4 | 0.0408994366389144 | 0.0408994366389144 | -0.001430132 | 0.69773061544519 | 2.14462207802743 | 0.179833016962564 |
| KLHL31 | 0 | 1 | NA | NA | NA | NA |
| KLHL40 | 34.9091114596686 | 34.9091114596686 | -7.040982078 | 13.493358075924 | 0.707106781186547 | 0.539497285386707 |
| KLHL41 | 35.872749134279 | 35.872749134279 | -5.320915082 | 17.904474727975 | 1.21923265858662 | 0.366275438800159 |
| KLK10 | -34.99872758 | -33.99872758 | -14.35810697 | 7.49221752464166 | -0.707106781 | 0.539497285386707 |
| KLK11 | 0 | 1 | NA | NA | NA | NA |
| KLK12 | 0 | 1 | NA | NA | NA | NA |
| KLK13 | 0 | 1 | NA | NA | NA | NA |
| KLK14 | 0 | 1 | NA | NA | NA | NA |
| KLK4 | 34.8061593639633 | 34.8061593639633 | -6.556039627 | 12.5640129842708 | 0.707106781186548 | 0.539497285386707 |
| KLK5 | 0 | 1 | NA | NA | NA | NA |
| KLK6 | 0 | 1 | NA | NA | NA | NA |
| KLK7 | 0 | 1 | NA | NA | NA | NA |
| KLKB1 | 0.0729284667103241 | 0.0729284667103241 | 0.372441192148954 | 1.0760721891179 | 3.79817903401087 | 0.0411222892664865 |
| KMT2A | -1.72951268 | -0.72951268 | -16.35795016 | 3.67094933384939 | -1.098648813 | 0.366275438800159 |
| KNG1 | 0.0459485844909809 | 0.0459485844909809 | -0.034744635 | 1.08320002473271 | 1.67277303206492 | 0.183650685850336 |
| KPNA1 | -0.012298392 | 0.987701608 | -0.469025209 | 0.254148293995307 | -0.516575192 | 0.621617284366183 |
| KPNA2 | -36.78699226 | -35.78699226 | -12.27852825 | -11.43619804 | -63.35513568 | 0.000273271710161686 |
| KPNA3 | -0.007638018 | 0.992361982 | -0.371357564 | 0.2321640719418 | -0.458078677 | 0.658252010682239 |
| KPNA4 | -0.423356083 | 0.576643917 | -14.13084491 | 7.25256767441254 | -0.722106092 | 0.539497285386707 |
| KPNA6 | -0.031283849 | 0.968716151 | -0.612928846 | 0.0158653748558266 | -1.688775271 | 0.180616181545238 |
| KPNB1 | -0.01203912 | 0.98796088 | -0.280965321 | 0.0177827671661545 | -1.558768691 | 0.209084267485673 |
| KRAS | -0.011628297 | 0.988371703 | -10.76570086 | 10.6164021948417 | -0.012081365 | 0.999130434024431 |
| KREMEN2 | 0.203639500417302 | 0.203639500417302 | 0.936578683607192 | 3.14689164801197 | 3.29536965484085 | 0.0488911633452712 |
| KRI1 | -34.7595157 | -33.7595157 | -12.16430315 | 6.34746665905902 | -0.707106781 | 0.539497285386707 |
| KRR1 | -2.071559412 | -1.071559412 | -18.6400876 | -0.099532676 | -2.257625412 | 0.177365510008242 |
| KRT1 | 0.0297086523888957 | 0.0297086523888957 | -0.984692576 | 1.66666282320647 | 0.447582644049926 | 0.657423743030449 |
| KRT10 | 0.038587602510057 | 0.038587602510057 | -0.958516934 | 1.80616577906792 | 0.53235817007254 | 0.612233031829465 |
| KRT13 | -0.011565016 | 0.988434984 | -3.893499405 | 3.64962310323718 | -0.07181417 | 0.979078017074215 |
| KRT14 | 0.107365281507116 | 0.107365281507116 | -1.118565004 | 3.52086787041303 | 1.13749708454732 | 0.38162584839503 |
| KRT15 | 0.860904551595002 | 0.860904551595002 | -7.654197566 | 19.8497910068989 | 0.993636057763787 | 0.446249950313389 |
| KRT16 | 0.0397455145267217 | 0.0397455145267217 | -1.386901238 | 2.19849800473083 | 0.440451218638001 | 0.667233726295542 |
| KRT17 | 0.035711773677485 | 0.035711773677485 | -0.667975658 | 1.42311493616917 | 0.637198515182074 | 0.551036879047474 |
| KRT18 | 0.408049463040853 | 0.408049463040853 | -7.758798634 | 14.643922747646 | 0.690968961044345 | 0.547266939638037 |
| KRT19 | 0.109079286861958 | 0.109079286861958 | -2.220751908 | 4.62986723254031 | 0.778841107533589 | 0.539497285386707 |
| KRT2 | 0.0146368511867776 | 0.0146368511867776 | -1.202390667 | 1.51034805993966 | 0.20028797524073 | 0.865629352694556 |
| KRT23 | 0 | 1 | NA | NA | NA | NA |
| KRT24 | 0 | 1 | NA | NA | NA | NA |
| KRT25 | 0 | 1 | NA | NA | NA | NA |
| KRT3 | 35.8622113840695 | 35.8622113840695 | -5.417325396 | 17.9093069161654 | 1.20510635418037 | 0.36731973075278 |
| KRT31 | 0 | 1 | NA | NA | NA | NA |
| KRT33A | 0 | 1 | NA | NA | NA | NA |
| KRT33B | 0 | 1 | NA | NA | NA | NA |
| KRT36 | -34.946482 | -33.946482 | -13.84744656 | 7.22574933030052 | -0.707106781 | 0.539497285386707 |
| KRT4 | 0.0139393965198143 | 0.0139393965198143 | -11.77996301 | 11.9784656900156 | 0.0144562801137809 | 0.999130434024431 |
| KRT5 | 0.0755351481491783 | 0.0755351481491783 | -2.386100285 | 4.10238194951945 | 0.586105385767664 | 0.59981565799656 |
| KRT6A | 0.0235564220337811 | 0.0235564220337811 | -2.273043133 | 2.7768645476231 | 0.214112106169427 | 0.859623126258847 |
| KRT6B | 0.0959522220313376 | 0.0959522220313376 | -13.40213437 | 14.3191926964811 | 0.0573048992742367 | 0.987378189240374 |
| KRT6C | 34.8606810274817 | 34.8606810274817 | -6.80854365 | 13.0479124111054 | 0.707106781186548 | 0.539497285386707 |
| KRT7 | 1.09269712876638 | 1.09269712876638 | -9.276561716 | 16.7938635510881 | 0.504162199122678 | 0.62887762002885 |
| KRT71 | 0 | 1 | NA | NA | NA | NA |
| KRT72 | 0 | 1 | NA | NA | NA | NA |
| KRT75 | 0.13029317896815 | 0.13029317896815 | -11.4667359 | 12.0789940970662 | 0.0450796327417987 | 0.99301710306099 |
| KRT76 | -35.7718297 | -34.7718297 | -16.70865138 | 4.97525519416197 | -1.217677538 | 0.36636668125889 |
| KRT77 | 36.517807933127 | 36.517807933127 | -0.61716579 | 20.2953743628102 | 2.11750701944194 | 0.1803694486231 |
| KRT78 | 34.8888436972168 | 34.8888436972168 | -6.94275811 | 13.3051213843253 | 0.707106781186547 | 0.539497285386707 |
| KRT79 | 0 | 1 | NA | NA | NA | NA |
| KRT8 | 0.454855120912453 | 0.454855120912453 | -7.449570772 | 15.4222554244362 | 0.781346870769359 | 0.539497285386707 |
| KRT80 | -0.095912745 | 0.904087255 | -12.25702978 | 11.795848291594 | -0.033210257 | 0.99766937227783 |
| KRT84 | 0 | 1 | NA | NA | NA | NA |
| KRT85 | 0 | 1 | NA | NA | NA | NA |
| KRT9 | 0.039708936589852 | 0.039708936589852 | -0.777578047 | 1.63822359307613 | 0.621420325001657 | 0.557600959595212 |
| KRTCAP2 | -0.0791861 | 0.9208139 | -0.989403233 | -0.561605584 | -6.32865332 | 0.00523873405719211 |
| KRTDAP | 0 | 1 | NA | NA | NA | NA |
| KTI12 | 0.0426514958736151 | 0.0426514958736151 | -12.32180567 | 12.6917020522336 | 0.0255917386516795 | 0.998861061793165 |
| KTN1 | -0.009411354 | 0.990588646 | -0.362782002 | 0.18046561816192 | -0.586849221 | 0.576865732782607 |
| KYAT3 | -0.029052955 | 0.970947045 | -0.691975981 | 0.141385726621503 | -1.303178077 | 0.336028293303638 |
| KYNU | -0.058099995 | 0.941900005 | -0.914593714 | -0.170801463 | -2.620594239 | 0.082725204963347 |
| L1CAM | -34.88515875 | -33.88515875 | -13.27118066 | 6.92504746660167 | -0.707106781 | 0.539497285386707 |
| L1RE1 | 0 | 1 | NA | NA | NA | NA |
| L2HGDH | 0.580600719562814 | 0.580600719562814 | -8.7409646 | 15.0108224787839 | 0.459062963736588 | 0.65324940354771 |
| L3HYPDH | 0.095982261293392 | 0.095982261293392 | 0.135244006633054 | 1.61916615978734 | 2.47343969917856 | 0.137811885699468 |
| LACC1 | 0 | 1 | NA | NA | NA | NA |
| LACTB | -0.062328614 | 0.937671386 | -1.134940175 | -0.034656483 | -1.938596393 | 0.163764737853643 |
| LACTB2 | -0.068824416 | 0.931175584 | -1.170333298 | -0.052388854 | -2.315351101 | 0.159395442620735 |
| LAD1 | -35.78830405 | -34.78830405 | -16.85809139 | 4.9899418772395 | -1.222409588 | 0.366275438800159 |
| LAGE3 | -36.61888387 | -35.61888387 | -11.96602244 | -9.14029408 | -16.80846767 | 0.00624997235227341 |
| LAIR1 | -36.37460161 | -35.37460161 | -18.36256629 | 0.54386034753371 | -2.120863872 | 0.1803694486231 |
| LAMA2 | 1.05952215827026 | 1.05952215827026 | -4.643651383 | 18.0311013089622 | 1.32440218566766 | 0.359221828802674 |
| LAMA3 | 2.02932729226632 | 2.02932729226632 | -0.333105839 | 21.1067757178735 | 2.17861324281683 | 0.1803694486231 |
| LAMA4 | 0.051993428856593 | 0.051993428856593 | -0.013704774 | 0.971304449547209 | 1.79676550454961 | 0.1803694486231 |
| LAMA5 | 0.0418074649930244 | 0.0418074649930244 | 0.012822641963125 | 0.759663498940957 | 1.80883102910959 | 0.170592731777117 |
| LAMB1 | 0.0816935685101119 | 0.0816935685101119 | 0.368201377768034 | 1.24477407716109 | 3.19456369303055 | 0.0452948939884123 |
| LAMB2 | 0.0508040677818796 | 0.0508040677818796 | 0.0692869564081634 | 0.936788315685394 | 2.39524821890245 | 0.142065785779085 |
| LAMB3 | 1.15463373931293 | 1.15463373931293 | -3.72870513 | 18.8126637473123 | 1.49731565922788 | 0.307719809933799 |
| LAMC1 | 0.0543681048132551 | 0.0543681048132551 | -0.000181977 | 1.0904153154218 | 1.73582598475029 | 0.179354927659756 |
| LAMC2 | 1.17023640972599 | 1.17023640972599 | -3.620040615 | 19.1006495642762 | 1.53011437899737 | 0.299124250438868 |
| LAMP1 | 0.0264014653872261 | 0.0264014653872261 | 0.0351404260273199 | 0.55860584963426 | 1.96381587038351 | 0.13962809743939 |
| LAMP2 | -0.031757835 | 0.968242165 | -0.661293442 | -0.033925509 | -2.116063445 | 0.152039171565611 |
| LAMTOR1 | -0.00903065 | 0.99096935 | -0.679250704 | 0.495449088381055 | -0.274205665 | 0.797861200745264 |
| LAMTOR2 | 0.0176378867139895 | 0.0176378867139895 | -1.00079095 | 1.33106503036264 | 0.261989422153825 | 0.811347541938364 |
| LAMTOR3 | 0.0109962206148342 | 0.0109962206148342 | -0.235488689 | 0.456863973908152 | 0.566355675626404 | 0.591451909222715 |
| LAMTOR4 | -0.003351536 | 0.996648464 | -0.484893722 | 0.422279830759002 | -0.147188975 | 0.916655251130597 |
| LAMTOR5 | 0.0321267910118598 | 0.0321267910118598 | -0.362885737 | 0.928702724768147 | 0.865983068949374 | 0.490378820239143 |
| LANCL1 | 0.0807879141347409 | 0.0807879141347409 | 0.504293520658508 | 1.22694015923991 | 4.18983023933842 | 0.0232301628989628 |
| LANCL2 | 0.0166491253869709 | 0.0166491253869709 | -0.049310278 | 0.35225208363684 | 1.43123125954147 | 0.276620871943393 |
| LAP3 | -0.040365363 | 0.959634637 | -0.82012424 | -0.080581282 | -2.127551802 | 0.119393297483336 |
| LARP1 | -0.081990966 | 0.918009034 | -1.096745436 | -0.560020016 | -6.002215185 | 0.0201118879791381 |
| LARP1B | -36.35477805 | -35.35477805 | -18.12025287 | 0.544712935943199 | -2.118983447 | 0.1803694486231 |
| LARP4 | -1.742251513 | -0.742251513 | -16.64083877 | 3.68230091172517 | -1.106473482 | 0.366275438800159 |
| LARP4B | -2.04362099 | -1.04362099 | -20.51615018 | 0.190353353102065 | -2.207504336 | 0.1803694486231 |
| LARP7 | -0.028220791 | 0.971779209 | -0.510026578 | -0.043857002 | -2.20284676 | 0.129534293750536 |
| LARS1 | -0.004890772 | 0.995109228 | -0.220092067 | 0.122356481576951 | -0.49600631 | 0.633597723009551 |
| LARS2 | -0.015014081 | 0.984985919 | -0.45496618 | 0.166839609955971 | -0.812096668 | 0.498020696391771 |
| LAS1L | -0.043844379 | 0.956155621 | -0.913058445 | 0.126188666841099 | -1.438831956 | 0.274794527015319 |
| LASP1 | 0.0404777692417792 | 0.0404777692417792 | 0.255319606529046 | 0.667459442589351 | 4.38387608647377 | 0.0407413914760697 |
| LAT2 | -2.026582507 | -1.026582507 | -19.82527687 | 0.341588192300247 | -2.173777938 | 0.1803694486231 |
| LAX1 | -36.92617705 | -35.92617705 | -13.44780789 | -12.66880874 | -75.44422726 | 0.000243867488171237 |
| LBP | 0.0328329514865596 | 0.0328329514865596 | -0.163433294 | 0.791033223704968 | 1.16480188254751 | 0.356675249847261 |
| LBR | -0.077808207 | 0.922191793 | -1.140842592 | -0.449239961 | -4.387657512 | 0.0364304456689909 |
| LCAT | -0.879309563 | 0.120690437 | -13.89758351 | 8.61019223843325 | -0.406817544 | 0.683274709407609 |
| LCK | -0.997850009 | 0.002149991 | -18.32322352 | 5.42881611248183 | -1.220657139 | 0.366275438800159 |
| LCLAT1 | -0.383768031 | 0.616231969 | -13.47072078 | 7.4549833949934 | -0.64608066 | 0.567215214842298 |
| LCMT1 | -0.036059017 | 0.963940983 | -11.83018186 | 11.328816772422 | -0.037463216 | 0.996395923375039 |
| LCN2 | -0.205477658 | 0.794522342 | -2.634533632 | -1.350201716 | -6.195349444 | 0.0223067705528745 |
| LCP1 | -0.083933173 | 0.916066827 | -1.550420451 | -0.374354268 | -3.460938676 | 0.0809531445735674 |
| LCP2 | -0.057882937 | 0.942117063 | -1.205870209 | 0.122300536018041 | -1.578600206 | 0.236162241478758 |
| LDAH | -0.495398822 | 0.504601178 | -14.12227553 | 6.25088821917579 | -0.868721226 | 0.509607148662422 |
| LDB3 | 1.89322239365837 | 1.89322239365837 | -1.623415114 | 20.6564693591191 | 1.91726932750806 | 0.207340879703139 |
| LDHA | 0.00818023589482465 | 0.00818023589482465 | -0.204503966 | 0.401690509568059 | 0.608325124518155 | 0.570483755061683 |
| LDHAL6B | 0.54262680189415 | 0.54262680189415 | -8.314436332 | 13.7027992911098 | 0.426455851449539 | 0.669086310925532 |
| LDHB | 0.0182771518898839 | 0.0182771518898839 | -0.072593671 | 0.489326275026987 | 1.50993487717522 | 0.275046503563771 |
| LDHD | -35.8926077 | -34.8926077 | -18.10338941 | 5.34542057993652 | -1.22435347 | 0.366275438800159 |
| LDLR | 0 | 1 | NA | NA | NA | NA |
| LDLRAP1 | 0.0326277199105234 | 0.0326277199105234 | -0.091994557 | 0.664541541864825 | 1.36324288799053 | 0.283386420318423 |
| LEMD2 | 0.0662667421288463 | 0.0662667421288463 | 0.35171616344802 | 1.02726216374142 | 4.23667356560525 | 0.0540427128835424 |
| LEMD3 | -0.02849025 | 0.97150975 | -0.56848074 | 0.0511233015441838 | -1.583868342 | 0.227844271321584 |
| LENG9 | 35.7032813797171 | 35.7032813797171 | -4.68498031 | 15.8739123128937 | 1.2247149052239 | 0.366275438800159 |
| LEO1 | 34.8262841132829 | 34.8262841132829 | -6.648133367 | 12.7405016885676 | 0.707106781186548 | 0.539497285386707 |
| LEPR | -34.75702467 | -33.75702467 | -12.14331777 | 6.33651625744986 | -0.707106781 | 0.539497285386707 |
| LEPROT | 0.617193058040145 | 0.617193058040145 | -7.202371246 | 12.8551226144119 | 0.489361661512767 | 0.636959755614727 |
| LETM1 | -0.027787259 | 0.972212741 | -0.511190601 | -0.044723723 | -2.118304522 | 0.126559938102032 |
| LGALS1 | 0.0467826033310881 | 0.0467826033310881 | 0.305512637817342 | 0.872402949665765 | 3.99208253623173 | 0.044832922345443 |
| LGALS3 | 0.00647265014992577 | 0.00647265014992577 | -0.496488082 | 0.648871811965875 | 0.240834774484237 | 0.829099883084152 |
| LGALS3BP | 0.0766175686000371 | 0.0766175686000371 | 0.562305132787643 | 0.98433035268227 | 6.90361205523755 | 0.0103578874883521 |
| LGALS7B | 0.128523469414909 | 0.128523469414909 | -1.320807362 | 3.8456784130957 | 0.991383923743439 | 0.426073040011617 |
| LGALS8 | 0.452341071411436 | 0.452341071411436 | -6.62086621 | 13.6591869434569 | 0.780761507888794 | 0.539497285386707 |
| LGALS9 | -0.09508891 | 0.90491109 | -1.936931889 | 0.188550473125263 | -1.633289187 | 0.229893724436788 |
| LGALSL | -34.82020824 | -33.82020824 | -12.68695821 | 6.62019379348387 | -0.707106781 | 0.539497285386707 |
| LGMN | -0.08909673 | 0.91090327 | -1.591669954 | -0.144966929 | -2.095851751 | 0.123637539973339 |
| LHPP | 0.0233940032810287 | 0.0233940032810287 | -0.12411078 | 0.60354210172523 | 1.15063060084438 | 0.358702379478436 |
| LIG1 | 0 | 1 | NA | NA | NA | NA |
| LIG3 | 0.00217914121436556 | 0.00217914121436556 | -0.358228116 | 0.396402013066716 | 0.109789129746921 | 0.948868630404795 |
| LILRB1 | -34.71252921 | -33.71252921 | -11.77451133 | 6.14406901377664 | -0.707106781 | 0.539497285386707 |
| LILRB2 | -35.82829264 | -34.82829264 | -17.32709588 | 5.12538380007954 | -1.222932672 | 0.366275438800159 |
| LILRB5 | -0.026170412 | 0.973829588 | -12.98296282 | 12.7494773952604 | -0.015700507 | 0.999130434024431 |
| LIMA1 | 0.0710938025639899 | 0.0710938025639899 | 0.278715450731645 | 1.17142867076085 | 3.52572992998209 | 0.0839188755590668 |
| LIMCH1 | 2.08403686567893 | 2.08403686567893 | 0.232496653099917 | 19.818462324191 | 2.28059941269975 | 0.175024732685753 |
| LIMD1 | -1.067784943 | -0.067784943 | -17.47931086 | 4.41027783223805 | -1.34100616 | 0.355095971803847 |
| LIMD2 | -36.85699321 | -35.85699321 | -13.9526307 | -10.94112948 | -18.60173897 | 0.00498575641984829 |
| LIME1 | -36.99444054 | -35.99444054 | -14.37891197 | -13.0031576 | -44.78900922 | 0.0005434985811995 |
| LIMS1 | 0.0328638621018542 | 0.0328638621018542 | 0.00851205872265082 | 0.643201258814337 | 1.83788365062079 | 0.172764906484805 |
| LIN7A | 0.573589946511974 | 0.573589946511974 | -9.60707397 | 16.3715222848838 | 0.452927886900836 | 0.655402648100291 |
| LIN7C | 0.0527687592919023 | 0.0527687592919023 | 0.302155751793874 | 0.814865038502758 | 3.78160648668622 | 0.0295101905873908 |
| LIPA | -0.485307421 | 0.514692579 | -13.59667369 | 6.14001999700733 | -0.846170837 | 0.519619590473051 |
| LIPE | -34.99885909 | -33.99885909 | -14.35941589 | 7.49290053674267 | -0.707106781 | 0.539497285386707 |
| LITAF | -0.487890048 | 0.512109952 | -13.91312094 | 6.24821690842719 | -0.851493877 | 0.517642586759047 |
| LIX1L | 35.0600799953676 | 35.0600799953676 | -7.817705327 | 14.9818727187107 | 0.707106781186547 | 0.539497285386707 |
| LLGL1 | 0.0783206308102964 | 0.0783206308102964 | 0.587222498199735 | 0.884375683469455 | 8.68609134023846 | 0.00135930837203558 |
| LLGL2 | -35.67362075 | -34.67362075 | -15.55224446 | 4.59099917841072 | -1.224550155 | 0.366275438800159 |
| LLPH | 0.020133989383073 | 0.020133989383073 | -10.79098831 | 10.942542280096 | 0.012066017919517 | 0.999130434024431 |
| LMAN1 | -0.075773027 | 0.924226973 | -1.584166881 | -0.190989789 | -2.841471042 | 0.124895697264244 |
| LMAN2 | -0.092468437 | 0.907531563 | -1.33317786 | -0.722051324 | -5.909287403 | 0.00748736034234612 |
| LMAN2L | 0.0384428124373199 | 0.0384428124373199 | 0.0586243478351357 | 0.62249187174396 | 2.10087541472383 | 0.120980763005907 |
| LMBRD2 | -2.031866907 | -1.031866907 | -17.97935051 | 0.207116638328329 | -2.172606008 | 0.1803694486231 |
| LMCD1 | 0.0183531487519052 | 0.0183531487519052 | -0.200259248 | 0.555558135788746 | 0.950347311340122 | 0.447598170755214 |
| LMF1 | -0.094382456 | 0.905617544 | -1.495693014 | -0.196943541 | -2.540820101 | 0.111972442737426 |
| LMF2 | -0.002530345 | 0.997469655 | -0.546647355 | 0.493190307468061 | -0.109626458 | 0.948868630404795 |
| LMNA | 0.0586674091896342 | 0.0586674091896342 | 0.11459047100937 | 1.3796734159108 | 2.60548203867318 | 0.140196817481724 |
| LMNB1 | -0.079073951 | 0.920926049 | -1.215768426 | -0.575842585 | -5.182904897 | 0.0201240250769653 |
| LMNB2 | 0.00237419398790464 | 0.00237419398790464 | -0.242637483 | 0.298226023330175 | 0.201464822055835 | 0.867982676504852 |
| LMO7 | 0.0991030744095906 | 0.0991030744095906 | 0.113748785267031 | 1.65011870024279 | 2.24657105255418 | 0.14219977567621 |
| LMOD1 | 0.540065507472999 | 0.540065507472999 | -5.885608136 | 14.6955537875405 | 0.962035372109707 | 0.462938981503828 |
| LMOD2 | 0 | 1 | NA | NA | NA | NA |
| LMOD3 | 0 | 1 | NA | NA | NA | NA |
| LNPEP | 0.0149453753782146 | 0.0149453753782146 | -0.165867466 | 0.463626220787183 | 0.946729071992507 | 0.447498117047045 |
| LNPK | 0.0219517767723534 | 0.0219517767723534 | -0.086214716 | 0.51262777224506 | 1.55023848144499 | 0.283900196902605 |
| LONP1 | -0.050152091 | 0.949847909 | -0.900282345 | -0.114253163 | -2.366897243 | 0.109087951789005 |
| LORICRIN | -35.2215229 | -34.2215229 | -16.75580357 | 8.74336188077828 | -0.707106781 | 0.539497285386707 |
| LOX | 0.151983831377728 | 0.151983831377728 | 0.436929303550911 | 2.61967139304833 | 2.54419596893193 | 0.0901822238791405 |
| LOXL1 | -0.019759773 | 0.980240227 | -13.41676219 | 13.2341033727408 | -0.011859138 | 0.999130434024431 |
| LOXL4 | 0 | 1 | NA | NA | NA | NA |
| LPA | -1.98560692 | -0.98560692 | -20.40625719 | 0.705834639039551 | -2.087932451 | 0.180892263409905 |
| LPAR1 | 0.105475080682542 | 0.105475080682542 | 0.42515748746847 | 1.44033234971009 | 3.3899947544214 | 0.0531018349927428 |
| LPCAT1 | -0.434754291 | 0.565245709 | -13.73788999 | 6.89492173470951 | -0.74577197 | 0.539497285386707 |
| LPCAT2 | 0.0147995893131089 | 0.0147995893131089 | -0.348392463 | 0.644737986981218 | 0.518572998894959 | 0.620915125340906 |
| LPCAT3 | -0.011444705 | 0.988555295 | -0.523675585 | 0.307495185204788 | -0.481504823 | 0.646179481380366 |
| LPGAT1 | -0.389007575 | 0.610992425 | -12.68574111 | 6.95242748946249 | -0.655863993 | 0.562031639448264 |
| LPP | 0.0764617043982793 | 0.0764617043982793 | 0.4946610490823 | 1.0356903575309 | 4.99689147250138 | 0.0146699253894409 |
| LPXN | -1.076329348 | -0.076329348 | -19.45496464 | 4.80814885239261 | -1.357331646 | 0.351292861416331 |
| LRBA | -0.031209381 | 0.968790619 | -0.545752135 | -0.039757833 | -2.346018097 | 0.141738434001977 |
| LRCH1 | 0.383565990370605 | 0.383565990370605 | -6.973287157 | 12.5959942721154 | 0.645290079140152 | 0.567664180590699 |
| LRCH3 | -0.380099762 | 0.619900238 | -13.09318016 | 7.29296444719127 | -0.638537751 | 0.570731371819992 |
| LRCH4 | -0.063584991 | 0.936415009 | -0.857198981 | -0.247241603 | -3.203882137 | 0.0499670920464179 |
| LRG1 | 0.0311725616967606 | 0.0311725616967606 | -0.44129431 | 1.04211719597639 | 0.765204427371364 | 0.534281958550569 |
| LRP1 | 0.0891161946292999 | 0.0891161946292999 | 0.530584269742362 | 1.35318089537227 | 4.33261764283124 | 0.0357523135237048 |
| LRPAP1 | 0.0529519282473398 | 0.0529519282473398 | 0.14828543536568 | 0.877312529081062 | 2.47631621750236 | 0.0864259180378225 |
| LRPPRC | -0.034996143 | 0.965003857 | -0.608685584 | -0.12499505 | -3.121281249 | 0.0877829558062199 |
| LRRC1 | 0.520211894456952 | 0.520211894456952 | -8.539975409 | 13.7755545172202 | 0.409398698307865 | 0.681830768898224 |
| LRRC15 | 0.0834787688838821 | 0.0834787688838821 | 0.366058589198898 | 1.41092885702433 | 3.01594066532625 | 0.0564864938788008 |
| LRRC17 | 34.938113730678 | 34.938113730678 | -7.183958129 | 13.767357785565 | 0.707106781186548 | 0.539497285386707 |
| LRRC20 | 1.05477516360243 | 1.05477516360243 | -4.791492568 | 18.3853281086274 | 1.31921407740474 | 0.360722200576953 |
| LRRC25 | -0.085552306 | 0.914447694 | -11.05435549 | 9.97604463925915 | -0.088793993 | 0.965343173934503 |
| LRRC40 | 0.0187344635829388 | 0.0187344635829388 | -0.139460647 | 0.494267813019051 | 0.986798003988213 | 0.396376407 |
| LRRC41 | -36.81565027 | -35.81565027 | -12.67930279 | -11.51120785 | -46.60290419 | 0.000497992726450439 |
| LRRC47 | -0.01399632 | 0.98600368 | -0.367489266 | 0.0811679856456532 | -1.186526934 | 0.360277268145826 |
| LRRC57 | 0.0126889900449869 | 0.0126889900449869 | -0.47220526 | 0.69530055587344 | 0.350532640059986 | 0.734776426462175 |
| LRRC59 | -0.036731162 | 0.963268838 | -0.658558047 | -0.19197923 | -3.336214681 | 0.0531018349927428 |
| LRRC8A | -1.012040538 | -0.012040538 | -14.67708941 | 8.45685532004685 | -0.467874925 | 0.648994611354729 |
| LRRC8E | -34.67151638 | -33.67151638 | -11.44449938 | 5.97186516282972 | -0.707106781 | 0.539497285386707 |
| LRRFIP1 | -0.023609694 | 0.976390306 | -0.424222608 | -0.032816574 | -2.027229355 | 0.130235766873387 |
| LRRFIP2 | 0.0354697978140333 | 0.0354697978140333 | -0.156133036 | 0.76757215818589 | 1.3712246186968 | 0.325768992680793 |
| LRRK1 | 0.00784340649660349 | 0.00784340649660349 | -0.857023795 | 1.00527214169589 | 0.170057862969021 | 0.898980900260294 |
| LRSAM1 | 1.99799441228432 | 1.99799441228432 | -0.597118834 | 19.671513271751 | 2.1171155196875 | 0.1803694486231 |
| LRWD1 | -36.75500398 | -35.75500398 | -12.02786638 | -11.16682961 | -60.61960149 | 0.000297414913898614 |
| LSAMP | 0.153980217883175 | 0.153980217883175 | 0.552780449206534 | 2.26458720768511 | 3.58080026813426 | 0.0822265037287458 |
| LSG1 | -0.429221461 | 0.570778539 | -13.55099898 | 6.87844685291454 | -0.734670832 | 0.539497285386707 |
| LSM1 | 0.99380831612968 | 0.99380831612968 | -5.408698422 | 18.1384370482631 | 1.21222676679182 | 0.366735091564231 |
| LSM12 | 0.0149404893949521 | 0.0149404893949521 | -0.054722606 | 0.335132346855733 | 1.24458105330495 | 0.316916081642846 |
| LSM14A | -0.052722067 | 0.947277933 | -0.988502288 | -0.000518505 | -1.954606739 | 0.179117559639718 |
| LSM14B | 0 | 1 | NA | NA | NA | NA |
| LSM2 | -0.039672227 | 0.960327773 | -0.732821492 | -0.007359882 | -1.914522023 | 0.174982558439639 |
| LSM3 | -0.021036181 | 0.978963819 | -0.592289509 | 0.149857932804747 | -1.151096129 | 0.366275438800159 |
| LSM4 | -0.040173283 | 0.959826717 | -0.894154205 | 0.093087668899362 | -1.796257117 | 0.22670492938347 |
| LSM6 | -0.023359059 | 0.976640941 | -0.50149553 | 0.0310421824239021 | -1.644286914 | 0.205341495400578 |
| LSM7 | -0.017259582 | 0.982740418 | -0.916704352 | 0.553389251407111 | -0.540422437 | 0.625008231843565 |
| LSM8 | 0.372805349722175 | 0.372805349722175 | -8.343892228 | 14.773446047107 | 0.624749852565483 | 0.57827242635708 |
| LSP1 | -0.156742607 | 0.843257393 | -2.441313829 | -0.57512052 | -3.447329025 | 0.0829291783160025 |
| LSR | -36.49937702 | -35.49937702 | -20.07431838 | 0.645906533789226 | -2.110031423 | 0.180428937397777 |
| LSS | -0.00688412 | 0.99311588 | -0.275984959 | 0.136358909597344 | -0.66622549 | 0.546638926108471 |
| LST1 | -1.092089311 | -0.092089311 | -18.78587172 | 4.45027352046883 | -1.384309855 | 0.343283635298893 |
| LTA4H | -0.059198819 | 0.940801181 | -0.815490192 | -0.375006067 | -5.975836348 | 0.0395508495523429 |
| LTB4R | -36.31908969 | -35.31908969 | -17.67233432 | 0.526232125212768 | -2.120187727 | 0.1803694486231 |
| LTBP1 | 0.0947044310247018 | 0.0947044310247018 | 0.380795015501346 | 1.52329118782088 | 3.00784965325771 | 0.0615278226161233 |
| LTBP2 | 0.117238372124211 | 0.117238372124211 | 0.565703248785756 | 1.82059071131266 | 3.39899831785744 | 0.0452948939884123 |
| LTBP3 | 0.0812157128600142 | 0.0812157128600142 | 0.450083587800115 | 1.04450999264283 | 4.8565891630295 | 0.0314747933795631 |
| LTBP4 | 0.0608709818560885 | 0.0608709818560885 | -0.099881344 | 1.24972113231492 | 1.54573681340632 | 0.222691386301682 |
| LTC4S | -0.987390091 | 0.012609909 | -18.4464992 | 5.57439555168728 | -1.201352256 | 0.367336155156715 |
| LTF | -0.207177622 | 0.792822378 | -3.10527827 | -1.487588139 | -5.39621168 | 0.0222025529056802 |
| LTN1 | -0.464523994 | 0.535476006 | -12.7310676 | 6.02706346234201 | -0.801268832 | 0.537375417204127 |
| LTV1 | -36.34948542 | -35.34948542 | -18.04447974 | 0.533298892078757 | -2.121132199 | 0.1803694486231 |
| LUC7L | -1.534033991 | -0.534033991 | -16.93443622 | 4.82992378589731 | -0.962510097 | 0.404394259798308 |
| LUC7L2 | -0.021765449 | 0.978234551 | -0.563410374 | 0.135710301321853 | -1.058499861 | 0.366735091564231 |
| LUC7L3 | -0.441292638 | 0.558707362 | -14.05603295 | 6.96621225339835 | -0.758554511 | 0.539497285386707 |
| LUM | 0.131755276175417 | 0.131755276175417 | 0.720650761014833 | 2.6755923007033 | 3.07328461374917 | 0.054423395199397 |
| LUZP1 | 0.0675304509882576 | 0.0675304509882576 | -0.063797528 | 1.26385388546556 | 1.90595648604822 | 0.19010882411289 |
| LXN | 0.0549423678531108 | 0.0549423678531108 | -0.042951703 | 1.13591697295819 | 1.61300340644245 | 0.187940717985324 |
| LY6D | -1.123124834 | -0.123124834 | -15.30324242 | 8.32170991798496 | -0.51778831 | 0.621617284366183 |
| LY6G6C | 0 | 1 | NA | NA | NA | NA |
| LY6G6F | -0.061813321 | 0.938186679 | -10.11850859 | 9.87120990498166 | -0.021414676 | 0.999130434024431 |
| LY6K | 0 | 1 | NA | NA | NA | NA |
| LY75 | -0.062799607 | 0.937200393 | -1.332805371 | 0.209320342152844 | -1.294048082 | 0.305152177662157 |
| LYAR | -0.409699663 | 0.590300337 | -13.43126142 | 7.07647771374405 | -0.69607228 | 0.544203424467315 |
| LYN | -0.002903635 | 0.997096365 | -0.507979555 | 0.450893431598912 | -0.117149178 | 0.941745859120953 |
| LYPD2 | 0 | 1 | NA | NA | NA | NA |
| LYPD3 | 0.570819102960047 | 0.570819102960047 | -8.960308133 | 15.154138677454 | 0.446686278029506 | 0.657626623414471 |
| LYPLA1 | -0.057662136 | 0.942337864 | -0.774583895 | -0.386951811 | -5.262620535 | 0.0115763926050529 |
| LYPLA2 | -0.007666854 | 0.992333146 | -0.40827573 | 0.259663729239513 | -0.416848 | 0.681281627188306 |
| LYPLAL1 | -0.028740216 | 0.971259784 | -0.473955808 | -0.088270745 | -2.795009653 | 0.0877975327665516 |
| LYRM4 | -0.622974623 | 0.377025377 | -15.26482591 | 8.50044733952584 | -0.494104637 | 0.634031727574291 |
| LYRM7 | 0.011966138075166 | 0.011966138075166 | -10.35773312 | 10.5075998150078 | 0.0124277327972091 | 0.999130434024431 |
| LYSMD2 | -0.481930392 | 0.518069608 | -14.22462115 | 6.47672493839369 | -0.838539551 | 0.52319341260848 |
| LYVE1 | 1.58115540796385 | 1.58115540796385 | -4.458494695 | 16.5732501255092 | 0.996650361295661 | 0.388312991309222 |
| LYZ | -0.120876107 | 0.879123893 | -2.214600697 | -0.419295604 | -2.595433538 | 0.0805192700628092 |
| LZIC | 0.0542129054827439 | 0.0542129054827439 | 0.270119132648152 | 0.758306671182353 | 3.82911845826888 | 0.0383041704369688 |
| LZTFL1 | 0.66118917711203 | 0.66118917711203 | -7.986200333 | 14.9319580165505 | 0.525429835881866 | 0.616550083670876 |
| M6PR | -0.04079562 | 0.95920438 | -0.923567 | 0.126319079340241 | -1.364436096 | 0.281932107446751 |
| MAB21L4 | -34.99862917 | -33.99862917 | -14.35712761 | 7.4917064853388 | -0.707106781 | 0.539497285386707 |
| MACF1 | 0.0285492112606612 | 0.0285492112606612 | -0.021320211 | 0.625160891981765 | 1.62052937693193 | 0.185822782336519 |
| MACROD1 | 0.015213589899542 | 0.015213589899542 | -0.329382609 | 0.615321390881888 | 0.554096190670498 | 0.602556984583984 |
| MACROH2A1 | -0.015251996 | 0.984748004 | -0.684419969 | 0.33761736077102 | -0.623339248 | 0.561015630932865 |
| MACROH2A2 | 0.0511875684587381 | 0.0511875684587381 | 0.260821196033486 | 0.922773065676762 | 3.1895293763662 | 0.0525595373037303 |
| MAD1L1 | -0.05110079 | 0.94889921 | -1.179203487 | 0.271143228668175 | -1.111428507 | 0.366275438800159 |
| MAD2L1 | -36.53465079 | -35.53465079 | -20.518855 | 0.609565350857984 | -2.120483139 | 0.1803694486231 |
| MAEA | -34.89896575 | -33.89896575 | -13.39879929 | 6.99164026557058 | -0.707106781 | 0.539497285386707 |
| MAG | 0.0415739124542799 | 0.0415739124542799 | -11.00321681 | 11.187943884487 | 0.0144059280401669 | 0.999130434024431 |
| MAGED1 | -0.986697778 | 0.013302222 | -14.45861765 | 8.44247339946774 | -0.456542689 | 0.654169551195503 |
| MAGED2 | 0.0301917317562511 | 0.0301917317562511 | -0.091968863 | 0.648380781143782 | 1.41777787298351 | 0.279498586365108 |
| MAGT1 | -0.099381073 | 0.900618927 | -1.446356377 | -0.717362108 | -5.136774857 | 0.0103578874883521 |
| MAIP1 | -0.036526292 | 0.963473708 | -0.609899659 | -0.020426196 | -2.189450629 | 0.164702566587332 |
| MAK16 | -0.435326459 | 0.564673541 | -12.14114063 | 6.08364091481276 | -0.745991091 | 0.539497285386707 |
| MAL2 | -34.8770986 | -33.8770986 | -13.19724307 | 6.88646602207211 | -0.707106781 | 0.539497285386707 |
| MALT1 | -0.62137308 | 0.37862692 | -14.78505297 | 8.24566428411968 | -0.492946341 | 0.6344490471806 |
| MAMDC2 | 36.9557882999596 | 36.9557882999596 | 12.6852967424806 | 13.972901138385 | 46.5901837962621 | 0.000497992726450439 |
| MAN1A1 | -0.119788328 | 0.880211672 | -1.584001482 | -0.744586178 | -5.193619339 | 0.0218015022926752 |
| MAN1A2 | -2.125074805 | -1.125074805 | -18.93172735 | -0.525246243 | -2.376454933 | 0.168462283243728 |
| MAN1B1 | 0.435753079367641 | 0.435753079367641 | -6.568266534 | 13.117245034485 | 0.747158050706812 | 0.539497285386707 |
| MAN1C1 | 0.508797393683339 | 0.508797393683339 | -7.464680831 | 17.3580573670404 | 0.895735711115651 | 0.495719493985637 |
| MAN2A1 | -0.045846526 | 0.954153474 | -0.860631765 | -0.027759165 | -1.859074193 | 0.161407722878652 |
| MAN2B1 | -0.067005889 | 0.932994111 | -1.145665433 | -0.161938307 | -2.577251987 | 0.10761361335075 |
| MAN2B2 | 0.0697509295396853 | 0.0697509295396853 | -0.186415219 | 1.4011618445677 | 1.35805424750786 | 0.279498586365108 |
| MAN2C1 | 0.010841247693192 | 0.010841247693192 | -0.321597728 | 0.567879798069374 | 0.540526932697385 | 0.616983638982579 |
| MANBA | 0 | 1 | NA | NA | NA | NA |
| MANBAL | 0.115175157501106 | 0.115175157501106 | 0.435643898045098 | 1.58300319168854 | 3.36340669788964 | 0.0611905113435923 |
| MANEA | -36.55013187 | -35.55013187 | -20.73893356 | 0.614853649587727 | -2.120738934 | 0.1803694486231 |
| MANF | -0.136790862 | 0.863209138 | -1.716744627 | -1.143325735 | -9.260316656 | 0.0032255225249016 |
| MAOA | -0.416050248 | 0.583949752 | -13.65059784 | 7.09528944748156 | -0.707511588 | 0.539497285386707 |
| MAOB | -0.059256928 | 0.940743072 | -1.206871816 | 0.0731714488973223 | -1.809927132 | 0.199089626473623 |
| MAP1A | 0.0967013904658172 | 0.0967013904658172 | 0.650002317159122 | 1.26321557635386 | 5.43142840810452 | 0.00928406583063397 |
| MAP1B | 0.115017535608372 | 0.115017535608372 | 0.611933028577203 | 1.68857061252432 | 3.81252511795144 | 0.034944397588941 |
| MAP1LC3A | 35.7879936720404 | 35.7879936720404 | -4.968125942 | 16.8337224392587 | 1.22473561606728 | 0.366275438800159 |
| MAP1S | 0.00920671550105298 | 0.00920671550105298 | -0.110811134 | 0.291673855930804 | 0.777726194920877 | 0.517049179673688 |
| MAP2K1 | -0.007166638 | 0.992833362 | -0.274524558 | 0.140529454362348 | -0.675773378 | 0.547473602821171 |
| MAP2K2 | 0.0055119250620554 | 0.0055119250620554 | -0.476416922 | 0.584001164669357 | 0.183910032466137 | 0.882115393202798 |
| MAP2K3 | 0.00506515901709729 | 0.00506515901709729 | -0.294579422 | 0.38917082286612 | 0.248676196018719 | 0.821512918961126 |
| MAP2K4 | 0.0129129951200108 | 0.0129129951200108 | -0.830317898 | 1.06845655003821 | 0.262925290038446 | 0.815769250044788 |
| MAP2K6 | 0.0286751194468452 | 0.0286751194468452 | -10.84941408 | 11.2297097654351 | 0.0298045660743175 | 0.998435102357591 |
| MAP3K20 | 0.551606477844261 | 0.551606477844261 | -5.664326515 | 14.5198188904476 | 0.986619369877977 | 0.450866419056598 |
| MAP3K4 | -0.010234746 | 0.989765254 | -11.80724468 | 11.7237873069977 | -0.006136716 | 0.999130434024431 |
| MAP3K6 | -35.79092551 | -34.79092551 | -16.86802602 | 4.97829184075409 | -1.224728951 | 0.366275438800159 |
| MAP3K7 | 0.481531371403754 | 0.481531371403754 | -5.828433763 | 12.7835015149922 | 0.840185681938559 | 0.523094221217977 |
| MAP4 | 0.097395331073405 | 0.097395331073405 | 0.694657041553491 | 1.37097504810809 | 5.70601302927839 | 0.0163865667283959 |
| MAP4K1 | -0.933063352 | 0.066936648 | -17.11636611 | 5.75930149029433 | -1.109707881 | 0.393885324509724 |
| MAP4K4 | 1.69037209052682 | 1.69037209052682 | -4.132352323 | 17.5563097330193 | 1.0722896578933 | 0.366275438800159 |
| MAP4K5 | 1.03121684282239 | 1.03121684282239 | -4.972916825 | 18.0678438094833 | 1.27782634106176 | 0.366275438800159 |
| MAP7 | -35.75950176 | -34.75950176 | -16.50445886 | 4.87089827898559 | -1.22474412 | 0.366275438800159 |
| MAP7D1 | 0.00507229170059974 | 0.00507229170059974 | -0.401845559 | 0.495115347184286 | 0.199515861905467 | 0.868881446764451 |
| MAPK1 | 0.0519327663337529 | 0.0519327663337529 | 0.407488984437347 | 0.731525183844006 | 6.61847752908572 | 0.0119780099521387 |
| MAPK13 | -1.038137385 | -0.038137385 | -18.75754103 | 5.08068584061853 | -1.288613685 | 0.366275438800159 |
| MAPK14 | -0.014508637 | 0.985491363 | -0.289550603 | 0.0152823766614224 | -1.57464387 | 0.200644118003916 |
| MAPK3 | 0.0400154664147174 | 0.0400154664147174 | 0.26977890552408 | 0.544317524759693 | 6.3799895980517 | 0.0316815233111557 |
| MAPK9 | 0.0329406929907879 | 0.0329406929907879 | 0.141428089185237 | 0.478310285449974 | 3.1868929479458 | 0.0452282221099089 |
| MAPKAPK2 | -0.401200637 | 0.598799363 | -13.0350568 | 6.95378240798484 | -0.675831548 | 0.553039963762948 |
| MAPRE1 | -0.019779172 | 0.980220828 | -0.483749606 | 0.0616627086751836 | -1.399345749 | 0.270798111321858 |
| MAPRE2 | -0.381866448 | 0.618133552 | -14.52621263 | 8.07428856077362 | -0.642195611 | 0.569057716596462 |
| MAPRE3 | -36.55364844 | -35.55364844 | -20.79138839 | 0.61819612053568 | -2.120374026 | 0.1803694486231 |
| MAPT | 0 | 1 | NA | NA | NA | NA |
| MARCHF5 | 0.0110717601074233 | 0.0110717601074233 | -0.523093446 | 0.720348889573165 | 0.296923312202716 | 0.78120353302249 |
| MARCKS | 0.0415437695028869 | 0.0415437695028869 | 0.179787301137108 | 0.680652015070655 | 3.00812148831504 | 0.0545448167689707 |
| MARCKSL1 | -0.000222153 | 0.999777847 | -0.481109325 | 0.476979726316024 | -0.007605309 | 0.999130434024431 |
| MARCO | 0 | 1 | NA | NA | NA | NA |
| MARK2 | -0.046104559 | 0.953895441 | -11.14947688 | 10.5522055681314 | -0.047623545 | 0.992135986860447 |
| MARK3 | -0.036694272 | 0.963305728 | -11.61662637 | 11.1157930458578 | -0.038125827 | 0.996318639344248 |
| MARS1 | -0.052870634 | 0.947129366 | -0.738150178 | -0.276261856 | -3.810971459 | 0.0289973150604434 |
| MASP1 | 1.02839035546761 | 1.02839035546761 | -8.409724232 | 14.7349366220873 | 0.475882036518104 | 0.645403310754965 |
| MASP2 | 1.60506442970884 | 1.60506442970884 | -4.757304111 | 18.2207537090868 | 1.01383485413811 | 0.381391477592172 |
| MAST4 | 0 | 1 | NA | NA | NA | NA |
| MAT2A | -0.019226346 | 0.980773654 | -0.497051251 | 0.109423041477523 | -1.185834377 | 0.360245704961941 |
| MAT2B | -0.020809219 | 0.979190781 | -0.346885545 | -0.066364228 | -2.931885573 | 0.0896916084940673 |
| MATN1 | 0.614985487380431 | 0.614985487380431 | -5.573516703 | 16.857072619279 | 1.11776312583761 | 0.390056596414302 |
| MATN2 | 0.579678629871672 | 0.579678629871672 | -10.02338735 | 17.1931347972684 | 0.458123437674218 | 0.653774096383628 |
| MATN4 | 0 | 1 | NA | NA | NA | NA |
| MATR3 | -0.038104831 | 0.961895169 | -0.607974884 | -0.208641636 | -3.645346577 | 0.0385666087989108 |
| MAU2 | -0.957618058 | 0.042381942 | -13.91896134 | 8.25876528736563 | -0.443019741 | 0.6590499433979 |
| MAVS | -0.014803149 | 0.985196851 | -0.349452804 | 0.0745189336564506 | -1.137956754 | 0.363301226496658 |
| MAX | -1.923415427 | -0.923415427 | -21.34285878 | 1.38300747860012 | -1.974021625 | 0.197739541290329 |
| MB | -0.013782071 | 0.986217929 | -12.27454509 | 12.0735342246728 | -0.014284477 | 0.999130434024431 |
| MBD1 | -2.038794253 | -1.038794253 | -18.63180079 | 0.18324509081111 | -2.191904494 | 0.1803694486231 |
| MBD2 | -0.047235075 | 0.952764925 | -0.805606237 | -0.062583463 | -2.261198058 | 0.136597230826191 |
| MBD3 | 34.83816053 | 35.83816053 | -6.703087382 | 12.8458157195436 | 0.707106781186548 | 0.539497285386707 |
| MBL2 | 0.128661668040558 | 0.128661668040558 | -10.3557184 | 10.9015992914079 | 0.0445170291198132 | 0.993220185640509 |
| MBLAC2 | 0.0120182831660716 | 0.0120182831660716 | -0.406395821 | 0.619724509824767 | 0.376820876908243 | 0.711138518579206 |
| MBNL1 | -1.635645562 | -0.635645562 | -15.91277077 | 4.01495006184108 | -1.033393083 | 0.374314121532703 |
| MBOAT7 | -0.025520545 | 0.974479455 | -0.688007221 | 0.193111803375052 | -1.152034245 | 0.36731973075278 |
| MBP | 0.0159949021127808 | 0.0159949021127808 | -12.64461523 | 12.8876608032547 | 0.0164704742971452 | 0.999130434024431 |
| MCAM | 0.0491910816301454 | 0.0491910816301454 | -0.038358231 | 1.0141714309661 | 1.71260404836073 | 0.186464286475205 |
| MCAT | -1.045467907 | -0.045467907 | -17.61187604 | 4.69235478528663 | -1.302795441 | 0.363817806593495 |
| MCCC1 | -0.047291093 | 0.952708907 | -0.683399063 | -0.234832746 | -3.555047449 | 0.0353293394402478 |
| MCCC2 | -0.060953922 | 0.939046078 | -0.782690349 | -0.433205577 | -6.330012423 | 0.00947903194156041 |
| MCEE | -0.10376886 | 0.89623114 | -1.467134643 | -0.51736874 | -3.643237786 | 0.0337544610971382 |
| MCEMP1 | -36.91705447 | -35.91705447 | -13.93731778 | -12.01467681 | -30.37516428 | 0.00142412998339416 |
| MCL1 | -36.7977267 | -35.7977267 | -12.80711735 | -11.08471761 | -31.21487807 | 0.00134576390418309 |
| MCM2 | -0.069869883 | 0.930130117 | -1.617093344 | 0.409506525667277 | -1.034685495 | 0.37438739649163 |
| MCM3 | -0.112151876 | 0.887848124 | -1.642856463 | -0.37991059 | -2.935071076 | 0.0698765466924951 |
| MCM4 | -2.026136578 | -1.026136578 | -19.92661047 | 0.33336148165725 | -2.170441966 | 0.1803694486231 |
| MCM5 | -0.019541092 | 0.980458908 | -0.519802564 | 0.160593787713722 | -0.958024971 | 0.418688240405903 |
| MCM6 | -1.116134554 | -0.116134554 | -17.33591182 | 3.86459868926516 | -1.426124951 | 0.33021351282907 |
| MCM7 | -2.067976495 | -1.067976495 | -19.28741775 | -0.039114413 | -2.256431606 | 0.179117559639718 |
| MCMBP | 0.0615293547590977 | 0.0615293547590977 | -0.80317431 | 1.97266777099687 | 0.923366905716957 | 0.477654864213928 |
| MCRIP1 | 0.0552754663046514 | 0.0552754663046514 | -10.26448153 | 10.9680631676624 | 0.0573511491486327 | 0.987378189240374 |
| MCTS1 | -0.002119202 | 0.997880798 | -0.238631133 | 0.192680400902321 | -0.205385557 | 0.863661105923864 |
| MCU | -0.019743838 | 0.980256162 | -0.504461865 | 0.0983217705584361 | -1.287737207 | 0.332391787611187 |
| MCUR1 | -36.84680447 | -35.84680447 | -13.16902276 | -11.54954966 | -34.34754755 | 0.00104930801944221 |
| MDC1 | 0.0137942869401412 | 0.0137942869401412 | -0.653854253 | 0.890659544020261 | 0.269967097379753 | 0.801829697302329 |
| MDH1 | -0.006874668 | 0.993125332 | -0.380931475 | 0.226163856929158 | -0.533225459 | 0.625689848851569 |
| MDH2 | -0.0474026 | 0.9525974 | -0.881384845 | -0.200714885 | -3.356037686 | 0.0841851891763818 |
| MDK | 0.525757402156507 | 0.525757402156507 | -5.992674856 | 14.4700152677653 | 0.931555899244348 | 0.477932819265767 |
| MDN1 | 0.352780600577728 | 0.352780600577728 | -7.092447864 | 12.1129543500552 | 0.58706442591702 | 0.600281717789629 |
| MDP1 | -0.037158817 | 0.962841183 | -0.5630832 | -0.137772819 | -2.890941955 | 0.0596811273470357 |
| ME1 | 0.0348709627378651 | 0.0348709627378651 | -0.273938398 | 0.939622576312627 | 0.970206207228539 | 0.405365987267894 |
| ME2 | -0.094996578 | 0.905003422 | -1.421938076 | -0.560413804 | -3.992939726 | 0.0255994622577781 |
| ME3 | 1.57330353841283 | 1.57330353841283 | -4.729964022 | 17.4336417719666 | 0.99174194621119 | 0.390098642797282 |
| MEAK7 | 0.513508337218248 | 0.513508337218248 | -6.090377823 | 14.319424137534 | 0.902871810052233 | 0.491705776536746 |
| MECP2 | -0.044221948 | 0.955778052 | -0.867931829 | -0.027114736 | -2.037587612 | 0.164566197052059 |
| MECR | -1.031600553 | -0.031600553 | -18.28923561 | 5.02973088954169 | -1.278488852 | 0.366275438800159 |
| MED1 | 0.0143923683446701 | 0.0143923683446701 | -0.489360788 | 0.746084621321261 | 0.359758189736098 | 0.722749591103998 |
| MED15 | -34.84991498 | -33.84991498 | -12.95090534 | 6.75792429496301 | -0.707106781 | 0.539497285386707 |
| MED16 | -0.508463667 | 0.491536333 | -13.76335685 | 8.63665379609053 | -0.399583028 | 0.689301717179151 |
| MED17 | -0.008226354 | 0.991773646 | -11.28694813 | 11.2498130198318 | -0.002851018 | 0.999376962148702 |
| MED20 | 0.632886797784516 | 0.632886797784516 | -9.184085663 | 16.6645250926752 | 0.502178812396975 | 0.629519640440438 |
| MED21 | -34.76794467 | -33.76794467 | -12.23558135 | 6.38466040184828 | -0.707106781 | 0.539497285386707 |
| MED23 | -0.011443348 | 0.988556652 | -0.581325884 | 0.374562898129388 | -0.387078464 | 0.701781000264582 |
| MED24 | -0.030766554 | 0.969233446 | -10.27245688 | 9.89967529759445 | -0.031978074 | 0.998435102357591 |
| MED30 | -0.402317293 | 0.597682707 | -12.3527994 | 6.605387045 | -0.68127062 | 0.551534708253067 |
| MED4 | 1.56165622329413 | 1.56165622329413 | -4.634150969 | 16.8406992788999 | 0.983539105294618 | 0.393933863628188 |
| MEF2C | -0.398986686 | 0.601013314 | -14.94021912 | 8.04260151335666 | -0.675183311 | 0.554353627193476 |
| MEF2D | -0.136464235 | 0.863535765 | -1.776538874 | -0.559298082 | -4.080017267 | 0.0615278226161233 |
| MEGF6 | 0.0896398434033385 | 0.0896398434033385 | 0.163196909463477 | 1.47466338165456 | 2.30263446160525 | 0.117192419255221 |
| MELTF | 1.14172853637049 | 1.14172853637049 | -3.900151479 | 18.8552566273459 | 1.47463310553891 | 0.315108462496261 |
| MEMO1 | 0.0336837509646151 | 0.0336837509646151 | -0.38103401 | 0.997336795922065 | 0.801124349483596 | 0.509188064289834 |
| MEN1 | -0.416267483 | 0.583732517 | -13.19534841 | 6.89615675183077 | -0.705319858 | 0.540028981078929 |
| MEPCE | -34.81479448 | -33.81479448 | -12.63943922 | 6.59539786399532 | -0.707106781 | 0.539497285386707 |
| MEPE | -36.61532113 | -35.61532113 | -21.72591833 | 0.671659617647555 | -2.115363398 | 0.1803694486231 |
| MESD | -0.070733827 | 0.929266173 | -1.080425369 | -0.425788718 | -4.938354513 | 0.0478015473611303 |
| MET | 0 | 1 | NA | NA | NA | NA |
| METAP1 | -0.428153396 | 0.571846604 | -13.88115495 | 7.05122097021739 | -0.731497709 | 0.539497285386707 |
| METAP2 | 0.0203660807794769 | 0.0203660807794769 | -0.198817194 | 0.566151476344888 | 0.847213380884746 | 0.478042854830948 |
| METTL1 | -0.511401287 | 0.488598713 | -13.40479423 | 5.73363038375788 | -0.901777244 | 0.49304168959344 |
| METTL13 | -0.006402263 | 0.993597737 | -0.464856308 | 0.357999314785829 | -0.288511857 | 0.796279557751275 |
| METTL14 | -0.377486565 | 0.622513435 | -13.33608144 | 7.46390043842523 | -0.633562864 | 0.57361626965992 |
| METTL16 | -0.930980534 | 0.069019466 | -19.59493602 | 6.64523198714494 | -1.110108389 | 0.394572995101535 |
| METTL3 | -34.97175749 | -33.97175749 | -14.09218639 | 7.35345725352662 | -0.707106781 | 0.539497285386707 |
| METTL7A | -0.091233349 | 0.908766651 | -1.211411346 | -0.72768855 | -6.957629718 | 0.00306401850664402 |
| METTL7B | 1.7534412049743 | 1.7534412049743 | -3.579347443 | 16.4168474705663 | 1.11447809848099 | 0.366275438800159 |
| MEX3A | 0.116257762888937 | 0.116257762888937 | -10.91059832 | 12.5432022654555 | 0.120625715366831 | 0.936760076765164 |
| MFAP1 | -0.084412399 | 0.915587601 | -1.244134819 | -0.290859754 | -2.980234664 | 0.0702628828293677 |
| MFAP2 | 0.146838202743217 | 0.146838202743217 | 0.79741005120656 | 2.48273323728408 | 3.70478406681609 | 0.0503461979643727 |
| MFAP4 | 0.458890415973266 | 0.458890415973266 | -7.214720579 | 15.0937225961055 | 0.792457134694271 | 0.539497285386707 |
| MFAP5 | 0.216266895979321 | 0.216266895979321 | -0.192634528 | 4.33372231938587 | 1.61960094941705 | 0.19283643768128 |
| MFF | -0.043297465 | 0.956702535 | -0.810609612 | 0.0409302583516653 | -1.574576352 | 0.199089626473623 |
| MFGE8 | 0.206885694780477 | 0.206885694780477 | 0.759330972945535 | 3.1987948151006 | 2.81024276505681 | 0.0605364962388785 |
| MFN1 | -0.012044595 | 0.987955405 | -0.646973947 | 0.433156869619416 | -0.370982486 | 0.718396675321089 |
| MFN2 | -0.006255339 | 0.993744661 | -0.304644708 | 0.187527480652358 | -0.485833436 | 0.648994611354729 |
| MFSD1 | -0.42819896 | 0.57180104 | -13.81661779 | 7.01642675451149 | -0.731358085 | 0.539497285386707 |
| MFSD10 | 0.0151674263054288 | 0.0151674263054288 | -0.483834155 | 0.787028010133974 | 0.432585855284965 | 0.667845084393481 |
| MGAM | -34.71942946 | -33.71942946 | -11.83096243 | 6.17352581738717 | -0.707106781 | 0.539497285386707 |
| MGAT1 | -0.053051732 | 0.946948268 | -1.082829815 | 0.0869246126395236 | -1.727999606 | 0.2143374716663 |
| MGAT2 | -0.082384033 | 0.917615967 | -1.101356845 | -0.497576943 | -4.592860616 | 0.0159729447348982 |
| MGLL | -0.02425411 | 0.97574589 | -0.899578661 | 0.491793959761643 | -0.606227574 | 0.581348471052249 |
| MGMT | -0.062350072 | 0.937649928 | -0.975189546 | -0.179393048 | -2.521405796 | 0.0805192700628092 |
| MGP | -0.04292761 | 0.95707239 | -1.619122836 | 0.778358565985766 | -0.609407617 | 0.562359213415735 |
| MGST1 | -0.638542944 | 0.361457056 | -15.4283599 | 8.45044075915482 | -0.506992741 | 0.627348453175941 |
| MGST2 | -0.016829017 | 0.983170983 | -0.744971196 | 0.405745838933033 | -0.62673631 | 0.5723102731992 |
| MGST3 | 0.0436067827568411 | 0.0436067827568411 | -0.842435563 | 1.70975066181153 | 0.721166278327734 | 0.539497285386707 |
| MIA2 | -0.501648145 | 0.498351855 | -13.27931691 | 5.78342504981766 | -0.878749637 | 0.503680318364621 |
| MIA3 | 0.00483160573166407 | 0.00483160573166407 | -0.24569316 | 0.337981326856158 | 0.27363919580919 | 0.797861200745264 |
| MIB1 | -0.694882253 | 0.305117747 | -13.39291789 | 6.91039974090329 | -0.553009462 | 0.597338595226894 |
| MICAL1 | -0.032811563 | 0.967188437 | -1.036225861 | 0.404537431371769 | -0.900627388 | 0.47766170964528 |
| MICALL1 | -1.037267227 | -0.037267227 | -14.86324243 | 8.44298591212489 | -0.47991575 | 0.642981031952378 |
| MICALL2 | -35.84415549 | -34.84415549 | -17.50233181 | 5.16571806607543 | -1.224694859 | 0.366275438800159 |
| MICOS10 | -0.432839537 | 0.567160463 | -13.64654649 | 6.89477924669628 | -0.739076667 | 0.539497285386707 |
| MICOS13 | -0.002558069 | 0.997441931 | -0.871867077 | 0.82687587713933 | -0.057970531 | 0.987958171023924 |
| MICU1 | 0 | 1 | NA | NA | NA | NA |
| MID1 | 0.527737144034044 | 0.527737144034044 | -5.944214353 | 14.4244033537117 | 0.936697794131384 | 0.475727178647703 |
| MIDEAS | -1.056707645 | -0.056707645 | -15.33897114 | 8.62989142736982 | -0.488252584 | 0.637535164767707 |
| MIEF1 | -36.5297148 | -35.5297148 | -20.44882627 | 0.607537207152239 | -2.120472295 | 0.1803694486231 |
| MIEN1 | -36.52147817 | -35.52147817 | -20.33546297 | 0.60712906710338 | -2.119854552 | 0.1803694486231 |
| MIER1 | -0.376761736 | 0.623238264 | -12.10992299 | 6.79002951143446 | -0.631090842 | 0.574986493138539 |
| MIF | 0.0515870904555796 | 0.0515870904555796 | 0.256185805994164 | 0.993802348798979 | 3.11076132335441 | 0.0612951020097337 |
| MIF4GD | -34.50754103 | -33.50754103 | -10.21492779 | 5.33026123704893 | -0.707106781 | 0.539497285386707 |
| MINDY1 | 0.236531126712464 | 0.236531126712464 | -10.92028352 | 11.9929419287906 | 0.0815200803985429 | 0.970298134767008 |
| MINDY2 | 1.08989631036683 | 1.08989631036683 | -7.869098718 | 14.2387357935339 | 0.503563016033767 | 0.62918291865217 |
| MINDY3 | 0.390543115800787 | 0.390543115800787 | -6.880811671 | 12.5882620226303 | 0.659221994918813 | 0.561015630932865 |
| MINK1 | -36.7932513 | -35.7932513 | -12.59639848 | -11.22143605 | -38.98140887 | 0.000748555873716068 |
| MINPP1 | 0.486662965971081 | 0.486662965971081 | -6.201511833 | 13.75488226 | 0.848789727053601 | 0.518665703646773 |
| MIOS | -0.378612871 | 0.621387129 | -12.94520323 | 7.23387168568351 | -0.636078929 | 0.5723102731992 |
| MIPEP | -35.88705613 | -34.88705613 | -18.06494098 | 5.35597119802609 | -1.221103929 | 0.366275438800159 |
| MISP | 0 | 1 | NA | NA | NA | NA |
| MIX23 | -0.044679957 | 0.955320043 | -0.660685867 | -0.163069409 | -3.258435841 | 0.0729045590054497 |
| MKI67 | -34.90142873 | -33.90142873 | -13.42169339 | 7.00358666204709 | -0.707106781 | 0.539497285386707 |
| MLEC | -0.015860568 | 0.984139432 | -0.413504724 | 0.0775752126879685 | -1.340841933 | 0.320026974451544 |
| MLF2 | -0.965613533 | 0.034386467 | -17.95607991 | 5.69037026369194 | -1.166753688 | 0.3766467818649 |
| MLIP | 0 | 1 | NA | NA | NA | NA |
| MLKL | -0.513709327 | 0.486290673 | -13.75806578 | 5.85650862797808 | -0.906164736 | 0.49124461671169 |
| MLLT1 | -36.43557085 | -35.43557085 | -19.1644619 | 0.57658742360673 | -2.118876208 | 0.1803694486231 |
| MLYCD | -2.0658112 | -1.0658112 | -19.66189127 | -0.016842404 | -2.252653826 | 0.179117559639718 |
| MMAA | 0.0396288286369686 | 0.0396288286369686 | 0.00670771203791097 | 0.73665566749406 | 2.08656991944559 | 0.175707003568027 |
| MMAB | -0.428089604 | 0.571910396 | -13.33255939 | 6.78185558512458 | -0.732263401 | 0.539497285386707 |
| MME | -0.475613544 | 0.524386456 | -13.83533232 | 6.38612364874952 | -0.826346297 | 0.528014169078056 |
| MMGT1 | -35.71804856 | -34.71804856 | -16.04000657 | 4.73595844814647 | -1.224387478 | 0.366275438800159 |
| MMP1 | 0 | 1 | NA | NA | NA | NA |
| MMP10 | 36.0066834483207 | 36.0066834483207 | -5.814774841 | 19.6224880483338 | 1.22151123752262 | 0.366275438800159 |
| MMP12 | 35.913062644721 | 35.913062644721 | -5.42952418 | 18.3696675426639 | 1.22355351029274 | 0.366275438800159 |
| MMP13 | 0.926977101013524 | 0.926977101013524 | -5.983606558 | 17.5243611307047 | 1.10312834307472 | 0.397249255515765 |
| MMP14 | 0.0775520263404549 | 0.0775520263404549 | 0.294737572598902 | 1.2807548911211 | 3.11586340103156 | 0.0765716370351513 |
| MMP2 | 0.126087265571851 | 0.126087265571851 | 0.72855918396775 | 1.73389743984294 | 4.25681419743949 | 0.0209277986139644 |
| MMP3 | -1.582258771 | -0.582258771 | -18.56001545 | 4.98144978088277 | -0.997984969 | 0.387557496341511 |
| MMP8 | -37.00373562 | -36.00373562 | -14.57746849 | -12.9815895 | -38.86068922 | 0.000748679488460952 |
| MMP9 | -0.209255035 | 0.790744965 | -2.679835973 | -1.467360272 | -6.209451622 | 0.00974692033665045 |
| MMRN1 | 0.0568335670159365 | 0.0568335670159365 | 0.107852572398106 | 0.916481924999583 | 2.25536800866774 | 0.111343306872831 |
| MMRN2 | 0.0200346606157079 | 0.0200346606157079 | -0.51477697 | 0.848621459524472 | 0.509748338381519 | 0.637496335882231 |
| MMS19 | -0.025394482 | 0.974605518 | -0.429602565 | -0.034470047 | -2.09366325 | 0.130235766873387 |
| MMTAG2 | -36.76783483 | -35.76783483 | -11.87075921 | -11.53114269 | -155.0630875 | 0.000135517346157358 |
| MMUT | -0.030797762 | 0.969202238 | -0.573776744 | 0.00112997347956352 | -1.945709487 | 0.179833016962564 |
| MNAT1 | 0.0277314571779703 | 0.0277314571779703 | -12.99163191 | 13.2439333772121 | 0.0166406174127532 | 0.999130434024431 |
| MNDA | -0.08757732 | 0.91242268 | -1.565839199 | -0.224179786 | -2.358601358 | 0.0989774487129094 |
| MOB2 | -0.043797475 | 0.956202525 | -11.55899751 | 10.9664971781442 | -0.045521292 | 0.992838135314568 |
| MOB3A | -2.068443307 | -1.068443307 | -20.11567575 | -0.046625856 | -2.257111267 | 0.178994347526889 |
| MOB4 | -1.023649446 | -0.023649446 | -19.28848878 | 5.40535745969944 | -1.263658224 | 0.366275438800159 |
| MOCS3 | 0.0398207264898653 | 0.0398207264898653 | 0.184246714751428 | 0.55124191001877 | 3.77718877251599 | 0.0462481886440984 |
| MOGS | -0.029420401 | 0.970579599 | -0.559745072 | -0.073003088 | -2.249469658 | 0.102932593123907 |
| MON2 | -0.023324181 | 0.976675819 | -0.51297031 | 0.0803677317097847 | -1.311481176 | 0.302485340561127 |
| MORC2 | -34.95946259 | -33.95946259 | -13.97260061 | 7.29105608289155 | -0.707106781 | 0.539497285386707 |
| MORC3 | -36.50595016 | -35.50595016 | -20.2235808 | 0.706448115206854 | -2.098419307 | 0.180925617554127 |
| MORF4L1 | -0.946948228 | 0.053051772 | -13.65193361 | 8.14419187102963 | -0.43842882 | 0.66180921540292 |
| MORF4L2 | -36.00743582 | -35.00743582 | -19.60484402 | 5.78992816884945 | -1.224193332 | 0.366275438800159 |
| MOSPD2 | 0 | 1 | NA | NA | NA | NA |
| MOV10 | 0.0157802760463261 | 0.0157802760463261 | -0.088204141 | 0.384947783816731 | 1.14274665714221 | 0.366275438800159 |
| MOV10L1 | 0.0603713842313079 | 0.0603713842313079 | -0.171667349 | 1.19486102462189 | 1.3407934003429 | 0.290078274907324 |
| MOXD1 | 0.137357737279392 | 0.137357737279392 | 0.413656897120924 | 2.3287870261486 | 2.84926477942303 | 0.0950403565941456 |
| MPC1 | 0.551800952880063 | 0.551800952880063 | -9.752095053 | 16.2555866432872 | 0.435543391961349 | 0.663582139480957 |
| MPC2 | -0.011914723 | 0.988085277 | -0.86272694 | 0.646588110311225 | -0.253823032 | 0.817090366166205 |
| MPDU1 | -0.085878487 | 0.914121513 | -1.33302764 | -0.372775375 | -3.081861456 | 0.0497268643178361 |
| MPEG1 | -36.90015906 | -35.90015906 | -13.46873591 | -12.1811074 | -44.82705599 | 0.0005434985811995 |
| MPG | -0.00766207 | 0.99233793 | -0.297180844 | 0.149349123688334 | -0.649533406 | 0.554393952209213 |
| MPI | -0.449595567 | 0.550404433 | -13.092214 | 6.36194290847859 | -0.773092691 | 0.539497285386707 |
| MPIG6B | -0.017021553 | 0.982978447 | -11.40766011 | 11.3301403305569 | -0.00589905 | 0.999130434024431 |
| MPO | -0.217162275 | 0.782837725 | -3.3112217 | -1.447749972 | -4.48460483 | 0.0196658587740803 |
| MPP1 | -0.017398095 | 0.982601905 | -0.811709078 | 0.467952608473042 | -0.472767063 | 0.647590537137961 |
| MPP7 | -0.00399141 | 0.99600859 | -11.93279915 | 11.9137339159715 | -0.001383315 | 0.999577493567163 |
| MPRIP | 0.0990486338213892 | 0.0990486338213892 | 0.485325208161002 | 1.34178778281357 | 3.78464942088953 | 0.03422145439256 |
| MPST | 0.0131829191226668 | 0.0131829191226668 | -0.189348946 | 0.457326827734046 | 0.755100266880329 | 0.534470832811491 |
| MPV17 | -0.01519751 | 0.98480249 | -12.68733981 | 12.554280366553 | -0.009121037 | 0.999130434024431 |
| MPZ | 0.00184018301133003 | 0.00184018301133003 | -2.630017504 | 2.66682356753433 | 0.01202987832404 | 0.999130434024431 |
| MPZL1 | -0.304216113 | 0.695783887 | -13.35324239 | 8.5080094361911 | -0.497267699 | 0.648456915230199 |
| MPZL2 | -34.81661385 | -33.81661385 | -12.65538872 | 6.60372048527168 | -0.707106781 | 0.539497285386707 |
| MRC1 | 0.0298781690026582 | 0.0298781690026582 | -0.109805289 | 0.686045813964235 | 1.36967245756332 | 0.297846198381887 |
| MRC2 | 0.122339602149998 | 0.122339602149998 | 0.659197436523956 | 1.81919291245844 | 4.63660992530063 | 0.0540427128835424 |
| MRE11 | -0.024262696 | 0.975737304 | -0.528751045 | 0.0759919828099757 | -1.306995957 | 0.294403235671687 |
| MRFAP1 | -36.89281909 | -35.89281909 | -12.98828005 | -12.53139656 | -125.6943583 | 0.000152272230019378 |
| MRGBP | -0.45275632 | 0.54724368 | -12.76010007 | 6.18929863909063 | -0.779624626 | 0.539497285386707 |
| MRGPRF | 1.12785247911543 | 1.12785247911543 | -4.06753223 | 18.9309615439274 | 1.44962216202389 | 0.323079459136434 |
| MRI1 | -0.035948309 | 0.964051691 | -0.590788933 | -0.116302127 | -2.586580044 | 0.0753698265741615 |
| MRM3 | 0.0685856329465987 | 0.0685856329465987 | -0.42116233 | 1.67235108266546 | 1.03527655166041 | 0.373738817295334 |
| MROH1 | 0.00919148449033522 | 0.00919148449033522 | -0.755731001 | 0.908510320328634 | 0.173359041979534 | 0.893394969909098 |
| MRPL1 | -1.667356191 | -0.667356191 | -17.58597199 | 4.2659109681223 | -1.055570533 | 0.36731973075278 |
| MRPL10 | -0.664553972 | 0.335446028 | -13.54877209 | 7.21705691137271 | -0.5285622 | 0.614533004164038 |
| MRPL11 | -0.471120623 | 0.528879377 | -14.24010847 | 6.63644854029735 | -0.818760682 | 0.531490813258076 |
| MRPL12 | -0.484925545 | 0.515074455 | -15.00616646 | 6.79492418584792 | -0.847246917 | 0.519619590473051 |
| MRPL13 | -1.036617766 | -0.036617766 | -19.01209306 | 5.17130508118923 | -1.287507398 | 0.366275438800159 |
| MRPL14 | -0.450555283 | 0.549444717 | -15.43619057 | 7.50966024991929 | -0.777044308 | 0.539497285386707 |
| MRPL15 | -0.032010602 | 0.967989398 | -0.951433596 | 0.395462436470255 | -0.735349734 | 0.539497285386707 |
| MRPL16 | -0.615841092 | 0.384158908 | -15.60628449 | 8.75596314229172 | -0.488262456 | 0.637428610542048 |
| MRPL17 | -0.067909387 | 0.932090613 | -0.953661986 | -0.331698645 | -3.771397934 | 0.0400932328388338 |
| MRPL18 | -0.06253758 | 0.93746242 | -0.870869031 | -0.290232694 | -3.60906665 | 0.0411660223126904 |
| MRPL19 | -0.082464175 | 0.917535825 | -1.063580661 | -0.466489383 | -4.464136945 | 0.0184565795377057 |
| MRPL2 | -0.067980343 | 0.932019657 | -1.057607787 | -0.213643511 | -2.931125597 | 0.0845612712882046 |
| MRPL20 | -0.892471361 | 0.107528639 | -18.64627244 | 6.78899202146778 | -1.048547982 | 0.420656783472752 |
| MRPL21 | -0.432344612 | 0.567655388 | -15.20213986 | 7.66898078938423 | -0.740908025 | 0.539497285386707 |
| MRPL22 | -0.050070096 | 0.949929904 | -0.848234096 | -0.104143803 | -2.276368572 | 0.108627734565813 |
| MRPL23 | -0.040922646 | 0.959077354 | -0.768066344 | -0.019896801 | -1.867228327 | 0.165981324839939 |
| MRPL24 | -0.406739533 | 0.593260467 | -14.31554414 | 7.58894994098939 | -0.690449591 | 0.54733731056867 |
| MRPL27 | 0.60570601377574 | 0.60570601377574 | -9.071828137 | 15.9989948264471 | 0.479991638166596 | 0.642680309295651 |
| MRPL28 | -1.03844802 | -0.03844802 | -14.05675572 | 7.98061343039688 | -0.480385041 | 0.642680309295651 |
| MRPL3 | -0.047289762 | 0.952710238 | -0.675554106 | -0.191036207 | -3.117351117 | 0.0494267633366755 |
| MRPL30 | -36.23184028 | -35.23184028 | -16.6408639 | 0.500968381081593 | -2.11879805 | 0.1803694486231 |
| MRPL37 | -0.061222465 | 0.938777535 | -0.860936447 | -0.309962341 | -3.69061152 | 0.0318429534811337 |
| MRPL38 | -0.045026784 | 0.954973216 | -0.638158713 | -0.264286196 | -4.585970175 | 0.0324314269886733 |
| MRPL39 | -0.034944376 | 0.965055624 | -0.908984691 | 0.270304577671885 | -0.993646422 | 0.402643473413631 |
| MRPL4 | -1.015521927 | -0.015521927 | -18.81421812 | 5.36535187596095 | -1.250910665 | 0.366275438800159 |
| MRPL40 | -2.038417963 | -1.038417963 | -19.6532941 | 0.2192665653651 | -2.195246436 | 0.1803694486231 |
| MRPL41 | -1.591991231 | -0.591991231 | -18.03137202 | 4.78232007977768 | -1.004835847 | 0.384558053356644 |
| MRPL43 | -0.089179898 | 0.910820102 | -1.334868 | -0.357537254 | -3.038307957 | 0.0538679412155676 |
| MRPL44 | -0.435476298 | 0.564523702 | -13.92197529 | 6.97876622202853 | -0.747236094 | 0.539497285386707 |
| MRPL45 | -0.441532189 | 0.558467811 | -14.54178734 | 7.20306994340622 | -0.75935924 | 0.539497285386707 |
| MRPL46 | -0.463527296 | 0.536472704 | -13.87939235 | 6.58009745762118 | -0.801519035 | 0.537375417204127 |
| MRPL47 | -0.43400558 | 0.56599442 | -14.34116801 | 7.21977940269412 | -0.743210894 | 0.539497285386707 |
| MRPL48 | -0.015883456 | 0.984116544 | -0.47765835 | 0.192731854264546 | -0.753147016 | 0.532167832158021 |
| MRPL49 | -0.054240154 | 0.945759846 | -1.061016708 | 0.0538183072181373 | -1.773343528 | 0.193186504889997 |
| MRPL50 | -0.067419979 | 0.932580021 | -1.17082662 | -0.074204343 | -2.310075726 | 0.147005931601574 |
| MRPL53 | -2.036223525 | -1.036223525 | -20.03094761 | 0.25659165747217 | -2.193101595 | 0.1803694486231 |
| MRPL54 | -1.980937488 | -0.980937488 | -21.64976616 | 0.825238439011167 | -2.08349565 | 0.182063758237489 |
| MRPL55 | -0.451017911 | 0.548982089 | -13.22762636 | 6.43036147612241 | -0.777475384 | 0.539497285386707 |
| MRPL58 | -0.058738602 | 0.941261398 | -1.026084447 | -0.083224437 | -2.160480575 | 0.131038262594095 |
| MRPL9 | -0.443656058 | 0.556343942 | -15.07441381 | 7.43488602635981 | -0.763414004 | 0.539497285386707 |
| MRPS11 | -1.06805084 | -0.06805084 | -18.15415756 | 4.54553740165516 | -1.337482844 | 0.354826113544616 |
| MRPS14 | -1.01150328 | -0.01150328 | -17.237806 | 4.95329115710125 | -1.24295262 | 0.366275438800159 |
| MRPS15 | -0.4897744 | 0.5102256 | -13.89602179 | 6.22614659195464 | -0.855806713 | 0.515814391238517 |
| MRPS16 | -0.551282228 | 0.448717772 | -15.5049499 | 6.00960530091316 | -0.982380437 | 0.450866419056598 |
| MRPS17 | -1.057958403 | -0.057958403 | -17.98201809 | 4.64832072457546 | -1.324441851 | 0.359451193776443 |
| MRPS18A | -1.038833628 | -0.038833628 | -19.59001552 | 5.30343951784615 | -1.291442773 | 0.366275438800159 |
| MRPS18B | -1.043959918 | -0.043959918 | -19.58605751 | 5.23849319183892 | -1.300388209 | 0.364559456625742 |
| MRPS2 | -0.064354307 | 0.935645693 | -0.888855165 | -0.363883422 | -4.193992741 | 0.0242841266519135 |
| MRPS21 | -0.982986491 | 0.017013509 | -18.16325326 | 5.55035829302499 | -1.195263958 | 0.369001682738711 |
| MRPS22 | -0.048962778 | 0.951037222 | -0.742567026 | -0.171830445 | -3.138705111 | 0.0765716370351513 |
| MRPS23 | -0.04560597 | 0.95439403 | -0.780620405 | -0.084971902 | -2.344161011 | 0.119490662174419 |
| MRPS26 | -1.05628945 | -0.05628945 | -19.3000137 | 5.00906110740166 | -1.321396607 | 0.360245704961941 |
| MRPS27 | -0.069314452 | 0.930685548 | -1.155338662 | -0.147317804 | -2.442641053 | 0.111433687454078 |
| MRPS28 | -0.482472209 | 0.517527791 | -14.56252061 | 6.63008569694161 | -0.841591717 | 0.522548220095479 |
| MRPS30 | -1.039867436 | -0.039867436 | -16.12282676 | 4.34292230973602 | -1.291855836 | 0.366275438800159 |
| MRPS31 | -0.467620727 | 0.532379273 | -13.86472148 | 6.50887916903381 | -0.811026285 | 0.534281958550569 |
| MRPS33 | -1.711414702 | -0.711414702 | -16.77474571 | 3.8807603425626 | -1.082412613 | 0.366275438800159 |
| MRPS34 | -0.064761844 | 0.935238156 | -0.89901044 | -0.334958145 | -3.79853503 | 0.0292840823068327 |
| MRPS35 | -0.498577547 | 0.501422453 | -12.64505403 | 5.56506085781566 | -0.872868195 | 0.507195209981315 |
| MRPS36 | -0.010250356 | 0.989749644 | -0.670887329 | 0.477688433279949 | -0.296847213 | 0.778725814650526 |
| MRPS5 | -0.472320611 | 0.527679389 | -14.54709394 | 6.76833035814602 | -0.820769038 | 0.531094722652365 |
| MRPS6 | -1.02527925 | -0.02527925 | -19.1131734 | 5.3424204280641 | -1.265695126 | 0.366275438800159 |
| MRPS7 | -0.05723599 | 0.94276401 | -0.931151981 | -0.162115806 | -2.486341053 | 0.0841851891763818 |
| MRPS9 | -0.451566246 | 0.548433754 | -13.63466084 | 6.62696965338607 | -0.777161748 | 0.539497285386707 |
| MRRF | -0.43085401 | 0.56914599 | -14.58792696 | 7.3859374102222 | -0.737241907 | 0.539497285386707 |
| MRTFA | 0 | 1 | NA | NA | NA | NA |
| MRTO4 | -0.457379445 | 0.542620555 | -14.97274922 | 7.1820089386624 | -0.791036448 | 0.539497285386707 |
| MS4A1 | -1.110864899 | -0.110864899 | -18.91345163 | 4.27375238487782 | -1.419936417 | 0.333192165343881 |
| MSH2 | -0.007825321 | 0.992174679 | -0.513840259 | 0.375093349491689 | -0.286352405 | 0.789075852792094 |
| MSH6 | 1.03052625500998 | 1.03052625500998 | -5.116647112 | 18.6017618819275 | 1.27528133370544 | 0.366275438800159 |
| MSI2 | 0.00231523454184325 | 0.00231523454184325 | -0.507568968 | 0.551353903951046 | 0.0788738752835693 | 0.972765179053006 |
| MSMO1 | -34.61369347 | -33.61369347 | -10.99487681 | 5.73724718173881 | -0.707106781 | 0.539497285386707 |
| MSN | -0.000411733 | 0.999588267 | -0.282041854 | 0.272437148697695 | -0.030025504 | 0.998435102357591 |
| MSR1 | -0.394148167 | 0.605851833 | -12.73146792 | 6.89755699562665 | -0.664503882 | 0.558631800775978 |
| MSRA | 0.0639498781581644 | 0.0639498781581644 | -0.429409678 | 1.57222935629865 | 1.00014079185258 | 0.389202761398707 |
| MSRB2 | -0.422299243 | 0.577700757 | -13.80417636 | 7.09906501145983 | -0.720563752 | 0.539497285386707 |
| MSRB3 | 0.0813872036896336 | 0.0813872036896336 | 0.379068674473594 | 1.23101801296017 | 3.5266688297214 | 0.051055672178882 |
| MST1 | 0.0918480926474799 | 0.0918480926474799 | 0.542575753648521 | 1.21476666175545 | 4.56259819886649 | 0.0172642638532251 |
| MT-ATP6 | -0.041358536 | 0.958641464 | -0.917552283 | 0.126031166046487 | -1.40784186 | 0.277198186035603 |
| MT-ATP8 | -0.465591931 | 0.534408069 | -14.30676479 | 6.74289174400629 | -0.807261871 | 0.535425881029537 |
| MT-CO1 | -0.036480277 | 0.963519723 | -11.29725864 | 10.8136768913633 | -0.037846047 | 0.996395923375039 |
| MT-CO2 | -0.046766568 | 0.953233432 | -0.787128542 | -0.275503621 | -3.929200423 | 0.0434144250591685 |
| MT-CO3 | 0 | 1 | NA | NA | NA | NA |
| MT-CYB | -0.433137951 | 0.566862049 | -13.53313686 | 6.81310383229036 | -0.742444992 | 0.539497285386707 |
| MT-ND1 | -0.61666555 | 0.38333445 | -16.05482779 | 8.99898009144733 | -0.489027143 | 0.637060379747688 |
| MT-ND2 | 0 | 1 | NA | NA | NA | NA |
| MT-ND3 | -0.054584279 | 0.945415721 | -1.20690743 | 0.201444005683208 | -1.423760093 | 0.296555862907277 |
| MT-ND4 | -0.045502226 | 0.954497774 | -0.766700923 | -0.118585766 | -2.436725797 | 0.0936910072024128 |
| MT-ND5 | -36.946887 | -35.946887 | -13.82619779 | -12.66802774 | -51.47839347 | 0.000421976602842467 |
| MT-ND6 | -35.8183228 | -34.8183228 | -17.19352431 | 5.07584222822066 | -1.224497148 | 0.366275438800159 |
| MTA1 | 0.00146157674071732 | 0.00146157674071732 | -0.42265325 | 0.449461215191864 | 0.0570117581651976 | 0.987911517811832 |
| MTA2 | -0.039657918 | 0.960342082 | -0.614524146 | -0.174177928 | -3.099221787 | 0.0487319717290352 |
| MTAP | 0.0412380934226792 | 0.0412380934226792 | 0.130830621884277 | 0.686225202828078 | 3.02770403126799 | 0.0926613647437377 |
| MTARC2 | -0.335051621 | 0.664948379 | -12.810704 | 7.73757202709379 | -0.553557423 | 0.619956495708675 |
| MTCH1 | 0.0592385893138867 | 0.0592385893138867 | 0.142600662533993 | 1.01810044486177 | 2.34722139642768 | 0.100763636811655 |
| MTCH2 | -0.017923891 | 0.982076109 | -0.69631036 | 0.321050704388149 | -0.753583573 | 0.539497285386707 |
| MTDH | -0.087540642 | 0.912459358 | -1.182473131 | -0.725955428 | -8.35968643 | 0.0109939244517726 |
| MTFP1 | -1.659282532 | -0.659282532 | -16.84801335 | 4.11863945042651 | -1.051139853 | 0.367582922952445 |
| MTFR1L | -1.002371444 | -0.002371444 | -16.9825225 | 4.98055226229459 | -1.226529015 | 0.366275438800159 |
| MTHFD1 | -0.017328332 | 0.982671668 | -0.371878172 | 0.00642418692788485 | -1.676656042 | 0.1803694486231 |
| MTHFD1L | -0.060847103 | 0.939152897 | -0.777175953 | -0.324987672 | -4.25319755 | 0.0218015022926752 |
| MTHFD2 | -37.01686981 | -36.01686981 | -14.32098761 | -13.49011163 | -75.32307414 | 0.000243867488171237 |
| MTIF2 | -0.072406242 | 0.927593758 | -1.099515267 | -0.237268207 | -3.020081021 | 0.0810869445560843 |
| MTM1 | -0.423538347 | 0.576461653 | -13.70643375 | 7.01892030112765 | -0.721230294 | 0.539497285386707 |
| MTMR1 | 0.381341959195195 | 0.381341959195195 | -6.954184897 | 12.5105077689695 | 0.641130983266788 | 0.569611555591851 |
| MTMR12 | -2.04352817 | -1.04352817 | -17.42474963 | 0.144687781281621 | -2.203713405 | 0.1803694486231 |
| MTMR14 | -0.455498671 | 0.544501329 | -12.48505895 | 6.00568906226307 | -0.786054649 | 0.539497285386707 |
| MTMR2 | 1.65592635415438 | 1.65592635415438 | -3.768268087 | 15.3511947527882 | 1.04882027092033 | 0.368275328443468 |
| MTMR3 | -0.337351546 | 0.662648454 | -12.92444793 | 7.77113939771207 | -0.556149743 | 0.618161624117539 |
| MTMR6 | -0.002980299 | 0.997019701 | -0.471727627 | 0.419377602850333 | -0.112060659 | 0.945186096678396 |
| MTMR9 | 35.9259344123209 | 35.9259344123209 | -5.466609144 | 18.5227214163585 | 1.22473416849114 | 0.366275438800159 |
| MTOR | 0.0171442086752396 | 0.0171442086752396 | -0.007065853 | 0.318819430869069 | 1.66089668822322 | 0.1803694486231 |
| MTPN | 0.0153376477731364 | 0.0153376477731364 | -0.148075708 | 0.463579784549965 | 0.897798130378674 | 0.444085436766949 |
| MTR | 0.0883680598272803 | 0.0883680598272803 | -11.31002673 | 12.5758666264726 | 0.0917768641394376 | 0.9623155826899 |
| MTREX | -0.029439157 | 0.970560843 | -0.51200829 | -0.050590855 | -2.109841868 | 0.118370583821899 |
| MTX1 | -0.055896705 | 0.944103295 | -1.013898494 | -0.043049268 | -2.287659799 | 0.160257194908202 |
| MTX2 | -0.366836629 | 0.633163371 | -13.90863422 | 7.95488728079759 | -0.612041983 | 0.585817099552445 |
| MTX3 | 0.0305854741507748 | 0.0305854741507748 | -0.200050859 | 0.736968049775437 | 1.27281178686921 | 0.366275438800159 |
| MUC1 | -1.695308892 | -0.695308892 | -17.49555691 | 4.10147359244335 | -1.074643818 | 0.366275438800159 |
| MUC21 | 0 | 1 | NA | NA | NA | NA |
| MUC4 | 0 | 1 | NA | NA | NA | NA |
| MUC5AC | 1.64393006094911 | 1.64393006094911 | -4.539326607 | 18.201891030671 | 1.03994160144579 | 0.371925820869984 |
| MUC5B | 0.714651875281865 | 0.714651875281865 | -9.951266369 | 20.1707396688515 | 0.763127873786696 | 0.539497285386707 |
| MUSTN1 | 34.6180019233851 | 34.6180019233851 | -5.754406481 | 11.0277609433629 | 0.707106781186548 | 0.539497285386707 |
| MVB12A | -35.90760569 | -34.90760569 | -18.28940279 | 5.39811272139103 | -1.224680932 | 0.366275438800159 |
| MVD | -0.011887352 | 0.988112648 | -0.434077588 | 0.21478354191061 | -0.608980264 | 0.566398099030184 |
| MVK | 0 | 1 | NA | NA | NA | NA |
| MVP | 0.000956722560686182 | 0.000956722560686182 | -0.323110566 | 0.344070198469547 | 0.0553891439798715 | 0.988399409038841 |
| MX1 | 0.0021798204559015 | 0.0021798204559015 | -0.996179893 | 1.03860371994722 | 0.0366626622594487 | 0.996797192456917 |
| MX2 | 0.0363234141509787 | 0.0363234141509787 | -0.184209785 | 0.902054132255191 | 1.22363646563474 | 0.347637007430169 |
| MXRA5 | 0.0876856159031528 | 0.0876856159031528 | 0.197599697276931 | 1.53325544385688 | 2.81319551992628 | 0.119866482067661 |
| MXRA7 | 0.0265270591759624 | 0.0265270591759624 | -0.351200448 | 0.86577480533977 | 0.873527279426854 | 0.493086542348739 |
| MXRA8 | 0.110149254656594 | 0.110149254656594 | 0.511844797461617 | 1.56206099050266 | 4.005057004 | 0.0548717713947671 |
| MYADM | 0.0646212532596859 | 0.0646212532596859 | 0.115920179649241 | 1.24715810574088 | 2.1657167067965 | 0.124570854411906 |
| MYBBP1A | -0.072103678 | 0.927896322 | -1.056153586 | -0.330474717 | -3.386886801 | 0.0437718992653409 |
| MYBPC1 | 36.0379783049733 | 36.0379783049733 | -6.045906559 | 20.1564080363224 | 1.211850033 | 0.366735091564231 |
| MYBPC2 | 0 | 1 | NA | NA | NA | NA |
| MYBPH | -34.89502561 | -33.89502561 | -13.36225587 | 6.97257150949096 | -0.707106781 | 0.539497285386707 |
| MYCBP | -0.499327505 | 0.500672495 | -14.32777126 | 6.29327863085886 | -0.876711703 | 0.50575395903854 |
| MYCBP2 | -0.455595837 | 0.544404163 | -12.72947949 | 6.13197295597354 | -0.786660348 | 0.539497285386707 |
| MYD88 | 0.0176990267994202 | 0.0176990267994202 | -0.008008511 | 0.338892004573117 | 1.68316054266789 | 0.1803694486231 |
| MYDGF | -0.093380325 | 0.906619675 | -1.194408547 | -0.675413269 | -6.422233975 | 0.00707266612384511 |
| MYG1 | -0.018138114 | 0.981861886 | -0.484759033 | 0.145637380505965 | -0.945479752 | 0.417816748040054 |
| MYH1 | 0 | 1 | NA | NA | NA | NA |
| MYH10 | 0.181328290624038 | 0.181328290624038 | 0.987853808873265 | 2.84996101772328 | 3.61572755361278 | 0.0357523135237048 |
| MYH11 | 0.114179333048896 | 0.114179333048896 | 0.300516919663813 | 1.90254308966642 | 2.39624275885278 | 0.0896916084940673 |
| MYH13 | 0 | 1 | NA | NA | NA | NA |
| MYH14 | 0.4825782889683 | 0.4825782889683 | -7.508499252 | 16.4980880297313 | 0.84200605652073 | 0.52237446039615 |
| MYH2 | -0.115062181 | 0.884937819 | -15.54661421 | 14.3731260543435 | -0.06803497 | 0.979715024510606 |
| MYH3 | 0 | 1 | NA | NA | NA | NA |
| MYH4 | -0.038960459 | 0.961039541 | -14.28040371 | 13.8996234064199 | -0.023383695 | 0.999130434024431 |
| MYH6 | 0 | 1 | NA | NA | NA | NA |
| MYH7 | 0.0287892536645518 | 0.0287892536645518 | -11.08257748 | 11.2111140133159 | 0.00997676735938517 | 0.999130434024431 |
| MYH8 | 0 | 1 | NA | NA | NA | NA |
| MYH9 | 0.0403542310181705 | 0.0403542310181705 | 0.0533976062613842 | 0.893199581708714 | 1.98068132494417 | 0.14219977567621 |
| MYL1 | 0.0260812197263448 | 0.0260812197263448 | -2.032225624 | 2.54600584762763 | 0.195855299325285 | 0.869136021744139 |
| MYL11 | -0.319241957 | 0.680758043 | -14.00369009 | 8.67615242000225 | -0.518426055 | 0.636761008502395 |
| MYL2 | -1.575561528 | -0.575561528 | -20.03134524 | 5.43125739036401 | -0.992104618 | 0.390098642797282 |
[truncated: 327,991 more chars]
